# Development and Applications of Chromosome-Specific Cytogenetic BAC-FISH Probes in *S. spontaneum*

**DOI:** 10.3389/fpls.2018.00218

**Published:** 2018-02-26

**Authors:** Guangrui Dong, Jiao Shen, Qing Zhang, Jianping Wang, Qingyi Yu, Ray Ming, Kai Wang, Jisen Zhang

**Affiliations:** ^1^Fujian Provincial Key Laboratory of Haixia Applied Plant Systems Biology, Center for Genomics and Biotechnology, Haixia Institute of Science and Technology, College of Life Sciences, Fujian Agriculture and Forestry University, Fuzhou, China; ^2^Key Laboratory of Sugarcane Biology and Genetic Breeding, Ministry of Agriculture, Fujian Agriculture and Forestry University, Fuzhou, China; ^3^College of Life Sciences, Fujian Normal University, Fuzhou, China; ^4^Agronomy Department, University of Florida, Gainesville, FL, United States; ^5^Department of Plant Biology, University of Illinois at Urbana-Champaign, Urbana, IL, United States; ^6^Texas A&M AgriLife Research Center, Department of Plant Pathology and Microbiology, Texas A&M University System, Dallas, TX, United States

**Keywords:** *Saccharum spontaneum*, sorghum, polyploidy, bacterial artificial chromosome (BAC), fluorescence *in situ* hybridization (FISH)

## Abstract

*Saccharum spontaneum* is a major *Saccharum* species that contributed to the origin of modern sugarcane cultivars, and due to a high degree of polyploidy is considered to be a plant species with one of the most complex genetics. Fluorescence *in situ* hybridization (FISH) is a powerful and widely used tool in genome studies. Here, we demonstrated that FISH based on bacterial artificial chromosome (BAC) clones can be used as a specific cytological marker to identify *S. spontaneum* individual chromosomes and study the relationship between *S. spontaneum* and other related species. We screened low-copy BACs as probes from the sequences of a high coverage of *S. spontaneum* BAC library based on BLAST search of the sorghum genome. In total, we isolated 49 positive BAC clones, and identified 27 BAC clones that can give specific signals on the *S. spontaneum* chromosomes. Of the 27 BAC probes, 18 were confirmed to be able to discriminate the eight basic chromosomes of *S. spontaneum*. Moreover, BAC-24, BAC-66, BAC-78, BAC-69, BAC-71, BAC-73, and BAC-77 probes were used to construct physical maps of chromosome 1 and chromosome 2 of *S. spontaneum*, which indicated synteny in Sb01 between *S. spontaneum* and sorghum. Furthermore, we found that BAC-14 and BAC-19 probes, corresponding to the sorghum chromosomes 2 and 8, respectively, localized to different arms of the same *S. spontaneum* chromosome, suggesting that there was an inter-chromosomal rearrangement event between *S. spontaneum* and sorghum. Our study provides the first set of chromosome-specific cytogenetic markers in *Saccharum* and is critical for future advances in cytogenetics and genome sequencing studies in *Saccharum*.

## Introduction

Sugarcane (*Saccharum* spp., *Poaceae*) is an annual or perennial crop grown in tropical and subtropical regions worldwide. Sugarcane is an important crop for sucrose production, and accounts for 70% of the world sugar production. In addition, as a C4 plant, sugarcane can efficiently convert solar energy into chemical energy, and is therefore an ideal biofuel crop for ethanol production (Lam et al., 2009; Santchurn et al., 2014). The genus *Saccharum* has six species: *S. spontaneum*, *S. robustum*, *S. officinarum*, *S. barberi*, *S. sinense*, and *S. edule*, among which, *S. spontaneum* is considered the wild species with a basic chromosome set of *x* = 8. *S. spontaneum* is also one of the two main *Saccharum* species contributing to the modern sugarcane cultivars development (Dhont et al., 1998; Ha et al., 1999). *S. spontaneum* has a strong environmental adaptability and contains important genetic traits for disease and drought resistance, thus contributing to the stress tolerance of modern cultivar hybrids (Grivet et al., 2004). *S. spontaneum* has the widest geographic distribution, and its ploidy levels range from 2*n* = 5*x* = 40 to 2*n* = 16*x* = 128 (Panje and Babu, 1960).

Sugarcane chromosomes have similar morphologies and have a small size of 1–6 μm at the condensed metaphase stage (Ha et al., 1999; Dhont, 2005); hence, it is very challenging to discern between the different chromosomes based on traditional cytogenetic methods. Moreover, as an autopolyploid, its chromosomal structural alterations caused by polyploidization, duplication, deletion, and recombination are very common (Piperidis et al., 2010). In *S. spontaneum*, genetic linkage maps have been developed (Silva et al., 1995), but development of a cytological genetic map lagged behind other grass species. Reliable cytological tools to identify individual chromosomes can be used for effective genome research and germplasm resource utilization, specifically with the complex genome background of modern sugarcane cultivars. Chromosome-specific bacterial artificial chromosome (BAC) clones are invaluable resource for sugarcane genome researches, and will have many applications in physical mapping, chromosome identification, and marker-assisted breeding of the *Saccharum* spp., and in assisting sugarcane genome sequencing and assembly projects.

Fluorescence *in situ* hybridization (FISH) is a powerful tool for molecular cytology (Jiang and Gill, 2006). In *Saccharum* spp., 45S rDNA and 5S rDNA were used as probes to detect the basic chromosomes of *S. spontaneum*, *S. robustum*, and *S. officinarum*, to identify the different basic chromosomes, *x* = 8 in *S. spontaneum*, and *x* = 10 in *S. robustum* and *S. officinarum* (Dhont et al., 1998; Ha et al., 1999). Based on FISH, modern sugarcane cultivars were found to contain 70–80% of the chromosomes derived from *S. officinarum*, and 10–23% of chromosomes from *S. spontaneum*, while 5–17% appears to be the product of the recombination between *S. spontaneum* and *S. officinarum* (Dhont et al., 1996; Piperidis et al., 2001; Cuadrado et al., 2004). Moreover, chromosome elimination, recombination, and translocation events were detected in some progenitors derived from the hybridization between *Saccharum* and *Erianthus arundinaceus* using FISH (Dhont et al., 1995; Huang et al., 2015).

Sorghum has a small diploid genome (730 Mbp) with a low frequency of chromosome recombination events. Sorghum shares high synteny with the sugarcane genome, making it an ideal reference plant for comparative analyses with the sugarcane genome (Ming et al., 1998; Nazeema et al., 2007; Wang et al., 2010; Aitken et al., 2014). In this study, based on the available sequences for high coverage BAC resources, reliable BAC probes were developed for FISH chromosome identification and cytogenetic map construction. The objectives of this study were to: (1) develop a set of chromosome-specific BAC-FISH probes on *S. spontaneum* chromosome and (2) explore the chromosome rearrangement between *S. spontaneum* and sorghum.

## Materials and Methods

### Materials

*Saccharum spontaneum* SES208 (2*n* = 8*x* = 64) was used for cytological analyses in this study. The SES208 plants were grown in the field on the campus of Fujian Agricultural and Forestry University (Fuzhou, China) in February of 2015 and maintained under regular sugarcane growth conditions.

A BAC library was constructed from the haploid genome of *S. spontaneum* AP85-441 (4*x* = 32), which was derived from *S. spontaneum* SES208 (2*n* = 8*x* = 64) via anther *in vitro* culture. The genome size of AP85-441 is about 3.2 Gbp. The library consisted of 38,400 clones, with an average insert size of 100 kb, covering 6× of the whole genome. This BAC library has been used for identifying the sucrose transporters and fructokinase gene families in *S. spontaneum* (Zhang et al., 2016; Chen et al., 2017).

### Screening the BAC Library

35,156 BAC clones from the AP85-441 libraries were pooled into 701 libraries. Each library contains an average of 50 BAC clones. The DNA libraries were prepared with the PhasePrep^TM^ BAC DNA Kit (Sigma, United States) following the manufacturer’s protocols. BAC DNA libraries were sequenced using Illumina HiSeq 2500 platform with PE250 model. A total of 686 libraries (18 libraries failed for sequencing) were sequenced, and 267.5 Gb of cleaned data were generated after trimming using Trimmomatic version 0.36 (Bankevich et al., 2012). Data from each BAC pool were assembled using SPAdes version 3.09 with default parameter. A total of 2,611,145 contigs were assembled with contig N50 of 7.38 kbp (Zhang and Ming, unpublished data).

The AP85-441 BAC library sequences were BLAST searched against the sorghum genome with an *E*-value of 1*e*^-4^. The sequences that had single BLAST hits in the sorghum genome were selected as candidate FISH probes. To identify low copy number BAC clones from the BAC library, sequence-specific primers were designed using Primer 5.0 software to enable BAC pools to be screened by PCR specifically. Primer lengths of 18–25 bp, *T*_m_ values of 55–65°C, and nucleotide compositions of 40–60% cytosine and guanine were selected. A two-step PCR method was used to screen the 3D dimension pools of the BAC library (Asakawa et al., 1997; Crooijmans et al., 2000) with some modifications. Each clone from a 384-well plate was cultured in 80 μl Lysogeny broth + 34 mg/ml chloramphenicol overnight at 37°C in a 384-well culture plate. In total, eight 384-well plates with a total of 3072 BAC clones were cultured. For each 384-well plate, we first constructed 16 row pools (row A to P) and 24 column pools (column 1–24) for each 384-well plate by mixing equal volumes of culture of each individual clone, and then mixed the 16 row pools and 24 column pool together with equal volumes from each row and column pool as one plate pool or superpool. In this study, we focused on screening eight 384-well plates. Thus, eight superpools were prepared for the eight plates separately. The positive 384-well plate from the eight plates was identified and subsequently the positive row among the 16 mixtures was screened. Finally, the PCR positive columns were selected among 24 column mixtures. Twenty-four PCR reactions were performed to identify positive clones from the eight 384-well plates.

PCR amplifications were performed as previously described (Bouzidi et al., 2006). Each reaction included rTaq premix 7.5 μl [10× PCR buffer, MgCl_2_ (25 mM), dNTPs (2.5 mM), rTaq polymerase (3 U/1.5 μl)], 0.6 μl of each primer (10 μM), 1.0 μl bacteria liquid template, to a final volume of 20 μl. Following initial denaturation at 95°C for 5 min, 35 cycles of 95°C for 30 s, 55°C for 30 s, and 72°C for 1 min were performed. PCR products were analyzed on a 1.5% agarose gel.

### Metaphase Chromosome Preparation

Chromosome preparation was performed as previously described (Lou et al., 2010) with minor modifications. In brief, *S. spontaneum* plants were grown in the greenhouse conditions for root tips harvesting. Excised root tips about 1–2 cm were treated with 0.002 mol/l 8-hydroxyquinoline at room temperature for 2–4 h, rinsed in water for 15 min, and fixed in ethanol:acetic acid (3:1) for at least 24 h at room temperature. The root tips were then digested in an enzyme solution (4% cellulose R-10 and 2% pectolyase in 0.1 M citrate buffer) at 37°C for 1 h, washed with ice deionized water for 30 min, and finally incubated in ethanol:acetic acid (3:1) for about 30 min. Slides were prepared according to using the “flame-dried” method (Iovene et al., 2008).

### BAC DNA Purification and Probe Labeling

The BAC DNA was extracted with PhasePrep^TM^ BAC DNA Kit according to the manufacturer’s manual. Purified BAC DNA was labeled by standard nick translation reaction, including diluted DNase I, 10× nick translation buffer, DNA Polymerase I, dNTPs, biotin-/digoxigenin-labeled dUTPs, and BAC DNA. The mixture was incubated at 15°C for 1.5 h. The cut products were then examined on a 1.5% agarose gel for the presence of a smear between 300 and 500 bp. The obtained probes were stored at -20°C until used.

### *In Situ* Hybridization and Detection

Probe mix and hybridization: First, the probe mixture (50% deionized formamide, 2× SSC, 80 ng digoxygenin-/biotin-labeled DNA, 10% dextran sulfate, >1 μg C°t-100) was placed in a 90°C hot block for 5 min, then immediately transferred on ice until ready to probe for hybridization. The previously prepared flamed-dried slides were treated with 70% deionized formamide, denatured on a heat block at 80°C for 1.5 min, immediately immersed sequentially in ice cold 70% ethanol, 90% ethanol, and then 100% ethanol each for 5 min, and then air dried on the bench. Finally, denatured probes were added to each slide, and covered with 24 × 32 mm coverslips. Slides were placed in a moist chamber at 37°C overnight.

Probe detection: the coverslips were removed and the slides were washed at room temperature in 2× SSC for 5 min, at 42°C in 2× SSC for 10 min and at room temperature in 1× PBS for 5 min in this sequence. Biotin-labeled probe signals were detected with 2 mg/ml Alexa Fluor 488 streptavidin and digoxygenin-labeled probe signals were detected with 2% anti-digoxigenin-rhodamine from sheep. The antibody cocktail (100 μl TNB buffer, 1 μl Alexa Fluor 488 streptavidin, 1 μl rhodamine anti-dig-sheep) was added to the slides, which were covered with 24 × 32 mm coverslips, incubated for 1 h at 37°C in a moist chamber, and washed at room temperature in 1× PBS three times for 5 min each. Excess liquid was removed and 4′,6-diamidino-2-phenylindole (DAPI) (Sigma, St. Louis, MO, United States) in the antifade solution Vectashield (Vector, Burlingame, CA, United States) was added to counterstain the chromosomes. Images were captured with an Olympus BX63 epifluorescence microscope. FISH signal images were analyzed using the CellSens Dimension software.

## Results

### Screening Low-Copy BAC Clones

**FIGURE 1:**
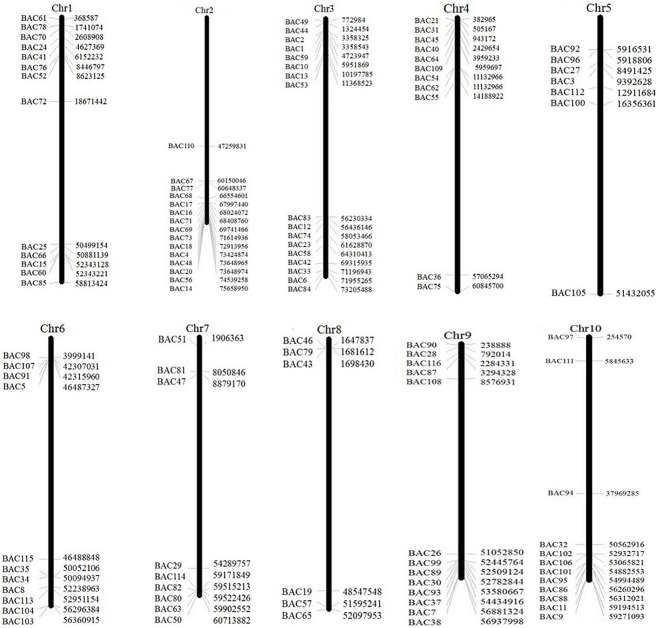
The distributions of 114 BAC clones from AP85-441 in the chromosomes of sorghum. BAC1-114 represents the BAC number, the data on the right are the physical location of BAC in corresponding, region of the sorghum chromosomes. Unit = bp.

To screen the BAC clones that potentially have low copy number sequences in the *S. spontaneum* genome, we BLAST searched the sequenced AP85-441 BAC libraries against the sorghum genome. A total of 2000 BAC sequences corresponding to 10 sorghum chromosomes with good collinearity and showing sequence specificity were screened. The 2000 AP85-441 BAC sequences were masked by REPEATMASKER against the high-repeat sorghum DNA database and TIGR gramineae database to filter repeat sequences. Finally, 114 BACs distributed on 10 sorghum chromosomes were selected for FISH analysis (Supplementary Table 1) with 7–16 BACs on each of the 10 sorghum chromosome (**Figure 1**). To screen positive BAC clones, 114 specific primers were designed based on the BAC clone sequences (Supplementary Table 2) and 49 positive BAC clones were screened from the BAC libraries (Supplementary Table 3).

### BAC-FISH Signal Strength and Distribution

DNA samples isolated from the 49 positive BAC clones were labeled with biotin or digoxigenin. 5S rDNA and 45S rDNA probes were used as control and were hybridized to *S. spontaneum* somatic metaphase chromosomes following the FISH procedure. Due to the correlation between signal strength and the variation of repeated sequence content of the BAC sequences, we used C°t-100 as a competitor (**Table 1**). BAC clones displaying strong and steady hybridization signals were selected for further FISH analysis.

**Table 1:** The classification of 27 positive clones with blocking DNA.

Groups	Blocking DNA	Signal numbers	BAC IDs
I	Not required	7, 8	2, 18, 20, 29, 84
II	25×	4, 6,7	11, 14, 19, 31, 33, 43, 71, 73,74
III	75×	8	26, 66, 76, 78
IV	100×	>8	24
V	150×	1∼6	4, 5, 32, 34, 35, 69, 77, 81

**FIGURE 2:**
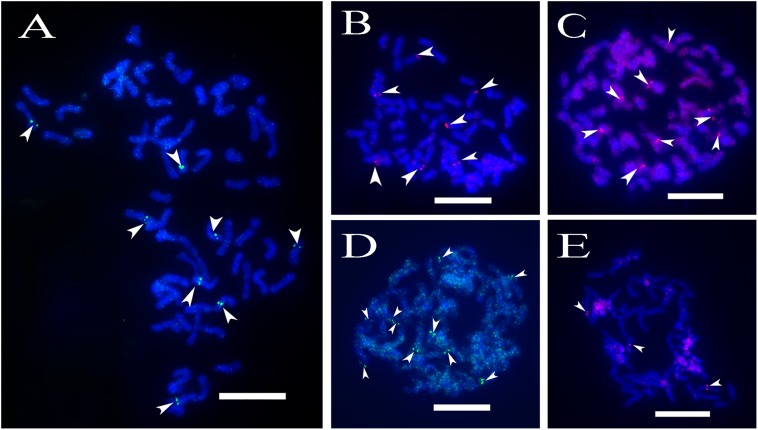
**(A–E)** The spread of the *S. spontaneum* chromosome hybridized with chromosome-specific BAC clones using BAC-FISH. **(A–E)** show hybridization of BAC probes (**Table 2**) to *S. spontaneum* chromosomes. **(A)** BAC-20, **(B)** BAC-43, **(C)** BAC-66, **(D)** BAC-24, and **(E)** BAC-81 probes labeled with biotin (green) and digoxigenin (red). The arrowheads indicate the signals. Scale bars = 10 μm.

**FIGURE 3:**
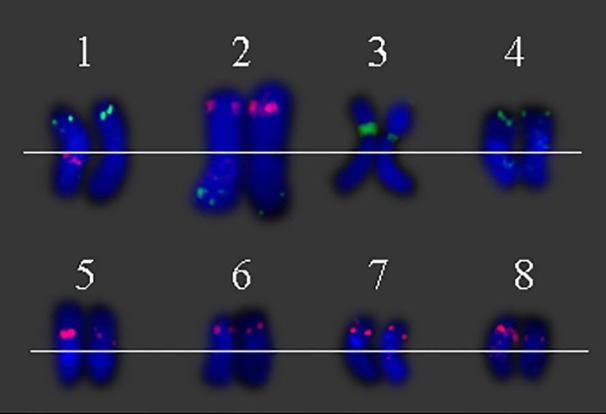
Eight individual *S. spontaneum* chromosomes with chromosome-specific BAC clones. (1) BAC-24 (green) and BAC-66 (red), (2) BAC-14 (green) and BAC-19 (red), (3) BAC-2 (green), (4) BAC-31 (green), (5) BAC-35 (red), (6) 29 (red), (7) BAC-26 (red), and (8) BAC-32 (red) probes labeled with biotin and digoxigenin.

A total of 64 distinct chromosomes can be observed in the metaphase of the *S. spontaneum*. Each BAC-FISH was performed in four independent experiments (or slides). At least 10 spreads of somatic metaphase chromosomes were analyzed in each slide. The results showed that the 27 specific probes (Supplementary Table 4) can be classified into five groups based on the signal of hybridization (**Table 1**). In Group I (including BAC-18, BAC-20, BAC-29, and BAC-84), probes displayed eight distinctive sites without competitive in *situ* suppression (CISS) using C°t-100 (**Figure 2A**), suggesting few repeated sequences existed in these BAC sequences. In Group II (including BAC-2, BAC-11, BAC-14, BAC-19, BAC-31, BAC-33, BAC-43, BAC-71, BAC-73, and BAC-74), C°t-100 was used as competitor for BAC probe hybridization, and six to seven quite distinct sites were displayed in karyotypes of this group (**Figure 2B**). The hybridization result was similar to 45S rDNA with seven signal sites, which may be caused by chromosome structure variation in the homologous chromosome. In Group III (including BAC-26, BAC-66, BAC-76, and BAC-78), C°t-100 DNA was used as a competitor and eight distinct sites could be observed in the karyotype, suggesting this group of BAC clones have repetitive DNA sequences (**Figure 2C**). In Group IV (including BAC-24), more than eight signal sites were observed in the karyotype and appeared in the pericentromeric and telomeric regions (**Figure 2D**), suggesting these BAC probes had repetitive sequences in *S. spontaneum*. In group V (including BAC-4, BAC-5, BAC-32, BAC-34, BAC-35, BAC-69, BAC-77, and BAC-81), the probes showed dispersed signals in all chromosomes with limited regions of individual chromosomes displaying specific signals (**Figure 2E**). The hybridization result indicated that this group of BAC clones contained large amounts of repetitive DNA in *S. spontaneum*, which may be caused by a rapid evolutionary divergence for the repetitive regions after the split of sorghum and *S. spontaneum*. Therefore, of the 49 BAC probes, a set of 19 BAC probes (Supplementary Table 3) from Groups I, II, and III showed specific signals on *S. spontaneum* chromosomes corresponding to nine of the sorghum chromosomes beside Sb05. The signals of the other 22 probes were dispersed in all chromosomes. We selected a set of 19 BAC probes as *S. spontaneum* chromosome-specific signals (**Figure 3**). Of the 19 BAC probes, one probe was located on Sb02, Sb04, and Sb09; two probes on Sb07, Sb08, and Sb10; three probes on Sb01 and Sb06; and four probes on Sb03. This probe set provided a useful tool for comparative analysis of *S. spontaneum* and sorghum (**Table 2**).

**Table 2:** The distribution of BAC-FISH probes in chromosomes of *S. bicolor* and *S. spontaneum.*

*S. bicolor*		*S. spontaneum*	
Chromosome ID	BAC IDs	Chromosome ID	BAC IDs
1	24, 66, 78	1	24^∗^, 66, 78
2	14	2	14^∗^, 19(Sb8)
3	2, 33, 74, 84	3	2^∗^, 33, 74, 84
4	31	4	31^∗^
5	N/A	N/A	N/A
6	5, 34, 35	5	35^∗^, 5, 35
7	29, 81	6	29^∗^, 81
8	19, 43	N/A	N/A
9	26	7	26^∗^
10	11, 32	8	32^∗^, 11

*^∗^ The BAC-FISH results were presented in **Figure 2**.*

### Construction of a BAC Library Specific for *S. spontaneum* Chromosomes

Sb01 and Sb02 are the two largest sorghum chromosomes. To detect the genome synteny between sorghum and *S. spontaneum*, 10 chromosome-specific BAC clones were selected from Groups I, II, and III. Of these 10 BAC probes, three probes (BAC clone ID: 24, 66, 78) were homologous to Sb01 and seven probes (BAC clone ID: 14, 18, 20, 69, 71, 73, 77) corresponded to Sb02. We utilized dual-color detection of FISH for complementarily labeled BAC pairs and the probes for mapping the chain cytogenetic relationship for the BAC clones. By doing so, we constructed the cytogenetic map of *S. spontaneum* based on the 10 BAC probes.

**FIGURE 4:**
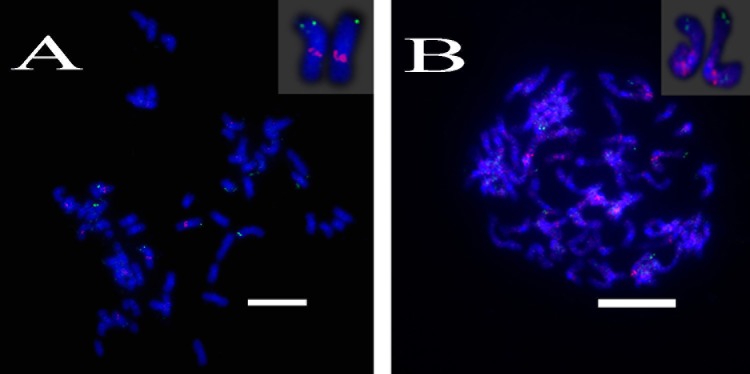
FISH mapping of BAC-24, BAC-66, BAC-78 on SsChr1 in *S. spontaneum*. **(A)** BAC-24 and BAC-66 probes labeled with biotin (green) and digoxigenin (red); **(B)** BAC-78 and BAC-66 probes labeled with biotin (green) and digoxigenin (red). Scale bars = 10 μm.

**FIGURE 5:**
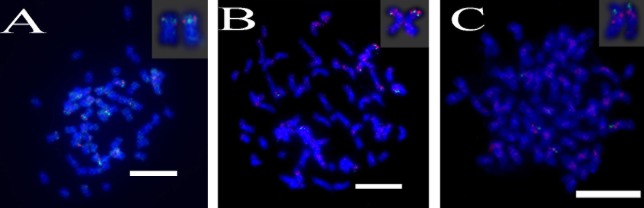
FISH mapping of BAC-69, BAC-71, BAC-73, BAC-77 on SsChr2 in *S. spontaneum*. **(A)** BAC-71 and BAC-69 probes; **(B)** BAC-73 and BAC-69 probes; **(C)** BAC-71 and BAC-77 probes labeled with biotin (green) and digoxigenin (red). Scale bars = 10 μm.

For three probes corresponding to Sb01, FISH analysis of *S. spontaneum* (SES208) showed that BAC-24 and BAC-78 probes were mainly located on the distal region of *S. spontaneum* chromosome 1, and BAC-66 was mapped in close proximity to the centromeric region (**Figure 4**). BAC-24 and BAC-66 located to the same chromosome (*S. spontaneum* chromosome 1); simultaneously whereas probe 66 and probe 78 also located on the same chromosome, demonstrating that the three BAC probes distributed to the same chromosomes in *S. spontaneum* thus suggesting synteny between *S. spontaneum* and sorghum for chromosome 1. FISH results revealed that the seven BAC probes aligned to Sb02; only probes 69, 71, 73, and 77 of the seven BACs generated intense signals on one *S. spontaneum* chromosome (SsChr2) (**Figure 5**); while probes 14, 18, and 20 were undetectable on *S. spontaneum* chromosome 2 but were observed on other *S. spontaneum* chromosomes. These results demonstrated the chromosome rearrangements occurred in the corresponding Sb02 between *S. spontaneum* and sorghum.

**FIGURE 6:**
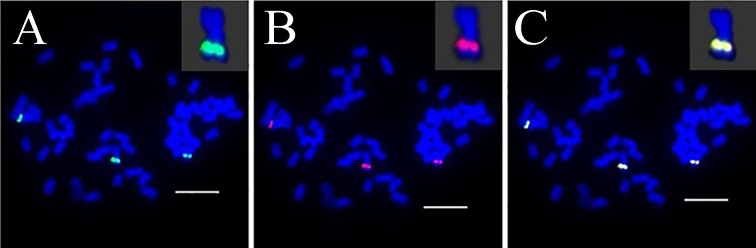
FISH mapping of BAC-2, 45srDNA on SsChr3 in *S. spontaneum*. **(A)** BAC-2 labeled with biotin (green); **(B)** 45s rDNA probes labeled with digoxigenin (red). **(C)** Merged images of **(A)** and **(B)**. Scale bars = 10 μm.

Moreover, the sequence of BAC-2 shared high similarity (97%) to a fragment on chromosome 3 of sorghum. Double color FISH analysis showed that both BAC-2 and 45s rDNA were mapped to the same location of the *S. spontaneum* chromosome (**Figure 6**).

### Chromosome Rearrangement on Sb02 between Sorghum and *S. spontaneum*

**FIGURE 7:**
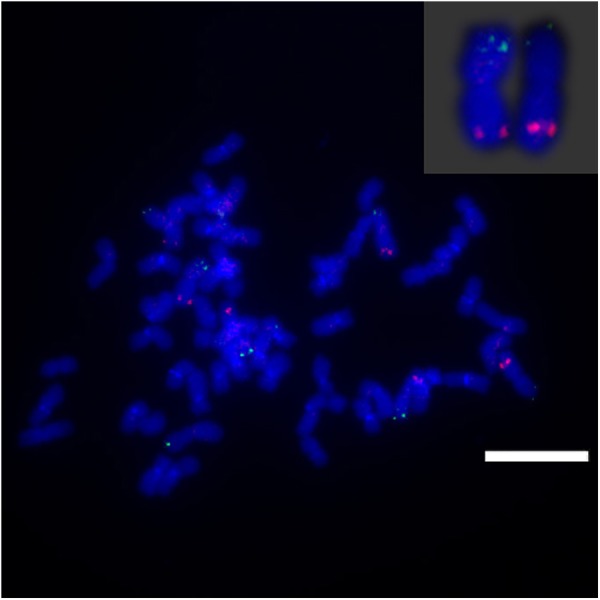
The rearrangement of chromosome in sugarcane and sorghum as revealed by FISH. BAC-14 and BAC-19 probes labeled with biotin (green) and digoxigenin (red). Scale bars = 10 μm.

To further investigate the chromosome rearrangement on *Sb02* between sorghum and *S. spontaneum*, chromosome-specific probes corresponding to Sb02 (BAC-14, BAC-18, and BAC-20) and corresponding to Sb08 (BAC-19 and BAC-43) were used for FISH analysis. The results showed that BAC-14 and BAC-19 were mapped to different arms of the same *S. spontaneum* chromosomes, indicating that chromosome rearrangements took place between chromosomes 2 and 8 in *S. spontaneum* and sorghum (**Figure 7**).

## Discussion

*Saccharum* is a complex genus characterized by high polyploidy levels, with small chromosomes (Ha et al., 1999; Dhont, 2005). Distinguishing individual *Saccharum* chromosomes based on their morphology is very challenging. Taking advantage of sequences that are highly covered by the BAC library of *S. spontaneum*, we were able to screen for the potential low copy number BAC clones in *S. spontaneum* using the sorghum genome as a reference. As a high polyploid species, *S. spontaneum* may contain more genome rearrangements than diploids. The differences of the genomes between *S. spontaneum* and sorghum may be the cause of inexactitude in the predictions of the low copy number BAC sequences in *S. spontaneum* genome. In this study, we have initially obtained 114 potential BAC sequences with single-copy based on the sorghum genome. However, only 49 could be identified in the partial BAC libraries, and 27 were found to be chromosome-specific BAC-FISH probes. There are at least two reasons to explain these results. Firstly, the 114 BAC sequences were partial fragments of the BAC insertion due to the limitations of NGS sequence assembly, and may contain repeat sequences in the other regions that have no sequence information. These potentially uncovered repeat sequences could mislead the detection of low-copy sequence through sequence comparison. Secondly, the recent polyploidization in *S. spontaneum* may cause the variation of repeat sequences between *S. spontaneum* and sorghum, and thus result in the nonspecific FISH signal in *S. spontaneum*. In a previous study, sequencing 20 BACs in sugarcane hybrids generated 1.45 Mb contig sequences, the sequences aligning with sorghum genome spanned 0.99 Mb, which accounted for about half of the sugarcane BAC sequences (Wang et al., 2010). Obviously, deletions/insertions existed between sorghum and *Saccharum*, and the utilization of the sorghum genome as reference cannot replace the unique features of the *Saccharum* genome, and thus may cause unpredicted FISH results.

Some FISH slides had strong background noise, which may be due to small regions of repeat sequences (such as SSR sequences) in the BAC clone, which consequently produce interference during hybridization. The sorghum genome has a repeat content of approximately 61% (Paterson et al., 2009), whereas about half of the genome is composed of repeat sequences in *Saccarrum* hybrids (De et al., 2014). In these repeat-rich genomes, it is difficult to develop signal-specific FISH probes, which distinguish chromosomes cytogenetically with similar morphologies. CISS with blocking DNA C°t-100 can efficiently preclude repeat sequences. In this study, blocking DNA C°t-100 was used for FISH analysis of the BAC probes in Groups II and III (**Figures 2D–F**), which were verified to be the chromosome-specific BAC probes, while the FISH of BAC probes in Groups IV and V produced strong background interference (**Figures 2D–F**). It is obvious that there are variations of repeat sequences among the examined BAC probes. Therefore, optimization of experiments would be necessary for BAC-FISH analysis in *Saccharum*. Six to seven sites were displayed in Group II, which could be caused by the homologous chromosome structure variations, such as the fragment deletion in one or two homologous chromosomes. In hexaploid wheat, BAC 676D4 hybridized more strongly to the A-genome chromosomes than to the B- and D-genome chromosomes (Zhang et al., 2004). These undetectable of one to two signals in the homologous chromosomes also could be caused by the technical issues of BAC-FISH for such many chromosomes with small size.

In this study, we identified 27 chromosome-specific BAC-FISH probes that correspond to 9 of the 10 sorghum chromosomes (all except Sb05). In a study of the genetic map derived from a cross between *S. officinarum* and sugarcane cultivar, Sb05 was merged with Sb06 in sugarcane (Aitken et al., 2014). In our study, a genetic map based on the F1 population of *S. spontaneum* revealed that Sb05 was divided into two segments, and the two segments were merged with Sb06 and Sb07, respectively (Zhang and Ming, unpublished data). Recently, the genetic map of a member of the *Andropogoneae*, *Miscanthus sinensis*, demonstrated that Sb05 has poor collinearity with the corresponding linkage group in *M. sinensis* (Ma et al., 2012). Similarly, the two ancestral maize chromosomes orthologous to Sb05 retain the smallest number of syntenic orthologs to sorghum genes (Schubert and Lysak, 2011). Therefore, the absence of the BAC-FISH corresponding to Sb05 may indicate chromosome fusion in *S. spontaneum*.

Sorghum and *S. spontaneum* diverged 12 million years ago (MYA), the basic chromosome number was reduced from *x* = 10 to *x* = 8. In Aitken et al. (2014), the HG2 (homologous group 2) of sugarcane aligned to Sb05 and Sb06, and HG8 (homologous group 8) to Sb02 and Sb08, providing evidence for the basic chromosome reduction event (Aitken et al., 2014). In this study, we observed that the sorghum chromosomes Sb02 and Sb08 had interchromosomal rearrangement in *S. spontaneum* as demonstrated by the evidence that BAC-14 aligned to Sb02, and BAC-19 aligned to Sb08 (**Figure 7**). Our study provided the first physical and cytogenetic evidence for the sorghum inter-chromosome rearrangement in *S. spontaneum*. Unpublished data from our group also revealed that Sb08 is divided into two segments, and were merged with of Sb02 and Sb09 in *S. spontaneum*, respectively (Zhang and Ming, unpublished data). The probes corresponding to Sb09, Sb08, Sb05, and Sb06 could be further used for investigating the inter-chromosomal events between sorghum and *S. spontaneum*. *S. spontaneum* has a wide range of ploidy levels (2*n* = 40–128) (Irvine, 1999). These BAC probes could be used to confirm the inter-chromosomal rearrangement of *S. spontaneum* with the different polyploidy levels.

Due to different basic chromosome number between sorghum (*x* = 10) and *S. spontaneum* (*x* = 8), the sorghum chromosomes were not a one-to-one correspondence with *S. spontaneum*. As our genetic mapping study mentioned herein before, Sb08 was divided into two segments which merged with segments of Sb02 and segments of Sb09 in *S. spontaneum*; Sb05 was divided into two segments which merged with segments of Sb06 and segments of Sb07 *S. spontaneum*. Therefore, the probes corresponding to Sb08 and Sb05 were not specific to single *S. spontaneum* chromosomes, whereas the other probes corresponding to the other eight sorghum chromosomes can be used as chromosome-specific cytogenetic BAC-FISH probes for *S. spontaneum*. Thus, the BAC probes tested were based on the eight sorghum chromosomes (**Table 2**) were sufficient for chromosome identification using BAC-FISH. A satellite chromosome was found in one *S. spontaneum* chromosome (chromosome 3) (Ha et al., 1999). Previously, simultaneous FISH revealed that the signals of 45S rDNA were located on the secondary constrictions of the satellite chromosomes within the chromosome 3 from the anther culture-derived *S. spontaneum* clone (AP85-361) (Ha et al., 1999). In this study, BAC-2 which corresponds to Sb03 and 45s rDNA were mapped to the same location of the *S. spontaneum* chromosome, thus further supporting previous findings (Ha et al., 1999). Our results also provided direct evidence that chromosome 3 of *S. spontaneum* named in the previous study is homologous to Sb03.

## Conclusion

In this study, we developed chromosome-specific BACs of *S. spontaneum* as a step toward the development of a simple and reproducible method for chromosome identification using BAC-FISH cytogenetic markers, confirming the feasibility of isolating chromosome-specific BACs based on the sorghum genome to construct a physical map of sugarcane. We also provide the first cytogenetic evidence of inter-chromosomal rearrangement between sorghum and *S. spontaneum.* The establishment of the sugarcane BAC-FISH technology system offers new opportunities and the means for sugarcane molecular cytogenetics research, including karyotype analysis, gene localization, and physical map construction. These results are essential for assembly of *S. spontaneum* genome required for whole-genome sequencing.

## Author Contributions

GD, JS, and JZ conceived the study and designed the experiments. GD, JS, QZ, JW, QY, RM, KW, and JZ carried out the experiments and analyzed the data. GD and JZ wrote the manuscript. All authors read and approved the final paper.

## Conflict of Interest Statement

The authors declare that the research was conducted in the absence of any commercial or financial relationships that could be construed as a potential conflict of interest.
