# Supplementary material for: Development and Applications of Chromosome-Specific Cytogenetic BAC-FISH Probes in S. spontaneum
Source: Front Plant Sci. 2018 Feb 26;9:218. doi: 10.3389/fpls.2018.00218 (PMC5834487; doi:10.3389/fpls.2018.00218)
Supplement: Supplementary file 1 [file Table_1.doc]

**Supplemental table 1. The distribution of AP85-441 BACs in sorghum.** Notes: A:contig number; B: the most frequent aligned chromosome; C: identity/%; D: alignment length/bp; E: the starting position in chromosome corresponding to alignment; F: the end position; G: e-value; H: bit score.

| A | B | C | D | E | F | G | H |
| --- | --- | --- | --- | --- | --- | --- | --- |
| 1 | SbChr3 | 93.06 | 4065 | 3358543 | 3362578 | 0 | 5845 |
| 2 | SbChr3 | 91.11 | 3262 | 3358325 | 3361541 | 0 | 4329 |
| 3 | SbChr5 | 88.23 | 1640 | 9392628 | 9391008 | 0 | 1908 |
| 4 | SbChr2 | 89.56 | 6321 | 73424874 | 73431073 | 0 | 7805 |
| 5 | SbChr6 | 86.13 | 1550 | 46487327 | 46488848 | 0 | 1563 |
| 6 | SbChr3 | 94.27 | 2878 | 71955265 | 71952430 | 0 | 4340 |
| 7 | SbChr9 | 94.82 | 1757 | 56881324 | 56879591 | 0 | 2710 |
| 8 | SbChr6 | 90.03 | 3942 | 52238963 | 52242811 | 0 | 4924 |
| 9 | SbChr10 | 95.58 | 1245 | 59271093 | 59269852 | 0 | 1986 |
| 10 | SbChr3 | 91.21 | 910 | 5951869 | 5952772 | 0 | 1230 |
| 11 | SbChr10 | 90.94 | 2240 | 59194513 | 59192336 | 0 | 2928 |
| 12 | SbChr3 | 93.02 | 1905 | 56436146 | 56438023 | 0 | 2739 |
| 13 | SbChr3 | 93.82 | 1684 | 10197785 | 10196127 | 0 | 2484 |
| 14 | SbChr2 | 88.84 | 2742 | 75658950 | 75661616 | 0 | 3247 |
| 15 | SbChr1 | 93.49 | 1766 | 52343128 | 52341374 | 0 | 2584 |
| 16 | SbChr2 | 96.85 | 3997 | 68024072 | 68028052 | 0 | 6663 |
| 17 | SbChr2 | 94.57 | 4146 | 67997440 | 68001559 | 0 | 6362 |
| 18 | SbChr2 | 89.32 | 3521 | 72913956 | 72917472 | 0 | 4401 |
| 19 | SbChr8 | 92.25 | 6332 | 48547548 | 48553778 | 0 | 8802 |
| 20 | SbChr2 | 93.15 | 6802 | 73648974 | 73642250 | 0 | 9867 |
| 21 | SbChr4 | 93.91 | 5307 | 382965 | 388244 | 0 | 7954 |
| 23 | SbChr3 | 94.04 | 8361 | 61628870 | 61637139 | 0 | 12504 |
| 24 | SbChr1 | 92.73 | 5987 | 4627369 | 4621448 | 0 | 8514 |
| 25 | SbChr1 | 93 | 5043 | 50499154 | 50494242 | 0 | 7206 |
| 26 | SbChr9 | 88.69 | 1706 | 51052850 | 51051196 | 0 | 1993 |
| 27 | SbChr5 | 90.02 | 2746 | 8491425 | 8494153 | 0 | 3500 |
| 28 | SbChr9 | 91.38 | 3354 | 792014 | 795287 | 0 | 4449 |
| 29 | SbChr7 | 94.24 | 6113 | 54289757 | 54283671 | 0 | 9291 |
| 30 | SbChr9 | 94.14 | 4249 | 52782844 | 52787048 | 0 | 6362 |
| 31 | SbChr4 | 89.79 | 3791 | 505167 | 508908 | 0 | 4756 |
| 32 | SbChr10 | 94.98 | 1812 | 50562916 | 50561124 | 0 | 2811 |
| 33 | SbChr3 | 89.77 | 1378 | 71196943 | 71195587 | 0 | 1725 |
| 34 | SbChr6 | 90.85 | 1639 | 50094937 | 50096552 | 0 | 2150 |
| 35 | SbChr6 | 95.78 | 1660 | 50052106 | 50053746 | 0 | 2660 |
| 36 | SbChr4 | 95.15 | 5813 | 57065294 | 57071063 | 0 | 9073 |
| 37 | SbChr9 | 92.03 | 3238 | 54434916 | 54438071 | 0 | 4429 |
| 38 | SbChr9 | 93.45 | 2624 | 56937998 | 56935384 | 0 | 3838 |
| 40 | SbChr4 | 89.29 | 3090 | 2429654 | 2426621 | 0 | 3808 |
| 41 | SbChr1 | 90.77 | 2622 | 6152232 | 6154787 | 0 | 3391 |
| 42 | SbChr3 | 92.07 | 2838 | 69315935 | 69313122 | 0 | 3912 |
| 43 | SbChr8 | 85.87 | 729 | 1698430 | 1699147 | 0 | 763 |
| 44 | SbChr3 | 88.61 | 2432 | 1324454 | 1326807 | 0 | 2861 |
| 45 | SbChr4 | 89.57 | 5880 | 943172 | 937391 | 0 | 7203 |
| 46 | SbChr8 | 87.33 | 1902 | 1647837 | 1649712 | 0 | 2132 |
| 47 | SbChr7 | 86.83 | 3934 | 8879170 | 8882978 | 0 | 4204 |
| 48 | SbChr2 | 92.92 | 6791 | 73648965 | 73642250 | 0 | 9749 |
| 49 | SbChr3 | 90.76 | 4264 | 772984 | 777154 | 0 | 5535 |
| 50 | SbChr7 | 85.19 | 2444 | 60713882 | 60711487 | 0 | 2429 |
| 51 | SbChr7 | 89.22 | 3998 | 1906363 | 1902487 | 0 | 4802 |
| 52 | SbChr1 | 93.15 | 3998 | 8623125 | 8627088 | 0 | 5779 |
| 53 | SbChr3 | 96.79 | 3423 | 11368523 | 11371940 | 0 | 5692 |
| 54 | SbChr4 | 89.54 | 3337 | 11132966 | 11129701 | 0 | 4124 |
| 55 | SbChr4 | 93.78 | 1992 | 14188922 | 14186974 | 0 | 2931 |
| 56 | SbChr2 | 92.68 | 1516 | 74539258 | 74540756 | 0 | 2161 |
| 57 | SbChr8 | 93.94 | 1781 | 51595241 | 51597015 | 0 | 2667 |
| 58 | SbChr3 | 95.75 | 3643 | 64310413 | 64314031 | 0 | 5816 |
| 59 | SbChr3 | 89.43 | 3729 | 4723947 | 4727595 | 0 | 4532 |
| 60 | SbChr1 | 92.35 | 2314 | 52343221 | 52345496 | 0 | 3238 |
| 61 | SbChr1 | 93.08 | 2472 | 368587 | 366139 | 0 | 3572 |
| 62 | SbChr4 | 89.54 | 3337 | 11132966 | 11129701 | 0 | 4124 |
| 63 | SbChr7 | 93.15 | 2935 | 59902552 | 59899681 | 0 | 4211 |
| 64 | SbChr4 | 94.83 | 1374 | 3959233 | 3960596 | 0 | 2130 |
| 65 | SbChr8 | 95.16 | 6204 | 52097953 | 52091785 | 0 | 9714 |
| 66 | SbChr1 | 93.28 | 6101 | 50881139 | 50887175 | 0 | 8874 |
| 67 | SbChr2 | 90.99 | 2507 | 60150046 | 60152497 | 0 | 3302 |
| 68 | SbChr2 | 90.13 | 2806 | 66554601 | 66551847 | 0 | 3550 |
| 69 | SbChr2 | 91.72 | 3671 | 69741466 | 69745103 | 0 | 4968 |
| 70 | SbChr1 | 88.64 | 3214 | 2608908 | 2605737 | 0 | 3698 |
| 71 | SbChr2 | 96.4 | 5612 | 68408760 | 68403183 | 0 | 9190 |
| 72 | SbChr1 | 94.97 | 2067 | 18671442 | 18673495 | 0 | 3214 |
| 73 | SbChr2 | 92.6 | 2745 | 71614936 | 71617657 | 0 | 3866 |
| 74 | SbChr3 | 92.31 | 3890 | 58053466 | 58049649 | 0 | 5437 |
| 75 | SbChr4 | 92.82 | 6061 | 60845700 | 60839741 | 0 | 8604 |
| 76 | SbChr1 | 94.27 | 4730 | 8446797 | 8451492 | 0 | 7171 |
| 77 | SbChr2 | 88.74 | 3747 | 60648337 | 60651936 | 0 | 4379 |
| 78 | SbChr1 | 91.12 | 3087 | 1741074 | 1744100 | 0 | 4048 |
| 79 | SbChr8 | 89.31 | 1871 | 1681612 | 1679769 | 0 | 2287 |
| 80 | SbChr7 | 95.35 | 4541 | 59522426 | 59526924 | 0 | 7138 |
| 81 | SbChr7 | 87.7 | 1870 | 8050846 | 8052649 | 0 | 2098 |
| 82 | SbChr7 | 92.22 | 2520 | 59515213 | 59512723 | 0 | 3502 |
| 83 | SbChr3 | 92.09 | 4414 | 56230334 | 56234700 | 0 | 6163 |
| 84 | SbChr3 | 95.75 | 5930 | 73205488 | 73211397 | 0 | 9492 |
| 85 | SbChr1 | 93.91 | 4316 | 58813424 | 58809161 | 0 | 6412 |
| 86 | SbChr10 | 93.95 | 5822 | 56260296 | 56254531 | 0 | 8667 |
| 87 | SbChr9 | 80.53 | 2137 | 3294328 | 3296423 | 0 | 1557 |
| 88 | SbChr10 | 93.43 | 1888 | 56312021 | 56310153 | 0 | 2750 |
| 89 | SbChr9 | 94.06 | 3284 | 52509124 | 52505885 | 0 | 4920 |
| 90 | SbChr9 | 93.09 | 5296 | 238888 | 244116 | 0 | 7614 |
| 91 | SbChr6 | 92.37 | 2921 | 42315960 | 42313068 | 0 | 4082 |
| 92 | SbChr5 | 80.45 | 2291 | 5916531 | 5918806 | 0 | 1701 |
| 93 | SbChr9 | 91.13 | 2604 | 53580667 | 53578109 | 0 | 3443 |
| 94 | SbChr10 | 95.72 | 3664 | 37969285 | 37965650 | 0 | 5860 |
| 95 | SbChr10 | 90.51 | 1865 | 54994489 | 54996281 | 0 | 2383 |
| 96 | SbChr5 | 80.45 | 2291 | 5918806 | 5916531 | 0 | 1701 |
| 97 | SbChr10 | 92.67 | 2796 | 254570 | 257348 | 0 | 3956 |
| 98 | SbChr6 | 89.54 | 2284 | 3999141 | 4001388 | 0 | 2828 |
| 99 | SbChr9 | 95.14 | 3147 | 52445764 | 52442637 | 0 | 4939 |
| 100 | SbChr5 | 87.35 | 577 | 16356361 | 16355789 | 0 | 649 |
| 101 | SbChr10 | 92.71 | 2085 | 54882553 | 54884613 | 0 | 2976 |
| 102 | SbChr10 | 93.73 | 2106 | 52932717 | 52934776 | 0 | 3114 |
| 103 | SbChr6 | 91.34 | 4146 | 56360915 | 56356851 | 0 | 5509 |
| 104 | SbChr6 | 93.63 | 2261 | 56296384 | 56298628 | 0 | 3345 |
| 105 | SbChr5 | 91.2 | 1387 | 51432055 | 51430697 | 0 | 1851 |
| 106 | SbChr10 | 90.04 | 3194 | 53065821 | 53062701 | 0 | 4039 |
| 107 | SbChr6 | 94.08 | 3529 | 42307031 | 42303525 | 0 | 5284 |
| 108 | SbChr9 | 84.83 | 2294 | 8576931 | 8579174 | 0 | 2198 |
| 109 | SbChr4 | 92.19 | 3853 | 5959697 | 5963514 | 0 | 5349 |
| 110 | SbChr2 | 92.84 | 5907 | 47259831 | 47265666 | 0 | 8419 |
| 111 | SbChr10 | 92.88 | 4831 | 5845633 | 5850386 | 0 | 6870 |
| 112 | SbChr5 | 93.73 | 2837 | 12911684 | 12914489 | 0 | 4178 |
| 113 | SbChr6 | 87.77 | 2290 | 52951154 | 52948952 | 0 | 2551 |
| 114 | SbChr7 | 93.23 | 4816 | 59171849 | 59167121 | 0 | 6977 |
