# Supplementary material for: Development and Applications of Chromosome-Specific Cytogenetic BAC-FISH Probes in S. spontaneum
Source: Front Plant Sci. 2018 Feb 26;9:218. doi: 10.3389/fpls.2018.00218 (PMC5834487; doi:10.3389/fpls.2018.00218)
Supplement: Supplementary file 2 [file Table_2.docx]

**Supplemental table 2. The list of primer sequences for BAC library screening.** Note: only list the primer sequences for the positive BAC in PP (Plate-pool) mixed pool.

| BAC IDs | forward primer (5’-3’) | reverse primer (5’-3’) | products (bp) |
| --- | --- | --- | --- |
| 1 | ATGAGCCCTTGGGAACCT | GCACGACCCTGAGCATAGA | 691 |
| 2 | CTGTATGAGGTCCAAGTGAGG | CAAAGCAGAAGCAGGAGTGTA | 442 |
| 3 | CCCTGGCAGAGCAGACTTA | CTTCTTGCGGCACTCTTCA | 384 |
| 4 | ATGTAGATTGCGCCAGTTAA | TCATAGCCAGGAGACAGAAA | 622 |
| 5 | CGTGAACGCATCAAGGTCG | TCGCCCTCCATGATGACCC | 557 |
| 6 | TGCTCCACAAACTACTCCA | CTTGATGCCTTCACCACAG | 664 |
| 7 | TTTTGTTGCGGTTTGTTCC | TTGCTCGTTCTGCGGTCAT | 563 |
| 8 | GTGCCAACTGTATCACCAGC | AACAATGAAGCAAGGTAGCG | 576 |
| 9 | GCCAGTAAGGGTCTTCACG | TGTTCCTCCTCCACGGTCT | 772 |
| 10 | CGCTACTTAGTTCCAGAGGGG | CGCTTCGCATCTACTTGTCGT | 619 |
| 11 | TCCCAATGCACAAGGTCA | CTCCCGCTCATCGTCTAA | 616 |
| 12 | CCATTATTCACAATCCCGTAG | TACACCTCATCACCTCCACAT | 817 |
| 13 | AGGGATCATTGCCAGTAGG | TCCAGGGTAATAGCAGTCG | 762 |
| 14 | TCGTGCTCATTGTTGACTTA | ACTTCGTTTCATCTGCGTTT | 628 |
| 15 | CGTGGTCTTCTTCTTGATGC | CGGGTAATAGTAGTCGTGCC | 820 |
| 16 | GTCAAGAACGGCAAACACG | GGAGAACAGATGCTCGCTAA | 747 |
| 17 | AACGCATTGTTACCAGCAT | TCACATGGCAGAGCCTACT | 720 |
| 18 | TTGAGCGTGTAGGGTTTATGC | TGCTTTCCTTGGAGGTATTTG | 412 |
| 19 | GCCCAACTATGTTCTGTCACCA | GACCATCTTCAACCCACTGTATCT | 735 |
| 20 | TTGAGGGTTTCCATCTACTG | CTGATTGAGGACTTGGCATA | 598 |
| 21 | TGCCAGTAGTGTAGTGGTTTCG | ATTTGCGAGCGTCGATTT | 510 |
| 22 | GCAAGGAGGTCTGGGTATG | CCAAACTGCTCGGAGTATGTC | 617 |
| 23 | TAGGCACCAAGGAGATACGC | CCCAACTCTAAACCGTCGTAA | 533 |
| 24 | TCATTGACCGTGCGGAAAC | GGACTCATCAGCCCACCTC | 780 |
| 25 | CGTTTGCTGGCTATGGTCAC | ATCGGATAATGTCAGGGAGG | 545 |
| 26 | AAGGCTCTGCTGCGACAA | AAGGAAACAAGCCCAACG | 757 |
| 27 | CGACAAGACCCAAAGGAGAAG | TCCAGGTTAGCGTTAGCCACA | 579 |
| 28 | GAAATCATCCGTCACATAAGC | GAACCCTCACTCAAGAAATACA | 420 |
| 29 | AAGGATACTCGGGATGAAGAC | CGTTGAGGCAATGAACCACT | 408 |
| 30 | GCCTCGTCTCATCTTCCC | TCCTTGTCCACCCATTCC | 675 |
| 31 | GCTCCATCTCATACTTTCATACCG | CCGTAAATAAACCTTCCGCTTC | 900 |
| 32 | AGGGTCTTGGCAGGTTGTA | ATCAGGTCGGTTCGTTTCC | 588 |
| 33 | GGCGTCCCATCCCTTCT | CGGCTCCTCATCATCTCG | 414 |
| 34 | ACATCAACTCTGGCTTTCCC | ACGGCGAGCACCTTCTG | 417 |
| 35 | ACTTCACTAACCCAACCATACCG | GGCTACCTTACACCCTCGTTTT | 622 |
| 36 | GGTGGTGGAATGGGATAG | TTGCCAGTTCTTATGATGCT | 540 |
| 37 | ACTGAGGCCCGATTTGGAG | TGGAGAACCTTTGCCGTGA | 450 |
| 38 | TCTGGGTCAGGAATAGCACG | GCGTTTGTTTGCCGTTAGAT | 777 |
| 39 | AATGTGCCGCCTTCAACCT | TCCTAATCGCCCAACTCCA | 473 |
| 40 | AAGAAGCACCCACAAATCACG | AACAGCCTGGCAATACGAAG | 559 |
| 41 | GCGCCATAATGACGTTTC | CAAGCTGCCAGGCTACAA | 461 |
| 42 | AGAGCAACATACGCCACAT | GCTGACCACCTGAAGCAATA | 485 |
| 43 | TACCTCGCCTGAATCTTTAC | TTGATGAATGAGTTCCGTGA | 495 |
| 44 | TACCGCAACAAACCCAGAG | AATGCCAAGAAGCAACGTG | 482 |
| 45 | AAGAGGTCGCAAGCGTTTT | CCTGCCTGAGGAGCATAATA | 463 |
| 46 | GGCTTGTTTGTATTAGGTGCT | GTGATGGTGATAAAGGTCGC | 613 |
| 47 | GACGACGACCCCGAGTAG | CGGAACATCCAGGAAACC | 874 |
| 48 | TCAGCATCTCCGTCCACTC | GCATACCGAGCACAAAACA | 689 |
| 49 | CGTCAAGACGTTCGTGGTG | GGACTTTGTTGGGGGCA | 495 |
| 50 | TGTGTTGGCGACATCTGGA | GCGTTGGGTTGCTCTCTTT | 509 |
| 51 | CACACGGTGACACGGAGAC | CCCACAACAAAAGCAGGAA | 305 |
| 52 | GCAAGCAATCAAAAGCCAA | CACATCCTAAAGTCCAAGCG | 309 |
| 53 | GGAGTTGGGTGGATGTGTT | GGGATTCTTTGCTTGCTGT | 579 |
| 54 | GGTTCCATCGTTTATCCGT | AAAAGGAAAAGTCACGGGT | 565 |
| 55 | CCAGGCTGTGAGTATGTGAAT | TTTGTTGGAAGGAGGGAGT | 608 |
| 56 | GTATTGTGGATTTAGGGCTGC | TGAGTTTGTGAGTCTCGGTTTG | 388 |
| 57 | CACAATCTAACATCAGCCACG | AGGGAACTAACGAACGAACAC | 429 |
| 58 | GGAGGAATGCGGCAGAAGA | GAATGGGGCAAGGGGCT | 214 |
| 59 | GGCAGGAGTTCGGGGGTTT | TTGCTTTGTGGCTGGGAGAGT | 255 |
| 60 | AAGCGAATGGTCGTCTAACT | ACAGAATGACCCTAATGCCA | 717 |
| 61 | CATCATCTGTTGTTGTGGCA | GGTTGAGCACAGACTCCTTG | 168 |
| 62 | TGTTTTCTTTCACCCGTTCTG | CGGTCCTCCATGACTTCTCC | 520 |
| 63 | CAGGCAAAAAATGACGAAGT | ACACTGAAACCAAAACGAGC | 426 |
| 64 | GCTGAAATAAACTGCCACGAG | GCCAAAATGAAGAAACCTGC | 545 |
| 65 | TTAGCAGTGCGGTTGGAG | AGTGGAAGGCAGGATGAGA | 303 |
| 66  67 | TGGAGCCTTTCGTTTTCAG  TCTCTTCCTCTACCGTTCCT | CCCAGACGGATGTGTGAAC  CGGTCAAGCCAATACTAAAG | 474  490 |
| 68 | CAGCGAGGTTGAAAGTTGAGAC | CCCAGCCCAAAGATAGAGAA | 489 |
| 69 | CAGCAAGAGGAGCAGACAGAG | GCGAAATGGAGATGAACGAG | 250 |
| 70 | GCGAGGAACAAACAAACCA | CAAAAGAAGCGGAAGCGAT | 203 |
| 71 | CCAAGGAGAGGAAAAAACAGC | GCAAAGAAGCCCAGAAGGTC | 222 |
| 72 | TCCCTAAAACGCCTCAACG | TCTCTTCCGCATCCTCCTC | 385 |
| 73 | CCGTGAACCTCATCCGACT | GCCACCTCCTTCTCCAACT | 213 |
| 74 | AACTCAGCCAAGCCGATAG | TACTGTTGGTGCCTGTGTTG | 320 |
| 75 | TCTCCGTTCCTCCGTATTT | AGGTGTTCCCTCCCTCTTG | 411 |
| 76 | AGGGGAAGGTCACCAAAAT | CCACAGAACAGCGTCAACA | 367 |
| 77 | AAGATGCCTCCGTCTCCAC | TCGCCACAAGCCACAATAG | 575 |
| 78 | GCTTGGCAACAACCCTCTC | AACCTCGCTCCTCACCTTC | 427 |
| 79 | CAGCAGGCAGCCCAACAT | TAGCAAGGCGAAGTAAGCG | 679 |
| 80 | CGGGGACGCTAAGTTTTG | TCCTGATGCCTGATGGTTC | 839 |
| 81 | CTTCCTCGTGTTGGTCCT | ACAGCCAACCATCGCAC | 309 |
| 82 | ACATTGGAGGAGCCGTTGA | GCCGTCTCTCGCTTTCAA | 596 |
| 83 | TTGTGGAGGATGGTAACGA | GATGTTTACCTCGACGCAG | 443 |
| 84 | CCTTGGCAACAACCCTGTC | TACCTCGCTCCTCACGTAC | 457 |
