# Supplementary material for: Development and Applications of Chromosome-Specific Cytogenetic BAC-FISH Probes in S. spontaneum
Source: Front Plant Sci. 2018 Feb 26;9:218. doi: 10.3389/fpls.2018.00218 (PMC5834487; doi:10.3389/fpls.2018.00218)
Supplement: Supplementary file 3 [file Table_3.docx]

| *Sorghum bicolor (L.)* chromosomes | ID of positive BAC clones |
| --- | --- |
| 1 | 15, 24, 66, 70, 76, 78 |
| 2 | 4, 14, 16, 17, 18, 20, 69, 71, 73, 77 |
| 3 | 2, 6, 10, 13, 23, 33, 42, 44, 53, 58, 74, 84 |
| 4 | 31, 36, 40 |
| 5 | 27 |
| 6 | 5, 8, 34, 35 |
| 7 | 29, 51, 80, 81 |
| 8 | 19, 43 |
| 9 | 7, 26, 28, 38 |
| 10 | 9, 11, 32 |

**Supplemental table 3. The distribution of 49 positive clones in sorghum chromosomes.**
