# Supplementary material for: Development and Applications of Chromosome-Specific Cytogenetic BAC-FISH Probes in S. spontaneum
Source: Front Plant Sci. 2018 Feb 26;9:218. doi: 10.3389/fpls.2018.00218 (PMC5834487; doi:10.3389/fpls.2018.00218)
Supplement: Supplementary file 4 [file Table_4.docx]

**Supplemental table 4. BAC clones sequences of the 27 probes.**

>BAC-78

TTGGTATGATTTAAATTAATACGACTCACTATAGGGTCAGTGCGGCCGCGACTTCAAGTCACGTGAATTCCAAGCTTCAGAAAGCGATGGCCCAGTGGCGGTCGTGCACGTAGACGAAGGCGTGGTAGGAGCAGGCCTGGCACCTGAGCTTGCACATCCCTAGGGACACCAGCGACGAGAAGGGCGTTTTGCACCGGGAGCATTCGGTGGGGCACGTGAACTTCTCGCCGTCGTCACGTTGCCGCCGCCACGGCGGAGGAGGATCACGTGGCGAGGGCGCTCTGCAGGGCAGGGAGCGACGCCAAGTGCCTCTTGGCGTTCCGGGCCACCCGCAGCGCGCCGTCGAGATCGTCGACGAGGAAGCGCTCCTCCGCCGATCTGAAGGCTTTCTCCGCCTCCTCCGTGCGGACAGTTCGACTCCAACTTGCGTGATCGATCGCAACCTACACGTCTGTTTACCGCCTCGGACTGGTGCTCTCTCTCCCCGCATTTATATGGCCGTATTCGTAACATACTTACATATAGTAATAAAAATATAAATAAATAAAAACATCTCTCTATGTTCATTTAATAAACAATCTAATTTGACTTCAGTAAAAAAAAGCATAAAATTCAACAGGTAATAATGGTAGGGTAACTATCAACACAAAAGTAAATTTGTCTCAAATTCAGTGATTGCTCCTCTATCACATTTAAAGAGTCATACAACTCAGTATGGGTACGGGTATCCTCTTGATTCCCACTACGTAACACTTCGGCTACGGTCTGTTTTCACAGATGCACCAACACATCCTCACATTTTACTGAAGCACAGCGATGGTAAAAGTTAAACATCCGCAGAAATTGCTCCGACGGCAACTGTTTACTAGGATCAGAAATTTGTCCGACGGCACCAGTTTATTAGGATGTCCCATATGAGGAGCAGGTTATTAGAGTTAGGTTTTGGCTTTGACTCTACTGCCACCTCATTTCTTCATTGCAGTTCTCCAACAGGCGCTTCGTTCAGCCACAAGAAATGGATGAAGTCATACAGCTTAGTACTCACGCTTACCTGTTCAGGTACAACGATGCATGCTCGATCAAATTGTCTTAGCCTGTGACATTAATAGCATCCAGATCAAGAGTAAGATTAGACTACCTTGTAAGGTGTTGGGTTCAGCCGCCTCATATGGTCTGGCCTCATTCGGTCCAGATGGTGAATACACCGAGTCCTAATCTCATGGATGGATGGAAGCACCTCCCGTGGATTCGCTTCACCAATTGACAACACAACACATTTTGACATCAATGGCTCCCAGCTTAAATAACAATTTTATTATCCGTCTAACAATCTAAACTAAACCAGGACACATACTTAACTGATGTACTTGATAATCCTACAGCTCTTGCGGTACGGTTGTTATGATAGCAGTGCACTATGCCTAGTGGCAGAAACCCTATTCTATATTTGGCTGTGGTGATTAGCATCATACGTGCAGTAATTATAAGTTATTTTAGGTAATGCCAATACTAGCAGCACATCATAGCCTACTACAGTACACATCAGTGTATTGTAAATATGCTTCATGAAAGACAACTAGCATGGTCAATGGAGCATGCATGCACAAGGGGCAAATAAGGTAACAAAACACTATGCAGAAGTAACTCAGGTGGAAGTTCATACATGAAGTTCCAGGCCAGTAGCACCTCAATAATTCCTCAACATGCTGCGGAACAACATACGCTCTCTTTGATTCGTTGAAAGGGTGACGACACAGGAGCCTTTCACCAATCTATAAACAAATGGTAAATCATAAAACCCACCAGCTGTTTCAGATTCCGTAAAATACTAATTATGGCATACAAAATCACAACTGAAAGAAGAACATGTAGGTGCCATGTAGCCTATAGAATTTTGAAGTGAAATATTTTTTTTATGCAAGCTCACCGTTTGTATTTTTAAGCGTTAACATGGATATTGTTTACCTTTGGACCTGGCTCATCTTCTCCAGTCATTATATCAACCAACGGATATCCTTCTTTGCCATACAATCTGTAGCATTTTTTCTTACATGGTATCGATACCTGGAGCAGACAACTAAGTTCTCGGTCTGTTTGGATGCATAGAAGACAGCTAAACTTGTTTCTGCTATGAAGTGATAATGGCATACATACCTTTGTGACATCTTCAGAAAGCTTAATGCGAGGCTGTTTGTTAATCTCGACTAATTTGAATACACAACCAAGTGCAGCTTGTGCATAGCATGTAACTAGGTTGGTGCCAATACCAAATGCATCTACCTCATGGCCCTACACGCGATAAATGTAATTAATGCGAGCATACATGTATTTATCGCGGCCTTGTACATTTTAACCTGCTTGCACAGCCATGTGTTGTGTTGAATTTATCATAAGAAAGAATTAAGCAGAGGTTTCCACTAGCCGGTTAAGTGCTACCAGGTATTTAGCACAAATAATTCATTTGTTCCTGTTAGTAAGGGACCATGCTGTTCCAGTCTGTATTAGATATGTGCTAGTCAGTAGCCATAAAGCCCATAACTACTAAGCACGTAGTTCAGTTAATGATATCTGACCATTGGATCACGCATATCATTAGCACAAACTTACACATGTCAGAAATTACAATATACATAATTTCTAAACCTGTTCTACAAGAGACAAATCTACATGCTATAACGTCAACCATTTTGGATGGATACTATGCGGATTACCTGCTTGTTCAGGGCATCTATAGTTTCTTCATTAAGATCATTGCTTGCTGTGATACTTGTTTTCCTAAAGCCAACAACTCCAAATTCTTTTTCAATTGCATGGAAGAACTTGCGGGTCTCAATGGATAGGTATGCTAAATCACCAGAGTCCAACCTAATTCCGACCGCCTTATACCTGCAATGAAAACTTCTGTAAGTGCCAATGGCAAATGCTTATCACTGATGTTTTCTTTGGAACACTTAACAATCACTAATAAAATCAATGAAGTATCTGTGACGTACATGCTTCTGCTCAAATTGCATATTTGACACTACTAACTCGAATTTGTCGTTTATAAAAGCATGAATTGCAAATATGAAACTGACATGTCAGCTCCATCATGCTGTGGCATGACAAAGTTGCCCCTATCCGCATGTCTGTCCTCTGCTTGCTGTCTGCTCCATCTGACAAGATCAATAGTCAGCCCAGCCCCCTAAGCTCCCAGAGGAGCTTGACCGCCCATGGCTTGTAGAGGACAGCGATGATGTGTGTTAGTAGCCATGCATGGTGGGATGAGAGGGGCAGAGGTTGGGGGTACTTGGGTCATTTGAACAGCGTCAAGTGAATTGGGCAATCCAGTGGCAGTTCACGCCTTGCCGATAGACCGGGACTTCCAATCATAAATTGGGTACTTGGGTCACTTGAGCAGGGTCAAGTGAAATGGGCAATCCAGTGGCAGTTCACAGTTTGCCGATAGACCGGGACTTCCAATCGTAAATTTATAATTTGAGTTTTTATAGTGGCAGATCGAATTTTTAGTCAGACTTATCAAGTGCAATAAAGAATTTTTAGAATCATTTGTTGCTGAAATATATAAAAAAACTAGACCAACCTAGGAGGAACTAGGTGGCATTTTATTAAGAAGGAAATCGGCACCCAAATTAGCCTTGACGAGGACTTGAACCTGGG

>BAC-24

TATACGAATCAGACTCTAATAAACATAGAAAAGAAAATTAATTCCTATCTTATTCCCTTGTCCGCCTAAATTACGATGGAATTCCCATCGACCTCCTTTCCATCGGCATACTCCCTGTTTCATCGGCAGCAGCCTTCAAGTCTTCTTTCATCGGCACACTTCCTATTTCATCAGCAGCAGTCTTCAAACCTCCTTTATCGACATCGGCAATAATACCTTTAACCTTATCGGCAACACCCCAATCCAACCTAGCTTCTCCATACACCGCCACCATCCATCGGCAGCCTTCTTCACAAGCTATCTTTTATCGGCTATCAACATACACAATAACTTTTCTTCTGCCGATTAGCCCACTCTGATTATCTTGACACGTGCCAAAAAACGATGTCAACAGGTATATTGGGTAATTCGGGTACCGCGACTTGTTACCCGAACTACCCGATATAATTTCGGGTATCTCAAGTTAATATCCGAACTACCAAAAAATTTTCAAGTTCGGGTAATTCGGGTCCGGGTCCAGAAATTTCGGGTTTGGGTAATGGGTATCGGGTATTTTGCCCAGGCTGAGCGGCTTCTCGTGCTCTCCTTCTTCTCGTTCCCCGTTATCCTAAAAAGACAAAAAACGCTTCAAAAAAAAAGAAAAAAAACCTGCCTAGGGGCGGCCGGCGCACACCGGCGAGTAGGCGGCGGACAAGCGGTTCACTCCGGCGTGGCGGTTGCGTGCGCAGCGCTGCGGGCACTCTAGGCGAGGGCGCGAGGCGCTGAGCACCTGCAGCGGCGTGGCGGCGGCGGTCGCAGCGCGGCAACGGGCGGCCGTCCGGCCGAGGCGGCGTCGCGACGAGGGCGCGAGGCAGCGACCACATGCAAAGCTGCAGCGCTGGAGCACTGAGTGGAAGGCGGAGCCGCGGAGGCAGCAGCCAGAAGCGCGGAACTGCTGTGAGCCTGGACCTGGGAGGCTAGCGGCCGGAGGCTTGCTGCTGATTGGAGTAGATGACTAACCGGCGAATGAATTCAAATTGTAAGTTAATTTGGTTAATTGGCTTACAGTTTACATATATTATCCATTGCAATTTGCCTATTAAACTGATACAGTGGCTAAGACAGTCATAGTTGGTTACTTGCTTAGAGTAATTTACTGATTCGCCAATGTTTATCTTTTTTGTTGTTTCAAATCCGCTATATATTTTGATATTTCCATTTTGTATGTTGAAAACAGTTTGTATTTATATATGTTAAACAATTTCAAATCATGCTCAATTATATGCTATGTATGCTATATATTATACAATTACTACGTGTTCGTTTGGCTGATAAGCCATGGCTGAAAGTAGCGTTCGCTGATTTGTTGTGAGAATGGCCGGCTGATTTGACTGATAAGCTCAACAACAACTATTTTGTTTTGCTTTTGTTCATGCACTGAAATCTATTTTAATTGCGTCTTTCCAAGTTTTGCATTTATGTCCCATTCTCTCAACTATTGTCGCTGGAATATGAGTTCAGTAGTATTTTTGAAGTGGTTTGATTATCACAGTATATAATCCACTGTTCCAACTTCCAAGTGCCTTAAATCCTGCTTATGATGGTTACTTTTCTCATGCCCATTCTTATATTGCCTGCAAAGTTCTGTGGTACCCCTGGCACATAATCAGATAATTTTGATGGCAGGGCTCTGTTAACATATAATAATTATCCTTTCTGTGGCAATGATTTATTCGCAAAAGTTTAGAACAAGCTTTAAACATGAAAAATATTATTCTACATCTTCTGAGAAAATAGTTCAATAAGGCATCTCATATGTTATAATATTACATGTTCCAAAATTTCCTACATCACTGTCTCAAGGTGTGCCTTCACAAACTGATAGCGTCTGCAACATTTGCCAGAGAATGATTCTCTGGGTACTCAATGCTGGAGCACGGGCTGCTATGAAGCCCCTCCTTGACAAGGGTGCTTGTTTCTGCACCTAGTGATATAAATATATCATCGTTTTTTTTGAGAGAGAGAAAGTTGAAGGGATAATATATTTCTATTTACAAGTAGGGCCCATCTTAGTTTAGACTACGAGTTCAAATAGGTTGGAGCAATTTAGCCCATAGCATTACTAGATGATATGTCTTTGCTTAGTAATTTATAGTAGAAATCCTATATGCTGTAGCTGTTGTAACTGACGGGGCGAGGTGTCCCTTCCTTCTAGTCTTATCTAAACGCTGAGAATTTGGAATCTAAAGAACTAGAAGAATGTCCCGCGCGTTGCTGCGGGAATGACGGGTGAAATTATACTCTTTATATTTACTAATATGAATAAATAAATATCACAATACTACAGGTTAGTGGATAATTTTTAGAATCCTGATGTGGACAACTAAATGTATGTGTTAGTAAATGATGTGGTTTGCTAATGTGAATAGCTCGAATGAAGACATAATGATATGTGTTAGTTGGATGTGTTAGTGGATGCTCATGTGGACACTTCGCATGACGAGATAATTGCATGTGCTCATTGGCGAATCCAGCCCGAGAAATGTCCTAGGGCGGACTTGCACCGTAAAATTTTGGCTAACCAACGATATCATGCATGTTGACGGAAAAATTTGTAGAACCACCGATAGGAATCGAGATGCTACCTTTTAATAGGGAAGT

>BAC-76

ATACATGTTATTATCAATACTATTCTATGAAAAATTGGATAAAACCAGGTTCATTTGGTTTGCGACGAGCTAGAATGATTTTCTTTAAGGCACACAGATAGATGGAGTATTCTTGCAGTACTAGTCCGATACTCGGGTATATTGATGTTATACAAGGACTAATATTTTTTCTTCAAATCAAAAGATGTAAAAATATAAAGTATCCTTTTTACTCACTGGCCAAAACAGATCGAGAACCGACATAGAAGGTAGTTGATGATTTTTTATTAACAAGAAAAAACGGGCATCTAATTTTGAAGTTGAATAGAGCCCGAATCAGAATGGACTCATTGTCACACCTTCCATCCCAATAAATTCGGCTACGCTCACTTCCTTGACTCAAAGTCACAAAAAGTCTCGACCCTGCACGTTGACACGTACTGTACGTACGAAGGAAGCATGCAAGTTGTTGTTAGTTTAAACTTAGGCTAACTGCATCACCAATAATGGAGAGACCTGACTGATGGGCGGATACATGATACATCACGCATCTCAGACAGAAGCAGCTGCGTACTCCAGTGCAGCCGGCCGCGGAGATTACAGGCAGATGGATGGATTGGCATGATGTGGGCAGCTCGTCCTCAGGAACATTGCGTGCACCTAAACCCTTCCTATCCTAGTCTATAAAAATGCAGCTCACTTTGCAACTGGGACCTGCATCACCAATATATGGCTTGTTTAAGGTCAGAATCTGATCAAAATAACATTGCATCCACCTAAGCACATGCATTTTCACCGGACACTTCAACTAACACGACGCCTTCTAGCGAGTGGCTTGTGTATGTGTATGCATGGGTCCACGCAGATCTCAGCTGCACAGTGGAAAGCATGTCATCAAAAGGACAAAAACGCATAAATTTTCTTAAGGAAAGCATAGTTCAAGTTCCCAGCGGGTAGAAAACAAAGCGGATGTTGTTGGTTCTTCCCCCGAGCTCTACGCCCCCAGTTGCCAGTTTGCCACCTCACAGGTGCCACTATCTTGGCGCAGTAGTTAGGAATATGATCTCGCTAGAGTTCTGATTAGAACCTAGACGGGCTATAGAAGCCATGACGATCCACCCCGTATCAATAACCATTTTGTACCGTCCCGACCTAGGCTTGTTCAATTAGTCTCTAATTCTAGGGGATTGGAGGACATCGAGGGAGTTTTGAACAAGTTCATAGTGGGCCTTACGTCAGCCTTTCCTCGTTGCACAAGTCTCAATAAAATTCAAGTTTTTAGAGGTGACTGCTTTATAGTGTTAATTATAAGATGTAGATATTGTCGCAAATTCCCTGATCCACATTCGTCCCCTAGTAACAGATTAGGCGTGGAGAAAATGATAAACGACCAATGTCCCTTTTGGATCCTACTACTCTCCCGACAACAGATTAGGCATGGAGAAGATGATAAATGTCCCGTCTTCCTTCCAAACCCCAAACGAACATGAATGAATAACACAGAAAACGTGAAAAGGAAATTGGTCCATTATTATTCATATAAAATATATGGGTTCTTCGGGGCAAAGCCAGGGCCCTTTTGCTGGGGTTCAGCCAACCGTGACGGTTTTCAAAAATTTAGTCTAAATTACTATAGGTGTGTTTGGTTTAATGGATATCCATGAATGAGAGATGGCGGTCCATGTATGAGCAGCGTTTGGGTCGTGGACGACTTGGACAAAGATATTCTATATAGGCGTTGTAAGCTCACCCATGAAAATCGGTTGGACAAGTATTTCCATGTCTTCTAATATCTATCATTAGTAAATAAATTAAGAATATGTTATATTATTACTTAGTAATTATGAGTACTATAGTGCAAGGTTAAAGACCACGGTGACGTATTGTGCTGACTTCGCTCCTGGGTTCTTGTTCCTTTAGATAGTGTCAGTTCGATATAAGCCTATAATCATCCCTCATCCCGGGATATATCTTGACCAACAGGATAGGACCCATATAGAAAGAAAGAAAAACATCGTTCACTTGAATTTTGATGAGGCCTTGTTTAGATACCTAAATTCACCCCTAAATTTCACTATGCAAAAAGAAGATTCCCCATCACATCAAAATGTCGGCATATATATGGAGTACTAAATATAGATGAAATCAAAAACTAATTGCACAGTTTTGTTGTACTTTGCGAGACGAATATTTTAAGCCTAATTAATCAATATTTGGACAATTATTACCAAATAAAAACAAAATTGCTACAGTGTGACAGGACACTTACTATGCAGATTTTGGTTGCCAAATTTGACATCTAAACAAGGCCGAGGAGCAGATGCTGGTCATCTCTCCAGTTTCCCACGCAGATAGTACAACGCAAACCACGATGCATCCAAAGCGGAAGAGAAGAAACAGTGAAACACGTTGGCCTAGGCACGACCACGCACGCGGTGCAGGAGAAGCGGTCAGGTCAAAAGGAAGGGCGCGGCAAAAATCCAAGAGGGCGCAGGCACCAGCCTGCAGCGGAACATTCCCCCGTGCGCCCGGCGCGCGGACGTGCAGATGCTTCCACTCCTCCGCGCTTTCCATGCTCCAGTCTCCAGCATCAGCCGCCGAGGGGACGGCAGGGCGGCAGGCGGCAGCAGCCACCGGTTCGTTGAGCCTGAGCCGGGCCATGGGCACGTCAGGTGGTTTTCTCGGAGACCTCGCGCGGCGGGCGCCGCTGCCGCGTAGGCGGCCGGCCCACGCGCTGGGTGCGCCGTGCGCGGCGTGGATCCCGTGTCGCCCGTGACCGCGCGTACGTTCTGAGCTCCACTCCACGCATCGGTGAGAGGGCGCATGGTGGCTCCACGTAAGCAGCCGCCAGGTGGATTCTCAGCTGTGCCAGATCGGCTGGTTAGACCAGGGTCTTTTGCGACGTAGTACTAGTACTACGCGGTACGCCTGCTTCGAGATATGGGCTGGCATGCCGCCCTGTGGCTGAAAGTCTGGATGAGTGGATGTTACTCCAACGTTTTAGCCGTAAAGCTGATGAGTAATTTCTGCAGGATTCCTTCGGAATACAGCTAGTAGGATTCTGTAAGCTGTTCCAGCACATCAAAATGCTTGAATCTTGGTGTATATACTAATGTTCCACCTGGCAATTTTACCGGAGCACGCGCAAACAGAACACAGATCGCCAAATCCCCGCTGCTAAAATGCAGCCCACAGCTCAGTGAATGCTTAGATCTAGCCAGTTCAATGCTCCAGAAAATTGACAGCCATTCACGTGGTACTAATTCGGCAGGCAGTGAAAAGTCCAACACAATATACAAACACAGAAGATACTGAGAAAGAATCACGCACATTCTACAGCCTGTAACATTAGCAGTGTTCCATGTTTACGGTGCATCGAATGTCGCTGTACCCAGCAGGTAGATCGGCTGGGCAGGGGCCGCAGGGCGCAGGAAAAAAAAACGAACCAGAAATGAGAAATCCACCGGCCGGCGGCCACCCACGAATCCACAGCCCCGCAGGCCGCCCGTGCTGCCACGCCGGCCGCTCGCAGCCGCAGAGCCGGTTGACTCGAACACAAGCCAAAGCAGAAGCAGCAGCCTCGTTGACCCTCCTCCACAATCCTCCCCGTCCGCTGTGCCCATCCATCCCATCAAAAACCCCATCAAACCCCCGGAATCGGCCACTAAACGCCGGCACCCACCCGCCCAACCCACCGCGCACCAGTTCATCTCATCAGTCATCACACCTGCTCCTGCACACCGCTAGCCACGGTCACAAAATTCCCAGAACGGCCTTGTTTAGATTGCAGAATTCACTCTCAAATTCCACTATGCAAAAAGAAGATTCTCCATCACATCAAAATGTCGGCACATATATGGAGTACTAAATGTAGATGAAATCAAAAACTAATTGCACAGTTTTATTATACTTTGCGAGACGAACATTTTGAGCCTAATTAATTAAGATTTGGACAATTATTACCACAAACAAACAAAAATGCTACAGTGTTCGGGGGTGGCACTGTGTAGATTTGGCCACCCCCGAATTCGTGTTCTAAACAAGGCGAACGATTGAGTCGTGGAACGAGAAACGTAGACCTTGAAAAAGATTTTCCACTGTTAGGATGAAACTGATCCACGTTTTTAAAACGAGAATGTACGTTCCCGAAACGTGAAACGCGCCACTCTACCCCGTTCCCGTGACTATGCCGCTGGCACGGCGCTCATCATCCGCGCTCGGCTCCTCGCGATCGCGACTCGATCCGGCCCCAGAAACACAGAGCCGGCGCCGTCAGATCCCAGAAACCCCAAACACCCAGCGGGTTCGCCCTCCTGCTGCTGAGTAATAAAAAACAACCGAACGTGGCAGCAAATCGCGGACAGCGCAGAGGAAGGAAGCAAAAACAAACCCTTTCCATTCAAAGCCCCCGAGAATTTCAAAAAAACATTTAACAGCTTCCTCCTTCCTTGCGCAAAACAAAACCCCGCAAAACAAGCATCGGCTTCGTCCCTCCTCCACTTCGTCTCTCTCTCTTCCCCAGCTCCGGCCGCGCCCACCGTCGCAGCCAGCGCGTGTGCTGCTTGCTCTGCGCTCCCCGCCGTCGCCGTGGCCGCGGGCGAGCTCGAGAGGTGCGGAATTTGGGTGCTGCTCATGCTGGGTTCGAATCGGAGGAGGGAGAAGTGGAGGAATAATAATTGGGAGGCCGTTGGGGGAGTGTAACTTTGGGCATTAATGGCGGGGTGGTGAGAGACCCCCAGCCCAAGGGAGGCCTCTCTGTCTCTGGTGGTGGCGCCCTGTAGTTACCTGCGTAGCTCGGCCTCTGCCCCCCTTCGCCTTTATTGCGTTTAATTTCGCTCCGAATTCCAGCGCTTCCGCCTCCTCCCCAATCCCATCGACACCTGTAACGCGGAGTGGACATGGGGGACGGCGGCGGCGGCGAAGGCAGGAGGAGGCGCTCGAGGTGATGATGCGCGCGCGCGCGCGCGAGCTCTGGGCGCTGCTGCTCGTTGCGCTGGCGGCGGCGCCCGGGGCGGTGCTGGCGCAGGGGAACCTGACGTCGCGGTCGGATCTCGTTGGGCTGTACGCGCTGCGGGGCTCGCTCGGCCTGAGGGCGCGGGACTGGCCGCGGCATGCGGACCCGTGCACGGCGTGGGCGGGCGTGGGCTGCCGCGCCGGACGCGTCGTCTCGCTCAACCTCGTCGGGCTGCGGCGCACGCGGCTGGGCCGCCTGTCGCCGCGCTTCGACGTCGACGGGCTGCGCAACCTCACGCGGCTCGAGGCCTTCAACGCCGCCGGGTTCGGGCTCCCCGGCTCCATCCCGGCGTGGCTAGGCGCCGGGCTCGCGCCCACTTTCCAGTCCCTCGACATCTCCGACTGCGCCGTCTCCGGGGAGATCCCGGCCTCCGCGCTCGTGGGCCTCGGCAACCTCACCACCCTCAACTTCGCAGGCAACCAACTGTCTGGGCAACTGCCGGCCACCGCGTTCTCGGGGCTCACGCGGCTCAGGACCCTCAACCTCTCGGACAATGCGTTTTCGGGCGCTCTGCCCGACGCGGTCTGGTCGCTCCCGGGGCTCACCGTTCTGGACGTGTCTCGTAACAACCTCACCGGCGCATTGCCCACGGCAGGGCTTGCGCTGCCAGCCAATGCGCAGGTGTTGGATCTGTCGGCGAACTTCTTCTATAGCGTCGTGCCAGAGACCTTTCGCCGGCTGTTCGCGCAGGTGCTGCTGGCCAATATTTCTGGCAACTACTTCGACGGCAAGCTAGGAGTGTCCGATGGTGGTGGTGGCAATGTTTCGTTTCAGTTGAATTGCTTCCTTGACGTTCTGGGACAGCGTACCCAGGCTGATTGCCAGCAGTTCTATGCCAGGCGTGGCTTGCCATATAATGGTCCAGTTATGCCACCCGCACCACAGCCTGCACCTTCACCGGCAAGGGAGAAGCACAAGAATCTGAAGTACATATTGATCGGGGCCATTGGTGGAGGCCTCCTGCTGATAGCCGTGGTTGCAGCCATTATGTTCTGCTTTGTGTGTTCTCAGAGGACAGGGAGGAGGAATGATCAGAGGGAAAGTGGGGCATCACCAAGTGCACCATCGGGAGTGTCAGCTACCGGCACAGCTGCAGCTACTGGTGGCACACAGCCTTCTGCATTGTCCGCAAACACGGCGAAAGTCGGCGATTCATTTGCTTACGACCAACTTGCCAATGCCACCTCGGGATTTGGGGAAGAGAGGCTCATCAGGCACGGTCATTCAGGTGATCTATACCATGGTGTTCTCCAAGACGGGACTGCTGTGGTGGTGAAGAGGATCACTTCACGCGTGGCTCGGAAGGATGCATATCTGGCAGAATTAGATTTGTTTACGAAAGGATTGCATGAAAGGCTGGTTCCTTTTCTGGGGCACTGCCTTGATAAAGAAGAAGAAAAGGTTCTTGTGTATAGGTTTGTCCGAAATGGTGATCTGTCAAGTGCACTGCACAGAAAGTCAAGGGAGGAAGACGAGGGCATGCAATCTTTGGACTGGATAAAGAGGTTGAAGATTGCAACGGGGGTAGCTGAGGCTCTTTGCTATCTGCATCATGAGTGTTCTCCACCAATGGTTCACAGGTATAAACTTCTATGTTGTTTTGTCAATGTTATATTTGTTTGCTGTGTGTCTGTTAGCAAATTATTTTTGTTTATAAGATGATAAAGTTTTAGAATTACCTTAGAAATTCAATCTTTATCATTACATGGGGTTTAGTGGTTGACAGTCGGTCTCCACTATCTGTGTTTCACTGTGGATGCTCTGATAATTGTTTTTCTTCGTTTTGGTACCAGAGTTTCAGCAGTTACTGCAATAAACTTTCCCCATAGATTCGGTGACAGCTAGGAATAGTACATTGAACATATGCAAATGTGTGCCAGTAATGCTGAATCAGAAAATTAGTGTTTCGTGCTTGATGCAACATATTCCGTGCGTTATCTGAGTACTGGAAATTCAATGTGGACACTGCTGTTATTTGCTTTAATATCTTTGCTGAGATGTTTCTGCTGGTCTATATCATTTAAATACCCACCAATTTTGCCATAAAAAATAACGGGAAGTTCTTTGTCACCCTGATTTCACCAGGGATTTTTTTGTTTTGTTATCAATGTGAGGCATGCAGTAGATCGCAATCAATTCTGTTTGTTTTGTCGTTGTCAGTTCTATATGCCAACACGTAGGATTCTGACTGTGTAGATGCTTCCTTCCAAGACCAATAAATTAGATGCAGTGCAAACGCTTGCTGTTGACTAATTTTGTCGGTTCTCTACTACAGGGATGTGCAAGCCAGCAGTGTCCTTCTTGATGATAAATTTGATGTGCGCCTTGGTAGCTTGAGTGAGGTGTGTCCTCAAGAAGGGGAAGGTCACCAAAATGTTATCACAAAGCTGTTAAGATTTTCTTCGTAAGTTTCAGACACCATCTCTGCATGGCTTGTTTCTCTTTTCGTCGCTACATGCTTCCTTTGTGGAGCATGCAAATCCATACTTATATTGTATTCACTATTGCACCATCCTGTTTCAACGGTAGTTTTGTTCAAGTGTTCACCATGAAATTAGTATTTACCTGTTGTGTAGCATGAATTTAGATGGATTACCTTTTTAAATGAATAATTGGATATTTGGTGAATTTAAATAGCGTGTTGTCAACATGATTCCAGTCAATTACTAGGTGAACCTGCTGTTTCTTGGCCAGATCTTTGTCATGTTGACGCTGTTCTGTGGCACTATGCAAAATCTAAATGATGTCAATAAGTACGATAAAACTAATGCTTGTAGGACCGTGAGAGCGTAGGTAATCGTGAACTATTTTATTTCCGTTCCTTTCATTTTACTTGCCATATCTTATTGTCAATGGACATGCAGTATCCTTGTTTTAGTTGACAGTTACATTATCAAATGCTTCCTTGGTGTAGCTTCATTTTTCTAATATCAAATTAGTCCCTTTCCATCTCCTGATGCTTTATTTTTGTGGGAATGTAGGACGGCAGATCAAGGTTCTTCTGGTGAGTTTGTGCATGCAACCTTTCATTGCTTTCAAGCATTTTTAATCTTGAGAATTGGTCATTAGTCCTCAGTTTCACGCTAAAATTGATGGCTCAACTGTCCTATTGTCTGTGAAAAATGCTTTGAACATCGTCTCTAATTCTATTTTTTACGTTATGCAAGTTAATTGACCAACTTTCTCTGTAAACAGGTTCCCCATCTGCAACTTGCCCATATGATGTCTATTGCTTTGGAAAAGTTTTACTGGAGCTGGTGACAGGGAGACTTGGTATCAGTGCATCAAATGATGCTGCCACAAGCGATTGGCTTGATGCCACCCTGCGGTACGTCAATATTTACGAGAAGGAGCTTATGAGCAGGATCATTGATCCAACGCTTATCATTGACGAGGACCATCTGGAGGAAGTCTGGGCAATGGCGATTGTTGCCAAGTCCTGCTTGAATCCTCGGTCTTCTAAACGCCCACCAATGAAGTACATCCTAAAAGCACTAGAGAACCCTTTGAAGGTGGTGAGAGAAGACAACGGCAATAGCTCGGCCAGGTTGAGAGCAACATCGTCACGGGGTTCATGGAACGCTGCACTCTTTGGGAGTTGGCGGCATAGCTCGTCTGATATAGGTCCTTCTAGGGATGACAACATTCTGAAACGCTCAGAGACAATCAAATCGTCTGGCGGAAGCAATGGGGATCATTCTTCCTCCCGTAGGAGGCAATCCAAGGAGATCTTCCCTGAGCCGTCCGGCTCTCGTGACACCGAGGATTAAGGATGGAGGAGAATCTTTCTTACTGATCATTTTTCGGTAAAGAGGGAATCCCTACGGATGAGGCCTCTGTTAGAGCTCCGCTGGCTGCTTCTGATAGAATTCTTTCGTCCAAAAAGTCAGGAATGTGAATTCTCTCTTCTAGTCAATGGCTTCACAACAGCACACTGGTGCCATGGTAATCATTTTTGGGGGAAGTAGCAATGGTGGCATGTACGTCGTTGGTGTACCTTGGTTGACGACAATGGCATGCAGTCGGCGCAGTGAAAAATTCTTCACAGTTGGAAGTTTGGTGTCTTTTCTGTCCATTATCTCATTTTCCCTTCCTCTCGGTTAAAGAAAGAATGGCGACCTGATTCTTTTTCTTCTCCCTACTGTAATTGATTGTTTCATTGTTGATCATAGCAGAAATGTTCTTTTCATTCATGCTTGCCATTTTCTTTTTCTTTTTTTTCTCACTTTGAATTTCTTTGTTTTCCGTGCAAGTTTGATGTTGAGAGGGGGCGGATCGCGCCATCCTAGGTGGACCAGAAACGTTCAGAAAAGGCCAAAAATTTGATGGGACCTCTATGAAAAGGCCAAATCTGCAAATCCCTTTTGGGAGAGCTCTTGTGCTGTACTGGCCCGGCCCAATAACAAGTGACGGGAGTATTATAATACATGGCGCCTGTAGCTCAGCGGATAGAGCGTTTGTTTCCTAAACAAAAAGTCGAAGGTTCGAACCCTTCCTGGCGCAAACTATTTTATATTTTTCCGGTGAACTGCACAAACTTTTTTAAAAAAAATTCATGTGAACTGCATAAATTTGTGTGTGATCTTTTCTTTTTATCACCAAGCTTTTCCCTTTATATTTTCAGGTGAACTGCACAAACTTTTTTAAAATATTTTCATGTGAACTGCACAAATTTGTCGCGTGATCTTTTTTTTATCACCCAGCTTTTCCCCTATTTCTTACGGTAATTCCATACAACACTCTAGTGTTTTGGAGCAATCCAACACTCATTCGATCTCTTCTCTTTCTCTCCCTCACTTCTCCAAATCCGGCGGCTTTAGAAGAAAGTTTTTGGGAGGATGGCCGGCCAGGCGGTGGGGACAGTGGGAAGACGGCAATTAGGTTCCAACGGCGTAAGAGGTGGTTGGGCCAGGTCGGGGTGGGGGCTCCCATGGCCCTGGCGGTAGCGGGAGCAGGGGGAAGGAAAGGAGACCGCCGCATGCAAGTTAGAGTCTCATCAGAGCTCCACAGCCATGGCCGCGACAGTAGAGTTCCGATGAGACCCTACCACACCGTGGCTTGGCAAAGCAGCGGCAGCGCTTTGGGAGGGAGAGAAAGAGGATGCAGTCCAAAAGAAGAGTGTGGGAACACTCTAGTGACGAGTAGACGTACCTTTTTTTTTTATTACCAAGCCTTACCGCTGCACAAAATTTCCATGTGACCTTTTCTTTTATCTCCAAGCCTTAGACCTTGTTTGGATGTGCATGTATTTGTCTTAATCTACATGTGTTGGAGTGGATTGGCTCCAATACGCTTCAACACATGTAGATTGAAGTAGATACATGCGCATCCAAACAAGACCTTCGTAATAGTGGCGCCATCAACTATTCCCTACCCCTTTCCCTCGTCCTTTCCCCCTCAGCTCCTCACATCTTCATTTCGTTAATGGAATTACCATTTCGTTAATGGAATTACCGTCATCAAAATTTCAAAACAGTAGACATCTTAGAATATAAGACGAAACACCAGCAAAGAAACAGAGAAGCAATTCATAGGTTTCAGAGTTTTTTTTTTGTTTTCTCGAAAACTCATAGGTTGCAGAGTTTCTTCTATGCCATTTAATGAAAGAGGCACTTGCTGTTTCAAGCAGAGTTAGTACTAGGACGGAGCTTAGAGCCTTTAAATCATCAAATGAAACACCCGATTAGTGAAAAGAGAAATATATAGAACAAGGAATAAGAGAAAAATGGTTAAGCATATGTTAGTTATTGTTCCATGTGATAATCACTGACACATAGGAGTACTTATTCAGTAATGATTTAAGTACAACAACTGGGCGGTACCTTGTATGCTGAATAAACTTCCAGTATTGGAACAACTTTTGAGGACGAACGAGAGTATACCATGTTTCTTATTTCATTTATTTCTCTCAAGATTCTGTTACCCCACCAATCTCAATTTGCTCATACAAAGCAGATACTCTTCAACTAGCTTTAGCAGCAAGCAACGATGGCTGTGGTGCAACCTCAGGAACCAAGGTCCCATTGATGGCAGAATTAGCGTGGTCAGTTTTTGCATCAACCGCCCAAAAGTTTGCTGTTTTGATGTCATCTTGTATCATTAACTCAGAGTACTCCTCGTGGTTGTACACCACATTGTTCTTGCCCCCAAACTCTATAGGAAGTACTTCCGGATCAATGTATTTATACATGACCTTCATGCTCTCCTCATCCTTCTGGTACACGAAGTTCACCTTCTCAATCGATTTTGGATCAAGGAAAATTTTGATAACCTGAAAGAATTTGAAACATTTAAGTTGGAAGAATGAAATGCAATTTTTTGTCTGTATCAACACTACATGTTGTTTTTTCATTTGTTGTGGTTAAAATCAGATAGATGCATAAAGGTCGAGAGCACTTACCTTATAAAAAGCTTCAAATACTTTTGGGGGATTAAATAGAAATCCAATGGCCAGCCTTTCAGGAAAATGATTTTGCAGGATATTTGCAGTTTCTCTAGAAGTCTTTATGGGCGAAGCATGGGCCACTTTCCATCCAGTGAAATCTATTAGCCATACCATTTTTTCTTGGCCTTCAGGTAGGCTGAAGATTGCATTCTCCAAAGTATATACTAGGAACCGTATCTGCCCTTCATGGGACGAAGTATTCTGAAATTTTGAAATAGCATACAGGTGAATCCTGAGCAGAAGAAGCTAGGGAGACTGAAAATTTTCAGAAACTAGAAAAGAATAGCAAGGGTGACACTTTAAAACAGATAACTTCTTACCTGTTTTGTAGGTTTCATGACAACAACAGTTCTGCCCTCCCTGTCTCGGAAACTTGCCCTGTACATTTTACCTGTTTCCGCTTCAACAGAAACATCAGGCTAAAAGGCAGTTAATATGATGTTTAGTCACTTGGCTAGGATTTAGCGAAATAAAACTAGAGCAATGCATGGATGGCAAAAAAAGGAAGAGCAAATTTTACCCAACGAATATCCTCTGGCCTGTAAGCTGCCCTCCACTTGAGACTTTCTTCCAACATTTTGCTCGCCTTAGCAACATTCCAGTTACGGGCTTCCAGATATCTTTTCAAGCATGCTTCACTGCAGTACTTCTCGCCACGGGCAGACGAAGTCCCAAGTGCAGCTTTCAGTTCACTTATCTGCTCATAGGAGATTGTTGCATGAGCATATGTCAAGAAAATAGCTGATACATCAACAAAGAAACTAAAACCATTTGTCAGGCCTGTATGCATTAGACATGTATAGGCTCAATTCTCCGCGATATGAGTTTGTTGAATGGAGGGTTTGTGTAACAACTAGTAACAGACTAACATAAATTTGCTCAAAGTTAGCAGACATATTACACATAGCCATGTTACAACTAGATGAACAGAAGTACAGGACACTTGTCCATTCAGACACATTACACATACACCTTCAGTGATCATCATGTGCTTTTTATGTACAACAATTTCAATGTGAAGTACAACTTTAAAACAATTTAAATGTACCAGAGCATAACATAGTGTTAGAAAGTGGACCACAGTGTAATTGGATGGGATATTATTGTACATGCATAGATTGAGAGTCCTGACATCCTAAAATAGTACTACTTCAGTAACAGGTGAAGATCAGAATGGCAATGGTGACTAAGTGTCCATAGAGCAAATAAAGTGGTATAAACCATTTGGCTCATGATGTAGACATATAATATTCCGAAGAGAGACTGATATATATCCCCTTTGTTGTTGACTTACTACACCTTTTAGGCCCCGTTTGGAATGCAGGAAATTTCCCTTGTTCCTGCGTTTTTCCTATGAAATTTCTAGTTCCAAACAGGCCCTGAAGCAGCAACAATCACTGCTAGATCCAGTTCATAGTACATGTTAATAGTACATAAATGTAAGCATAGGCTGTCCTGTGGTTGGCATCACGTTTGGCTACCCTTTCTTGTACATAAAATTCATTTCATCTTAATTGAAAGGCAGAGCTCCTGCCATTGTTTAAAAAAAATGTAAGCATAGGATGAGTTGAAAGTGTTAGTCAGAGTTCATTTAGCTTCTTTTTTATTTCCTTGGCTGGTGCAAGGGATCAAAATAATAAGTATGTGACGAAGAACGTAACAAGGG

>BAC-66

TTTCATGATCAAGTTTTAATTGTTTCCTAACATCTGAGATGTTACAATACCCATACCTACTAGTAAAATAATCTAATATCATTAAAATCCCTATACCCAGCTGCGGGTGGGAAATTTATCCAGAACCATACCCTGGTAGGTAAAACTAGATCTAGCGGGTTACCTATACCCACCAATAATGAAGGGTGCAGATGGTTTTCCAATTATTAGTGCTAGGCTACTTTGATTTTGGCCAAGGGAGGTGGAATGTCTACATGGGCTTCCGAGTATTTTTCACCCATGGGTTAATGGGTACGTGGATTGCTAAAACGTTTCAATCCCCGCATACCCAATGGGTGAAGGATTTTGCCCATTTATGTACCCATGGAACATATTTAGTCCATATTCGAACCTTAATAGAGTAAATATCCGTCGGCTATCGAGTGGAGGGTCCCCATTACCATCTTTAAGGCGTGTTTGGGACTGCTCTACGAACTCTGCTCTATAAACTTCACCGTGGAGCATCTCCAAAAAAAAACTTAGAGCTTGTAGAGCACCCGTTTAGATGCTCTCACAATTTTACTTTTTTTTTCTCGAATAGAGTACGTGAAGCTGGACCTGTTTGCTAAAAAATGTAGAGCATAGTTTTTTTAGCCAACGTGGAGTAGCTCTATATTTTTTTTTAGCTCCGCTCCGTGATATTTTTAGCCAAACAGGTCCACGTGGATTTTGCGAAGCGTTCGTTTGTTAGCCAGGTCCACGACGAAATTCGTGGAGTGAAGCAGTCCCAACTCGTCCACAGTCTCGTCACAACATACACGGGCCATACGGCCAGACCCTAAACCAAAAGCCTGTGCACAAGTTACTGGTGCGCCCGAAACTCGCAAGTCACAACCACCCGGTCCAGTGCGTCGCCTGTGCACAAGTTACTGGTGTGCCCGGTGCGCGGGCCGCGTGCATCCTGCACAGCCGAACCACAAAACCCCACCTCACCACGGGCCACCACACCCCTTCCAGTCTCCACCTTGTTCCACTCTTGCTCCCGGCCTCCCGCTCGCCTCAGCTACTCGCTGTTCGTCGCCGGCCTCAGCCCCTCTCCCCCGCCGCTTAGGGTTCGCCTCCGCCGCCGCTGCAGGAGGTAGGGTTTCCGGCGCCGGGCGCCGGGGTATGAGCAGCAGCCGCCGCCCTGCAGGATCTAGGGTTCTGACGACTCCGCTCACGGATGGAGGGTGGTAGAAGGGCGAGCGAATCCCTCCCGGTACCTCTCCATTCCTCCTTATATTTTTTCTCCTTCACGTGGCGTGCGTCGCTCCTGTGCCGCAGTGTGCGAGGCTTGTAGTGGGTCACCCGGGTCACGCGCGCGTAGCTTGGTGGGTTAGGGTTTGGGTTGGGTGACGCTTGAGCTTAGCCGCCTTGTTTTTCGGGTGGTTTCGAAGGCGTAGTTACCAATTCCAGCTTGACGCTGGGAATTAGGGTTTGGAGCCTTTCGTTTTCAGTAGCCTCATGTGCTTTGCGGTCAGACTGGTTCTGGGCTTGGTTCAATCCGACTGCTGTAAATTACAGCTTTACTTGCTTAGAGTAATGTTTTGCTAGGAGTTTAGTGCTAGCGAGCTCTCCTGAGGTCAAAGTGCACCGCGCATGTTCTATTCATCAGGCAGGAGGGGGTATGGTCTTAGTTGACTGAGAATGTTCTGCTAGCAAATGTAGAATTTGCACTTTTCTTTTGGCGCTTTTAAAACGGCCACGCCAATCGTTATGGTTCAGTGCGTACTGCAATTTTGTGCGGGTTATTTGTTATACAACAATCATCATTACTGGGTACTAAGTGGCAGTCTTTTAGAGTCGTCTGAGATGCTGCCTACTGCCTAAAATACTACCTTGCTCTCTCACATACACTGATACACACTCATACATGATGTTTTGTGCAATGATGTTCACACATCCGTCTGGGTAGTTTTGCTCCCATATATGAATTATGTTATATATAAATATGATGCTCCAATATATTTAGCTTGCTTGCTGCAAAGTTGCTGACTTATGTTTGACTTGTTGGGACTTCAACTTTATATGTTATAAACTTCTCTATTTTGTAGGCATACACCCATGGATCATGACAATACTGATTTTCAGAGCCAAAACTTTCAACTAGCTGGCGAAGGCAGTAGCAAATTCCCTTCAGGTTTGCGACCATTTGCACTGCCAAAACTTGATATTGAGGACCAACTACAAGGCCATCTTCGATTTGATAATTTGATAGATTCAGAAGCATTTTTCAGCGTACAAGGGCACGGGAATAGTTGGATTGAGGTTCTGTCCACTGGAAGCAGTGTTGTTGATTTTAGCTCTAGCGCAGCTGAATCTTGCTCAATTTCTAAAACTAATAATGTCTGGTCAGAGGCAACATCCACAGAATCTGTCGAAATGCTATTGAAATCAGTGGGAGAAAACGAGACAACTGCTCACCTTCAGTTAAGTGGTATGGACAGTCAAACTGATCAATCGAGTGCTCATCCTAAATCTGGTAACTCCCCGACAGATAGTACTGTAGTGCCAACTGAGAAAGATCAGTCTTGGAGCACTCATTCTAGAATGACAGACGATCTTGATCGTTCTCAGAGCACTCATTCTAGAATGATTGCTGACCTTTCAAACACCGAGTCTCAGCTTGAACATTTTGCACCTTTCTTAATGGACAAGAAAGCTGAGCAGGCAGCAGATTCTGTTGCTGAGAAGTGCATTGCAGGTGAAACTCTATTGTCAGAAAGTTCTGATGGATTACTAGAGGCCATCACAAATCCAGTTAAGATGCTGTACAGGAGTGATGATGCATGCAATAAAGTGAACAGCACTCTTCAACCATCTTTTTCAGCAGTGCAACATGGAACTGAAGGCCTGAAAAGTTCTTCAGTTCACAGGAGCAGTGAGCTCGTTGTTAAGGAGTCTAGTACTGGTTTAGATTCTCTTCTATCTGACCAATCTGAGGCAGACCCAAGACATTCTAATTCACATCCTGTCAGTTCTGTGTCTCCCAAAAGTAAAATTGCCGATGCCACTTGTGTTCCTGAAGAAACAAAGAATGCTGGAGGCAGTAGTACAAACACCTGCTGTACAGGTGATGAATCAAAACATGTAGTGTTGGAGCACGATCAAGATTCTGTTGACAACCAAAACAGTGGTGACATGGGAGAAAAGATAATTGAGGAGGAAATACCAGCAGTTTCAGGGAACATTGAGCAAATGATAGAAAATGATCATGAAGAAAATGCTACCAATGCTACTGGTACATCCAAAGATAAGGTTGGCCCTTCCAACAGTATTGCTCCTGAAAACTTTGCAGCTGGCACTTTGAATGCTTCAGAGGATCCAAACATTCCCTCGCTAAATCATGAGAGGTCATTCGAGGAACACGAATTGCCTGCTTTAGGAGAGGAGCCTAAAAGCAAAAATTTACTCCTATCTACTTCTGGGCTTCAAGAGAAAATTTCAGCATCAGTGATCAGTTCTAGCAGTGGTGTCACGCCTACCATTGTTACTGACAATGTTGGTACTTCTGAGGATAAAAATGGCTGTTCAATAGGTGTTTCTCCTGTTGATTCTTCTGCTTTACTAGATAAAAAGGATTTGAAAATGGCCACAGTGAATAATGAGGAGGCATTCAAGGAAGGTGATAAATCCGCTTTAGGAGATCAAGACCACAATGGTATTTCTCCTGGCTCTGAACAAGGAGGGGAAATGCCAGCTGTGCCCATGGACTCAAACATTGCCGTTTATAGTGGTACTGTTTCTGCTACAGAAAAGGAGAAATACAAGGAGCAGCCTAATTCTTTGGGTGGTTTGACCACAGGAGAAACTCAGGATAAATCAGGTGAGCCTTTTGTTTTTTTCCTCCTATCATGGCCTGAGCTTGTTGGGCATATTTCTAACTGCTGTGTGCATTTCGAGCTTGTTTTACTTGTAAAACTGATAATACTGGGATTCATTCAGGTAACCATCCAGATGCTCATTCACTGAAATGCCAGACTGATAGATCTTCAATACTTGCAGATCGTATAGATCCAACCACAGCACCTACGTTAGGTATCCCAACTGGAAAGGTGGCTGAAAATATAGTAAAAACTCCACTGGATGCAAGTGATGATTTGAATGCACATATGCAAGGTCAGTTGTTTGTGCTAGCACAATGTCAATGCAGTTATTTTCCTTCCCCTTTTCACTAGTTCGAAGCAGAAAAATAGATGTTTCAAACTTAGCACATTTATGGTCTATCATGCATGCATCACAATGTCTTTCCTAATATGTGCCAGGTGCACAATAGGTCATGTGCCAGGCTTATCTTAGTCATTTTGGTGGCATTTATGTTCTATTATCACTTTTTGACCTGTCTAATGTGCATGGTCAGTGACTTTAATCCCCAATCTGAGTAAAGTAACTTAGATTGGGAGATGGAGTGATAGACACATCTATGTAAAGCTGGTATCCTCAGCATACTCTATTATGCTTTCTATAGAGCTGGGACAAAAGCCTTTGGTCTAATATACCAAGTGTTATAAGCATTGCTAACGTAGACACCAACTGTTGTCAATGCAAAGTGACATGTTTTTTTTTGGGTACAGATATAGTGTTAAATCACGGTACAGATTGTAGTCCTGGTACTGTGCCATCCCAAGGGAGGCAAGGCTCAAGCTTATTGGAACCTGGTAATGGTAATGGAATCTGCACTGGGGTTACAAGTGAGTCCCCTTCAGTGATTAGTTGCGCTGAATCCTCTCCCCAAGAAGGTGGACATGACAGCAATGCTTTACTTCATCACACACTATGTGGGCAATCTGAGGATCCTAAAGATCGTGGGGCCACAGCTGATGCTACCCAGAGCTCAAAACAATGCTGTACCAGAAATATAGAATCTGCTCCTGGTTCTGAGGATGCTAGTGCAGCAGGAGGTGATAGAAGCTTCTCATTTGAGGTGGGAGCTCCACCAAATGTTGCTGAGAAAGCCCATAGCCCTGTTTGGAGCCCTTTCCCTAGATACTTAACTTCTCAGAGTACCAATGTCTCTCTCTCTCTCTCTCTCTCTCTGCGTGTGCCCGCTCGTATGACATATATTAACATGGTTTTGTATCCAAATTTTAGACGGCCACAGAAAATCCTCAAGCTGGAAGTTCTTTAAAGGATGCTAGTGATGATAGTAAGAAAGCATCTACTGTGGAGGCCGGTAAAGAACAGCTATCAGAAAGGAAAGTGACTGAAAGTTCTGGGGGCAGCCCCCCAGACAACTCTAATATAGGCGGCAACATTAAGAGTAGAAGTTCACCGCCGCGGCAGCATCCAATCCCTGAGTGTTCTGGTATGCAAATCTCATATATCTTGACGATTCGAAGCTATTAAAAATTTAATTGACTTGAATATTTCAGTTTCTTCTGCAGATTTAGTGAATTTTCCATTCACAGATCCACAGCATTTACAGCTACGCGCACAGATATTTGTTTATGGAGCTCTTATGTGAGTTATAATTCTCTTAAAGTTACTCCTATCCCAAATATAATAAATCCATATAGCATTATCTCGCATGCACCAATTATAAAGCCATGACAAACTTCAATGCACATTTTACCTTCTTCCATTTCTGCCCCTTCTTAATTGCACCCTGACCAGTGCACTAAACTTGTCACCTATCTAACTCTAGCAGTAAGTGATGAGCATAGTGCAGTTGTACCCGTTACCTTAATTCTTGTGCCCAAGGCGAAACTTGATGATTTTTTGGGATTGGTTGGAGTATTTATTTGTAAAACGTGAGATCTAATATAAGTTTATTTTCTTTGAGCAGTCAAGGAACGCCACCAGGAGAGGCTTACATGGTGGCAGCTTTTGGAGAACCTGGTTTGTCTGGCTTCACCGTTTAATATAGTCCTCTTCCGCTCTATATGTATCTTATAGATTTTTTGTTACATGAACAGTTGGTAGTGGCAAACCTACATGGGAGGCTGCTTGGCGAGCTGCTTTTGAAAGATTTCAGTATCAAAAATCGCTTTATACCAGTTTAGAGACCCCCACAAGTGCACGTATAGGTAATTATATGATGATAGTGGAACAATATCTGCCATGCATTTTTTTTCTATTTTTTCTGACCAGCCGAGTTTGCAGGTAGTTCTGTGCCTGAGAAAGCTAGCAAGGGTACAGCAATTACAACTGTGCCAGCTAGCAAAAAAGGTGGCAAAACTGTGGTGCCAGCACATCCTGCTGTAACCCTACACTCCCCAACTTCTAATGTACCTTTTGGTAGTTCTACATTTAACCTGCAACGAGGTACTCATCTGGATTTTAGCCAGGCAGTATCACCATTTGCATATAATTCACACATGAGACATCCGTCTTCTGGTGTTGCCCCCTGGTATACTCAAAGCCCTGGTCCACGTCCTGCACCCTGGTTGATTCCACCACAAAACTTAATATTTGATTCATCGATGCAACCAGCTGTTCCTACAAATGAAACTGCGAAGGGGGCATCATCCAAAAATATATCCATTTCACATACTGTTTCATCTGGTGTAGTTCTTCCCAGTCCAGCACCTTCTATCGTTTCTTCTCCAACAGCAGTTGTGAATGATGGGAAACAGAAGGCTGCTTCTAGCTCTAAGCATGGCACCGCATCACAAAAGCCAAGAAAAAGAAAGAAAGCTTCAGCAAGTCCGGAACAGCAACCTGTCTTTGCTTCTCCTCAGCTCAAAATGGACATGCCATCTTTTACTCCTGCCATTAAGCACACAGCAGGATTTGCCTTATCCGGTAGCAAGCTTGTTCCTAACACTGGCCAGATTGCCTCTGAACCTAACTACCAGATCACTGGTGGTATGGATAGTGAGCAAAGAATCATCTTTTCAGAACAGATCCGTGGTGCAGTAGAACAATCAACTGCACAGGCTAAAGGTGCAAGCATTGACTCAATGGAGGCAGTTAAGCACAAGGAAGGCTTATGGAGCCATCTATCCACAATCTCAAGAGACAAGTTACCTCCAGAAGTTGAAGAGAAGCTTACCTCGGTTGCAGCTGCTGCTGAAGCAGCAGTTTCTGTTGCAAAGGCAGCTGCAGAAGCTGCCAAGATGGCATCAGAGGCTGCATTGCAGGCGAAAATGATGGCAGAGGAAGCTCTTAGCTCTTCTATATCTGTAATGCCCATGCATCACGAAGCTGGTCAAATTAATGTCATTAGCAGTCCACGCACCTTGTCAAGTTCAACACCAGCATCATCTCTGAAAATTAAGAACAAGAGTCATGCCCCAGGTTCCATCATTTCTGTGGCACGGGAGGCTGCTAGAAAGAGGGTTGAAGAGGCATCTGCAGCTGCAAAACGTGCTGAAAACTTGGATGCCATATTGAAAGCTGCAGAGCTTGCTGCGGAGGCTGTGTTCAGGGCAGGAACCATTATTGGAATGGGAGAACCACTACCTTTTACCCTAAGAGAGCTTTTGGAAGCTGGGCCGAATGGCTATTGGAAGTCAGCGAGTGTGAGGAATAAATCTGGAAGTGGTAATGATAATCCAGTAACAGAAACATTGGAGGTAGATGCACCTGTTAACCTTAACAAATCTGGCAGAAAGCGTGGTCGGAAACCTAAATATGATCAAGCACTACTGGGTTCGGAGCCATCTTCAAGTTGCAAAGAATTACAGCCATATGGAATACACTCAGGTAAAGTTACAATTAGCTTTTGGCATATCAAGACCATTGTTCAGTGTCAACTCATTTACCTTCTGTTTGGCTAATGTAGGACATGGGGTTGTTGAAGATGTTCCTGTCACTGTGTCGCTGGATGGTAACAATGTTATAGCGCCAATAAACATTATCTGGAATGGCATTGAAAAGGGATCTTCAGTTGAGGTAAGTTTCTTTGTTTGGCTTGGACACGGTTCTCCTTTCTCCCTTTCGACATGATGATCATAGAGTGGGTGTCTCTGGACTCTGGATTCTGGGCCTTTTGTTATTCCTTTTCTTATATTATGTACTCTAGGTATTTATTGGTTTCCTTGTTTCCTGATTGCTGCATTGTACATTAACATAATCTCCCCAATTACATTCACATCAATATAGTAAAAATCAGAAAATGCTAGTACCTCAACCTTTTCATGTTAATATGTTGGTAATGGTCATAAAAACATATTTGAGTCCGTATGACAAATCTATATTGGTTGATGTGCATCAGTCCAAAACTAATGAAAATTTTTCTGCTTACGTTTTAAGATGCTAATGATGTCCTTAAGTTTTTACATGCTCTGTTTCAGTATAAGTGTTTCTCAGCCTTGCTGTGATCTGATCTGATTTGCCACCTCAATTCTTAATGCAACCTTTTTACCGTGTTACTTCATTTTGTTTTTGATAGGTTTTATCTGACAAGGGTGGGTTTGGAGTAGCTTGGTTTTCTGCTAAGGTTATTGATATAAATGCAAATAATGCTTTTGTCAACTATGACAACCACAATGGTGAGTATCCATTCATTGACCCGCATATCAACCTTTTGTTGATGCTCCTTCCATGGCGTCGTTCCATTTTTGACCTTTTTCACAGCTACTTATTTAATTAAGCTTTTTTTTCAGGAACTGGTCCTCGTGAAGAATGGGTGCCATTGAGACAAGAGGGGGATAAGCCGCCACAGATACGTCTTGCAAACCCTGCTACCCTCTCTAAATTCAAAACTAGGAAACGACGCAGGGAAACAGCAGGCAGTTGTTTATGGGTTATTGGTGATCATGTAGACGCTTGGGTCAATAATAGGTATATGCTATTTGTAGAATATTGAGATTTTCTATTATTTTGGGCTAACCCTAGAAGGGTAGCTATATAGGTACCATACATGGGCCACATGGGCCTAGCCTCATGGGCCTAATACAACCACACTCCAACACTATTGCTTGATGCGAGTTCACTATTTGTTGTGGTATCAGTGTTCTTAGGTTTATTATCAACTTTATCTGATGCTTGTTTCTTTCAGCTGTTGGCGAGAGGGAGTTATTTCTCAGAATTATGAGACTGATGAGACGAAATATGTTGTACATTTTTCAGGTATTTTTTGCAGCCACTATCCCCTGTCATCTATTTTTCCTTTTTACCTCTTTGTGTCCTTTTACATGCATTCATTTGAACTTTTTTAGGAACTTAAGAGGCTAGAAACAATAGTTTGCCATTCATCAGTGCTGTACTAGTAGTCTGGTATTGATGGGAGAGCAGGCAGTGCCATTGAATTTTCAAAATTGCAGAATTAATATTCCTTGTACATCATACAGGATTAATATAACAAACTATTCTAGTAATTGTGGGTTTTATAAAACGAGAAGACATTATGAAATTCATGTTCATTCTCCACCCATGGCCGAAGCATTGTCGATCAGTATCCTGGTTGTTGGAATGTGTGTGTATTAGGCCCATGAGGCTAGGCCCATGTGGCCCATGTATGGTTACCTATATAGCTACCCTTCTAGGGTTAGCCCAAATCAATAGAAACACTTTCCCTAACATGGTATTAGCAGCCTAGGGTTAGCTTCCTCTCCTTCCACCTCTCCCCAGCCGTCGCCTCTGGTGGCCGCCGCCGCCGGCCAGCCGGCGCCATGGCTGGCGCTCCTCCTCCTTCCCTCTATAGTTACTTCCCCCCCTCTCCTCTTCTTCCACCTGGGCACAGGTTCTGGCGGCCGCCCCTGCTCCTCCAGGCGCCGCTCCTCCTGGCGGGGCCCTGCCAGCCGCCGCCTCCTACCCGGCTGCCCCTGGCGCGCGCGCGGCACTGCCAGCCGCCGGCGCCTCCCACCCGGCCGGCCCAGGCGCGGGCGCACCTCCCCCTGGGGCCGCCCATCTTCCTCCTGGCGCGGCTCTCACAGCCGGCGCCTCCTTCCCGGCCGGGCACGGTGCGGGCGCCCCTCCCCCTGGTGCCACTCCTGCTGGTGCGGGTGAACACGGGGATGGGGTGGCCGTCCTTCCCCTGCCCATGGATGCGACACCACTGGCGCCTGTTGTCCACCCAACGCCGCCTGGAGTCCGGCCGCCGCCCCCTGCCACCGACGTTGCGCCCATTGCAGCCCTGGGTGGGGTGGTCGGCGTTCCTTCTGCTGGCCCGCCAAAC

>4

ATATATATACCGTAAGATTTGATGCGATAGTTTTGGTTGAAATTTTTTTTTTTTTTTTGGTTATAAGGCCTCAGTCCTCCCAGGCCCAGCTCACGCGCGTGGGTGGGATTGGGATTGGGATTGGGATTGGGAACAACAGCTCGGCCCCGGCCCGGACGGACCCAGCTCAGCAACGGCTCGCCCTCCCACCAAACAAACCGAACCACCGCCCGCTGTTTGCTGTCCTTTCCTCAACGCCAGCCACGCCGAGAGAGACCAACCGCCGCCACCGGAATGGCGGGGTGACGAAGCCCACCGGACAAGACAGGGGAACTGCCGCCGCCGCGACCCACAGGACACCGGGTAGAGGAGGGGATCCAGCCAGCGAGCCATGGCGAGCGACGTGCCCATGAGCCCCGAGCTCGAGCAGGTGGACGGCGAGATCCAGGACATCTTCCGCGCGCTCCAGTGAGTCAGATCCCTCTCCCTAGTCCCTCCCTTCCCCCCTCTGCTTCCACCAATGCGCTTCTCGCCGCCCTCTCGGCTGGACTGGACTGGACTGGACTGCGGTGACGCTTCTCCCGCCCTCGCGGCGGCCGGCGCCCGGATTTCGCCGGCTCCTGCTCCAGTCGCAGCCAGTTCCTTCATATACAGCTACTACTCCCTACTGCTTCCTACGTTCTGCTCTGCTGCACACCAAGTCTTAATTCCTGCTTGCTCCAGGAATGGGTTCCAGAAGATGGACAAGATCAAGGACTCCAGCAGGCAGTCCAAGCAGCTGGAGGATCTCACCGCCAAGATGAGAGAGTGCAAGCGGTACGTCTGCCTCAACTCAGCCCCGTGTCTTTGAAGCTAACGCCTACCTCACGCAGATTCAGCTTTCCCCACCTATACTACACGTGGCATTGCTATTCCTGACTCGAATCAGAGACTTCAGATCTTCTTTTTCTACCCAACATGAAAGCGCCCTGAGACTCTTTACAGTTAGCTAAAATAAAATACGCTGCTATCTTTTGCAGCTTGATCAAAGAATTTGACCGTATACTCAAGGATGAGGAGAAAAAGAACACTCCTGATGTCAACAAGCAGCTCAATGACAAGAAGCAGTTCATGGTTGGTACTTGTTTTTCCTTCACATTTGGCTTTTGTTGTTTGTCACTTTTGGACACGGGTATAATTCTGTTTCTTGTGTGCTATTGCAGATCAAAGAACTCAACTCCTATGTAACCTTGAGGAAAACGTAAGTATAAGCCTTTCCTTTTTCTATTTATAATGCCCTCAGAGTTGAAGCATTCTACATTGAATCCCTTATTTGCAAATTTATGCCGGCAGCGTTGTTTGCTTTGTTGCAATACACTTCATGCTAAGATGTTGTATCCACTGCATCTTTTTTGGTGTTTCAGGTACCAGAGTAGCCTTGGTAATAAGAGGATCGAACTATTCGATACTGGTAATGACCAAGTGGCTGACGAAACTCCAGTTCAAATGGCATCAGGTGGTCATCAGTAGCTTTTAGTATTTCTAACAATATCCTCTTCAAACATCATAGGTGTTAACTTTCTCACTAATGTACCTGGATGGTTTTACAGAAATGTCAAATCAAGAACTCATTAGTGCTGGAAGGAAACAAATGGACCAAACTGACCAAGCCATTGAGCGCTCAAAAATGGTGTGCAACTAACATACACCCTTTTGTTTTCTTTTTTGAACCATCAAGCAGAATTCTGCTATTGGTTAAAGAGCACAAAATACTCATCTAGCCTCCATAAAAGAGAAGAAAGGAACAATAAACTTCAGCTCTTCCATAATGGATTTTAAAAGAACCATTTTCTTGTAGGTTGTAGCACAAACTGTTGAAGTTGGAGCTCAGACTGCTGCAACTCTGTCACAGCAAGTAAGTTTTCTTGAATGGCAATATAACCTGTTTGTTTACAACCAATTTTTTCCATAAAGTTTTTGCTAAGCTCTACTGGGGCTTACTGATCACAAAACTTCCATACTTAATTGGATAAGGCCATAAACTATTATAGCTTTTTTTTTGTCACAACCCTTCTTTCCCCTTATCTGCATCCATCTGACAATAGATTTGTATGCTTTATTTTACCTGTAAATGGTTCACTGCTCTGTCCCACTGAGTGTCAGTCACTCAGTCTTGAATATATTTGAGAATGGACAAAACCCAGAGGGCTTCAATTAGATCCACAATAATTGTCTCACACTACTATTTGACATAATGCTAATCAAAGTTTGGCTGTCTATTTACTTGAGACACTGCTGTAGTATTCTAAAAATGATAGTTCAACCACAATCTGTGCACTACACAACTCGATTGAAGGACTAAAAAAATCCTGCTACATTTTCCATTTGAAGTATGTTCACAAAGATTTCTTTAGACCAAAGAACAAAGTACCTTGAGATTCCATTTTCCTTAGGAATAAATATCAATCAATCTTGACACTTTCCAGAGCAAATGTAGCTTCTCCTTTTATATTAGTATGCTTCAGGAACTGGATATGTTTATAAAGGCAGAGTAGATGTTCTATATGCAATAAACAGTTATTAATATTTAGAATCTGATTTCTTGATCTGTTACATGTACCTGTATAAGTTGTTTCCCAAGGTCAACATGACAACGTCCATACGCTCGTGTGGTTGTTTTCTGAATATCATTTCGTCATTTTTTCAGACAGAACAAATGAAGAGAATAGGCAATGAGCTAGATTCTGTCCACTTTTCACTGAAGAAGGCTAGTCAAATGGTGAAAGAGATTGGCCGTCAGGTTCTCCCTCGCTCTCTAGCACTCAAATGCTTGTTTTTTTGTTACTCTTATGTGGCTGAATTTACAAGATTTTCCATTTCAGGTTGCTACTGACAAATGCATTATGGCGTTCCTGTTTCTGATTGTCTGTGGTGTTATTGCAATAATTGTTGTTAAGGTATGTTGCTGACTCTGTGAAGGTAGCATTGTATTTCTTTTGTGCCTAAATAGGATTAGCTATGCTTGGTGTTCAGTTTAGGAACAGAATCTCCACATGTAAGAGTAGAACTAGTACCAGTAGCCAACACCTTTGGGAGTGAAAATCCATGTGGTTGGGTCAGTTGTGGCCCCTATCTAACTTTCCCATAGGATCTGTTGGAATTAAATATCAAAGACAATTCGAGAAAACTTCGTTGGATAATTTACAGAAAGTTTTCCTTAAATTATTATAGGAACTAAAGTTGGGCAACTTTACCTTAGTTCTTTAGGGTCTAGTGCTAACAACTTGAACAGACATAGTGATGATTGAGAATGATGATGCATGTCAGGCCTTAGTGCTACTGATTTTGTGTTTGTTTTGAAACCCTATCTATTCTTTATCTCCCCACATAAGCCATTATCATTTTAGTTCCTAATGAGAATCTTAGACCGTCTGCTGGTTTCCTACACTGTACAATCAATAACCAATTACCCTTCTTTCTCACAGATTGTCAACCCACATAACAAGAACATCAGAGATATTCCGGGGCTGGCACCTCCCGCCATGAACAGGAAGCTGCTGTCCATCGATGCTTTTGGAGGGCTCTGAGCGCTGTGAATGGCTGCCCCCTGCAGTACAGCGCAGCACATCAGAGTTCTTTTATGAGCCACGATACATGATTTCGCGATTCTTTTGTGCTTGTGTATTGTCTCATGTTGTTTACGTCCGCCCATTCTTTCCCTATTGCATTGGTGAGTAGTAGCTTCAGGTTTCTAGCGGCACTCTTGTTGTTTATTGCTTGCGAGCACTGGCTTTCTGGTTGTCTGTGTATACGCGGTAGTGATGTGAATGTGAGAAATGAGATTCATCATGGTTCATTCTTGTGAGTTGTGACTGGTTTTGTGATCTTGCGGTGAAGGTGGCCAGTTTGGCATGCTTTTCGTGTGCTTCTGTACTCGAAGGTCACCTCGGTCCGAATGCGCCACCATGCCCAGCAGACCAGGTGGATTGGGATAGGCGGATAGGAAACTGGTGCGTCGTACGCCGCGTGCAGGGAAGAGGAAACTGGTAGGTTGCTTTACGAATTCCGATTGCATCATCGTTATGGCAACGAACCCAGCCACAGCCATCAGCCAGGACCAACGTGATGCTCAATTAAACACTAGTAGTAGCAAAAGGCGCCCGGGCTACAGCAAGCTCTCTTGCGCACTCCCAAACCCTCGCAGATCCACCTACAGAGGAGGCATTGAAGGGAGCTCGTCATCCTCGATCATCAAGACATCAGCAAAGAAGGGATCGCCCAGACAATCTCCAGCGGACTCGTAAGACGCCAGGCGTTTCTTGAACGAGCGAAGCTCCCGCTCGATTTTGTTGCCAGCAGCCTAGCCAAAAGTGTCCACAGGGATAGAATTTAGAAACGCAGACGCTGGTAGATTCCTTGGTTCACAGGTAATGTCAGTAGGGACTGAAAAGCGGAGGGGAATGATGAACTAACTCACAAGTGGTTTCAGCTTCTGCAGCACCATATCTACAAGGTAGATTAACCACACCACGTTTGTCTTCGGGAAGCTGCAGTGCAAGTACAGATTCAGGACCAAATCTGGCGTCAAGTAACTTTTTCAAGGGAACTTCAGCATCGCATGTGCAACAGAAGAGGACTCTGATTACTGTTTACCTGCCCTCCCAGTGCTCCTGGGTAATCTGTTTCATTCTTCGATATGTTTCTGCCTGCAAACAACGAGATGCAACGCGTTGGGTAAAGCACGTAACAATGTTTTAACATCGTACTCTCAGGCAGATCAGAAGAAAACCTGCTTGTCTCCCTTTTTCCCTTGAAATAGTGCAGGGTCCGCAGACAGGTCAAGGAAAAGAATGGCAGTCCCTAGAACAAAATAACAAGGAATATAAGAGAGGCAAAAAAAAAAGTTACAGCATTACATGTCCTATTCTTTAATCTTTAAAAGTTTAAATGATCTACAATCACTTTTGTGCTAGGAGCAAGAAGTTTCAAAATGTTGTTGCTGATTGCGTGGCATCAACTGTTTCAGGTGTAAACCTCAATAAATATTTCATCTGTTTCAGGTGTAAACCTCATCGCCTGCTATCATATTCCACTGCTCCTCAACTCTGTTCATATATTCAACTTTTCAAGCTGACAAAAGCTGCAGTAAAGTAAGAGCCAAAAATGGCAAGTATTATTTTATTAAGCATGCATTGTCTTGTTCTTATCTACGATATGAAGCATTATCTATTCTCCTTTCACCATGAAGGCCTACAAGTTGGCATACTTGTAAGTTGGCCTGTGGCTAGATGGCTAGGCTAGGCAGTGGCCGAGCTTAAGCACAATCTTAAGGGGGCCACTAATGATGCATGGATCAAATTAGCCACGGATTACTATATGGTCTAAGACAATTTAAGAAGCTAGGGGGAATGGCCCAGGTTGATCTTACCTAACCTCTGCCCCTGTGGTTAGGACTATCAGTGTGCATCCACCCCACCAAAGTTCAAATCCTAAATTCTGAGTCAAACGGTAAATTTCTAGTGTGTAGAGGACCAACAGCACCAGCACGTTTGTGCTGAGTGTGTCTACCTTATACTTTCTAAAAGAGTCAATAAACTTGGACAAGTTAGAAGATTGCCACATCGAACTTTAAGGGATTCCTCTAATCCAGTAAGTATTTGTAATTCCCTTCTATCAGTTGCAGCAACTTCTTATTTACGCATGGCAAAAAATTGTTGTACAATTGTCACAAATGATGAAAAACTTCAAAGACTAAAGGAATATTGCAAACTACAGAAACAACGGTAAATGCAATGCAAAAATGAAGAACTTAGTCTGTTAATAAGTTCAGATAATTCTGGGGGCACCTGTGTTGATCCGAGAAAGAGTGAAGTCAATTATGGAAATATTCAGACCAAAAGTTCTTGCATGCATCCTCTTCCCTTGAAGAGTGAAGCTCATTGTATGATTTGTGTCTGGAGTTTCATCTTGAGCCAAAAGAATGTTACCCCTGCCATGCCACGCACATAGTGGTGTTAACTAAAATATGAAATATAGCCTTAGGTTGTGAAAGCTAAAGCACTTAGGTATGAAATATATTTCCATTATTTTTTCATACGACACTGTTTTCACTATACAAAATGACATTGGCTCTGAGATTTTAATTTAAAGACATGAATCACCAAAGCTGTGTTGAGAGAACAGACCAGTGCAAATCTCGATGTTCAAATTCACAAGCACTTTCAGCTACTGCTAAGGAAGCAGTAACCTGCATCAATAGAATGAAATATAGCTGTCCTTAGAACTTTGTAGTTGAGGTCACACATGTATAATATAATATATTATAAAATTCATTGTGCGGACCTGAACCAGTAAACTGTGAGCCTCTTTATAGTCAACTAGAGCGAAACTTTCAAGATCAGTGCCACCATCAGCTAGCACAAAGACAATGTAGCACTGAAAACAAAGATTACTTGTGTAAGAGGTAAATTAAGAGATATGTTCTGTTTCCCATGTTCCACGACAATGACATGATATTTTGTTTCCCTAAGATGAGAACTTACCTGTTCCCTTGTAAACTCCTTTGGATGGTCATTTTCAGATCCACGTTCCGCATCATAGTCTTCCCAGGCACGAATCAGAGAAGGGTCATAGGGTCCTTGACAGACCCGAAAGCTGCAAGATATATGAACTTGAGAGGACTCAAACCAAGGTATTTAGTGTTATTGAAGTACCTTTGTACAGACAAAATCAAAATTATTCGTAGCCAGGCTTTAATCATTTAGTACAATTTCTAGATAAATAGTGCGGTAGAATTTTGAAAGAATATGTTGGAAACGGGTCTCACTGCGCAGGTGAGCAGCAGCAGCTCACTCACCCTCTCACAACTCACGGTTAGAGGTAGAAGATGAACTTGAGAACAGAGAGCACACACTCACAGCCTGCGCACACGTCTGCTCGTGCAGCAAACCGAACTGCTTTTTATTGCTAAACAAGTTGTTATATATACAACTCTAGTCAACTATAACTTGCTAGTACTATAGCCACTACTAGCTACAGCTGGCCACTAGTGGGCTGAGATCAGCCACTACAGACTGTTGCCATCCTGTTACAGTAGATAGAGATTAGAGAAGACAGCACGGTTGACTTATTGCCAACTTTTCTACTGCTGCAAAGGTGGAGTGCAGCAGGCCTTTTTCCTGCTGCTGCAAAGTGTGGAGAGTGCAGCAGATTATCTCACAGAATACAGGTTCTTCATACAAGCTATAAATAGTCAAAGTTTTATGTATTTTCCCACTGCAAAGATAATGAAAGGCACATACTCTTTTGTCTCAATGAAGCCATTGCATGAATTTGTTTTAACATCATCACCCTGATTTGCTCTCAGATTATTAAGCGTTAGAGAAAGTAAGACTTCCTCAAGTATTTCTTCCGATCTCTACAAAACAGAACCACCTCATGAGTACCAAACAAATATAGACCAGATAACACTAATTAATGAAGTGAACCTTTTGGGTTTCTCCATTGACTAACAAGTCTCCGTCAAAAGGTACCACTTTGCACACCGTTCTTCCAGCCCTGTAGGCCTCCCCATAGGTCCCTTCACCAAGCTTCGTGATGCTGCCTAGCTCACTATCCATGAACAATATCATTAACTAACAGTAAACCACAAAAGCATCCAGAAAACAAAGGAATAATATTTATATTGAAAGGTTGAACTCCTACCAATAAGCAGAGAATACTTCTGCCAGGGTAACTGGTGCAGACTGCCTGCAAACCATGAGGAGCTGGTCAAAAGCACTCAGAGCTTCACCATTCCATGGAATGCTAGCTTCAACAGGCTCCTCCTTAATGTTCACTTTACCGAATGATGTAATCAGGCTGTCTTTTGCACTGCTCTTTAAACTGATATCAGTGTAGCCGACAGGGATTGCCCTTCCAGCGGTGTATGTCCCTGACCCTCTGTTAGTTGTACTGTTGTTACTGAGGACTGATGGTAAGATTGGCGTCTCCATAATGTCAAACAAGGGCTCCGATGACGCACGTCTTGCAAGACTGGAAATTTTCCACCTCTCCAAAATTGCAGCCAGATCATGAATTATGATATTCTCCTCCATCCCTTTGTTCCACGAGGCAAAGTACTTAGGAGAAGGACTTTCTTCCATGAGATCAAAAGCATCGACCTCTTCAAAATATGCCTTCTCCTTGCTAAAATCGGGAGGGCCACCCTGATGTTTCTTCTGTAAACAACATGGCAAGCTTAACAAGAAACTAATTCAACAACATCAGAAAATATGCGTGTAGAAATGGCAGACCCACCACTCGGTTGCTAGGTTTGGGTGGTCGTTTCAGTGCTCCACAGGGTTTCTGTTGAAGACTTGCACCGGGTGCGACAAAAATACTCTCTCTCCCTCTGTATTCAAAACATTTGTTAGTTAAAAACTTACTTGAGATTCATATAATGCCGAAATTTGCTAACAAGGTGAAAAAGTTTGTTTAGAGGTTGGCTCATAACAACCTGCCAGTACATCGTAACATAGCAAGAAGAGGGATTAAATTAGGTCCGTGTGCCCATTGTGCATTCATATGGATGAAGACTGGCCATCTTTTTTTTTTCAAGTGCAGACTTGTCAAGGAACGCTGGTGAACAATGAAATGTGAAAAAGAGAGAACTATGCTGGAAGGCTGCAATTCAGGTATTGAAACTATCAGTTTGGTCCCCGATGTGGAACAGCAGGTGCAATAGAAGATTTGATGTCCCCTGAGTTTGTGAGTCTCGGTTTGCAATAGAATGTGTAATCAGGTATCGGATTGTTTAACTACTTATGGTGCAGGTATGTATGCCTCTAATCCTCATATGTTGGACCATCCGCCAGACTTTGTAAAGGAATTAGTCTCTAGTGACATTCCCGGAGACAATGACTAATGGAACCGTTTGCAAAGTGGAAGTAAAGCTGCGCGTCACATGAACAAATTTGCTACTGAAGAGAAATGGAAGCTAGGCGCACCTGATGGAGAGCGAGCGGTTCCAGCTGGTCCGCTTTCGCGGCACGAAGCTCGGCCGCCGCAGGCCGCTGAAGCTGGCCCTCCTGGGGGAACAGGAAAACGCGTTACCCAATCACGGCAGAGGCAGCCCTAAATCCACAATACCAGTGACATGCCGGCGAACGAACGAACGAACGATTGGCAGCGGCCAGGAGCGCTCTTACGTCTCCACGTTCGTCCTGCCGGACGACTCCTCGACCAGGCGACGACCGTACACGACGCCGACGTGGGGCGGCCGGGCCCGGCCGCCCCACGTCGGCGTCGTGTACGGTCGTCGCCTGGTCGAGGAGTCGTCCGGCAGGACGAACGTGGAGACGTAAGAGCGCTCCTGGCCGCTGCCAATCGTTCGTTCGTTCGTTCGCCGGCATGTCACTGGTATTGTGGATTTAGGGCTGCCTCTGCCGTGATTGGGTAACGCGTTTTCCTGTTCCCCCAGGAGGGCCAGCTTCAGCGGCCTGCGGCGGCCGAGCTTCGTGCCGCGAAAGCGGACCAGCTGGAACCGCTCGCTCTCCATCAGGTGCGCCTAGCTTCCATTTCTCTTCAGTAGCAAATTTGTTCATGTGACGCGCAGCTTTACTTCCACTTTGCAAACGGTTCCATTAGTCATTGTCTCCGGGAATGTCACTAGAGACTAATTCCTTTACAAAGTCTGGCGGATGGTCCAACATATGAGGATTAGAGGCATACATACCTGCACCATAAGTAGTTAAACAATCCGATACCTGATTACACATTCTATTGCAAACCGAGACTCACAAACTCAGGGGACATCAAATCTTCTATTGCACCTGCTGTTCCACATCGGGGACCAAACTGATAGTTTCAATACCTGAATTGCAGCCTTCCAGCATAGTTCTCTCTTTTTCACATTTCATTGTTCACCAGCGTTCCTTGACAAGTCTGCACTTGAAAAAAAAAAGATGGCCAGTCTTCATCCATATGAATGCACAATGGGCACACGGACCTAATTTAATCCCTCTTCTTGCTATGTTACGATGTACTGGCAGGTTGTTATGAGCCAACCTCTAAACAAACTTTTTCACCTTGTTAGCAAATTTCGGCATTATATGAATCTCAAGTAAGTTTTTAACTAACAAATGTTTTGAATACAGAGGGAGAGAGAGTATTTTTGTCGCACCCGGTGCAAGTCTTCAACAGAAACCCTGTGGAGCACTGAAACGACCACCCAAACCTAGCAACCGAGTGGTGGGTCTGCCATTTCTACACGCATATTTTCTGATGTTGTTGAATTAGTTTCTTGTTAAGCTTGCCATGTTGTTTACAGAAGAAACATCAGGGTGGCCCTCCCGATTTTAGCAAGGAGAAGGCATATTTTGAAGAGGTCGATGCTTTTGATCTCATGGAAGAAAGTCCTTCTCCTAAGTACTTTGCCTCGTGGAACAAAGGGATGGAGGAGAATATCATAATTCATGATCTGGCTGCAATTTTGGAGAGGTGGAAAATTTCCAGTCTTGCAAGACGTGCGTCATCGGAGCCCTTGTTTGACATTATGGAGACGCCAATCTTACCATCAGTCCTCAGTAACAACAGTACAACTAACAGAGGGTCAGGGACATACACCGCTGGAAGGGCAATCCCTGTCGGCTACACTGATATCAGTTTAAAGAGCAGTGCAAAAGACAGCCTGATTACATCATTCGGTAAAGTGAACATTAAGGAGGAGCCTGTTGAAGCTAGCATTCCATGGAATGGTGAAGCTCTGAGTGCTTTTGACCAGCTCCTCATGGTTTGCAGGCAGTCTGCACCAGTTACCCTGGCAGAAGTATTCTCTGCTTATTGGTAGGAGTTCAACCTTTCAATATAAATATTATTCCTTTGTTTTCTGGATGCTTTTGTGGTTTACTGTTAGTTAATGATATTGTTCATGGATAGTGAGCTAGGCAGCATCACGAAGCTTGGTGAAGGGACCTATGGGGAGGCCTACAGGGCTGGAAGAACGGTGTGCAAAGTGGTACCTTTTGACGGAGACTTGTTAGTCAATGGAGAAACCCAAAAGGTTCACTTCATTAATTAGTGTTATCTGGTCTATATTTGTTTGGTACTCATGAGGTGGTTCTGTTTTGTAGAGATCGGAAGAAATACTTGAGGAAGTCTTACTTTCTCTAACGCTTAATAATCTGAGAGCAAATCAGGGTGATGATGTTAAAACAAATTCATGCAATGGCTTCATTGAGACAAAAGAGTATGTGCCTTTCATTATCTTTGCAGTGGGAAAATACATAAAACTTTGACTATTTATAGCTTGTATGAAGAACCTGTATTCTGTGAGATAATCTGCTGCACTCTCCACACTTTGCAGCAGCAGGAAAAAGGCCTGCTGCACTCCACCTTTGCAGCAGTAGAAAAGTTGGCAATAAGTCAACCGTGCTGTCTTCTCTAATCTCTATCTACTGTAACAGGATGGCAACAGTCTGTAGTGGCTGATCTCAGCCCACTAGTGGCCAGCTGTAGCTAGTAGTGGCTATAGTACTAGCAAGTTATAGTTGACTAGAGTTGTATATATAACAACTTGTTTAGCAATAAAAAGCAGTTCGGTTTGCTGCACGAGCAGACGTGTGCGCAGGCTGTGAGTGTGTGCTCTCTGTTCTCAAGTTCATCTTCTACCTCTAACCGTGAGTTGTGAGAGGGTGAGTGAGCTGCTGCTGCTCACCTGCGCAGTGAGACCCGTTTCCAACATATTCTTTCAAAATTCTACCGCACTATTTATCTAGAAATTGTACTAAATGATTAAAGCCTGGCTACGAATAATTTTGATTTTGTCTGTACAAAGGTACTTCAATAACACTAAATACCTTGGTTTGAGTCCTCTCAAGTTCATATATCTTGCAGCTTTCGGGTCTGTCAAGGACCCTATGACCCTTCTCTGATTCGTGCCTGGGAAGACTATGATGCGGAACGTGGATCTGAAAATGACCATCCAAAGGAGTTTACAAGGGAACAGGTAAGTTCTCATCTTAGGGAAACAAAATATCATGTCATTGTCGTGGAACATGGGAAACAGAACATATCTCTTAATTTACCTCTTACACAAGTAATCTTTGTTTTCAGTGCTACATTGTCTTTGTGCTAGCTGATGGTGGCACTGATCTTGAAAGTTTCGCTCTAGTTGACTATAAAGAGGCTCACAGTTTACTGGTTCAGGTCCGCACAATGAATTTTATAATATATTATATTATACATGTGTGACCTCAACTACAAAGTTCTAAGGACAGCTATATTTCATTCTATTGATGCAGGTTACTGCTTCCTTAGCAGTAGCTGAAAGTGCTTGTGAATTTGAACATCGAGATTTGCACTGGTCTGTTCTCTCAACACAGCTTTGGTGATTCATGTCTTTAAATTAAAATCTCAGAGCCAATGTCATTTTGTATAGTGAAAACAGTGTCGTATGAAAAAATAATGGAAATATATTTCATACCTAAGTGCTTTAGCTTTCACAACCTAAGGCTATATTTCATATTTTAGTTAACACCACTATGTGCGTGGCATGGCAGGGGTAACATTCTTTTGGCTCAAGATGAAACTCCAGACACAAATCATACAATGAGCTTCACTCTTCAAGGGAAGAGGATGCATGCAAGAACTTTTGGTCTGAATATTTCCATAATTGACTTCACTCTTTCTCGGATCAACACAGGTGCCCCCAGAATTATCTGAACTTATTAACAGACTAAGTTCTTCATTTTTGCATTGCATTTACCGTTGTTTCTGTAGTTTGCAATATTCCTTTAGTCTTTGAAGTTTTTCATCATTTGTGACAATTGTACAACAATTTTTTGCCATGCGTAAATAAGAAGTTGCTGCAACTGATAGAAGGGAATTACAAATACTTACTGGATTAGAGGAATCCCTTAAAGTTCGATGTGGCAATCTTCTAACTTGTCCAAGTTTATTGACTCTTTTAGAAAGTATAAGGTAGACACACTCAGCACAAACGTGCTGGTGCTGTTGGTCCTCTACACACTAGAAATTTACCGTTTGACTCAGAATTTAGGATTTGAACTTTGGTGGGGTGGATGCACACTGATAGTCCTAACCACAGGGGCAGAGGTTAGGTAAGATCAACCTGGGCCATTCCCCCTAGCTTCTTAAATTGTCTTAGACCATATAGTAATCCGTGGCTAATTTGATCCATGCATCATTAGTGGCCCCCTTAAGATTGTGCTTAAGCTCGGCCACTGCCTAGCCTAGCCATCTAGCCACAGGCCAACTTACAAGTATGCCAACTTGTAGGCCTTCATGGTGAAAGGAGAATAGATAATGCTTCATATCGTAGATAAGAACAAGACAATGCATGCTTAATAAAATAATACTTGCCATTTTTGGCTCTTACTTTACTGCAGCTTTTGTCAGCTTGAAAAGTTGAATATATGAACAGAGTTGAGGAGCAGTGGAATATGATAGCAGGCGATGAGGTTTACACCTGAAACAGATGAAATATTTATTGAGGTTTACACCTGAAACAGTTGATGCCACGCAATCAGCAACAACATTTTGAAACTTCTTGCTCCTAGCACAAAAGTGATTGTAGATCATTTAAACTTTTAAAGATTAAAGAATAGGACATGTAATGCTGTAACTTTTTTTTTTGCCTCTCTTATATTCCTTGTTATTTTGTTCTAGGGACTGCCATTCTTTTCCTTGACCTGTCTGCGGACCCTGCACTATTTCAAGGGAAAAAGGGAGACAAGCAGGTTTTCTTCTGATCTGCCTGAGAGTACGATGTTAAAACATTGTTACGTGCTTTACCCAACGCGTTGCATCTCGTTGTTTGCAGGCAGAAACATATCGAAGAATGAAACAGATTACCCAGGAGCACTGGGAGGGCAGGTAAACAGTAATCAGAGTCCTCTTCTGTTGCACATGCGATGCTGAAGTTCCCTTGAAAAAGTTACTTGACGCCAGATTTGGTCCTGAATCTGTACTTGCACTGCAGCTTCCCGAAGACAAACGTGGTGTGGTTAATCTACCTTGTAGATATGGTGCTGCAGAAGCTGAAACCACTTGTGAGTTAGTTCATCATTCCCCTCCGCTTTTCAGTCCCTACTGACATTACCTGTGAACCAAGGAATCTACCAGCGTCTGCGTTTCTAAATTCTATCCCTGTGGACACTTTTGGCTAGGCTGCTGGCAACAAAATCGAGCGGGAGCTTCGCTCGTTCAAGAAACGCCTGGCGTCTTACGAGTCCGCTGGAGATTGTCTGGGCGATCCCTTCTTTGCTGATGTCTTGATGATCGAGGATGACGAGCTCCCTTCAATGCCTCCTCTGTAGGTGGATCTGCGAGGGTTTGGGAGTGCGCAAGAGAGCTTGCTGTAGCCCGGGCGCCTTTTGCTACTACTAGTGTTTAATTGAGCATCACGTTGGTCCTGGCTGATGGCTGTGGCTGGGTTCGTTGCCATAACGATGATGCAATCGGAATTCGTAAAGCAACCTACCAGTTTCCTCTTCCCTGCACGCGGCGTACGACGCACCAGTTTCCTATCCGCCTATCCCAATCCACCTGGTCTGCTGGGCATGGTGGCGCATTCGGACCGAGGTGACCTTCGAGTACAGAAGCACACGAAAAGCATGCCAAACTGGCCACCTTCACCGCAAGATCACAAAACCAGTCACAACTCACAAGAATGAACCATGATGAATCTCATTTCTCACATTCACATCACTACCGCGTATACACAGACAACCAGAAAGCCAGTGCTCGCAAGCAATAAACAACAAGAGTGCCGCTAGAAACCTGAAGCTACTACTCACCAATGCAATAGGGAAAGAATGGGCGGACGTAAACAACATGAGACAATACACAAGCACAAAAGAATCGCGAAATCATGTATCGTGGCTCATAAAAGAACTCTGATGTGCTGCGCTGTACTGCAGGGGGCAGCCATTCACAGCGCTCAGAGCCCTCCAAAAGCATCGATGGACAGCAGCTTCCTGTTCATGGCGGGAGGTGCCAGCCCCGGAATATCTCTGATGTTCTTGTTATGTGGGTTGACAATCTGTGAGAAAGAAGGGTAATTGGTTATTGATTGTACAGTGTAGGAAACCAGCAGACGGTCTAAGATTCTCATTAGGAACTAAAATGATAATGGCTTATGTGGGGAGATAAAGAATAGATAGGGTTTCAAAACAAACACAAAATCAGTAGCACTAAGGCCTGACATGCATCATCATTCTCAATCATCACTATGTCTGTTCAAGTTGTTAGCACTAGACCCTAAAGAACTAAGGTAAAGTTGCCCAACTTTAGTTCCTATAATAATTTAAGGAAAACTTTCTGTAAATTATCCAACGAAGTTTTCTCGAATTGTCTTTGATATTTAATTCCAACAGATCCTATGGGAAAGTTAGATAGGGGCCACAACTGACCCAACCACATGGATTTTCACTCCCAAAGGTGTTGGCTACTGGTACTAGTTCTACTCTTACATGTGGAGATTCTGTTCCTAAACTGAACACCAAGCATAGCTAATCCTATTTAGGCACAAAAGAAATACAATGCTACCTTCACAGAGTCAGCAACATACCTTAACAACAATTATTGCAATAACACCACAGACAATCAGAAACAGGAACGCCATAATGCATTTGTCAGTAGCAACCTGAAATGGAAAATCTTGTAAATTCAGCCACATAAGAGTAACAAAAAAACAAGCATTTGAGTGCTAGAGAGCGAGGGAGAACCTGACGGCCAATCTCTTTCACCATTTGACTAGCCTTCTTCAGTGAAAAGTGGACAGAATCTAGCTCATTGCCTATTCTCTTCATTTGTTCTGTCTGAAAAAATGACGAAATGATATTCAGAAAACAACCACACGAGCGTATGGACGTTGTCATGTTGACCTTGGGAAACAACTTATACAGGTACATGTAACAGATCAAGAAATCAGATTCTAAATATTAATAACTGTTTATTGCATATAGAACATCTACTCTGCCTTTATAAACATATCCAGTTCCTGAAGCATACTAATATAAAAGGAGAAGCTACATTTGCTCTGGAAAGTGTCAAGATTGATTGATATTTATTCCTAAGGAAAATGGAATCTCAAGGTACTTTGTTCTTTGGTCTAAAGAAATCTTTGTGAACATACTTCAAATGGAAAATGTAGCAGGATTTTTTTAGTCCTTCAATCGAGTTGTGTAGTGCACAGATTGTGGTTGAACTATCATTTTTAGAATACTACAGCAGTGTCTCAAGTAAATAGACAGCCAAACTTTGATTAGCATTATGTCAAATAGTAGTGTGAGACAATTATTGTGGATCTAATTGAAGCCCTCTGGGTTTTGTCCATTCTCAAATATATTCAAGACTGAGTGACTGACACTCAGTGGGACAGAGCAGTGAACCATTTACAGGTAAAATAAAGCATACAAATCTATTGTCAGATGGATGCAGATAAGGGGAAAGAAGGGTTGTGACAAAAAAAAAGCTATAATAGTTTATGGCCTTATCCAATTAAGTATGGAAGTTTTGTGATCAGTAAGCCCCAGTAGAGCTTAGCAAAAACTTTATGGAAAAAATTGGTTGTAAACAAACAGGTTATATTGCCATTCAAGAAAACTTACTTGCTGTGACAGAGTTGCAGCAGTCTGAGCTCCAACTTCAACAGTTTGTGCTACAACCTACAAGAAAATGGTTCTTTTAAAATCCATTATGGAAGAGCTGAAGTTTATTGTTCCTTTCTTCTCTTTTATGGAGGCTAGATGAGTATTTTGTGCTCTTTAACCAATAGCAGAATTCTGCTTGATGGTTCAAAAAAGAAAACAAAAGGGTGTATGTTAGTTGCACACCATTTTTGAGCGCTCAATGGCTTGGTCAGTTTGGTCCATTTGTTTCCTTCCAGCACTAATGAGTTCTTGATTTGACATTTCTGTAAAACCATCCAGGTACATTAGTGAGAAAGTTAACACCTATGATGTTTGAAGAGGATATTGTTAGAAATACTAAAAGCTACTGATGACCACCTGATGCCATTTGAACTGGAGTTTCGTCAGCCACTTGGTCATTACCAGTATCGAATAGTTCGATCCTCTTATTACCAAGGCTACTCTGGTACCTGAAACACCAAAAAAGATGCAGTGGATACAACATCTTAGCATGAAGTGTATTGCAACAAAGCAAACAACGCTGCCGGCATAAATTTGCAAATAAGGGATTCAATGTAGAATGCTTCAACTCTGAGGGCATTATAAATAGAAAAAGGAAAGGCTTATACTTACGTTTTCCTCAAGGTTACATAGGAGTTGAGTTCTTTGATCTGCAATAGCACACAAGAAACAGAATTATACCCGTGTCCAAAAGTGACAAACAACAAAAGCCAAATGTGAAGGAAAAACAAGTACCAACCATGAACTGCTTCTTGTCATTGAGCTGCTTGTTGACATCAGGAGTGTTCTTTTTCTCCTCATCCTTGAGTATACGGTCAAATTCTTTGATCAAGCTGCAAAAGATAGCAGCGTATTTTATTTTAGCTAACTGTAAAGAGTCTCAGGGCGCTTTCATGTTGGGTAGAAAAAGAAGATCTGAAGTCTCTGATTCGAGTCAGGAATAGCAATGCCACGTGTAGTATAGGTGGGGAAAGCTGAATCTGCGTGAGGTAGGCGTTAGCTTCAAAGACACGGGGCTGAGTTGAGGCAGACGTACCGCTTGCACTCTCTCATCTTGGCGGTGAGATCCTCCAGCTGCTTGGACTGCCTGCTGGAGTCCTTGATCTTGTCCATCTTCTGGAACCCATTCCTGGAGCAAGCAGGAATTAAGACTTGGTGTGCAGCAGAGCAGAACGTAGGAAGCAGTAGGGAGTAGTAGCTGTATATGAAGGAACTGGCTGCGACTGGAGCAGGAGCCGGCGAAATCCGGGCGCCGGCCGCCGCGAGGGCGGGAGAAGCGTCACCGCAGTCCAGTCCAGTCCAGTCCAGCCGAGAGGGCGGCGAGAAGCGCATTGGTGGAAGCAGAGGGGGGAAGGGAGGGACTAGGGAGAGGGATCTGACTCACTGGAGCGCGCGGAAGATGTCCTGGATCTCGCCGTCCACCTGCTCGAGCTCGGGGCTCATGGGCACGTCGCTCGCCATGGCTCGCTGGCTGGATCCCCTCCTCTACCCGGTGTCCTGTGGGTCGCGGCGGCGGCAGTTCCCCTGTCTTGTCCGGTGGGCTTCGTCACCCCGCCATTCCGGTGGCGGCGGTTGGTCTCTCTCGGCGTGGCTGGCGTTGAGGAAAGGACAGCAAACAGCGGGCGGTGGTTCGGTTTGTTTGGTGGGAGGGCGAGCCGTTGCTGAGCTGGGTCCGTCCGGGCCGGGGCCGAGCTGTTGTTCCCAATCCCAATCCCAATCCCAATCCCACCCACGCGCGTGAGCTGGGCCTGGGAGGACTGAGGCCTTATAACCAAAAAAAAAAAAAAAATTTCAACCAAAACTATCGCATCAAATCTTACGGTATATATAT

>14

GGTCGGGGTAGGGGTGGCATCTCAGGGACTGTTAGCATCGTGGGTGCCACGACGTGCATTGGTGTGCCTGACGGCGTTCGTAATTTCTGGAGGGCTGTGGTCCAGACGGACGGTTCACGGCTCCACGCTTTCCGCTCGCCACTCGGAGAGAGCGGGGGCCTATTGCCGGGTTTGGCGCGCCGATGTGGCGATGTGGGTAGGAGGGCTTGGGCGCTTGGTCCATCTATATGTTTTTTTTCTCTCCGATGGCAGGTCCATCTTATTCTCAAAAAAAAAAATCTATGTTGCTCAAGTGCAATGCTACCATTTCAAAAAAAAGTTCGAGAATTTTCATTGCATTAAATTCCAATTTGAGGTCATTCCATGTGATTTGATATATATGATTTACAGAGGCAAAAGCAATGTGTATGGACCATACGATAGACCCCGTGTCAAATGGCTACGAACCTATATATACATATATAGGCCATGTTCATCCGGCTTATAATCCATACTTTTCAGCTAGCCAGAACAGTGTTTTTCTCTCACAACAAATCAGCCAACAGTATTTTTCAGCCGGCTTATCAGCACAGCTGAACGGGGCCATAGTTAGTTTACTTGGCACACATTGGAAAATTCATGAAAATTTCATGGGGGCGTGTTTGATCTCATCGCTCAGCACTGTGTTGCTCGGCCAAGCGAGAATCCCAAGCCAGCCTACACGTTTGGTTTGCTTGCTTGCGCATCCTGGAGCTTATGCTGGCAAGATTTGGGTCGTCGGTGTGGGAGAGCCAAAACGGCTCGCCGTCAGAGCGAGCTTCTTGTGTTTCGCGCCGAGAGGCGCGGCGAGGTGAGGCCAGGGGGCGGTGCTGCTGACAACCAAACACTCCTTAAATAATCTGACATGACATATTATAAATGAAGAAAGTGGTGATAAAAGTTTTATAGGATGAAATAATTTTAAAGAGGTGAAATTTGTTTACACTATTTCTAAGGTATAAGAGCCTTGGAAACTGGGACAACAATTGAGACTGGCCTAAGAGCATCTCCAAGAACCTTTCTTAAACTCGCTATCTAAATCATCATTTGAAGAGTTATTTGAATAAAAATTGCTCTTTATATCTTCCCCATCCAACATATTTCTTATACCCTGTGCGCACTCTAGAGAGCGAATTCCACTCACCATCTTTGACTAGGGAGAAATCCAGAATAGATGATGACAATATTTAGAGATCCAATTAAAGAATGCGCTGGAATGTTTTTTTTACCAAAATCTCTATTCCTATCCATAAGAAAAGACATAGAGAGACCCTTAAAATTGTTATAAAGTTGGAATAAAATAGTTATTTTCTAATAGTCCCACCTCTTATGTCCCACGACTCTTTGTTGCTTCCCTTCGTGCCTCCGGCCAAGAGTGGATCCAGAATTTTCATCCATTTGAAGTCACTAAGCTATAATCTTACCAAACATCTATGATCTTTTGTTGACATTATAGTGGTACTCGTACAAAGTTATTGATGTCACATGACATCAATGTTTATATCGTGTATTCGACCTTGCCTCCGCCCATTGCTCCTTCGACGCCATCACCTTCTAGGCTTCTATCGTCCCTACCGAAGCGCTACCACCTCTCCCTTTGTCTGGCCCCTCATCCCTCCTCTTTGCCCGAGCTCCTCTTCCCCAACCATCTAAAGTTGTGCATGGGGAGGCAAGCTGCTAGACATAATCTCATCTCAGGTTTGATGCCTCAAGTTACTGAGACTGATATTATAAGTCTCAATTCTCCAATATGCAGATGATACCCTCTTATTTTGGAGGATAATTTAGAAAAAGCTAATAACTTGAAACGGTTGTTAATTTGTTGTTAAAAGTGATCTTCTTGCCATTGGTTAATATGCAGATGATACCCTCTTGTTTTGGAGGATAATTTAGAAAAATATAATAACTTGAAATGGTTGTTAATTTGTTATTAAAGGTGATCTTCTTACCATTGGTCTTGATGAGGAAATGGTGAAAAATATGTAAAGATTTTATGTTGCAAGGTTGGAGAGTTTCCTATTAAGTATCTTGGTGTGCCTTTACATTTTATAGAACTTAGAAAGGAGGATTTGCAACCTTTCATTGATAAGATTATTAAAAGAATTGCAGGGTGGAAGGGTAGGTTATTGTCCTATGCATGCCGGCTAATGCTTCTTAAAGCTTGTTTAGTTAGTACTCCAATCTATCTACTTTCTATCGTTAAATTCCCCAAGCGGGCTATTGAGATGATCAATTCTCAAATGACTCATTTCCCTTGGAATAACAATCTTGGTGTGCCTTTACATTTTACAGAACTTAGAAAGGAGGATTTTCAACCTTTCATTGATAAGATTATTAAAAGAATTGCAGGGTGGAAGGGTAGGTTATTGTCCTATGCATGCCGGCTAACGCTTCTTAAAGCTTGTTTAGCTAGTACTCCAATCTATCTACTTTCTATCATCAAATTCTCCAAGCAGGCTATTGAGATGATCAATTCTCAAATGACTCGTTTCCCTTGGAATAACAATGAAGATAAGCACAAATATCACTTAGCTAATTGGTAACTGAATGGCCAGAAAAAGGAGTTAGGTGGGTTTGGCATTTTAGATATGAGAAGCCTCCCTTTTAAGCTCTTGGGTCTTTAGGTATGATTTTAAGTCATTTGCTATTTGGACAAGAATTGTAGATTATAAATACAAAACTAATAGACCAATGTGTTGCGCTGCCCTGCTGATTTTACTTCTCCTTTTTGAAAAGGAGTGGTTTGGGTTGCATAGGTAGCTAAAATGGGTGCTCTATGGAAAGTTGGCAATGGCAACAAAAAGTTAGATTTTGGGAGGATCACTGGTTTGGGAATTCTAGCTTACCTATTCAATTCTGACCTCTTCCTGTCACTAATGAACAACAAGGTAAAACTATAGCACAGGTTTGAGATGGAAGTGAGTTGAAACTTTCTTGTAGAAGATCTGTCTCTGAAAAAAACTTATGAACATGTGGTATGAGCTTTTAGCTATAACAGAGGAGGTTACTCTAGACAAGGAAGAATGATCAATAATATAGAGCTTTAGCTCCGGTGGCAAGTATTCAGTGCAATTCCTCTATGCTGTTATTTACCACAGAGGCATTATTCTTATGTTTGTACATCCTATCTGGAAACTTAAGATACCTCTTAGGTTACAAAATTTTATGTGTTTGCTTTAAAAAAAACAAACTCCTTACCAAAGACAATCTGGTCAAGAGAAGACTGGTAGTTGATCAGACTTGTCTGTTCTCGGCAGAGCAAGAAACTATTATCCATCTGTTTCTTGATTGTTGTGTGGCGAGTATGATTTGGTCTGTGTTGTTAGAAATCACAACAGTGATGGGTGCCTAGGATTAAGAATATGTAGCTTCAAGATGGATTGCAAATAAAAAATATGCTATTACCAATATAATCAGCTTTGTTTTTTGGGGTTCTTATGAAAAAATTGAAATAAATCATATTTTCAGGGAGCTTGTTGGACTGGAGAGCGCGAGGTTCTAATTGGGATCACCAAAACCTTGAGGAGGTGGAGGCCAATGTTTAAACTAGAGACCATGTGGACACGTTGGTGAAGTTGTTGGAAGTGCAAGCGAATCAATCATCAATAATATGGTCGTGTTTGGATGCACCAACTAAATTTTAGCTAGCTAATTTTAGCTGTGGTGCATCCAAACAGCCCACAGCTAAACCTAGCTAAATCTTAGCTAGAGGCAAAAATGTACACAATGTTTTTAGCTGTTTCAGCCTAACTAAAACTAGCTAAAAGGCATAAATATTCAAACTACTCCAACTAAAGTTTAGCTACAAAATCCAAACACCACCAGCTAAACAATAGAATTAATGAAGGGTAACTTTTTGTCAAGATTTTCAATTAGCTGGCTAAAATTTAGCCAGCTAACTTTAGCTGGCTAAATTTTAGCTCTAGCAATTAGCTAGCTAATTTTTAGTAAAAGTGGATTCAAACAGACCATGTTGGACAAATTAGAGGACAATGAGCTCATTAGGGTTGGAGTCACTGGATCCTCAATAATCAGCAGCCGCAACTCGGTAGATTAGTGAGAAAGTAGAGGAGGAGCTATATGATTTCGATCTTTATTGAACTCTATTGGTTGTTAGGCCATGTTTATTTTCTGATGGTGTACATTAGAGCTTGGGGCCGTTTGGTTAGTTGGAACCGTTTCAATTCTTTTGAGAATCACTCGGAACATTGCCAAACGGTCGGACAGTAGGTTGTTTATTTGTGGCATGCTGAAAAAAACTTTAGGGCCAACTGCTGCTCCTGTCAACTTGTTATCAGTTCTGACTTAATTATTTTCTTTTTACCCATGCATTTTTCAGTTTAAAGAGCTTCTGATAGATAGCTGACATGCTGCTCCTCTCTTTCCGTAGGTTTGCTACTCCTATAGATCAGTGTTTTTGTGCGGATACTGAATAAAGAAATCTAAACTTTCCAATCAAAACCGTGAATATCTTCACGACACCGCCAGCAACGGTGGCTGATGGCATGATCAGATCATGTGATGTGTTGAGTGTTATTTATCTTCAATTCTGAGTTGAGATTTTAGATCATGTGATGTATTGAGTCTTGTATTTTATGTTTAATAAAGGTCTCTAAAGATTCTGTTTAATCATGAACCACTCTTTTTCTTGCTCTGTGTGCAGTTCAGCTTTGGAGTGCTAGCATCCACCGGAGTTGCATGCTGCATTGGACATGACCACATGAAATACATTCCTAGAAACTTTTCTTTGTTATATAAACTTTAGTTTGGATAGTTATAACTCACAAGGACGTGAGATCGTGTAGGGACGGGCAAGTTCCATCTGGAGAGGAGGTAAAGGAACATAATATGGTTACTGTTGCAATGATATATGTAGGCCTGCTAGAATATTTTTAAGAATTTTGTTCTAGAAGTATAGAGCTGATATGTTGTGTTTATTGCCTTTCTAGGGTTAATCTTAGGCTTTTGGCGTTTAACGTTGTTATTTCCCTCCTTTAAAAATTTTTGTCTTTCCCGTATTGTTAGCACGGGCATGCAATGATTTAGGCGGCTTCAAAAGATATAATTGTTGTGAAGTTATTTATTTGCAATGTTGCTCTTTAAGAAACATAGTCTTTTATTTTTTACCATAGCATTAGCACAGACACAGGAAAACCACTATCGTTTGGTTGTTTTTATGGTCTAATTGGTTTACAACCAAAATTTGTCATGCCAAAGTCAGGTAAAAAATCTTGCCATAGATTTGACAAGCCAAATATGAGGAATTGACAAGTTTTGGCAGGAAATCAAACAATGACCCTGTTCATCCGGCTTATAATCCGTCTTTTTCAGCTTGTTTTTTTCAGCCGGAACAGTATTTTTCTCTCACAACAAATCAGCCAATAATATTTTTCAGCCGGCTTATTAGCACAGTCGAACGGGACCAATAGCCAAAATTATGACTTGCCAAATCTTGGGTAAAACAAATTTTGATAACCAACCAAAGCACTATGTGCATTCCTGGAATACCACCGTGGCGTTAGCACAGTCACAGTAACATAAACAGCAGACAGATAGCCGACAAATGAATAGAAATTTCGATTTAATAAAGCACCAAAAACCTGCACCACTAGAAATGGAATTCCACTTCGTTTTCTTGAGAGCCCGGGTCGTGTGGTGCGGCTTCGGGCTATGTGGAGGGTCTGTTGTGAGGCTCGATGGCTCCGCTCGTCCGTCGTCACTCGTCCGCACGGGCACGGCTGCACGGTGGTGGGAGCAGAGCAGCCGAGGAGGCAAGTCTAGCCCGGCGGCCTGGCCGTCCCTCACTCCCTCAATCCCGCCCTCCCTCAGCCTCAATCCCTCTGGATTAGTGGAGTCTGGATTCTGCCACTTCATTTTGGTATTTCTGATTTCTCATGTAGAAATTAATGGGTCGCTACTAGATAGGAGCATAATGAGTAGTTTATACTTTATACCTCTAGGTAGCGCCGTAGCGGCCCTGCTCTTTGAGCAAATTCTGGGTCCGCTACTGGCGGCGGCAAGCTTCGTCAGGTGGGTTTCTCCGGCCCTGGAGGGGAGGTAGGAGGGGCGGCTACAAGGAGCTTCTCGTTATGGCGCTGTCTAAGGACGACTGCCTCGACGGCGCCAAGGTCGCGGAGGTAGGAGTGGGAGGCTACAAGGAGTTTCTCGTCATGGCGCTGCCTAAGGACGACGGCCTCGACGGCGCCAAGGTCGCGGAGGCCATCGGCGTCAGGCTACCGGACTTCGGAGGGGCCGTAAGGGTAAGGAAATCTCCACCTGATTTTATGTCGTCTCCATGTTGAACTAATTGAAACTGGCGTACTGTGCTTTCCCCAATTGGCCGCGTGGTTTAGTTTCAGGTTTTTGATGTGATGATTCGTCAGAGTTTCAGATTCCTATGTTTTGTCACAACGGCAAGTCTTTTTGCTGCTGAAGTGCTAATATGCGAACTGTGTTTGATCAAGCGAGATTTTTTTTTCCCACCGTTTTGCAAGCAATGACTTGCTGTGAAACCAACCTTTGGGCTATAATAGGATGTGGTATTTTGTGGTCCCCTACAGAAGCACATAATCGAATGTACATTACTTACCTCAATAACTTGTAATTTACACTATAATGCATAGATGTGGTGTCTTTAATAAATACTGAAATGCCTTTTGTTTATACGATGGATTAGATAACGTGGTTGCTAATTTGTTACACTATTGAAAAACAAAAGAATTATTAAATGCACTCTGTAACTTGAAGCATCTTACTCTGTAATCTTCGGTACATTGATTGACAACCATACAATGCCAATGCACTTGCAGTTGCAGGTAGGCTTTACCTAGAGAAGTTGCTTCAATTTCAATTCTCATAGATGCCATATGAATTTAAACTTTGAAGCTAGTGATGAATTTTTGACTGATTGTTTGAGGCCTTCCCTTTTATCCAATTCACCACAGACTATTTTGGAGAGCAGAGAGGCCAGAGAATTTGCCAGTGGGGCACTAGCTGGTGCTATGTCAAAGGCTATTCTTGCCCCTTTAGAGACTCTCAGGTAGTCACTAACTTTTATCTCTACCTTTAACCTTTGCACTTGTCCAGTTTAATTTATTTACATACAACTGTAGATGCAAATCCTACGGCTAGTTTGCTGAACGTTGAAACTTTACGCTTGGTTTTCATTGCTTCTTATTCGAGGTTGCATGCACTTTGCTTGGTTTTCATTGCTTCTTATTCGAGGTTGCATGCACTTCAGAGATGAGATGTCCCACCAACCAGATAATTACCCATAGCACATTTATCTTTTGCATGATGGTGTGTTCTGAAATATGACATATACCGATGATTCACAAATTACTTAGTTTCTCCAGAGCACTACCTTAGTGATGTGCTACAGATTTTTCTGGTATTGGTCCCCTAATACATCGAATTTGGAAAAAAAAAACTCTTTCTCTCTCTCTCTCTCCATGCAGTTTCACATTATATTACTGCTGCATATAACTAAAACTCTATACTCCATCCATCCTGAGATTATGGGGTAAAACAAATGACGCATGCGACCTCCCTTAATCGTGGGGAGATATTGTGCTTACCTTACTTTGAGCCTAGTGGTTGGGTTGACAATAGACATTAATGGCAGGTAAGCATGTGATTGGGGGCCTGGGGGTATTAATAAGTGATGAGGGGGAGTTAATTAATCTACAATCCTTTATAATTTGGGACAAATTTTGAATGCTTCAGTACCTTATTTTAGGATGAAGGGGGTATCAGTGAAAAGGGAGGAATTTCTTGACGTGTATAAATATATTATTTTGTTTTGTGCGCAGCAGAATATATTATTTAGTCGCAAAAAAGCAGAATATATTATTTTTAAAAATTATGCAAGCTTATTTCAACTGTGCTGTTGAAACATGTAGTTCAACAAATGCTGATGCTGAATAGTTTCATAATTATCTAGTGAGTAAGGCAGAAGGAATGGTCACATATACCATTGTGTACAAATTTCAGGACAAGAATGGTTGTAGGAGTAGGATCTAGGCATATTTTTGGTAGTTTGGTGGAGATCATCGAACAAAGTGGGTGGGGAGGGCTTTGGGCAGGAAATACAATCAATATGCTCCGCGTTATTCCAACCCAAGCAGTTGAACTCGGAACATTTGAATGTGTCAAGAGGAGCATGACAGAAGCACAAGAGAAATGGAAAGAGGACGGATACCCAAAGATACAGCTTGGTAATATGAAAATCGAGCTTCCACTTCACTTCTTATCTCCAGTTGCTATTGGCGGTGCTGCTGCTGGAATAGCTGCCACATTGGCGTGCCATCCTCTCGAAGTTATCAAGGTAATTGCTGAAGTGAGGCCTTATTGACAGCCAGCGCTATTTCTTCATCCGTACATTCAACTGTGAAAGTCATATTACATTGTTACTTAATGTCACATCATTGATGTTCTGACAGGATCGCTTGACTATCAATCGAGAGCTTTATCCAAGCATTAGCCTTGCTTTCAGAAGGATCTACCAGACTGATGGTATAGGTGGTTTCTATGCTGGCCTCTGTCCAACGCTAATTGGCATGATTCCTTACACCACATGCTACTTTTTTATGTATGATACAATCAAGACCTCTTACTGCCGCTTGCATAAGAAGCCATCTTTGAGTCGCCCTGAGCTTCTACTTATCGGGGCTCTTTCAGGTAAATGAATCAGCATCGCATTGCATCTAGTTTCACTCGGTCACCTGTTGCTTACCGCTTCAACATTGTCTTAAAACTGACGACGAGATAGAAAAGGCAGGGCCATTCCGTCTAATGATGACTAGGTGTCTGAGCAAATAGGACTGACATTTTATTGTTGGATCCACAGGTCTCACGGCAAGCACAATCAGCTTCCCGCTGGAAGTAGCAAGGAAGCGGCTGATGGTTGGCGCCCTGCAGGGGAAGTGCCCGCCTAACATGATTGCTGCTTTGTCAGAGGTGATCCAGGGGGAGGGCTTCCGGGGGCTTTACCGTGGGTGGCGGGCGAGCTGCCTGAAGGTCATGCCACATTCCGGCATCACCTGGGTGTTGTACGAGGCATGGAAGGATGTTCTTCTGGCTGACAGGAACAAGCCGCGCGGCTTAGCAAATTGAACAAGCTGTTTTTTCAACAATGGAGGTGTTGCCGCAGATGAAGTTTTGCTACTTTGCTGGGATTTTTTTTTTTTATCTATTTTCAGTTTTGGAATCCCGGCTCCGGCGAATGGGTATATAGGGTGCGTCTCTCTTTTTGGCTGAGTGGAAAGCATTTGTTGTAACATCAATTCTGAACAGCTGATTGATTTTTGTTCTGTTATAAGAAAAGGATTTGAGTAGATTGATTGCCGAATGTCTGAGTAGAAAATAAGCCTTGCACACAACCAAACAGCCATATAAAAACAGAAGATGTTGCATTTCCTAGAATGAACAGTAATACATAAGCAGTGCTTTTTTTTTACAACACATAAGCAGTGCTTTGTGCCCTGTTGGCTCGTGTGAACCGCCTTCAATCTGTTCGTGTTGGTCTGGGCTCCTGGCCGAGTTCAGACTTGGGCTTCGTTTCATTTCTACAGCCCAAATTTAGTTTTGGCTATTCGGGCGGCCCAACAACTTCTGCGCAGCAACCAAGCATTCAAATTCTTTCTCCTCTCTCCAAAAAAAAAAACCATTCAAATTCTTTCAGTCTCGCTCAGCACTCAGAACAAAGTAGAAAAGCATCTCATCAGTCCTCTAAAAAGGCGAGTACAGTGTGCAGACCATGATGCCGAAAGCACTTACATAATTTACATCTAAATCCGACAAAACTGCAAAACCAATAACGGAGGGAACAGTATCATGCCCGGCATCAGTGGGTGTTCCTCTTCGACAATTCTGTTCTGTCTAAACATTTTTTGTTTGTTAGGTTCCTGTTGCTGCTTGTCAGTCATCCCATGCTAAAATGCTATCGCTGTAATAACAGTAGTATAAAACTGGACAGACATTCCTCTTCGACAGGGAGACAAGGTCCACCAGAGGGCAAAAACCTTTGACCTTTCAGAGGGTAGAATTTCAGGGTCCTTATGAAACTCCCATCTACCATTACTCATACCAAGTCAGACTCCACAGAATCCTGCCGCCACCACGGAACCCTAGCCTTGTAAGAATGGTGCTCGCCTTCGTGATCCCAAGAGATCTCCGATGCTTGCAGGCCAATCACGGATTTTGGTTCCTTGCCATGGGAGGCAATCCATGCTTCAATGGCGCCTTCTTTCTCCTCTTCACTGTTGTAACTAAGCCTCTCTCTACCGAATGGGTTTACGCTTGGGTTGGCCATCGCTGCCATTGCTGCAGTCATATATGCAGAATCCCCTATAATTGGAGCGCCTATAGCAGCAAGCTGTGCCCTTATCTGCAACATAATGGATTTCCAAATTAGATCCTATTATGATGTACTATAAGCAAGCAATCACATTCCTGGCAACGATTAAAAAGCAAAACAAAACAACCGCGTATGCATAGCTTCCTTCTTGCTTCTCCAATCTATGTAACTCTGTACTTTGCTGTTATTTTGTCTTAATAAATTTGCAGTAGGAGCCACTGGCTCCTCCTGATTCCTCAAAAAAAAACTGCGTATGCATAGCTCATTCAACTAAAAACATAAGTGTTGGCAAAATGAGCAAACAGTGCTTTAAGGTTCTCCAAGTTGGCAGGCTATTAGCTCAGAAACCTTTTTATTATAGAAGGGGAATAATCAAGCCTCTAATGATGTATGTTTGACCGAAATTACATGATACCTGATGAGTTTTCCCTGTCAAGAGATTGATTTTACATTCATAAGCAGCTTCTTGCTGTGGCCACCCACAACCATTTACATTGTGAACTTTCCTGATCGAAGAGCTTGGCCATGGTACCTTCCTACAATCGAGTACCTCCATTTGACAGAGATGCCATCTTTCAATACAGTCTGGTCAGGTAAGAAGAAAACATTAATTAATGTACTAATAAAGGATTTCAACGTAACAACCAAGACCCAATAGACTAAAACAAGAAGGTTATTTAAAACTTCTGATGCAGGCATTCAGGACATGAGTTTATGCAGCATTCATGTCTTATGATGCAGCATTCATCCTCTAGAGCTTATTAACATCCAAACAGCGGTTAAGAACATAATAACAAGGCAACACGCGGATGGTTGAAGGAGAATGGACGAGTTTTAGATATAAACTGCTCATTCACATTTCACTATCTTCAGTAATGGCCACAACTTAACAAAAGGTGGTCAGTGTGCACATATGTTCATAAAGATAGCACACAGTTTCACCCAAAAATGAATAATATTCCTGACCCAGATATATGCATGCTTGAAGTCAGGACAATGGTGCCAGCATCAAAGCACAAAACACATATTGGCCGAACTAATAACCATATAGAAGCAACAAATTTCAGTTCAAAATTACCTTCAGAAACTAATCTAGGAGCGCGATTAACTGGACGCATATAATGGGTAATCGTTCCTGTTGACACAGGTGCTGTAGTAAGTGCAAGATAAAGTTTTTTAACCTGTTTCTCCTGTTACAAAGTGCAAAATAAAAGGAGGGATCATTGCCAGTAGGTTCATGGAGCAAAAACACACTGCATTGCAGTCAGGTAGTAACATACCCTTATCAGTCCATGGAAAACTGAACAGAACTCTTTCGTTTTAGATAGTACCACACTGCAAAGTTTTGAAAAAGGATGACATTACTTACCTATTTCCCACATTTGAGTATAATCAAGTAAAGTGATAGACAAACCATCCTTCAGAGCAGTTGTCAATCTGGTGAGTCGTCAGTAATGGCGTTTCCAATCCTAATGCACGTGAAGTAAACACTGCACAGGATTCCTCGATGTTATCAGTTGCTCCGCCCACCTAAAGGTTGAAAATTCACCGGTTGTTACCATTTGAACTCAGAAAGTTCGACCATAAACTAGTGACTCTCTGGGCATTTCTAACTTCACCACCTACAATAACTTAATAAAACAAATCTTATTTCATGAATAGTCCCATGTGGCATTGGTAGTGCATACGCATTTTAGTGGATGTGAATTTTATTCTGCGAAGGTAACCTAATGGTGAAACAAAATCACTATCATATTCATCGGGAATTCAGACAACATCAAGTTAAAGACATGAACAATATGTTAGTAGATGTCCAATCTTGCAGACACACATTCCATCCTACGACATCTTGCAGACACACATTCCATACTCACGAGCACAAACAAATGAGAAAGTGGAAGATAAAGAGAAGCATACCGATGTTGCAGCAGGTTTATCAAGAACAACATATTCATCAGCGACTGCTATTACCCTGGATTTCCAGTCAATTTCATAACACCTGTGACAGGATCGAGTAAAGGAACATATTCATAAGCAACCAAGCTGATCCAGTCATTCACCACAGCAGCAAGAATCCACAGCAGACAAGAGAGAACAGCATCCACCCTACCTTGGAAACCGTTTGGGATGCACATGAACCCGCACATACGTGCCGGCCTCGAGGCGATGGTTGGGGTCCGTCACCCTGAACGTCTTCTGAGCCTCCCTCACCGTCTTCCCCTTAATGGACGCCCTCCGGCGCAGGATGGAAGGCTCAGTGACTTCCCGAAAGATTTTAAAATGCTCCGGAGACGCGTAGGGAGGCGGCTGCGGCGCGACGAGCGCGTAGTACACAGCACCGAACTTGATGAGGTCTGCAGCATATCTTCACATCCACAATGTTAGGCATGGCTCACTCTAGTCTTACGGCAGCCAATCCATTCCGTGTTCAGGAAAGAGAGCGCAGGAGGGAGAGGTGTGCTTACAGAGGAGGCAGGCCGAGCGACCTGGAGATGAAATCGGCTGCCACCTCGTCCTCGCGAGCCACAAGGTGCTCGATTCTCGGAGGGTCGTCCTGCAAGGGGCACGGTAGAAGCCGGTCGTACACTGGGTACCTGCGTCACGGAGCACACTTCAACATCAGGGCGCAGTGCATCACCAGGGGATATCAGCTGCTGGGATAGTGGAAGGGTTGGGGGAGGAGGAGGAGGAGCTTACGGTGTGGCTGCTGGGGCCTCGACGGCGGCGGCGGTTGACACTGCGGCGAGGTGCGTGGAACGCGGGAGTACGGAGCGGCGGCGTGGGGCCGAGTGGGCGGTGAGGATCGAAGAGGAGGAGGAGAGGCCGCGGGCGCGGGAGAGGATGGGAGCGCGACGGAAGGGGCGGTGCCATAGCTGCGGGAGGAGGGAGGCGACGGAGGCCACTGGCTTCGGCATCCCGGCGGCGGCGGCGGGAGGAGCCGGGGGTTCTGCGTTTTGGGGCTGGGGCGTTTGCGTTGGTCCCGCTCACGAGTTCAGATAGGATATC

>20

TTGGAAATCACGAGATGAATCTTTTGAGCCTAGTTGGTCCACGATTGGATAATATTTATCAAATAAGACGAAAGTGATACTATTCATCGGGTTCACTTTTTTTTTGCAATCTAAAAAGCCCTAAGAATCGGGACTCTTGTCGTCTCTCACCTTTTCCTCTTCCCCTCGTGGCCTCGTCTGTTCCCGGCGGCCGTTCCCCTTTCCCCTCTCCAAAACCTCTCCCAAGGCCCAAACCCTTGGAAACTGCGCCGCCGGCGCCGCCCTCGCAACCACCGCCGCAGCAATGCGCCGATCTCAGGACCGCGGACCCTTCCGTCCGCCGGATTGGGTCCCACAGCCTCCCCACCTCCTCCGCGACCACCACTACCACAACGAACACCGGTACCAGCCTCACTCACACCCCCACCGCGACCGACACTACAGCACCGAACACCGGTATCAGCCCCGCGGCCAGCACCAGCGCGACTGCCACGTGCAGCCGTCCCCTCCCCCGTCGCAGTTCGAGGTACTCCTCGTCCGCCCGGGCCCCGATCTGTCCGGCCCGACCGCCATTGAGGTGGAGGCCCTCGTCGCGGACCTCAAGTCGCCGACGCCGGCCAGCATCTCCGTCCATTCCTCCGGCCGCCATGCCGCGCGCCTCGTCTTCACCTCCATCTCGGACGCCGCGGCCGCCGCGCGCGAGCTGTGGGCGCTCCGCCTCAAGGGGCTCCACCTCCTCACCCTGGACCTACCGCACCCCGCCCTCGCCGCCCACGCCTCGCCGCACTTCGCCTCGCTCTTCGCCGACCACGCCTCCCGCCTCCTAAACTCCGACCTCGTGGCCCTCTCGGCCGCCCGCTCCGCCGAGCTCGCGGCGTCCATACGGGATGTAAAGCAACGTCTGGGCTCGCGTAACCGCTTCCGCGACTTCCATCAGCTGCACCTCGAGAAGAAGACACTGGAGTCTGAGAAGGAGTTGATCGATGCCAAAATTGCCGAGTACAAGGAGGCGATGTGGTCGATACAGCGCGCAATGTTGCGCGGATCAGGGGACAAGGAGGAGGGTGTCGACTTGTTTGGGGCTGTGGAAGGTGCGGATGTGGACTTTGTGAGGGTGCATATGATGCTGCTGCGTGAGTGCCGGAGGCTTAAGGAGGGCCTGCCGATCTATGCATACCGCAGGAGGATTCTTAATCATATTTTCGCTAACCAGGTAACGATGGGTCATTGGAATTTGCTATGTGATTTTAACTTCAAGGGTATTGCAGTGCCATGTGAATTATTTGATGTTTTGTGCTCGGTATGCTTAGAACTGTAGAGAATTCTTGTGTCTAGATTTTGGTAGTGAGTGCCTTCTTAGACTGTAGTAAGAGAGAATTCCTTTAATGCATTAGAAATTATACTCCTCCCTTTTGTCATAGGGAATTATACTCCTCCCTTTTGTCACAGAAAAAATCGTTTCTGTTATAGGCCATTGGTTTAAGTTTTTGTTTCCTAAAAAACTCAGACATCCTGATTTTGTTCGTAAGAGTTTGTGGCAGCCTTCGAACCCGGACTGGTGACGGGGGTTCCATCAAACCCCGAATTTCAACTGTCTGACCACAGTATAACTCAGGAGAAGTTGGCCATTTTGCTCCTCTCCATGCCACTTCTGTTGGGTGCTAGAATGCTGTGAAGTGGTGAGGTAAAAGATGATTTTGCCCCTTTAGATAGAATGAAGCAAGGCTTTGCTATTTCTTTGTGTAAATGCAACGACATGACATAATGCCTCTATGACATAGTGCAATTGCAGCATAGTTAAACACCAGCACAGCTTGCATGGCCATGCGTGACCCAGCTGGCATATTATCTCTAATCGCAACAGTTTTGCCAAACATTACAAGAAAAATCTACATTGGCTTATGATTTTTTTTTGCAAGAATTGGCTTATGATTTTGTTTTGCCCAACTATGAAACAGGTGCAGAAAACGATGTTTTATTCATTCAAGTAAAAATCATGTCAATAGTTGATAACATTTTCTGTTTCTTCATAATTCATATCCTCTCTGCATATGCAGATAGGCCCTCCCCTCTCTGTCTCTCCATTCCTTTCTCCTTTTTCCTTTTGCAAATACAGCAACACTTATAACCTTCCATATGGCTAGATGAGACTTGATAGTTGGATGCTTCGTTCTGTATGGGTCATAATGTCTGCTCACTAGCACAAGTTCCTTACTGTACTAACCTCTTTGCAAATACTCCAGTGAAAGCCTTTGTCGTACGGTCACGATTGTTGCGCTGTTTCCAAATCTCCCAAGCGACCAAAATAATTAACGAGCTGAGCCCTTGCTTGCTTTCCTTCTCAATCTGTTTAATCACCCAAGACCACCAACTGGAGAAGCTTGCTGTCTCTGCTGAGGTGCTATAAAGGTAAACCACACGAGAGGCTCGAGCTTTCAGGGAAAAAATCAGATGAAAGGAAGAACTGGCACGAATGCAGAATGATTTATGCCTGTATGGTACATAACATATATAAGTGTAATGGTACAAAAGGGAAATAGCCAAGTTACTCGTAATCCTTCCTAGTGGGTAAAATTGTCTTTTCCCTCGCCATTCCACAGCAGTATAACACCCAACTAACAGAAGTGGCATGAAATGGTTCAAAATTTTGAACAAGTGGCACAATTCTATGGATTGCACATAGGGAAGAGTGTAATTTCTAGTGGCATTGAAGGAATTCGACCTTTGGGTTGTTCAGCATCCTAAATAAACTGAAATTGAGCCCTCTGTTTTTTATTCTCATGTGTATTGGCATAACATTCTTGCTTTTCCATAATTATGAGAAGAAGAAAGGTTATTCCGTGCATTGTTTTTTTGACGGGGCATCAAACTTTTGATTCTGTGGCCTGTTCAATGTTCATCTGTTAGCAATTTACTTTATCTACAATCAGCTGTTATTTACTTTCATTTATTTCTAAAAGTTTAGACAGCATAGTTTGTCGTTGAGAAAAAATTTCTTGCTCTAAACTTAATGAGCTCCATTTTCCTTAGGTCATGGTTTTAATAGGAGAAACAGGTTCTGGGAAGAGCACACAATTGGTTCAGTTTCTTGCTGACTCAGGTATTGCTGGTGGTGGTTCCATCATCTGTACTCAACCTCGAAAGCTTGCTGCTATATCTTTAGCACATAGAGTCGATGAAGAAAGTAAGGGTTGTTATGGGGACAGTTCTGTGCTGTCATATTCAACCTTACTAAATTCTCAAGGTTTTGGCACTAAGATTATATTCACCACGGACAGTTGTCTTCTGCATTACTGCATGAGTGATATGAGCCTGGATGGCATTTCATATATTATTATAGATGAAGCTCATGAAAGGAGCTTGAATACTGATCTTCTGTTAGCTATGATCAAGAAGAAGCTGCTTGTTAGGCTGGATTTGCGGCTCATTATAATGTCTGCCACTGCTGATGCTGACAGACTTGCTGAATATTTTTATGGCTGTCAAACATTTCATGTTAAAGGGCGAACTTTTCCTGTTGAAATTAAATATGTCCCTGATATATCAGCGGAGGCTTCATTGAATAGTGTACCAAGTATTTCTTCTGTTGCTTCTGCTACTGCTTCCTATGTTACTGATGTTGTACAAATGGTAAACATAATCCACAAGAATGAAGAAGAGGGTGCTATACTTGCTTTTTTGACATCTCAACTGGAAGTAGAATGGGCCTGTGAAACATTCAGTGATCCAAATGCTGTGGTACTTCCGATGCATGGAAAGCTTTCTTCTGTAGAACAGAATCTTGTCTTCAAAAGCTACCCTGGAAAGAGAAAGATTATCTTCTGCACGAATATAGCTGAAACATCATTGACGATAAGAGAAGTGAAGTATGTTGTTGATTGTGGCTTGGCCAAAGAATACAGATTTGTTCCCAGTAGTGGTCTCAATGTACTCAAAGTAAATTGGATTTCCCAAAGCTCTGCTAATCAACGTGCTGGCCGTGCTGGCCGAACCGGAGCAGGGAAGTGTTACAGGCTCTACCCTGAATCTGAGTTCAGTATGATGGAAGCGCATCAAGAACCTGAAATTCGTAAAGTTCATCTCGGCACCGCTGTTCTGAGAATACTTGCTTTGGGCGTTACGGATGTAAAATATTTTGAGTTTATTGATGCCCCAGATCCTGAGGCTATCAATATGGCTGTGCATAATCTTGAACAACTTGGTGCTATAGAATATAAATGCAGTGGATTTGAGCTAACTGAAACTGGACGTGATCTGGTCAAATTGGGCATTGAGCCAAGGCTTGGGAAAATCATGCTTGATTGCTTCAGCTATGGTTTGATGAAAGAAGGTTTAGTCCTAGCCTCTGTTATGGCCAATGCTAGTAGCATATTTTGTAGAGTGGGTACTAATGAGGAGAAATATAAAGCTGACCGTCTGAAAGTCCCTTTCTGTCACCCTGATGGAGACCTTTTCACTTCACTTGCTGTTTATAAGAAGTGGGAGGCTGGGCATGATAACAAAAATATGTGGTGCTGGCAGAATAGTATCAATGCCAAGACCCTGAGGAGGTGCCAGGAAACTATATCTGAACTTGAAAAATGTCTAAAGCATGAGCTAAACATTATCGTTCCAAGTTACTGGAGTTGGAACCCTGAGAAGCCTACTATGCATGATACTTCACTAAAGAAGATTATTCTATCATCTCTTAGAGGTAATCTTGCTATGTTTTCTGGACATGAGAAATTTGGGTATCAGATGATTTCAGCAGATCAGCCTGTGCAACTTCACCCTTCGTGCTCATTGTTGACTTATGGCAGCAAGCCTGAATGGGTGGTATTTTCAGAAATCTTGTCAGTCCCGAATCAATATTTGGTATGTGTAACTGCTGTTGATCGTGACGACGTGTGTACAGTTCACCCTTTTATTAAGCCACTGGAGGAGAGTAAATTGCAGAGGAAAGTGATTACTGGGATTGGAAATAAATCACTGAGAAGATTTTGTGGTAAATGTGGCCAAAATCTGCAGAAAATCATCTCACTTCTGAGAGAAGATTGCAGAGATGACCGCATAATGGTTGATTTAGACTTCAGTAGCAGTGAAGTTTTATTATTTGCTAAAGAACATGACATGGAAACGGTCTTTTGCATGGTTAATGATGCTTTGGAACTTGAAGCTAAAATGCTGAGGGATGAATGTGACGAGAGAAGATCTGGTGGCTCTACTATTGCATTATTTGGCTCTGGTGCTGAAATCAAGCATTTGGAACTTGGGAAGAGACATCTAACTGTGGAGATTCAGCATCAAAATGCTCGTGATATAGATGAAAAGGAACTTATTGGTTTGGTATATTCCCATGTTCCTGGTATAGCAAATTTTCATAGACTCGGGAATTTTCAGACAAACGCAGATGAAACGAAGTGGGGGAGGTTTACATTCCTCAAACCAGATTATGCTGATCATGCCATTTCAAAATTGAATGGGATTGAGTTTCATGGTTCCTCGCTAAAGGTGGGTCCTGTAAGCGCTTACAACCACTCAGGGCTACCATTTCCTGCAGTAAGAGCTAAAGTTTCTTGGCCACGTAAGCCAAGCAGGGGACTTGCACTTGTAACATGTGCTAGTGGGGAAGCTGAATTTATTGTAAAGGACTGCTTTGCCCTTGGAGTTGGTGGAAGGTATATCAACTGCGAGGTTAGTAAAAAGTATGCAAACTGTGTCTTTGTCACGGGGGTTCCATTGCATGTAACAGAGCCAGAATTGTATGATGCTTTTCGTAGTACAACCACCAGGAGAATCCTTGACATCCACTTACTTAGAGGACCGCCCACAGCTAGCTCTTCCGATTCTGAATGTGCAGAAGCACTGATGAGAGCGATATCACTATTCATGCCAAATAGGAATTTTCCTGGCCAAAATTTTCGTGTTCACGTGTTCCCTCCAGAAGAGAAAGATTCAATGATGAGAGCTACCATATCCTTTGATGGAAGTTTTCACAGAGAGGCTGCAAGAGCACTGGACCATCTTCAAGGAAGTGTTCTTCCTTGTTGTCTTCCTTGGCAGATAATCCAGTGCCAGCATGTATTTCATAGTACTGTCTCCTGTCCAATGCGCATATACAATGTCATCAGCCAAGAAGTTGGTGTTCTACTTGAGAGCTTCCGTAGCCAAAAAGGTATTGTTGTATTCCTCGCCTGAGAGAAAATTTGCTTCTATCTTCTTACTGTTCTGTTGTCATGAGTCATGACATATTCTGCTCTTCATGCATTGCTGCTTTGTTCTTGTTTGTTATTAAAAAATTGGCTGCCTCTAATGCTTACTGCTGCTGTGTTGGTAGAACCTTAATCTGCTATTCCCTCCTATTCTTGTTTGACTGTCATTTTGTTCTTCAGTTTTATATGAGGCAGTCACAAACTGTTTTGAGTCATCCTTTATTGCATTTCATTCAATATAAGGCCTTCTCTTTCTCTTCGAAAATCCTTTGCCCCTACCATTTCTCTCTAGGAAGCTACTTATGGTTGTTGTCTGTTTAGCTTTGTTGCTCTTTTGTTTTCTGGAAGAATCGGAATCTAGCCTATTTCAAGGCTCAAGAGATGATCAGGTATTCAGGTTGTATCCAATTATATTGTTCCCCATGCCTTCACTTGCTTCAGGAAGAAACCTATTGTAAATTCTAGGTTTGAGTGTAAGTAGAACTTGCGGAGCATGAGCAATTAGATAGCGATCTCCGTTTGAACTATTTCGGTTTGAAAAGGGTAAAATTATGGTGTCGTTTGCTTCTAAGGCTCAATTGAAAAGTAATAAACTTTGAAGTGAAGTGAGCTGTATGAAAACAAATCATAAAAGATCAACCACAACCACCTTGGGTGTCAAGAATGTGTTGCATTAGTGAATAGGTATATTCCAATGCTCCCTTATAGTTAAATCCTATAAGCATAATTCTGTGCACCACCATATTATTTCTTCGTTTTAGGCTGCAAACAAATGGATCTTTAGTGAATTTAAAGTCTTAATTACATCGGGCTGCCTATAACAGCACTAGTCCTAGCCGTGGTGTTTTTCCCTGGTGCTGATGTTTTTTTAAGTGGCCAGCCTTCTATGTTGTTGATGCAGTGTGGGGTGTGTGAGTCGTGTTGCGCGTGAGTGTGAACCATCATGGGTCTTTCTTTTGTGCCCCCCTTTTTTCTCTTATAATGAAATGATACGAGAAACAAAGTCTTAATCACATTGCTATTCCTACATGCAGGTGTGTCATACAATTTGGAGAAGAACGAGAATGGTAATTTCCGTGTTAAGCTTACTGCAAATGCCACAAAAACAATAGCAGATTTGAGAAGGCCTCTTGAGCTTTTGATGAAAGGAAAAATTATAAACCATCCTGATCTGATGTTAAGCACAGTTCAACTGCTATGGTCCCGTGATGGTATGGAACATTTGAAATCAGTTGAGCAGGAGACCGGCACTTACATTCTGTACGACAGGCTAAGTCTGAATATTAAGGTCTTTGGCAGCAGTGATAAGGTGGCTGCAGCAGAGGAAAAATTGGTTCGTGCACTTGTACAGCTCCATGAGAAGAAGCCTCTTGAAGTTTGTCTTGGTGGCCGGAACCTCCCACCAAACTTGATGAAGGAAGTGATTAAAAAGTTTGGAGCTGATTTAGAAGGACTGAAGAACGAGGTTCCTGCAGTAGATCTTCAGTTAAATACACGAAGGCAGGCACTTTACGTTCGAGGCAGCAAGGAGGACAAGCAAAGGGTGGAAGAGATGATCTCTGAACTGATAGCCTCTAGTGACCATAATGCTCCACTGCCATCAAAGAATGCATGTCCTATTTGCCTGTGTGAGCTAGAAGATCCTTTTAAGCTTGAATCCTGTGGCCATATGTTTTGTTTGGCATGCTTGGTGGATCAGTGTGAATCTGCCATAAAATCGCAAGATGGCTTCCCTCTTTGTTGCCTCAAAAATGGGTGCAAGAAGCTCCTCCTCCTAACTGATCTGAGGTCTCTCCTGCCTGACAAGCTGGACGAGCTGTTCAGGGCTTCCTTGAATGCATTTGTTGCATCTAGCGCAGGATTATACCGTTTCTGCCCCACACCTGACTGCACATCCATTTACCAAGTAGCTGCTGCAGGCGCAGAAGACAAACCCTTTGTCTGTGGGGCTTGCTCTGTGGAGACCTGTACCAAGTGCCATGTCGAGTACCATCCTTTTATCTCTTGTGAGGCATACAAAGAGTACAAGGCAGACCCGGATGCGAGGACTCTGCTTGAATGGCGCAAAGGGAAGGAGAATGTAAAGAATTGCCCTTCATGTGGATATACAATTGAGAAAGCTGAAGGTTGCAACCATGTTGAGTGTAGGTGTGGCAGCCACATTTGTTGGAATTGCTTGGAGAACTTCAAGAGCAGTGAGGAGTGTTATGGCCATCTCAGGTCTGTGCACTTATCCTATTAGTGAATTATGTAGATCTGATGTAAATTTGTACAGATGGTAAATAATCACGTGGTCTATAATATGGCAAGCTGGTTTTTCCTTCCAACAGTGATTGTCTGATCCCAACAGGTTCCCATCTCTTTCTGTGTTGAGAATATATTGTAGGCGCTTTATCATTTTTTTTTTGTGCATCAGCGAACCCCATTTAACACTGCCCTTGCTCTCATCCTTGTGAATTGTGGCATCGAATTGTTCTTAGGATGTTGTACTCCATCACTGTTACTTCTTGTCTTCCTTGTGAATTGTTCTTTGGAATAGTATGCAGAAAATATGCTCTTGTAGTTTAATCTGGCTCGACCGGGCACAGATCTGATAGCAGAAAATGGTGCGGTAGGCGCAGGCCAGCGATGCCTTTGCTTACACAAGGCTCGCGCGGTATTTAACCGAAACATAATTCCTATTTCTGTTCTGTCTCGTTTGGATGCATTCTTGTATTTGTTCGTAGCATGGGGACTATCCGTTTAAATATTCTTGTTCATTCACGAGTTGGTTAGGCCTTGTAAAGCTTGCTTTTTGTGCCTTTTGCCAATGAGCTTCAAGGAAAATGGATGGCATAAGCTCGATCCGGCTGCGGGGTTCCTGGGTCCTCTGCCGCGGTTTATTGAACTTCATTCGTTGTTTACACACTTCTCCAAAAGTATCACATGACTGTAGATATGATCACTTGAGTGCACTACTTAATGATGTCCATCGGTACCAATTTCAACACAACTATACATGCGCCATGCACAAACACATTTACATGGGCATGGCACATCAACATGAGGATAGATACGTGGGCAATCTCATCGGCACCATGAACACCAACAACGGACTCCTGAAGATTCTTTACAGCGCTATCATAGTTAACGAAGGCCATGAACCAAGAAAAGACTCCTGAAGATTCTTTACAGCCTGAATGTATCTCTGAGCAGGGTTTTGCTGACTTACTGTGCGAGTAACTGACCTCAGGTGAGGAAGGGGGTAAGACAACTTGTTCACACGTACATAAATAATTGATCAGGCATAGTTGGAACTAGGAATTAGCCACAAGTGTATATATCTTTCAGTCAGCAAAATTCAGAGCGACTCAAACAAAGCAACATAGAGATGCTTATAAGCCAAGACTAGCAAACCCATGGTTTCCATTTTGTTAATATGCCAGTAAGTAGCAAACTTTGTAGCACATGTACCAATCACTATACATAAAAATACCGATAAGTAATGTGAGATTAAGTACTCAATAGTATTTAGCAACCAATTGGTCACGAATGTGCTGAGCTATTGGTGATTAATACAAGGCCCTAGAGCCATGTTTGAGACTGAAAAAAAAAGCCTTTGTCGATTCTGCTGCCCTTACAGTGCCGATGGGCATAGATGAGACTGGCAAAACCTTTGTCGATTTCAAGGTGATGTATCATGTATGTGAGGATATATAATGGAAATGGATGTTTCACTCGTGAAACAAGCAATCAAATAGAGTATACCAAACAATACTGGTTTCAACCAACTTCATTCGGTATTTACACATTTTATCCAAAAGCATTCACATGCTACGGATCTGAATGCACTACTTAATAATGTGTACCAATTTCAACACAAGATGCACAACTATACAGGTGCCATGCACAAATACATTTACACGGGCATACTACATCCGATAATGGGCAATCTCTAGGCCCCGTGAACGCTTCGAACAGCCTCCTGAAGATTCTTTACAGCGCTGTCATAGTTCACGAAACCCATGAACCAGAACTCGTGGTTGTCGACCGAAACGACCTGAATGTACCTCTCTGCAGGGTTTTGCTGACTTGCTGTGGGAGTAACTGATCTCAGGTGAGGAAGGGGCAAAACAACCTGTTCAAGCGTAGATAAATGATCAGGCGTGATTGTTACTAGGAACTGGCCACAAGTGAGTATATCTTTCAATGACCAAAATTCAGAGACTCAAACAAAGCAGCATGTAGATGTTTATAAACCAAGACTAGCAAATGTGCCGTTTCCATTTTGTCAATATAATATACTAGTAAGTAGCAACTCTGTAGCACACGTATCAATCTCTATACTATACATAAAACTGGGGATAACTGCAAAATCAAGTACTGCCTCCATTCCTAATTACAAGACGTTTTTGCTTTTTTAGATACATTGCTTTTGCTATGTATCTAGACATAGTGTTTATCTAAGTGCATATCAAAAACCATGTATCTAGAAAAGCCAAAACGCCTTATAATTTGGAATGGAGAGAGTACTTTATATTTAGCAACCAATTGGTCATGTACTCTAAAAGTTCATACATTATATTATAACAAAGTCATGTGAAATAACCATGAGTACCCTTTTTTAGCATCAGACCCGGAGTACCAAAGAACTCACTCCACTGCACACACCGAAGAGGAGGAACAGAAGACGCGGGCACCACACACCTTATAAAAGGATGATTCAGTTTTGTTGTCCTCAGTCACATATTTCACAGGGCTGTCGCTACCGAATGCAATCTTGACAGTCGAAAGATACAGGACGCCCATGATGGGTCCATGCGACGTTGATAGGTAGCACACGTATGCCTTCTTGAGCTTCTCATCAGGCGAGCACTCAAAAGTCTGCTGAAATATCTTGTCGTAGCCACCCTCAGATATAACCTTGGATATCTGAGCAATCCTCCCCATGGCAGTATCAGTAATGCTGGGTCCAGTTTTCACTGCAGAGATGCACACACAGAGTATTATTATTGCAGTAACAATAGAGTATTATTTCAGTAACAATAGTTCACGTGTTACTATTAGTGTGCTTCGGATTGGATACTACGGATCACCACCATAGCAGTAGTACTCAGAATTGGAGTCAGTGTGAGGGAAGTCGAAGCACTCACGGTGCTGCCAGACGTCGCCGGCGATGCCCTCGGTCTTGCGCGCGGCCTCGTTGAACTTCTTCCCCACCATGCCAAACATCTCCCTCAGATTCTCCCTAGCGCCTGCAAGCGGCGATCCATCCCCCAAAACCAGAGCGATGAGGCCGCAACAATCTAGGCAGCGAAGCGATCACCAAGAAGAAGATGAGCGGCGAAGACCGCATCGAGGGGGCGGGCGCTGACTCACCCTTGGCCGGCGGGTTGGGCGACGGGGCAGAGAGCACGTAGGGGTTGGCGCCGGCCGGCGGCACGACAGGCGGCGGCGGCGGTTCGATGTCCTCCGGGGACAGCCGCGGATACGCCGCGTGCTCCTCGGCCGCCGCGGCGGGCGCGCTGGCGGCGGGCTTCGGATCCATGGGCGATTGGGGAATGGGGATTGGCGTCGGTATGGGGGAGGGG

>BAC-18

TGGTATGATTTAAATTAATACGACTCACTATAGGGTCAGTGCGGCCGCGACTTCAAGTCACGTGAATTCCAAGCTTTAGGAGGAGGCAGACGTTCTGAGTGGCCAGGTCGCGAACACCCAAGCCGCCCTCCTTCTTCAGTTTCTGGGCAACCTCCCAACGGACCAGGCACTTGGCGCCTAAAGCCTTGTCTGAACCAGACTAAAGGAAGGCGCGCTGTCTGCTGTCAATGGCGGTAGAGCAATAGCCTGCTTCATGTAGGTTGCCATGCCATCTAGTGACGAGTTGATGAGGATGCGTCTCCCTTGATGGTTGAGGAGGGCCGATTGCCATCTGGCTAGGCAGCGATCCACTTTGGAGATGAGTGGACTGAAGGCAGCAAGGTTGAGCTTCACGTTGGACAGCGGAAGCCCCAGGTAAGTCTGATGGAAGGTAGCTTGCTGGCACTGAAGGATACGGACTATGCGCTGAGGCGAGGCGCGGGCACATGCATGGGAACAAGAGTACTCTTGGAGAAGTTGATTTTCAGTCATGTGGCTAGTGCAAAGTTGTCAAGGATTTTTTTTGAGTCTCTGGATGTCAGTCACCTCTGCACAGACCAACAACAAGGTATCATCGGCATATTGGAGGACCGGGCAAGCTAGGGCCATCAATGGCGGGGTGACGGATGCCTATGTCTTGATTAGCTGGACATTGGCCACTAGCAGAAACAGGTAGGGAGAAAGGGCATCCCTGTCTGAGTCCGCGCTTGCACTTTATCCATCGGCCGATCAGTACAACATGGTTGTGTTTTGTTCTTTTGATTTTGCTGTAGAGTGAAGGTAGCTTGTGCCATAGCATCAGTACTCCTTTTCATCATGACGTGATGATGTTTTGGCCGTGACGTACGCAACATAGTTGACGTACACTTGTTACTTCATATGGGCTTTGATTAAAATTTTATAGTATATGGTTTGTGCATAGCCCTGCTTTTGCACGTGTGCATAGCCTTTTAATATCATTATTACTATACTCTCTTAGTGTTCAAGGGATAAAAGACAGCACTAGCTATATCTAGAAGAAATGGAAGGGGCATATATATGTATGTAGTAATGATGATGGGGGACTTCCATTCCATAAACGAGGAATCATTACACAGTGATCAGAAAAAAATATGCTTAATGAATACAGTATTACACGAGTGTTGAGTTGGATGATGATACATCCATGGTCAAATGAAACCATGGCCATTGCAAAACAGAAACCAGGATGGACACCCCATTTCTCCCCTAAATAACCCGGCTGATTCCAGGTGGTGGAGCAGGGTCCACGTCGCTCCAAATTTTTTTGGCCGTCGGATCTTCGATCGTGTGGCCATGAATGCTTGGAGCGGATTCGGCCGGAAGCGGCTTTTGCTGCTCCTTGTTCGCTTCCATCGGTGTTGTGCGGCTTCTGCTCCGGAGCCAGCCCACCAAATATATACGCGAGCTCCGGATTCTCATTCCAGATTCTTCTGCTTCCCGATCCTTCTCCTCTTCGTTCTCTCTCCCTCCCCCGACACTCTCTCTCCCGACACAGGCAACGGCGACGCCGCTGCGCCGCGCGTGTGCCCCCATGGTGGCCGCAGCCACTGAGCGACGTGACCGGTCCCCGCTAGTGGCCGCCGGCTTGGCTTCCCCTCGGAGCTGCCTCCTCCCACCCATCCGCGGCGGCTCGAGGAAACCCTAGCTGTCGCGTCCTCGCCGGTCGCGCCTGCGGCTCTGCTCTCCTCCGCGCCGCCCTCCTCCTCCTTCCCCCTCACGTCGAGTTCTGCCAGCCAGGCCGCGGGTGAGCTGCCGACTGGGCCGACGGCTTCCCCCGGTGCGGGTGGGCCCGGGTGCGAGGCAAGCGCACCCCGGCGCGGCGCTGCGGGTGAGCAATACACACCATGTTGCTTGGCTGACACATCATTATTGTGAGCTGTCAAATGTACTAGCCTAGCACTTCTCCTGTTTTCCATAAGAAGGATTGAAACTGTACATTTCATTGTTGTTCAATGTTCACTAAGTCATTGCACCAAGGGATCTATTGGGTTGGGAAATAATAGTTTGGTTCCGAAAATTGGTTGTGTATCTCCAGTTTCCTTTGTTGTGGCACAAAGATGTTCTATCTTAATTATAGGGATGGCGTTAGCGAAAGCCAATTTTATAGGACGCTGAACATTGAGTTTTATCAGGTGTTAACCTTTGAGTTTGGCACAGAAACTATTCGTGCTTATATTCTACCTTTTCGCAGAAGTTGATGGCATAGTTGACAACTAAGAGTATCAATCTGAAATGAACCAACAACTCGGCCTCTGCAGTTCTTATTAAAATGGTATGCATTCGGTTCCATATCATCTATCCCTTTCTTTCCTAAATCTGTTGCCTGTGTTTTCTGGTCCATTCAGTTCCTCCATGGTGATGGTGTTGCTGCTTCAGCATACAAAATATTAGCATCGTTGCTAACAAACATGGTCATCTTACTCATTGGATTACACAGGAATGCAATTGAACTTGAACTTTGGTTGTGCAGGTCTCTATTGTATGCCTTAACGAGCACCAAAGTAAATCTGGCTAAAGGCCATCAAGAGAACCGTTTCTGTTTATTATTCATGAAAATATTGATTCACATTTTACTTATCAAGCTGTGTGCCTGGTATTCAGTGATGCCTGAAATAAAAAAAAAGTCTAACAGCGTTAACATGTTCACATTTCATTGTGCAACCTGCATAATATCCTTTCCGCTCAGCGAGAGTTGAAGTAAATGAGACTAGAGGGCATCAAGGTATAACTTCTCTGCTTATCACACATGTCCATAGTCTCTCAAACCTTTTACTTAACGAATGTGTTGTTGCTCAGTGATGCCTTGCCCATAGTCAAACAGACTAAAGGGCAGTGTTGACATTGATCCTTACAAGGATGTTTAGGAATTAATGTTCATGGAATCCAGCAATATAATATATTCTAATTATGGTTTTTCTGGGTGTCACAACCAAAATCCAAAGGTTTTGGGATGCTACACTTCGGTTGTAGAATGCTCACAGTGTGAGGCATGTTCTGCGCTCTCTGCTCATGCATATATCGACGTTGTTTGAGTCATGAAAGAATTAGTGTCCCTTGCATAGAGTGGTTCAGCAACAACCCCAAAGATATTGATCAATTGATCAAAGAATATAGTGTGCTATTTTTGCAACCAACGAGCTCCTACAGTGGTTCAGGTTCGTCTTATATGTGTTAATGCATATTCTTTTAGCTACTCTTCAAAATCCTTACTACAACCAGATGATGTGTTCCATGGTCTGCTCTAGCAATTTTAGTAGTTTCTACAGTAATTTCTGTACCTAATGTGCACTTTAGTCCCCAACTTAAATATATATGCTCCTCCGTACCTAATGTGCAGTGTAATCATGTATCTAGAGAATACAGTGAATAGGATTGCCCTTTAATTTCATGGTCTGAACTTTCAGAGTCGCCAGCGATCCAGCGCCCAGGCAACGAGGTGCCTACCACGGCAACCGAGAATCTCCATCACAGGCCCAAGGTAAAGGGATCAGGTAAGAGCAGATGCAAATCAAATCAGCTTGCAGCATGGTTTCTTGGTTTCCTCGATGGCTCCACTTAGCGACCAAGAGATCTGTGATTTCATTTGTGCAGTTCTGCCGCGTTCTTCTAACCGCTCAGCGCCCTTTTTTCGTTAGGTGAGGATGCACAATGAGAGAATGGGAGGATGGGGGATGCACAAGCGTCAGTCCCTGTGTATGATAAGGTGAACTTCCTTGGCTCATGCTTCAGATGATGCCTTGGTTAACTTTGAGAACGAGGATTCCTATGTGCAGGTTCCAGTTTTCGAAAACAGAATGGTAGTGGAGGATTTCACACCAGCAATGTGAACACCCTGAAATGGAAGGTGTGTGCCTGTCTCCATTAATTTGTGTTTACATATTGGTTCTTTATTCGTGGCCTCGGCTCTCCATGAGCAGCCATAGAGTCCATGGCAGCAAAATTTATTTTCATTGTGGGATGAGAAATCGATTGCTTTCATCAATACCTTTCATATTGCTTCCGATGGAAAATAAAAATAGCTCTCGTCAGTTAACTTTGATTCCACTCTTCCTATTGTTTCAGATAGGTCAAAGGAATGATATACTAATATGTATATGTGGTTCAAAAAGAAAAAAGGAAAGAAAAAAAAATTGTTTCCGTCGCTCATTACATTATTTCAGTCCCCTCTCCCCTCCCTTACAATGTGAATGTGTGATTCCTACTTCCACATGCATCTGACATGATGATGAAAAAGATTGTTTCTGTTTCACAAAACCAGGTGAGCAGTCCTGCGATCCTACTTACTACTCTTGTTTCATTACACAAAGGAAAAAGATTAACTTCTACTTAAAGTTCCTTAATTTTACGTGTTCTTTACAGATACAGAGCCAAAAAGTTTTTTTTGTATCATACATCATTTGCAGCCCCAGGCCCAAAGAACCTAAACACCGAGGAAAGGGCAGCAACAACAGGACTATCAAACCAGCAGCAGAGGTAACCCAAAGATCCACTCATGTTTCCTACACTATCACATTGCTTTTCTCCTCCTTAAACTTCGTGTTACACAGTGTCATAGTAGCCAGCTTGTTTACATGTTACAAAGCTTGCATATTTAGAGCCAATGACCTGTGCTGCATTATTCCCACAACAGAGTACCCACATAGCCACTCTTTTCATGTCTTCAGTCCCCTTACCCAGCTTCAAGTTTCTTGCTCTATTAACAGGTGTCCCAGCTGCCTACCCGCTCTTTTCATCTCTTAAGTCCCCCTACCAGCTTCAAAGTTTCCTACCCTATTAACAAGTGTCCCAGCTGCATTATATATCTCCCTACGATACTACCCGCTTTCCTAATATCAAAATAATCATCTTTTTAATGCTAAGAACATGAATCATAGTATAAAAAATAACCCTCTTTCTAATGCTACAGATATGGATCAACAGAACCCAAGCAGCTGCCAGTCGACCTCGACGTGTCAAACCTATGTATATAGCACCACCTCCAGGACAGAACATGATTTATGAACTTTTAGATGTCGTCACTCCTGTGTACACTACATGCTTATCTACACGCTTTAGAATAATTCTCCTTAACCCTGTCTATTTTCTCTCCGTATCATAAATGTAAGTACGGATCTTTTACTCAAGTATCGCTTTAATCCATCATGCCCCGTGTTACTGTCTATCGTATAGTCGTCATGATGGCCATTAACTGAAAACGTGCAGGCCCACCTTGTTCCGTCTTGTTTACATTTGGTCCGTGCCTATTACATGCTGCGATTTTTAAATGCAAATTAGGACATGCTGCTCATGAAACCTACAACTGCTTCTAATGATGCCAACTTCAAAATGCATTATCAGCCATCAGCCTGATGTATCTCATGAAATTAAATATGGCTTTGCCCTATGCTATCTTGAAATCCATTATTATGTATAAACTGCCCTGTAGCTATCAGCGCGCGAGGGCGCGCGCCTTTCACTAGTTCCTATATATATGCCAGCATATGATTTTGTAGAGTGGACTACTACACTTAATTTGTTAATAATTTTTTTTTCTGTATCTTCCCCACCAATAACGCAGTTTGACACTTAATATATATAATATCTCATATGGTCCTTCACCTGTTCAATTATTGCCACTTAATCTTAATACCAAATTATTGTGCTAACCAAGAAAAATCATAGTGTCCACTTTGCTGAGCTGGTCATACCTCCATGCCACTGCGGCACCGAAAGAAAGAAAAAGGAAATAAAGGCTGCTACTTGTGTCCACTTTGCTGAGCCTGAATACCTGATCAGAAAATAACAAAAACAGTCATGGACACATGAGTTTTACGGAATGAATGTTATTAGTGTGTCTGTTTTTTTTTCTTAAAAATCTTTTATAAAGCTGTGTGCATCATGGTTATGCAGAGGATGGGAGTGTTTCAAGGTTTTTGTATCATCTCGATGTAACGATCTTGAAATTAATAAAGCTTCCCTTATCGAAAAAATGAGTTTTTATGTGTTAGTTTTATTTAGCACAACTGAGCAAGACTAATTTCACCTGAGATGGTTAAATGTATACCATGTGTTCTGAATAAAGTTTATGGGTTCAAAAACTTAAAATCCTAATCGATGCATGCATGCGTGGTCCTTGAATTGAGCAGAAGGTTTGATAGAGGCATTATTGGTGGACCATCAATACAATGCATGCATGTCTTCCTGGCCAAGGTGGATTATGAGCATGGTTGTGCAGATCAAGATCTTCTCTTATGAGCTAAGAATTGCTATCATACTGGAACCCAAGTATGTTACAACAAATCAGCTGGGAAGTATGTGCAGTGAAATGAGGTAGAGATTCATCTGAAAAAGATGAAGTAGAAACACTGAAAAAGCAGAGGAAATCCTGAAGTGAGCAGATCATCGACGTGCAGGCAGGCACTTGTTGGGTGGAAAGCTGCCTTCTCTCAAGACACACAGCTGCAGCTGTCATTTGTTTTCTTTTCTGGGGTCTGATCCAGCCACTTTCAGTGAAAAACTGTTATACTAATTCTGGACTGGAAAATGGGAGGCACAAAACAACAAGATCCAAAAGTTTTTGCTTGTGTAACTATTTGTATATTTGTCATGTCTTGGATCTTCCTTTGCCGATCATGGATGAATGTAGTATGGCCTCTGGCCTGTGACTGTACCAACTCATCATCAATCATGTATCCATCCGCTTCATCTGCATGTTCTGCTCCATCTGCATGCCAGCCAACATGGCTGGTGATTAATTCCTCACCAACGAACTGAATGGCAGGTGGAGCATGGCCAATGAAAATGCTGCTCACTGAAAGGAATTCACATGGTTTAATGCAGCGTTTTCTTTTGTTTCAGAACGATGGCAATCACGCTGTCCGCTAGACTATGCTGCAAATAAAACGTAGCCCTCCTGATTACCTTGTTGATTTGTGAAGATTGTGACGAATAGAAGGTCAAATAAGCTTATCACACGACTCCAATTGCTGATCAGCTCAACAATGACCTATGGCAGGTAAGGTGTGTGAATTTAGTTCACCACGTAAACAGTATATATATATATATACACACACACACCTCTTTACAAGGTTCTTTGAAAAATGTTTGCTGAAAACCCAGCATAGATGGTAAAGTCACACGTGTCAAGCTAAGTCGGATGCTGATGCATATGACTTTGTGGCATTATGAAAATTGCTGGATTTGGGCGATGATTGTTCACACAGACTGACCACAGGAGATTCAGGCCTGGAGGGGACAAGGTGGTGGATTGGTGGTGTTCATCTGGTGAGTTTCTTCAACCACGGCCAACACATTTTCAGCATAAAAGAGGGCAGGGTGATGTTGTTCATACGTCTTCTATTGTAAGCAAATAAAGATCGAACACAAAACGCAGATTTTTTACGTGGAAAACCCTTGCGGGAAAAACCACGAGCGCCAACCAGCGATCTTCACTGTATGATGAGAGTTTACAAAACGCAGAGAATACAATAAGCTGTCTGCTCTTCCTGTGTGGCTTACAAGATGTATATATATATATATAGGACAGATGGACTGCAGTCCAATACAAAAACAAATCCGCTAAACACATAACCCGAACCGCGGGCCCTCCAGGCGTCCCCGCAAGACTCGCGTTAGAATTCAGATCATATTCCAACATATGTGCCCTCATCATCCAATAAGGCATTGTCCTAGGTGGCGAATTGATGAAAATCTACTGTCTCACCTAGGAAGCAGGAAGCAGTTTTCACAGGCATATGTTGAGTTTTCCTGTGCCAGCCTTGATTTATAGGCCAGCAATGTCTCCAATTGCATGCAAATCTGTTTCAGCAATGGGTTGTTCAGTGAGACTATTTCAGTATAACATGGAGAAAATAAGTTATATTCATCGGTTCACAAGAACAACACCCTGTACTATATACCATAGTTCAATGGATGCAGTGATACTCTTTACATGTTGTTAAGAACAAGATGGCAGTACCAATAGTTTATAGCTAATATGAAACGAGGTACTGGACTCAAACTTCTCATCCCCATATAGTATAGTTTCATATGAATCTAAATTTATGTAATGCTCAATTTTCAAAAAAAAGCAGAGTACTGAAAATTCAACCCTTATGCAAGTGTTCAGAAATGAAAAGCATTAATAGCTACTTATATAATTTATTTAATTTCAAGATTCAGATGTAGTAATATTCATTCATCAGTTTGGTGATGAAGTATCACCTAGTGAATGTTCTTGATGAATTGAAAATGATGGTGTATGTAATTCACGAAAAGACATTAAACATGATAACAAACAAGTTTGATCAGAAAAAAGATTACCAAGTCATCATCTTCTTTAGTTTGTCCCCTTTCTGCTTTGTTTCTAGCAAGAGCTTGTTTTATACTTCTAGCTGAGCACATCTCCGCTTCGCAAAATCTGGATCGATTCAACAGATTTTTTGTTCATGGAGTAGAAGTTTTGCTTTAGCAGCCATTGCACTAGCAACCTAAAAGGATTAACAGGGGAAACGAGAAAGGGATCATTGCTGGTAGATACTACAAAAGAACAACTTATCTGAAGCATTCATCTAACGTCGCCGAAAAGACAAAATTTATGTTGCAAGGAAAAAACAGTTCTGCTAAGTCATTGTAAGCACAGAAAAGAGCTTACTTTGCTATTCCAATGGTCAAGATGCTTTTTTTAAAAACAAATCAGGCAGGAGCAATGCTTATGTTTTGATAATGCTACAAATCGTGAGAAAACATTAAGCAACTTCAGCTGGTACCCTGTCAATTCTCGAGTCTTGACTACAATACAACTCCGTAGATGGGTAAAGGAACTAGAAATAAATAGATTGGCAAAATAAAGGAACTCGAAATAAGTAGATCAACAAAGGAACTAGAAATATTTCTTAGTAGAAAATACAAACATCTGTGTGCTCTCCTGTATTATACTGAAAACTAATGACCCCATTTCATAAGACTAGAAAACATAAACTTTAACGTTGATCTGACAACAATGACCAGTAAATGTTTCTCTACATACAGGATGTATAATATCATATTTATATTCAAGATATAAGCATGTGGTACCTGACGTCGTGGAAGCATACAGTTCAGTCTCCTGGGCTGCTGATTCAGGTTAGTGGTGAGAGGTCAGGAGCGCTGGGTTTATCTACTGCTGAATTGGGCATATGAGAACTCGAACTGGCAGGTTTCCTTCTAATTTGAATTTTCTTTGTTTCCTCAACTCAATGATACTGGATGTCTTATTATCCACAATTGTGAGCCCTTCCTGTTAAACAATGGAAGCAACGGATCATCAATTTACAGTACAGATCAGGGTTATGCCATTTGTAGAAATAAAGAAACAAAAACAGATGTATAGAGTTAAGTAAAAACACATTACTTCCAACGCATTTTCTATTTTTCCATCCACTAAGCTGGTTGATAGAAGCAGAAAGGGCCTCAGTGGTTTTCAAATGATCAAAGGTCAGATTCTTGATGCCGCATCTGCTGCCATTTAGTGGTTTTCTGTTGGGCTGTGTGTGTGTATATATGTGTAATATGGCCCAAAGGCCCATGAACTAGGTATATATTTGTAAGCCTTGTAAAGAAACTGGATGAGTATTCCAGAACAATCATTTCTTCATGGTGTCGGAGCTGACCTAGGAGATTCCCTGCTCCTACCTGGTTGGCGTGGCAGAGGCTGGTGGCCGGCGGCGAGGTGCTGGGAAGAGCAGCAAGGCAGGAGCGGCAAGTGAAGCAGAGGGCAGCAGCAGTCTCGCTGGGCAGAGACGGGCCGTGCGAGGCTACAGGGGCGGAGCGCGAGTGGCAGGGCGCGACCGAGCATGCCAAGAAGAAGGGCGCTGCGTCCCTGAAGGTTGCAGACGGTGGCCGCACCGCAGAGAGAGCAGCTCACGGCCAGAGGAGGAGCAGCGGCAGGACTGCAGTTGAAGGTAATCCTGGGTGGATTTGGTTTTTTTTTTCCTCAGATTTGGGGGAAATAGAGGACAGCAGCGTGAGGTAGAAGAAGCAAGAAACTGCTGTTGTGCATGAATCAGTGAGGGAGAAGCCTGATTGTGCGTGAATCAGTGAGGGAGAAGCCTGCTTGTGCGTGCTTGCTGGTAGAAGCTACAAGATCATGGGGGAAAATCAAGGAATTGAGCAAGTTCTTGGAAAATTAGTGGAGTTACTCATGGCAAAAAAGGATGAGGCCCCAAGTTCTAGTAAGGAAATTATTGTGCATACAGAACCAGTCCAGAAGATAGAATTGATGCCAAATGATGTCAAATTAGAGGGCATAAAAAATTATTTGGCTTGGTCTAGAAGGGCACTTCGGTTATTGAAGGCGAAAAAACTTGAAGGCTTTATCAATGGAGAATCACCTGAGCCGTCAGACAAGTCAAGTTCTGAATGGAGTACATGGGATGCTACCAACTCTCTAGTGGCTGCGTGGTTGTTGAACTCTATGACTCCGGCCATTGCCAACGCAGTTGATACAATTATGAGTGCAAAGGAGATTTGGAAAACAGTGGAAAAGATGTATTCAGGTGTTGGAAATGTGATGTTGATGGTTGAAATAGAAGATCGACTCCACGATCTCAAACAGGGGGAGCGGTCAGTGATGGAATATGTTGCTGAGCTGAAGAGTTTATGGGCTGATGCAGATCATTATAAGCCTATCGAGTTGCCACACTCAGATTGTGTGGCATGGGTGAAGAAGTGGATAGAAGAAAAGAGAGTCATTCATTTCTTAAGGGGGCTGAACTCAGAGTTTGAGCCAAGGCGTTCTGCTATATTTCATCAGGCCACTCTTCCTAGCCTAGATGAGGCCATAGCTGCTATTTCACAAGAGGAGTATAGACTAAAGGTGATGAGGGAGACTGCTACATCTTCATCTCCTACATCTTTATCTCGTCCTGTTTTTGCAGCAACAGGAATTAAGGAGGACAGGAAGTGCTTCAATTGTGGTGACACTGGACACTTGATTCGTGATTGTCCTAAGCCACTTAGGACCAACAGTGTAAGAGGAAGAGGTAGCAGAGGTGTTATAAGGGGTAGTAGAGGACGTGGAGGCAGAGGTGGCTATAGGGCCAATGTTGTGGGCACAGGAGAGGAGTTTCCAAGAGTAGAGGCTTCATCAGTGATAGTTGAAGAACCCAGACAATGGAAAGAAAAAGTTGAGAACTCAAAAGATAAGGATCAAGGTAATTTTGTTGGTGATTTCGTCAACTTCGCCTACATAGATGAAGGTAATTATGCCCATGCATCAATACCTATGCACACATCACAGTTAGATTGGATTTTAGATTCAGGGGCATCAAAGCATGTCACAGGTGCAGCATGTGAGTTTGAATCATATATGCAATATCCCCCCACACGTAAAGAAACTATACAAACTGCTGATGGTACATCACAATTCATTAAAGGTGTTGGAACAATTCAGTGCACTCCATCTATTAAATTGTCATCAGTTTTGCATGTGCCAGCTTTTCCGGTCAATTTAGTATCATTGAGCGCTTTGGTTGATCAATTGGATTGTCGAGTTACACTTGACCGGGAGAATTGTTTAATTCAGGAAAGGAGGACAGGGAAGAGTCTTGGGACTGCAACCAGGCGTAGTGGGCTGTGGTATATTGATCGTGAGGGGACAAATGAGGCATTATGTACTGTGCTGGCTGCAACTACAGGAGAGAAGGAAGCTAGTGTGATGCTATTGCATTGTAGATTGGGGCATCTTTCTTTTAGTAAAATGTGTAAAGCCTTTCCTAATGTAATGTGTGGGGTGGATAAGAACAAACTATTTTGCGATGCTTGTGAGTTTGCAAAGCACACAAGAACATCTTATTTGAGTAAGGGCATTAGAAGTGTGTCCCCTTTTGTGCTAGTACATTCAGATGTTTGGACATGTCCTGTGGTTTCTATAAGCGGAATGAAGTACTTTGTCACTTTCATTGACTGCTTCTCTCGGATGACATGGATATATCTCATAAAACATAAAGATGAAGTGTTGAGATGCTTTCAGGATTTTTGTGCCCTTGTGAAGAATCAATTCAATAAACATGTTCAAATAATAAGGACAGATAATGGAACTGAGTACGTGAACAAGGAATTTAGTGCATTCTTGTCGGCCCATGGTATATTACATCAAACATCATGCCCAGATACTCCTCCACAAAATGGTGTAGCTGAAAGGAAGAACCGTCATATTTTGGAGGTTGCTCGATCATTAATGTTTACAATGAATGTGCCTAAATTTTTGTGGAGTGAAGCTGTTTTAACAGCTACATATCTTATTAATCGTACCCCATCAAGGATACTCGGGATGAAGACTCCGTGTGAAATGTTAATTGGTGAGAACACTTTTGTGGTTCCTCCTAAGGTTTTTGGATGCACATGTTTTGTTAGAGATCATAGACCTTCAGTGAATAAATTAGATCCACGTGCAGTAAAATGCATTTTTGTGGGGTACTCCTCAGGACAGAAGGGTTACAAATGTTGGAGCCCAAATGAGAGACGTCTATTTGTAAGCATGGATGTGACATTTAGGGAGTCAGTGCCATATTATGGCGAGAAGACTGACCTAAGTTTTATGTTTAAACCTCAACTTGTGGAGTCAGAGGAAGTTATTTGTGAGGGGGAGAACAATGATGTGGAAAGTAATTCAGTTGAGAAACTAACGAGTATAGAAGGAGT

>BAC-69

ATATTTAATACTTCATGCATGTGTCAAAAGATTCGATGTGACGAGGAATCTGAAAAATTTTGCAAAATTTTTTGGAAACTAAACAGAGCCTAAAAATAAGAGGAAAACTAAGGAATGTTGCAGATGGGCACCGTTCCCAAATCCTAGAGCTCGCTGCAGTCGGTGGGCTCAAAGGACAAGACGCCCCTCCCGACGTCGTACAGGACGTGCATGTTCTGCTGCTGCATGCTTCCGAGCACCGACATCCCCCGGGCGCTCACCATCCCCAGGCACGCGACCCCGGCGACGCTGTCCTCCACCACAGCGCTGCTCCGCGGCAGCTCCATGTCGGCGCCGTCGAAGTGGAGAACCAGCCGCGGCACGGCCACGGCCTCGGGCCCCCGCCCCTCGGGCGCCGCGAAGCAGACGCTGAGGCCCAGGTGCGCGCCTCTGGCCAGCGGCAGCGCCACGCGGGCGGCGACCGCTCGCGCCAGCACCACGAACGCGCGCTCCTCTAACGCCGTGAACGTCGTGCCGGAGTCGATGATGAGCCCGCCGTGGCCCGACGCGGTGAGCCGGAACACGGCAGGGTCGATCGGCAGCAGGGTGTCCCCCACGGTGATCCCTTCCAGGGAGAGGTAGTAGTAGGAGCTCCGCCGCGGGCCAGTCGGGTTCGGCACGAACGGCGTGGACTTGGCCGCCGGCGAGAGGCTCGCTGACGAGCCGAGGAACAGAGGACTCGACGTTGTCGTGTCGTTGAATGGCGTGAAGCAGTAGGAGAACCTGGTGACGCCGAGCTGGGACACCAGCGACAGCGGGCCCCGGCCCATGCCGACCAGGCCGGAGGAATTGTCCGTGCCGCCGAGGTTGTCCGTGCCACACCCGAAGGCGAGGCCGTGGACGGTGGTGCCCGCGCCGAACGTGAACGCCTCAGTGGCGAGGACGCCATCGGTGGAGCTGCCGTCGCCGTAGGAGTAGTAGTACGCGCAGCCCCGCACCTGCGCCGACGCCGACGCCGAGCAGCGGGAGGAGGGCCTGAGGCTCGGGAGCGCCTCGCAGAGCCTGGAGCCGCACGACACGTTGGCGTAGGTCGCGGACCTGGCCGGCGCGTACAGCGGCGCGGGCTGCGGGAAGCACCGGCGGCACGGCGCGTCGCACTGCGTCCAGATGAGGTCGCTGCCCGTGTCGAGGACGGCCGAGAGCGCGAGCGGCGGCGTGCCGATGGCGAGGTCGACGAGGTAGGTCGCCGTGCTCGCGTGCACGGATGCCGCCGCCGTGGCGGCAGCGCCACCACCACCACCGTCGCTCCGCAGCGTGGACGTGGACGCAGTGGGCGGCGAGACCGCGGCGAGGAGGCCGTTCACGCGGCGGTGGCTCCGGTCAGCGGCGCGGCGGACGCGGTCGGGCCCCGTGAGGTCGCCGCGGGCGTCGACGTGGGTCAGCTCAAGGCGGATGCCACGGCTTGGCTGGCCGTGGACGGGGACGGCAAAAGACGGCAGCAAGAGGAGCAGACAGAGGAGAAGCGATCGCTTGACGGCCATTACAGACGGTACGGAGGGCTCGGTCGTACTACCTTGTTCCTCGTTTCAGGATGCCGTTTGGAGAATTGGAGTGATTCTTGATGTTGATGAACTTGGTCTGGAGGTCAGATCTTTGAAGTATTTGAATGATTGAATGGTGGCTGCCAAGTGCGTTGCACGTCTTTGCAACGTAGAAGTTTATAAATCTCGTTCATCTCCATTTCGCCCTTTAGATTCTTTAATGGAAGTGACAAGTGATCACGACACTTGCTGAACTTGAGTCACGGGCTCCAGGCGCGGCGGCGGCGGCGACGAAGACGACGACGACGAGAGCTCCTGGACAGTGAGTCGCCGCCTCGCCGGTGCTTGCGTGCCCCTGGTACGTGCGACCTGAATAGCGTGGATCTGTCGTGCGTCCTTGCCCAACTGATTGGCCCATCTTAAGAAACCAAACCTTCGTGAGCCCAATTAACAATGATTCACATTCCACAGCCCACCTGTTTTGGCCCATCACGGTCGGTTTTTTTTTTAGCGTCGCTCTGCATTCTGCTACTGCAAGGCAAGTAGGCAACCGTATATTTCCTCTCCCTGCAGAGAGAAATCAGTGTATCGTCTCCGCCTCTCCGGCCGTGGGACGGGACGGGAGCCAAGTAGCCAACGCCGTCTTCCTCCGGTCGTCCCACGTCCGGCCCGAGACGCTCCGCCGCTGCAACCATCGACAAAAATGTGTGAGTTCATGCATGTCTTCCATCGATCACTCTCTTTTAATCTTGCAACCACGTACGTATGCGCTGTTTCTGCCTTTCACTGTAGATTCGCGTGCTACGTTGTCTTGCTTGTCTTTGTCTATCTGGTTGATGCATGCTAATCTGTTTCAATTTAGATCCATACTGATTTTTTAATAGATAGATCCATACTGATTAACTGTGTCTTTTGATTCTACTCTTGAAATTAACTGTACGGCTCCAGAGTATCATCACATTTTCCTTACTAGGATGAATCGTCTTGTGAAAATACTGTTAGTAGTAGTTTAGTCTGTGGAGTTGTGGTACCCGAGCACCTTATTAGGCGTCGAATGTGAGTATTATGCACAAAGTAGGTTTTCAATCAGTTCATGTAGACATAGCGGTTCATGTGTCTGCATTCTGAATTAATTGGTATTTTCCTAATAAATATTTCATTTTACTTTTTCATGGATACATTTTTTTAAATGGCATCTCCTCCTTTGATAAAATACTAAAGGTGTTCGACGAATGAAAATCGGACGACCACTATCAAAACCGAATCAAAGCTGCGGGAAGTAAAAATAACTGGTTTTGTAGAACAACCATGATTAGTTTTTTTAAGAAGAACAACCACGATTGGTTAAAGGATGTAAATTAGATTTTTTACTAGTTATTAGAATGGCAAGAAGGTCCAGTAATAAGACATCCAACTTGGGGACATTTAACTATTTGCCACTCTCGGGCACGGTCAATTAATTCCTCTCACATCCACTTCTCTCACGTGGGTGGCAAATCTGTTGCCACTCTCCCCTAAAAGTGGCAATGGGTTAAATATTCCCCCAACCTTGGGCCAACCTTCTCCGAGGCGCTAGGCCTGCAGCCCTGCTCTTCTCTCGGTCAATCCCTCTCACAATCCGCCCGTCGACGCCTGACCCCGAGCGGACCCCAAAACCTAGCGATTAGGCGGCTAGCGAACCAGCGAGCCGCGACGGTCCTTTCCGCTTGCGCTCGGCGCCGCGGCGGCGGCGGCTTCGGCAGGGGCGCGGTAGACGCCAGCCGGCTGTAGCGGAGGGGAGATAAGGTGCAGCTTGCAGCGGCCAGCAGCCTGGCTCGTCGGCCGGTAGGCGCCTAGGCGGCAGCCGTCCTAGGCATGCGGCATGCCCGACAGGGGCACAACCACACAAACTGCTACAGGTCAAGTTACGCGCGCGGCGCGCTTTAGTATTTTATATTTGTTGCCATTCGTTGTTGCTTCTGTACTCAAAAGAATGCACATAAACTTGGTCTTTACTTAATCACATTCCATGGAGTTGATTTCCATAGCCCTGAATTTAGTAAATAGTTTATACTATTTTCTGCGAGAATGCCAAGTTAGCCAACTTTAGTTAAAAATTCTAATTAGCAAATCTCTGATTAACTTAACTGTAGAACTCATAATCAACAACCGTCAATTTGTGTCACTGGCTACAGAATACAGAAATTAGGGTTCTTTTTTTCCTCCTTTTCTTTTGGAAATTGGAAATAAAGGCTGAACGAATGTTATGCATTATACATCTTTGTGAGGTTCAGTGAATTATTATGGAAGTTCTACAATGCACTAATAATTAACCATTATCCATATATGTGCTGTTTTGTGTAAATAGCAAAATGTGAACTGGAGATTGTTTCAGAAAATATGGTTCCATGACTTTGATCTGCTGTTTCAAGTATCAGCCGTGTTTCAAAGTACGTGATATAATGAATCTAGATTGGTTTGTTACCCTGTTATATATGCTCAGCCCCTCCAACTGTTTTTTTTTCTAGACCTGCCACTGCGTGGAATGGAATGGATTGATTTATCACCATTAATTTCTTAAAATCTAATAATTAGTTTATGCATGAGGAATGAGTCATTCCACTAAAATTCATAGGATGATGTCATTATGCACCATTTCATAACAACACAGCCTAAATATCTCGTTATGTACCGTAGGAATATGTCTAGCGTGGTGCAACCAGACTTTCCCAGGCTCATACCATTTGGTGCCCGAGGAGACTATGTCACATGGGCCAGAAATGTTGAGAATTATTTAGCCCAGAATAATCTCAGCGATACAATTGTCTCAGGCTCCAATTGCAGTAAGCAGCAGAAGGCACAGGCTCTGTTTTACATCCGCCGCCATCTGAACGAAGATTTCAAAAACGACTATTATATGCTCGAACGGGATCCACTTGTTATATGGCAATCTTTGAAGGATCACTTTGATAGGATGAAGGCGGTAGAGCTACCACAAGCGAAGTTGGACTGGGACCGCCTGCATTTCAGCAATTTTGACTCTGTGACTGCATATGATTCGGCTCTACGGCGCATAGTCTCTCAACTGAAGCTCTGTGACAAAGAAATCACAGATGAAGAAATGATCGAGAAGACTTTATCTACCTTCCCGCCAAGTCAGCGTATCCGCCATCAGATGCATAGGATGGCAAACTATGGCAAATACTCGGAGCTGATTTTCGCGTTATTGCAGGAGGAGAGACAGGAGTTGAGAGTCCGTCAGGAGCTTATGAGAAATCAAAATGTGAAACCAGCCCAGAGGGTGTCTTTCAAAAGGAAGAGAAGACGGGGTCAGAGTTGGAGGTTGAATTGAGTGGCATAGCGTCGCTTAGCACGGGACCTATGACAGCAATAAGGTTGCAGATTAGGGAGGTTAAGAATTGTATTATATCGTTTGGTCGTCTGTCCTTGTCAGACCTATGTATTATTCAGTCATCGATTTCATGTTTGCTTGTTGAACCCCATGTATCGAGTGTCTCATTCATGTAAGTGTTAAGCCACTTCTGGAATGCTCAGTTAACAATTCTGAACAAGTCTCAGTTTTCAATTATTGAATGCCGGATCAATGTATGCTACTTCACTCATCGATTCCATCTTTGCTTGTCAAAACCCATATATCAATTGTCTCGTTCATGTATTTTTCAAGCCACATTTGGACTATTCAGTTAAGAATTCTGAACAAATTCCAGTTTCCAAAAATGTAATTAGAGTGTTTGGTTAACAGTACACTGTATAAAAAAAATCATGAATCCTATGCACACATTTATATTAGTAACTGTATATATATACAACTATGGTATCGTCAAAGAGCGCTGCACTTGGCCGGAGCAAACGACAGCATCTCCTTCCGGACGTCGTAGAGGATGTGCATGTTCTGCTGCTGGTAGTTTCCGAACGTGCTCATGGCGCCGTCCGTCTGGTTCCGCATGGCCAGGCACCAGACGCCGGAGCCCGAGATCATGTAGCTGTCCGCGGGGAGCACCATGTCCGCGCCGTCAAAGTGGAGCGTCATGCTCGGCATGGCCGGTGGCGCCGACGTCGGGGTCGGCAGCGCGTAGCACAGGTCGAGCCCCGTGGAGTCAGACCCATCGATGGTGGGCAGCGTCACTAGGGACTGCACTGCGGCGCGGACCTGCTGGTACGCGGCGTTGACCAGCGAGGTGATCGTCGTGCCGGAGTCGATGATGAGGCCGCCTGTGCCGTCGGCTTTCAGGGAGAAGGCGTTTGGCGAGATGGACAGCGCCTTCTTGCCGAGGGATATGCCCGTCAAGTTGAGGTAGTAGTAGGTGCTCATGGGCGCCTTGGCCGGGCTAGCGACGAACGGCGTGGAGCGGACGCCGGTGCCGTTGAGCACCGCCGACGGGCCGAGGAGGAGCGTGCTGGTGCTGTTAGTGTCCTGGAACGGCGTGAGGCAGTAGGAAAACCTGCCAGCGCCGAGCTGCGAGACGAGCGACAGGTCGCCCCTGCCCAGCCCGACGAGCCCCGCCGAGCCGTTCCAGTCGCTGCTGCTGGCGTTGCTACAGCCGAAGGCGATGCCGGGGACGCGGGCCTGGTCGGCGGCCGCCGAGCCGAAGGTGAAGGTCTCGGAGCCCTGCACGCCCGCCGTCCACCCGGTGCCGTAAGTCTGGTTGTACATGCAGGCGCACCCGGGCGGCGGCGCCGCCCCCGCCAGCGCCCCCGCGCACATGCTCAGGGAGCTGTTGCACGGGAGCATGCCGAACGTGGTGGAGCTCGCCGGGTTGTACAGCGGCGCCGGCTGCGCGAAGCACTGGGCGCCGCAGGGAGCGCACTGCGTCCAGATGAGGTCGCTGCCCGTGTCGGCGATGGCCGGGTACGACAGCGGCGGCGTGCCGATGGACAGCGTCATCAGGTACTCCCCGCCGTTGGGCAGGTCCTTGCGGGTGCGCGCGGACACCGTGGTGCCGTCAGACTCCGCGAGCTCGCGGCCGAACACCCGGGACTGCTGCCGGTGCATGTCGCGGCGCAGTGCGTCGCGCACGAACTCGGGCGCGGTAATGTCGGGGTCGGAGTGGATGCGCGTGAGCCCGACGCGGACGCTGGCGGCGCCGGAGGCAAGCGTCGCGCAGACCACCAGGAAGACGAGCACGGCGAGCGACGCCATTTGTGAAGTCGATGACGACATGGATCTAGTGATGACAAGCTATGGTGGCTGGCTGGAATGATTATGCAAGAATGGAGGAATGGCATTGAGAAATGTGAGCTTCATATAAGGTGATCATGGATTGCCTGTGGCTTGCTTGCTCTAAACTAGCCTCTAATACCTGGTCAGAGCCGCCATCTTGTGCCGGTCTCAGCTGGTGAGTTGCCGGGAAGAGCTGAAACAGTCTGCAGCGCTAGCAATTGTTGTCGTGCTCACGCCAAGAAGGTGTCGCCTACGGTTGTATGGGCGCTGAATTTTTAAGCGATTAGGTGACCTATTTGTTCGGCGTGCGGGCAATAAGTGTTCAAACATGGTTCTACTAAATTGAACGGGGCCTGAGGTCGTTGGATAATTGTTAGGCACTTTTTGAGGAAGCAGCAGAAGCAGCCGTCGTGGTCCAAAAGCAGGTCAGATGCAGGAAATTGACTGAATCAGAAGAACGCAGGCGAAGAAGAACCGTGTGCAGGTATCGACTGCAAATATTTCCACCTCTGTTGGTACTAAATGAGATCATCAGCAGTAATCGGATGGAGAAAAAAATTGGGGTGCAATATAGTAAAGTAGGATAATTGTTTATTTCTTCAAAGAAAAAACAGCCGTTTCGGTTTAGTGATTTTTTTATTTATTTCTAACCCATGTAAATGTACTACTACTACGGTACTACCTTATCCTTCTCCAAACATTACCATTGACCCTCTTTTTTCATGTTATGGCACGTGTGCTGTGACCACAGGCTGTCCGCTGTCATGCTGAGGCCAGGCGTGACAAAAGTAGGGTAGGCGTGACAAATTGAGTTCTATCTCTGAATTAGTTGATCTAAAAAAATCTAATGCAACCACATTAGTTGTTCCTGGTGATCTCAACCTAATTTGCTGAGCCAAGGATAAAATTAATGGACGTATCAACATGCAAATGATACATGAGAAACTTCCTGTTAACGTATAACGGAATGGCACGAAGTCTTTTCGATCCCAATACCGATCTACAGGTGATTGCTTATTTGAGTTCTACCACTGCCTATGCTTGGCGACAGAATATTACTTTTATGGAGGCTTCAGATTAAAACGAGGAGATGGAACTATGACACTACCTTAAACAAGATCGTCCCTTGTATTGGGATCAATAGAATGGCCGAGAGCGCACCAAGATCCAAGAATAAATAACATGGATACGCAAGGGTAACACAAACTTCCTTTTTTTCAATTACACGGCAATGCCAGATGGGGAAAAAACATTACATAAGAGGCTAGTGGTGGAGGGAGTGGGAACCCTAAGTCAGTGTAATTTTCATTGGAAAATTTAGTTTTGATCAATTTTTTTTATACAATTTACATTGGACCCTTGTCAACTATATGGTCCCTGGCTATCCTTACCGAAGATAAAAACCAATGCCATGGAAGGGAGTGGGGCGGATCCAACTGATAGGAGGAAAAATTAAAACAGATAAGGGGTATACATAGAAACCACTGTCTATTGTAACCAACTTGTAACTCAATTGGGGCATCATTCTATTTGTAACCCTATCCCCGAACTATACTATCTCTCTAATCTAATGCACATGATTCGTTGATTACTCATCATCGTCAGAGGTTAAAAATATCGACAATTGGCACACCAGGTAGGAGAACATTGGGTGCAGATTCGTTTAAATAAGTCGGATGGGTTCTTCGACAATGGCATTGACGACTTAGCTCTAGGCAACACCATCACCTTCGGCATAGTATGGACTTCACCGCCGACTTGTTGGGGGTAACTTTGCATTGCCCCCACCTTTGTCAGCATCTAGGCTTTCCAATTCAGAAGCCTAGATTTCATGGTAGATGGATTTGCTAAACTCCACCTCTATGATTCATATTTGATTTTTGTTGGCAAAGGAATTGCCACATGCCCTTCCTGAGTACCCTGTCGGGCTCAGGAACAGGGCCCCGCACCATCCAAGATGAGCTTCTCCAGATGCATCTACGCTTTGTCCCTAACTTCCATTCCATGTTTGTTGGCATGATTAGCCCGATGGCGGACGACATCACCGAGTTCGACATCGCTAATTTTAACCATTTTGGGATCCTCACTAGGTTCTTCAACTACTCCACATGTAGTTCTGCATCTGATAATGAAAGCATGACAAGTACCAACCATCTCAGGAGTGCTACATGGCGGACACGGGACACCCCACAACGACTAGAGGGGCCTCAAGAGTACCCCGTCAGGCTCAGGAATACGGCCCCACACCATCCAAGATGAGCTTCTCCATAAGATGCATCTACACTTTGTCCCTGGCTTCCACTCCATGTTTGTTGGCATGATTAGCCTAATGGCGGATGACATCACCGAGTTCAACATCGCTGATCTTACCCACTTCCGGATCCTTAGCAATTTCTTCGGCTACTCCACATGTAGTTCTGCCTAAGAATGAAAGCATGAGAAGCTACCAGACGTCTCAGGAGTGATACATGGCGGACACCCCCACAATGACTGGAGGGCCCTCAAGAATACCACGAGTACCTCCATGAACAATTCAAATGCCTTAGGGATCAACAAGCGTACCTCGAAATGGAGAGGCGCCAACTTGTTGATGAACAAGCAAGAAATAGATTATCATAGTTTTGCTTAGGGTGAAGGTTATCGAACTTTACTGTGCATAGAGATGGGAATATGTTTGAATGGGCACTACTCAAGATAAGAAGGTGCATTGATGTGGATTTACAATGGTCACGCCACTGTTGTAGAGAACGCCGCCGAGGAACATCTTATCGAGAGGGATGATGCAAGAGGGTTCTAGAGAAAAGGTTATGACCAAGACATCCAAGGATGGTGATATGTTGGTTAGGCTCATGTCACGAGCTCAGTAGTGTTGGAGTTTGACCGAATTAATAGGACAGGCTAGTCCATGTAACTGAGCTCTAAACACCCCTGATAAACAGTACCAAAACTATTATTTACTTAATGGAGATTAAGTTGAGTAAGGAGGGCCATGGTCCATCTTGGCCTCAACCAAGCTCCACACCTGGGCTTGGGCTTCTCGGGCTGACCTAAGGTAGAAGGCATGTTTTACAAGGATTTAACAAAAAGTTTAATAAGATTGGATTTTTTGGGTCATTACACATGTATTACTTTATATACAATTTTATATTTCAGCTAAAACATTATGACATATTTATATTGATAGTATTGTTATACTATTTTCTTTTATAAAAAAGTACCACTACACAAATTCTTGATAAGACAATTCAATATCTTGAGCCACGAAGGAGGTTAGTGGG

>BAC-71

CCCTCTCTCTCTCCCCACGACGTCTCTCCGGTGGTGCGGGCGGGCGGCCAGCGAGGGCGGCCGGGCGAACCGGGCGCGGCTTGGCGGGCGGGACGGTCGCGGGCCTCCTCCGCCGGCCGCACGCCATCCCCTTCTCTTCCCCTCCGATGGCCGGGCGCGGCGCGGGCGAGCCAGCCGAGCGCGGCTCGGACGAGGCGGGACGGTCGCGGGCCTCCTCCGCCGGCCGGGCGCAACGCGGGCCTCCTCCACCGGCGGCGCGCCATCTCCTTCTCTTCCCCTCCGACGGCGGACCTTCTCTCTCCTTCCCACATGACGAGCGGAGGACGAGGCCGCGCGGGCCCCGCGCCACGCAGGCGCGTAGCGGAAGCCCTGCTCATCCGCCAGGAGTAGTGGACGCCCGCTCGTCCGCAACGATAGCGTGCGGGGTAGCGTACCCCGCTGTAGCCATTTCATCCGCTACACGCGTTTAGCGGATGACTTTACCGGACGCAGTGCGGACAGTGGCCCTGTTCATCTGGCTTATAATCCGTCTTATTCAGCTTGTTTTTCCAGCCGAACGGGGCCAGTCTTGCTTCCTCTGTTCCTGCATGTTCTCTCTCTTGCTCTCCTTTCCAGACTTCTCTACGGACAGCCGCTCCTCTCGACTAGTACTAGTGTACTACACCTCCTCCCACCCATTTGTTTTTTTATTTCTTTATTTGTTTGGTGGCCAACAGGCAGACGGTGGATTGACGGGTGTTGCACCATGCTATAGACCCTAGGAGCTAAAACGTCTTACTTTTTTATATAATTTTTTAATACTCTGACGTCGTTAATTCTGGGACAGAGGAAGAGTACACTCTAAAAAGTCAATCTTGCTCTCTATCTTTGAGAAATCCATAATAGAGAACGGCAATATTTAGATATCTAATTCAAGAAGTTGTTGGACGATATTTTTACTAAAATCTCTGTTTCTTCAATAAGGAAAGATATAAAGAGGTTCTTAGGGTTGGTTATGTCCCCTTGAACTAGAAAACCGGTTGTTTTGGCTCCTTCGACTATTGAAACTAGTTGTTTTACCTTTTTTTATTGTTTTCAAGGTGGTTTAGTCTCACATGATGCCACGCCAGACGTATTACGTGGCGTCACATTAGCCACAACGAGGTAAATTGGACAAAGTTGATACATGAAAGGGGTTAAATAAACTTTTTTCAAAAAAAAATGTTAAACAAAAATCTTTAGAAAGCTACCCATTTTTTTTAAAAAATATGGAATTTAATATCCCAAAATCTCGTTTAATCTTAGAAAATCAATAGGAAATTTACTTTAGCTTATAAAAATGAAAACAGTTCTAGATATTCAAAAATCATGCTCTATATTAATGTGCATAGATTGGAATCATTTGGAATTTGTAGGTCTCAGTTTATGTTTATTTACTAATGTATCTCTCATTATCTATTACAATTAATATAGCAGTGACAACACAAGTAATATCATTTAACCACCTCATAGTTGAGTTCCAGGTGAGCAGATTCAACCATAGGGAACAAAACCAGGTAGTAATATTTTGCTAACTTATTTCCACATCATTCTCATGTGTCAATTTTGTGCTAATAATTTAAATGTCACCAAACAAAACTCATATGCAAGTTTCCAAACAAGAAGCAAAAGAACGTCATGTGCGATAAAACGATGTCTCTTTGTGATGTGGTGGTATTTATCTAGTGGGACCTATATACGAGTCGACACCAATCAAGTAGCTAGATTGTTCAGTTACTGCCCAAACCAGAAAGGTTGCAGCCATATCCGTCTTACGGGCCAAGTTGTCGCCTGTGTTCATACTAAATAGGATCAAGTATAAGGTGGTTGCGTGGCACTTATATTATTATCGAAGTCACTTTAAATTGTATGTAGTATGAATCGTTGATATAGACTAACTATAACAGATAGCAAGAGTATATAAGATCATATAAGATCAAAATAATTGTAATCTATGCACTATAATATAAAGCATGATTTTAGAGAAAATATAAAACTGGTTTCATATTTTTTCCCTAGGATAAAATAAGGTTTCTATGAATTTTTAAGATTAAACCAGATTTTAGAATATTAAATTCTACTTAATCCATTCTAAATTATAAAACGTTTTGTAGCTTTTATTATGTATATAGATATATACTATGTCTAGATACATGCTAAAAGCAATATATCTAAAAAACAAAACGTTTGGAACGAAGGGAGCATATTTTTTATAAAAAAAATATTGAGTAGTCTTCTTTTTTGGAAGATTTATTTCTGAACTTTTTTTAAGCCTATTTGACTCCTAACTCCAATTTACCTCACTTATGGCTGATGTGGCACCTAACGTGACGCCATGTTAGACTAAACTACCTTAAAAACCATCCAAGGAGAAAAAAAACAGTCAACCCTTAAAAAACATCCAAGGAGAGGAAAAAACAGCTTCAATGGTTGAAACTTGGAAGAGCCAAAACATCTGGTTTTTAAATTTTTAGAGGCAAATTGCACAACGATAGTTGAGAGGTCAGGACTTTTTCTAAAAACCAAAGCAAATGCACAACAATAGTTGTGAGGTCAGGAGGACTTTTTCGAAAAAAAGAAAAGCAAAACAGTGAAGCTGGTCCGACCTTCTGGGCTTCTTTGCCACTGCTGAAAAGAAAAACAGTCAAAAGACACTCAGTTAAGTCAGTAGTCAGCTCTAGGCTTCTTGGACACTGCTTAGAAAAAGCAAAACAGTGTAAACGCTCCTTAATAAACACAGCACCTCAGCTTCTCTTTGCCGAGGCATGTAGGCCAACCATAAATGCATCCGCTACAATTAACAATAACAGCTGGGAGCAGTGAGTTCCATGTTCTATCAGGCTACCACTCAAGGCGAGGCAATATCATCATCATGACATCATGTCTACTCTTACAGCCACCGCTAGACAGACAGCAAATTACTAAACCATATACGCATAAATAAATGAATAGCGGTCTACAGTCTACACACCCAACCAACACCATGTTAGCAAAATATACATTTCTTGTATACAGGTTTCCAAGGGTTGCTCCCGACGGACTAGTGCCCAACTACTACTGCCAAGAAGTACCACTAGTCCTTGGATTTTATCCTATCTGCACTTACACAACATGAGATCATCAACATTGGAAACATTGGGAGAGGGAAAATCCCAGTGTTTTGCTGAACGGAAGAGAAAGGAATGTCGAGCTAACTGGCTCGAGTCTAATGCTCATCTGGGGTCATTTCATCCGCTCTATCTTCATTGGCTGTTTTAGCAGACAAAAAACTTGATGCGACGACCGCATCCATGTCCGCAAGAGGAGTGAGTTCGTCCGTCGGCTCCACCAGGTTCCCGGTGCTTTCAGCGTTGTCTAGTGGAACATCCTCTTTCAAGTCACTGCAATCACATGCCACAGGCGTGCCAGCTGCTCTGCTGCTTGTTCCAGGTGCTTCATCAACAAGTGGGAGTGCAGAAAATGCCATAGCTGAGTTATCTGATCCTTGCAATCTGACTCCAGCATCAGGAGTATCCTTCACTGGTGATCCAGAAGATTCGCTAGCTTTGTCATCTACTGGAGAGGAACTGGTTATCTGACAATCAATTTCAGGAGCCTCCGACTTGGCTGCAAGAGCTTCTGAACCATCCACGGTTTGGTTTGATTCTTCAAATGTGGGAGGTAACTCATCTTGCCCCTTGCTGCCAGCTAAAGATGACAACGATGCAGAGTTCAAGTTGAGTGAAGGTGCACCATCATTCCAATCGACAGAAGGGGCGGCTGTAGCTTCAATGGACGGGGGTGCTGGGGTAGTGACGCAATCACCTTCACCAGATCCAATTTTGGAATGCTCTACATGAGAGGTAGATTGCTTAACCTTGCAGAGTTTCCTTTTCTTTGAATTAATCCTTGGATCTTTTGGGCGAGAAAGAGGGGCCCCCGAGATCAGGAATGGAGGTATTGATGGTTCTTGTTCTCCAAGAAGAAGGCACACGGACTGAGCAATTGCTGAAACAGTTGCTGGTAGCTCGCGTTCAGGTGGCTTAGATGAAGGAGCACGTACAGCCTCCTGAAGCCAATGAGGTAACTTGTTTGATTTTAAACCTCCAAGGTCACTAGTATTATCAAGCTGAGGATTTGAGTGGTCTATGTGGTTGAGATTCATCTCGATGGGTTGTTTGCCTGACCTCACACGACGCTGCATCTCATGAAGCAAGTTGATCTGTCCATCCGAAACACCAGAAAGAAGAATGTTCTCACGAGAACTTGACCTGATTTCACTGTGCGGAATGGAACTATTATTTTTTCTGTTTATGGGAAGTGAACCAATGCTATCAGTCATGAAAGGATTTGGCATAAATGGCGGGATCGGTCCAACATCAGATTTCTCCAACTTGTCAAATGCACCGCCAGGAAAATCTCCACCATAACTTGATCTATGCTTGAAATCCTGCCATGGTATCTGAGGTGTTCCACCCTCCCCTGAGTTAATGTAATTAGCAGCCTCAATGTGAGGAAATCTGGATAGGATATCACTAGAACCTAACTGAATATCAGTTAAGTGGGACTGCAATTTAGGCTGCTCCATCCTCATTTTAGAGAAATTACTTTCATTCAGCGCCCGAGACATCATTGCATCAGATATTCCAGGGAATGTTGTTGAGTTAGAACGTCTGGTGGCTGTAGACATAGGTTCATCAATAATCTTTTGCTCCTCCAAAATCCACCTCGAGGCCAGCTCTTCACTAGTTCGGTTGTTCAAAAACTTCAACTTTGGATCTCTAAGCATAGCATCCCAATTTCCCCTACCATGCCTACGAACTCCTATCCACAGAGCATCAAGTTCATCCTCTGACCAATAATCTAGTTTTGATCTCTTCTTCAAGAATTTGTTTGAGGCATACTGAGCACGCATCATTATGTTGTCCAAGACTTTCTTGTGATTATCAGGCATAGCCGAATGGATTGGTTGCCCTGGGCTAAGACCCAGCCCAGGAAGTAGACCAGACATAAATGACTTTTGCTTTGAAGTACCAGCAATATCTGCCATGAAGTTTGGTAACAATGAACTATCTGGCAACTCTGGGACAGAACTACGAATATAATCCTTATGAGCACCCAGTGACAGGTTAGGAAATATATCAGGTACAGGCTTAATATGCTTAAGTGGAGTGTTGTTTTCCAGACTGAAGTTTGGAAGACCCAGTTTCCCTTGAAATAATGCAATGGAGGGTCCAGGATTTTCAACAGGATCAACCCCCTTCCCTGATATAGGAGGAATCTGCACATAAGAACTAGTGTTATTTTTCCTGCTTGCTAATACCGCTCTTTACTTGATAAAAAAAAATATAGGTATGCTCCTCTTGAGAATGATTTATTTGCTATATTAATACTACCCATAAAATCAAATATCAACAGTTGAGCTATGTTGTAGATAATAAAGAAATAAGAAAGTGAGAAAGAAACTATCTTAAAAGTTTTGACTTGTGAAGAAACAAATGCCCCAAAGAAGAATGATAAAGCAAGAAACTGACTATATGGACAATTAATTGGACTGAGTGCCAGGCTAGGGAAGATGACGTACATGACTGAAGGGAAAATAACTTTCTGGAATCATGTTACTTATTCTTCGCAAAGCCTCCTCAGATGCACCAGGAAAGATGGCCTTATCAGAAGCTGGTTGAGCTTCGTTTCTATGCTCATTAGAATGGTCAGCAGAAGTTGACAGTTGTTTGTTGACTATGTCACCACTAGCCCTTTTATGCTCCTTTATGCTTGGTGCACAAAGTGAGTTTTTGTATGAGTTCACCTGATCAGCATTTGGAGCACAAAGGCCAAGTACAGGTAGCAGGTGATTCGGGGTCCCTGGATTGAAGTCATCGGTGGCCTTTGAGTGGTGCTTGGATGAAGCAGCATTACCAGGAATTCTGGCATAAATATCTTGTGGGATTTCAGAATATCTCTTTGATTTCTTGGAGGATTGCCTCATTTCCCCAGTTGATTCACTAAAACGTTTCGCACCAGATAGCTGTGAAGAATTTGGGTCTTCTACGATTATCAAGGGATTTTCAGCAGGTTCATTAGCAATAGAGTCGTATGGAGTCATGAACTCTTCAAGATTATCATCAGCATAGTTCTTGATAATGTGCCGCTGGGCAATCCTTTCCTTTTGTCTAGCTCGAAGCTTTCCACTGCCAAAAGCAGAGGCAGATCAGTGCCTGAAGATGAAAATTGCACCTTTGCAAACTAGCATATGGGCTGAAATCCCACACATGACATCCCACACATGACAGAAAGCGAGGGAGAGAGTGGAGAAGAGGGCATACTATTTTTCCTTCAAGGCCAGTCCAGCTGCTGTATATTCTCTCTTTGGTTCATCTCCCTCATCAGAGTCCTACATAGCAATATCAGCAAATCGTTGCAAGATATCATAAACAGTTGGAAGATAAGCATGAAAGGACAACAGCAAATGAAAAGTAACAATTCATACAAATACCTTTGGAATGTGCAATATTACACAGGACAAATTAATTTACACGCGCTATTCATGTGAGCTATGGAGGAAAACATGAGCCTTCGATTAACCCTATCCTTCTATACTCTAAAATTGACTCCTAAAGCATGTCAATCCTAGTATCCCAGTCCAAGGCATACACACAGAAACCAAATTTGGTGACATGTTTGAGCAACTTAAAAGACAAATGCCTTGATCACAACTTCAGTGCAAGTTGATGTACCCACAATGCAATTCGCTGAATAAATAATGAGTGCTCACATATTCCATTCAATGTACACATATTAGTCACAGCAATCATTATGAAGAACAAAGATTATATGGAGCCCAGGGGGGATGGTGTCCTCTTTTAACTAAAATATCATGTTGCAATGTTTCAAAAAAATCATCACAAAAATCATGTATGCAGTACACCAAGTTAAGTTTAACCGTAACAATTCAGTTTGAAATTCATCTTGATGTTTCAAAAAAGACAAGTGTCACTGTCAGCACATGATGAACAGTGCTAGTTGGCTGCTTGTCTATTTCGTGTCTTAACATCTAGGATGAATTTCAAACTGAATTGTTGCAACTATACTTAACTTTATGTGTACTACATACATAATTTTTTTCATTGCAACATGATATTAACGAGGGTGCCAACCACCCCTGGGCACCAAATATTCCTCTTATTATGAAGAGAGCCTCAAAATTGTACGACAACACATACTTTTCAGGAAAGTTCACATGGCCTAAAATGAATTTACAATGCAAACAGAGGGATGCCGCCATATTTGAGTGTGTGTTGCTATTAGAGGGTATTCTTGTCACATCTAAGCCCAAGACAATATGGTTTCCTTGGTACATTTATCCGGTCAAAAGTGCCATATATTTGAAAACAGAGGGGGTGTTTAGTATCTAGGACAAATCTATGTGATTTTTATCAGTCTTCATGTAGTTAGTAGCAAAGCAGTAAATGAATCATCAATGCTTCCTGATGGAAAGTGAGAAATGTGCACAAAAAGGTCAGTCCAACAGGAAAATACCTCACTTAAAGCTTCATTAGGAAGTGTTGCAAATGTCTCTCTGTAAGAAACAGCCTTCCTTAAACGTTTGCCTCGGCCAAGAGATGCCTCCTCCTCTATTTGATATTGTTCCCATCTGAATAACATTATTATTATTATAGTATTCCTCAAGGTCCAAAGAGAGAGAGCACACTAAGCAAGCCGATCAATTATTGCTACAGAGGAACCCAGGCCTTTCCTTGTCTCACCAAACTATGTTTAAGTAGGTAGATAGATTTTCACTGTGAACAATTTCCTAGGTCCAAAGAGATTTTGATGCCCATGCGGGCACCAAATTCTGTCTATTCAGCACAACTGCATCCACCATACAAATCAAGAATGGTGCATGCACGTACATTTTTCCCCTAAATAAGAAATAACACAAACTTTGCCTCCATTATGATGGCATAAAGCATAGAAGTTGATAAACCACACTTTTGAATTATTTATTATACATGCTAGTGTTGGAGTATTCATTCTTCCATTTGTTTTGCTTTCTGACAAGTATACTTTTTAAAAATGAGGGAAGATTTTTAGGTGCATCATTAGGACCATCACATCACATCGCACCTACTTAACTCTCACAGTGATGGGATTGTTAGACAAAAAGATACAAGTTTATATGGTATTGCAGAGTACCTGACACGTAAAAGTTTATCCCATTCATTTTCTTCTGAACGATTAGTAGCACCTTGCTTTGATTCAGATGCTTGCTCGCTCCCATCATTATCAATAGGTGCAATATCCTCATTGGTACCAGGGTCGTCATTCAGCTCATCATTCCAATCTATTGACTGTTTAACAGAGGGAAGTAAAGAGAAAAGTGTTAGAAAGATCTCCAATGAGAAACTATGAATAGCACAAAGTAAACCTAGACCTCACCCTGCTAGTGGGTTGGAATCCAAACTGTATCCACCCTAATTCAAGTCTACTGAGCATGCGTAATTAACCCACCCCAATCCACGTCTTCTACTGGAAGGAACTAAACCACACCGAACTGTTCCCTTCTTCCAAGCCAAAGTAATCCAACCTACCATCCTAATCGTGGATTGAACCCGTTTTTGTAACCCACGAGAAGGCTGCACCGGGTGATGGCGGAGCAGCTGAAGGTGGCGAAGAACTCGTCGGAGGCAATGGAGGCGGAGGTCGGAGCACCGAAACGACCCGTTGCTGCCGCCGGACATCAGCAGGGCACAGGTCAGGTGGCCGCGGACCCACTGCCCGAACTCGTCTCGCCATCCGGACTCCTCGTCAAATGTGTCATCCTCGGCGACGTCCGGGCCCTCCATGAGCGGCGCCAGGACCTTGCCGGCAGGCCGCAGGCTGCCGGAGTGCGCGACAAGTGGGTCCGGCGCGTACTGGGCGAGCTCCTCTAGCGGCGCTACCGCCACCGCCACCGCCGCCGTCTTGCGGCTGCGCGGAAGCAGCAGCGACTTCCCCGTCATCGAAGCACTGGAGCGCGACATGGAGGAGTAGCTAGGAGTCTTCAGCAGCCCCGCCGATGTCGAGCACGCCCACTCCGCCGCCAGACGGCTGCGAGGAGCAGTGGCAGCAGAGTGACAACAGCCGGACCATGTAGGCAGTCGGCGAGGGCGTCAGCTTGTGGCAGCGGCGGACGGCGTGGTTGGGTACGCTTCGACAAACATAGGCTACTGGATTGTGATTAGCAACCATTGAGGAAAAACCAAACGAAACCATGGGCTAAAGCTGTTTCAATCCAAACAAATTGAAGCCCACACCAATCTAATCGAAACGATTTCTAGCTTAAGATCGATCCAGCCTAAACCATTTGACGGCTCAACCCAAATAAAACCAACCAAAGGCAATCCAAAGCAAAACCCACACTGTTAAACCCATTTCTACCCAAACTGTTAGCAGGGTGACCTAGACCACCATATACAAAAAAAAAGGGATAACAGAGGTGGTCCACTAGAAGTCAGGTGGTACGTAGGTCCAGGCAGGTGCCTGGGCAGGATAAATCTGCTACGTGGGATACATAGTCAGCACGTAGGGGCCCACAAAATGTAAATATGTAGTCCTACCTTTCCAAATTGTAAGTCATTTTGGCTTTTCTAGATACATAGTTTTTTACTATGTATCTAGACATAGCATATATCTAGGTGCATAGCAAAAACTATGTACCCACCAAAACAACTTACTCAGGATGTACTAATTCATGTGATAAAAAATTTACATAATTTAACAACAATTTTGACACATGGATAAGAACAAAATCCTGATATCATAATTTACCAAAAGAAATTCATTTAGGAATAAGCAATTAGGCATACAGATCAAATTACCTTCACAGTACCAAGCATATCGTTGTCCAAGTCCCCATCAGTGCCTTCGGCCACTGATGATGGAAGGTTTGATCTATCAAGCAGCTTCAAAATAGCATTTTCATCCCAAACAAGTTTGGTAGAACCACCAATACATTTGTCCTCGTAAACATCACCTAGGCCACCGGTTTTTCTTCTATGCTTAAATTCAACATCAGCAACAGGACCTGAGGCTTCATTGCTATCTTTATCATCCACACTGTCACTATTCCCAAAGAGTTCCTCTGTACCCCAGCGAATGATATCTTCCACTTCTTTCTGTGATTCTGATTTGTTAACGAAAAGTTGATCAAGCATCAATTTCTTCTTGGCTAATTGCAAGATACGCTCTTCAACACTAGCACGTACTACAAGCCTGTAAACCAGAAGTCTGTTCGATTGCCCAATTCTGTGTGCCCTGTTCATCGCCTGTATATCAGCATGTGGGTTGAAATCTGAATCATATATGATAACAGTATCTGCAGTTGCCAAATTAATACCAAGTCCACATGATCGTGTAGACAGCAAGAAAACAAAACGTGTCTTATCCTGGTTAAAGCGAGCAATCGCTGCCTGGCGCTCCGCCACAGATACCGAGCCATCAACCCTTTCAAATGTTTTAGGACCGAATTCTAAAGTCAGATAATCTTCAAGGATGTCAAGAAGTTTTGTCATCTGAGAGAAAATAAGGACACGATGGCCTTCCTTGTGTAATATTTTTAGCATAGAGTGCAACAAAGCTAGCTTTGCTGAGGCCTTTATGCGCATTTCATGCAGAAACTCAGGTGAACCAGATTCAGGTTCAGTTCCAGGAATAAGATATGGATGGTTGCAGACTTTCCGAAGCTGCATTACTATGTTCAATAAAGACTGGTGAGCACCACCTTTTCCAATGTTGCGCAGTACTTGATAGTTCTTTGTAAGCATAGCACGGTAGTATTCAGCCTGGATTGATGTCAATTCAACAGGTACCATTCGCTCTGTCTTTGGGGGGATGTTTTGCATTGCATCTTTTTTCAACCTCCGAAGCATATGAGGAGCTACAAGTTTCTTCAGCTCCTCTACTTTTTCTGCTGTTGTAAGGTCATTAAACTTCTCCTCAAATGATGATAGAGAAGGAAAAGAAGTAGGTTGTAAGAAGTTCAATAAGTTATACATTTCACCAATGTTGTTCTGTAAGGGAGTTCCAGTCAACAAAACTCTGTGCTGAAATGAGAATGAATTAAGTAAACTAAAGAGTTTGCTGCTAGAATTCTTCAGGCGATGTCCCTCATCAACTATAAGAACTTCCCATGAGACAGAACGAAGATATGCAGCATCAACAAGCACCATTTCATAAGTTGTAAGCAAAACATTAAACTTGTATGATCTCTTTGTTTTGCCAATCTGGCTTGCATCGCCTGCATGCCACTCATACTGGCGAATGATAGATCTTGACCTTGCAGAACCATGATACTCTACAACATTTAAATGAGGAGCCCAAGATGCAAATTCAGCCATCCAATTGGGCATTGTAGAAAGAGGAACCAAGACAAGACATGGCAAACTAATCTTAAATTCACAACATAGGGATGATAGAAAAGCACAAGCTGACACTGTCTTTCCAAGACCCATCTCATCAGCAAGGATAACATTTTTTGACTTGTACCAGCATTTACGTAGCCAGTTCAAGGCTTCCAGTTGATGGGGGAAGAGCATGCCGCCCTGAAGTTCTTTTGGCTGATCAACTAACACATTAAGCTCCTGTCGGTTCCTTGTGTTTGCGTAATCTTTTACCACATCTTTATCCAAAGCCTGGCATTCAAAATTCTTAAACTGAGTGACCAAATGGCTATATTTCATCAGGGTAGGTTCATCTATTCTTTCCCAAGTGCATTCATCATAAGGAAGACCACACCATTTGATTAAAGCCTCTTCCACTTCATCTACAGAAGCACGAAGAGCAATAACTCGTTGTGGCTGGCACCACTGTTCCTTACAAATGTTTATCAAACTCGTTCCATACTTCGCTTTATAGTTTTCTAGTTTCCTTTTTGCTAATATTTTTACCTCGGATTCAGCAACCCAGCTGTTGTGGATATTTGATTTTCCAACCCATTTGATCAAGAACTCATATCCAGCATCATTGTAGTCCTTATCTGTTATTTCTTCTATTTTCCCATGCTGCTTACTTTCATCTGTATGCTCTTGATTGGTCCTGACCATTGTATCAACTCTAGTGACTGCAAGTTTGGTATCATCCTTTTCTGCTGGCAAATCAGATGTCGTCTCATCTTCACCATTTTTGGAAGGTGAAACATTCTCTCTGTCTACTTGATCATCTTTCATTATACTGGTCTCATCACCAGAAGATAATATCTTCTTGGCTTGTTCTTGCTTAATTGACTCCTTTTCATGGGATTTACTGTTACTATCCTTTTGCATTTCCAAACTGTGACCACTATGATTGGCTGATTCATCAGCACATATATCCTCCACTGTACCATTTCTAGAAGACTCCATATCATCCACCTTGAGATTAGCTGATTCAAACTGCTCCAATGAAGCATGGGATGTTGGGTTGATTTGGCTCGCCTGAAGCCGACAGCCTAGGATGCGATCGACCTAAGCAAAGGCCACCATATAATGATAATCAGATGAGAATAACACAAATGATAGGACAAAACAGAGGAAAGACTAAGAATGCTTGTATGAAGATGCAATGTAGTGTAAATTGCAACCAACCTGCTGATCCTCCTGGGGAGTCATTATTTTGTCTCCTACCACACCCTGCTCCTACAACATGTTTGCAGGTACATGAGCATCACTCTGGCATATTTGTGTTGCAAAATTACTGTGCTAGGACATTGGATAAGAGAGCAGCATCTGATCGGGTATTCATCTTGCCACAAAACTTGAACGAATTGCAATTGTTAACAGATGAGCACAGCACACAATAGCTAAACAGGGGTGGGCAGATGCCTAATGATGCTATCACACTAGCAGCCAGAGACAAGAATAAAAGAAGTGTGGCACTAAAATATATGATGACAGAAGGTTAAAAAGGAAAGAAAAAAAGTGATCAAGCCAGCTTTATGTTCAGGATGCACAAAAGGTGATCAGAAAGCATTATATGGTATGTTTACCCAAAAAAAGAAAGAGAGAATTGTTAGTTCATGATCAAAATTTGAATCTGCGTCCTAAGTTATTCAGTTTATTTATAAAGAGTTATAACTGACCGAAAAAGAGAGGCTTAAGTAATCATAACAGGGAAGTTGTCAATTTCAATGAAAAGGAAAAATAACATGCAGAAAACGAAAAATATAGCACTACAGTAAATGGAGGCAGATCAATGTGAACAACTGATACTAATCTGTCCATCAAATCACCAGTAAGAAACAAAATCAATCTGGACCATTTCTGAGCAAATAACATGGAAATTTTATTTAGTTGATGAATATTTGAACATAGCAAAATGTTAGCTTCAATACCTCAGAGTGCTTTTTCTCAACAAATTTCACTTTCTTTTTCCCTTTCTTGGTACTGGATGAAGGTGCTTTGCCATCGGAGGGTTTTCTTTTTGGTGGTGACTCACTGTTATCAAGAGATGTCGACGGTTCTGATGAGGAAACACTGTCATCATCGGACATTGAATATTTAAGCTTTTTTGTGCCAGATCTTTTACTCTCTCTCTGACTATTTTGCCTCTTGCTTTTAGATTTATTTGGAGACAAGTCCATACGGTGTGGATGTTTCTTCAGTTTCAAAACACTTCCATGTTTAACAATTTTCTCTTCTGAAGGTTCTCCAACAAATTCTGTCTTCGAGGGTTTAGCATTCTCTGATAATTCCTTGGTTGAGCGCTTCTTCAAATGTAATATCAGTGTCTTCTTTTTCTCAACTTCATTATTCTTCATACCGGGGTCACCCCCCTTCATTGTGCCACCAGATGAGTGTTTTCCTTGTTCGTTTGATCCAGTTTTGTCTTGTGTGGAGTTCCTGGTCTTAAAGGAGACTTTTGTATGAGAAGGTGGATTATCTGATGCCGTACCTGCATACATCCTTGTACTTCTTTCATGTTTCGAAGTGTCAGCCTCTACATTACCTAACAGCTTCAAGCTACCTTTTTTTGGACGGCATCTTGGACATTGCCACTTCCCAGGAGGTGCACGCTAGATTGGGGACGGAAGAAGGCAAGGATTAAAAATCCGGTCATTATAATGAAAATTTGTAACTTTTGAATCAAATGAGAGGGCACAATACAAACCTTAAGAGGAGGATTGAGGCATTCCAAGTGGTAAACACGAGGACAGCTATCACAACATAGCAAATTGCCACCAAGATCACATTCTACACACTCGAAAAAATACTGAAAGAACAAAAGTAAAGTGATAAGCAAATTTTAAAACCACAACAGAGGTCACATACAAAGCAATTCATAAATAGCTAGTGTATATAAACCTCAAATTTTTTTAAAAAATACTCCCTCTATTCCAAATTATAAGACGTTTTGGCTTTTCTAGATACATAGTTTTTGCTATACACTTAGATATACACTATGTCTAGATGCATAGTAAAAACAATGTATCTAGAAAAGTCAAAATGCCTTATAATTTGAGATAGAGTGAGTACTATACTTCTTGTTGATAATTGGAGAAAGATGTAATTGCTCAGCCCTCAGCTGTTATTCATAAACATTTTTTCAATTGCAGTTGCTAAGAAGTTCCCCACAACCATTTATCATAATTTACTTAGATGTGTCAGTTCAGTATAGTTGCAGGCATGTATATTGAAACAAAAAGCACCATAGGAGCTCCGGGCTCAGTCATATCTCACGTACATGGGAGTCTGACAGACTATTTCATCTGTGAGAGAGTTCATATGTTAATAAAACATGTAAGTTCCCTTTCGCTTGGCTTGCTTATACTTGTATTTGTATAAAACTACTCCTGAAGACCTAACTGAAGCCCAGGTGTTTAACGAAAATAATACTATGTGTGCTCCATGAAAAAACGTCTTTTCTATAATGGCTACATTATTAAATCATTGTTTAAGCCTTCTGCTCGTATTATAGAAGTGCGGCACATAAAGGTAGAACAGTATATTAATCAGACACAACATCGTATCTAAAAGATCACAAATAATATCTGCACAAAAGACTAAGGTCAACTATGAAAAGTTATTAGGTAAGAAATTTACCCCATCGTGCCCTCTTATCTTCTTGGCTGATGGAGAAATGTTAGTGTCATGCTTCAACTTCTTTTTGGTGGAAACATGGCTCATTGGAGAGTCTGATGTTGGTGAGGTAGCCTCATTCTCGTTAGATGGGCTAACTGTTAACTTGCTTCTTTTCCTTTTATATTTCAAACCCCAGTTTCCATCGGCTGCGCTTTCACATAAAGAGCTCCTCTCCTTCATCTTCAGCACACTTGCACCAGAGACATACTGCAATCAAAATGAGGAGAAACATGTTTTTTTTGTCATTAGTAACCCAGCTCAAAAGAGACAACGCGTAATAGATAAACTAACCGTTGTGTATTGTAATCTAATATACATATGCAATCACAGGACAAGAGTCAGATCTCAAGTGAGATCAAGCGTGGTTTAATCAAGTATAAGAACACTACGGAGTGCTGATGCATTAGAGCAGTAGGCATTTTGAGGCATAACATTGTACTAAAATGTCAATGAACAGTTCTACTCAAGGTCCCAGACAAAGAAAGGCGGATATTACTCATCTTACTTTGGCTATTGGTTCAATTGAAAGTTCCCTTTTCTCTTTAGAGAATATTGTTGTGGAAGAGCAAATGCAAACATAACAAACTACCTTTTACATAGTAGCTTTAAATGAACAAATTTTCTTAGCATAGGAGATAAACTGAGTGTGAAAGCTAAACGACAGAAACAAGATCAGCCAGAACTTAACTTGTGGGTTGTTGCTTATAAATTTGTTAATATAATCACATCAATTAATACCCAAATTGGAACTTAACATACAAAGGCAGAACACAATAAATAGCCAAAAGCAATTAAGGAAGATCACTATAAACCCTTACTAATCAGTTTTGCACAGCTAGCTAGCATTACTCATCTAGCGAAACAATACTTAAGTAGTTACCACAAACATTCCATGTCTTGCTGTTCTCTAAAAGTCCATATGATAAACTGTCATAACCATAACTGGAACCAGATCACGACAAACAGAGGCATCAAAAGTCCACTGCTACTTCAGACCGGACACCCACCCTAATCTTCACCTCTGAACTGTATCCCCCCAATTTTGGAGTTCACAGCATTTTTACCCAAGTGCAGAAGAAACAGAGCTGCAAAGACAGACAGATAGACTAACTCCCAACCGGACCCTGTCAGCAGAAGCACAAGCATCAGAGCTTGCAAAAGCAGCTCGCCGCCTGTAACTAGCTTCACATGCTCATACGGCACAGCCACCACCCACGCCCAAATCCGAATCTCCGTCTCCGTCCTAACCCCAAACTCCATCCGCCGGCAAACCATTTCACAACAAAATTCCACACAAAACCCCCATTAACCCGAACAAAAGTGGCGCCAAATCGCGCGGTAGAGATCCATCTCACCTCCCCGCGCCGCCGAATCGATGCCGGAGCAACGGACGCAGCGGCGGATCGCGCGCGAGTAGCACCTCCGGAGGCCGCGGGGCGGAGCAGAGGCGGCGGCGGCGTTGGGTCGAATAGGGGAGGGCGGCGCTAGGGCAGGGAGG

>BAC-73

ATGGGTTTAAGGGAAAGCCTATGATTCCCGAACATACGAAGGGCCCAGAAGAAACTTCATTCCAAACAGAGAAATGTGTTGTAGCTGTTAGTCTAACGGCACTAATTGCTGTGACGATGAAACAGTTCAATGCACGAATCTGCAATGGAATAGGATAGAAGCGAGAAAAATATGAATCGAAAGATTGAGTATGAAATTAAAGAACAAAGAAAGTGATGAGAGATCAAGGGGGGGAATGTTAGGAATGATCTCCCTCCATTAGACCTAACGGCCTTTGGGCCTTGTTCTCGCGCCCTGATCGGAGGCGCCCAACCCGATATGGTTGGTGGGCCCCCGTCGCACAGCGCTATAAATAGGAGGTGAGGACCGGGGCTCGAGGTACGAGACCTACCACGCAGCCAGTTCTCCACCGACATCCTAACCCTAACCCGATCAAGAAAGGGGCGCTGCCAGCGACGGGAAGCTCCACCGACGCCGGCAACGCCACCCCGACCACCGCTACGCCACCAAGGACGTCAACTGCTTCATCACCGATCCAATGGCGCCGGCGAACGACTCCAACACCGGCGCCTCCTCCGGATCTGCTACACCTGCGCCATCGGATGGTTTGCGATTTCTACTGTTCCCTTTTTCCTCTCTCGTACTCGCGGTAGTAGTTGTATTAGGTCTTATGCATATACTGTGAATCACTTTTGACTATTTCCTATGACGAATTTGTACCCGATCAAATGAACCTGTGAACTATGAAATCTAACACAGAGCGCTTCTTAAATGCAAGAATATGCATCTAGATGACTCAAACTGTGTTCTGTGCTCCCTGTCTGTTGAAGTGGATCTCTTTCACTGGATGCTGGATTGCCTTTTGCTTCGGCTTGCTGGTTTTCTCCGAACCTGTTTATACGCCCTAACAGTGGCCCTTTTGAAATCTTGGAAACTTTCAGAGATCAACTTCAACTTCCTTTCTTCATGGAGATTATAATCGCAATGTGCTGGGATTTGAACGATGCGTAATGACAACAATTTTAGCAATGTTCCACACTAAGTGCAAAGATGCAAGCTGGTCTTCAAGAAATAATTTTCTCTTGTTATTCTAAGAGCAAAAGCTCGTTTTCATCCAAGTATAAATTTATGGCCAGAAGCCTTCATGCAATCCTCTTTCGGGTCTATTTGGAACGGAGAAATGTAAAACAGAGAAAAAAAAAAGAAAATACAAGAATAGTAAATGAGTGTAGGTAGAAAAAAGAGGAATAAAAACATAGAAATTTTATAGGAACGAGTGTTTGGAACACGGGAATAAGAACATGTATATTCATCCATTCTTGTGAATAAGGAGTTGCTCGTACCTTCATTCACACGTAGGAAAATTTTCCAAGAGCTTGAAGTGGATGTTTTATTTCCTCCATTCCAAACACCATGTGATGTAAGCAAAAATATAGGAATTAGATTCCTTTGAAATTCTTATGAAAATCCTCAAATCCAAACAAGCCCTTATTGTCTTTTTTATCTTTTTTTTTGTTGAACTCTGCACTTTGTTTGCTCTTTGCTCTTTCCTTTTTTTAATGGGATTGCTAGCGCCCAGACGTATGGACGGATAGAGCGTCCGGACGCTCGTGCCTGTAGCTATTTTCCACATCCACATGGGGCCCACGCCGCCCCAAACGTCACCAGCATCGAGAAAGAGGGAGAGGGGGCGGCGGATGCCCAGCCAGCGCCGGGAGGAGGAGGAAACACCGAGGCGAGGCAGAAGAAAGCGAGGAGGGAGATGCAACATTTGATCTACTTTTGAAATATTGAGATGCAACAATTGTAACATACGTCTGAAGGCAGATGAAACACTTGTGGAAAACGCCTGGAAAACATTTGAAAACCATTGCAAACATATGCAACATCAAGATGAAACACTTGTAAACATACGTATGAAAACACCTGGAACACTTAAAACATATGCTTGCAACATGGATGTACATGCAACATCCAAATCTATTTTACAACATACGTCTGGAACAAATGAAACATTTGGAACATACAATTAAAACATACGTGTATAGCCATTACAACATGTGCAACATCCCGATCTACTTTTGTAACATCAATATACAATATTTGCAACATATCTCTTAAATACTTGAAACATACTCTTGCAACATGCGCTTTCAGCGTAGCATCTACTTGCTGCTCGGATAAATGGAGGCTCGTCGATCTGGAGCTCGATGCCACGGAGTGGGAGGGGAGATACCAGCGGGCGGGTGATTGCGTGGCCCTAGCGGGCCATTCTCACGCTACTTCCCGTGATCATCCGCGCAGTGCAACCGGTGGGTGCGACTAGGCCGCTTGAGCCGCCGATGGGCCCCGCTACCGTGAACCTCATCCGACTCGTCGAGAAGCTCCGGGAGTAGGAGTCCTGCCACCGAGCTGCTGGAGCACAAGATCCTCAAGGAGACCGTCGCCATCGTGCTGTTCCTTGAGACCGACCTCGCGGCTCCAGGCCGAGAACGCGTGCCTCCGAGCCGAGCTCGACGCTGCCAGGACTAGCCAGCAGCGGGTGGTTGAGTTGGAGAAGGAGGTGGCGGAATCATTTTTTTAGAAGCAGGAAACATGCTCCGTATTAGGCTCTGCAGGCCCACAAAGAGTGCATCCAGAGCATCGGACGTCCTAAGCAGTTTTTTAAAATAAATTTCTAGTAGGGGCTTTCACCCTCCTGTTTCGTAAAAAAAAATGGGCCAGGATTTGATGTACACGATTGCGACAGATAAAAAGACAACCCTGTTGCTGTAGCCTGTAGGGTCCGTTGTTGTCAACAGTAGGAAAATTTGCTCAGCCAGTAAACGCCGCTGCTTGGATTTCTTCTCCCGGTAAACGACGGTGACATTTTTCCAGGGGACCGTAACCAAGTTTGAAATTATGAACTTTGAAATATCAACTCTGTTCCACCACTATCTCAGTAACAAGATCTTCCATTATCAAGTTTACCTATTTCAAAAAAAGTGAAATGGAGATAGGATAAATTTGAATCTGAAAAAAAAAGGTCCCGGGTTACTGAAAACCTGGCCTGTTTGGAAAGGGACTTCTGTGTTCTGTGAAGCAAATTCTTGTGATCTAGTAGACCCTAAAAAATTCCTAGTGCATTCCCGGTCCTCGCGTAGGCTAATTTGCCTTGAGATTGAACCCTGGTGTACAGCCATTGCAGCTAAGTGCCTGCTAAAACTGGCAGATGGCACAGGTTTTTAGCAACAGGACGAGTTATGATCAAATATTCGTGGAAAAAGGATGGAATTGTACTTTCTACAGACAAGTTAAGATTAGGTGATCGACCTACACGAAGTTGATGACAAACTTGAGTTTCGCAAATCCAAAAGGTACATCAAGATTCAAGAGTGTGGTACTCTACTCTGGGATAAGTGCCATTCTGCAGTCAGCACCCAGCAGTCATCACCAAGAAGAGCAACTGCAGACTGAACATTGAAATGTGCAACGATTTGCTTTGCCATAAGGCAAAATTTGAGGCATTTTGGCACAAAAATGCATCGACTGACTACACTAGAACGAGCAGGATCCTCTAACGTTCTTAGGTTCCTCTTTACACCGCAGACGCACATACACCTGACACTTGTTACTTTCCCTGACAATGGCCAACACCCAGGAGGGAGAGAGGAGGAAGAGCAAGAAACGACGACGCACGCGGTTGAGGTGAAGCTAGCTAATGATGGCAGAGCGGCGATAGATCCCAGAGCGAGTCGCAGGCGGAGGCGTCGAGCACCCGGTCGAAGTACTCCTGCAGGCCGTTCATGGCGTACAGCGACGCCGAGATGCGCCGCTCCACCTTGATCCGCTCGAACTCGGCCATCTCGGCGCCCGGCGTCGCGGCCGCGACCGGTTCCTCCGTGGTGCCAGCGGCTGGGACAGCGCCGTCGACGGCCGCGAAGCTGTCCAGCGCCGGGAACTGGCCGAACGCCGAGTCGAACATCCACGAATCGAACTCCTCGGCTCCCGCGGCGGCGAACTCCGGCCGGTACACCTCGCCGGCGTCCAGTCCGCCGATGCCGCCGTGCTGCATGGCCATGGCGTCGTCGCAGCTGACCGCGCTGCTGGAGCTGGAGCTGCTGCTGCTGCTGCTGCTGGAGCCACCACCACAAGCACTATTCGTGCTATGACTATTGCTTGTGGTGGGAGCAGGGGAGCTCGACGCCTGCTGCTTGCCGCCGGCGGCGGCGGCGGCCGCGGAGGAGCTTGCGGCGGCGGCCTTGGCCTTCTTGATCTTCTTGTGGTGGAGGAGCCCGCGGATGCGGACGGCGAGCGGGCAGTCAGGAGAGATGCGCGGCGCGAAGTTGGTGCGGGCGTCGCTGCCGCGGAGGAGGCGGGCGGCCTCATCGTAGGCCTTGGCCGCGGCCTCGGCGGTCTCGAAGGTGCCGAGCCACATGCGGATCTTCTGCGTGGTGTCCTTGATCTCCGCCACCCACCGGCCCGACGGGCGCTGCCGGACGCCAACGAACTTGCTGTGGTTGTTGCTGCCCTTCTTCCGGCCCCGTGGCTTGGACGGCTTGGCCTGCGCCGCCGCGTCCTGCTGGTAGTAGTAGTAGTAGCTGCTGTAGTCCTCGAGCTGGAACACTGGTGGCTGCGCCTGGAAGTGGAGGAGCTCCATGTGAGCTAGCTTTCTTGGCTTTCAGCTGCTGGGTGGAGTGGTTGCAGAGGAGGAGGAAGACAGGTGTCGATGCTTGGTACTTCTGCTGGTGGTGAGCAGTTGAGCACAGGGGGGTGAGGATTTATAGTGGATTTCAGGACTTGCAGATTGCAAGGCATCCATCCACTCTCTTGGCAATGGAACCTGTGGGGTTACTGTCAGAGGCTGGTCAAATGCTGGTATGGTCTTCCTATGATCATGTCACGGAAATGGAAGTGAGTGTTTGGGGGATAAGGCATCTTGTTGGGATCCTTTTGTCCTTTCAGTTTATTTTTCGGTTTTGCCCTCATGCATTGTCAGTCAGCAAACTAGCTAAGCCTGTCTTTTTTTAACTTATTAATCAATTTCAGTAGTTTTTGTTTTTGCTGAATACAATCAACAAAATTGAGGAATTTGTAGCTATAGTGCATCACCACTCACAACTATTTTGACCTTTTGCATTTCCTATCATTTGGATTCTCATTTTAATATCATTATCCGTGTTTGAATATGAGATATCACTACTTATTATGAAAGACAACCATTTTGGTTTTGAGTCAATAGAATGGAAATTCCTTTTTTCAATTTAAACTTTATAATCCATTCCTGCAGGCCGCAGGCAACAAGCACACTAGAGGTCAGCAAGTACATGCTCCCCACTACTATGCCAAATCACCACTAGGTAAAGCTTGGAAGCATGAGTCAAGGGGTCTACTTCTCCTGGTGACTTAAATTACAACGTCTACACCAACATGCATTACCTTAACTGAAAACGTTCAGGACACTGATTGGACTAGTTCTTCCCTAATTTCCGATTACCTCGCTAGTCAAGCCACTAACATGAGACCAGGTCTGATATGCTCCATAGCCCGCCATATGGCAGCATTAGAGAGAATGACGACGATGATTAGGATACAAGGTAGAAACGGCTGAACATCAACTTTTCATCAACCACGCCACTTGTGCCTTTGTTTATCTTGTTTCTTGACAAATACTGCTATCAACCAAGTCTTTCTTCGTCACTGGATGATGTGTAGTTTTGTACTTTTTTAGTTCAATTTCGGGAGCCCTTCAAATTTTCAGAGCATCTTTTGTACAGATAAAATATTCAGCCGCCCAAAATTGTTTTTCGTAGTATTAAACGCTTACAAGTAAGCTTTAGTACTTCAAACACATTTCAAAGAAAGTGTTACTCCGACATGGACATGGGTGTCGGAATCCGACTAGGATTCGGGTGTCGGTTGGATAAGAAAAACTTTATTGAAAGTGGATAGTGTTAATGTCTCTTTGGAGACATCCGATAAAGTTACCAGGTATAGTGTCAAGGCCAACTCGAATTTGATGAATTCTTGTTGACATGAGAGTTTTGTAAAAGGACAGATGTGAAAGGTCGATTCCCCGTTTTTGAATCACATCTTCGGAGACATCCGATAAAGTTACCAGGGGGTGATTGATGATCTTTTTTGAATCACATCTTACTAGCCAAGTGGTGGTATATGCTTCACTACGGATACACAAAATTCTTGTTGCAATTAGGTGATATTGAGCTGCGTTCATATTTCCATTTTTGGCAACGGGCACTTTGGAGTTCAAGGAACAACAACTAGAGTCTTAAAGCTGTGTTTTTGCAATAAGAGACAAATCTAGAGCCCGGGGAATCTTTGCTTGGCTTTGAGACTTGCAACAACTCTATAGCCGGTTAAAACTGAAGCAAGCATGACGGCAAAACAGTTCTTGATATCTCCAACCTAAAGAGACGGCACTGCTAGTTTAATGAAATAGGTCTTTGCACCCACAAAGTACTGTAAGGATAAACAAACCTACATTCCTCGGTCATCAACGGGCATTATTTTCTTCTATCACTTGAGAAAGGGTCATCGTCATGGCTGATAGCCACATCTCATTACGGACTCCTTCAACCTCAAGCTAGGGCATGCATGCCTACCTCACCTATCAAAAAGGCAAACTTTGTCAAATTGTGGCGCGTCATTCCACCACATAAATTATGTTCAGCCTGCTGATTGAGCAATAGGATAGGGCACCCACATTGGCCATAGTTTTTTATACTACGTACTAATCAAGATGACTCCAAGAGGTTATGGCTTGTAAAGCTCTTGTTTCATAGCAGCGCAGATGCCTTGTTTGTCGCTGTCAAAGTGCATTAATTTGTCGAGCTTGGTGGGCTACTTGCGCTAACACGACAAAATACCACTTTTTTGGGCAGCAGCAACGTCAAGAAGATGGCATCTTGGCATCGACTTTTGGGTTGGTTTCAGATTTGTCTTGTGGTGCTGAGCCGCCATGGAGGTGGAAAAGATGGACTTATTCCAGCCGTGTTTTGGGAGGAAAACCTGTGGTGACCAATTCTAGCTCATTGCTGATGTCAGAACTTTTTTTGCAGCGAAGAGCCGAAGATGATCTGATAGGCACCTTTCGAATTTGTAACGATTGATTCTTCTGCTATGACGAATGTACCAGCTTTGTGATGTGTATACAATTTCAAACAAGGACTACCTGGCTATTGGAAATTGGGACGGTATACAAGCTCCACATGATACGGCTCAATTGAAGATATTATGTAGCCGGGATAACAATTTCAGTTGGGAATCCCCAGCCGTCGCGAATCTTCAATTCCACCGCAGTTTTTTTATGCCACTCATATGCAGACAAGGTCAAAACAACTGATGCAACCACTGTCAGCAAACTACCAGATGGAAATTTGCAACGGTTTTATCTCTGAACATATTATCTATTTGCTGCGGGTTTGTTCACTGAACAAATTAGCAGTGAAGCAAAAGTTGACAAATGACACCAAATACGCACCATCCGGCTTAAATTCGAGTTAAAACGGCTTTTTACACAAAACTGGTGAACATTTATTTATCAATTACTCTTACATGATCCCGGGGCAGGAGCTTCTGTATCTTCAAACAGAGCACAATTTCATAAATAGCATAGAAGAGAAGCAGAAAGTAATCACTCCGCTCAAATGTATAATGATCTCATTACCAAAACTGCATATGTCCAACCCATCCAACAATAAATCCCACCATTTCCCTTGCACCTCTGTTGCAGGACATCTCAAACCATATTTCGAATACATGACAATATACACTCTATATCAAAACCATGTGTGGTGGCAGCTTCCGGTTCATTGAAGCAAGCAATGCATTCACAAATTACCCCACCCAGAAAAAAGATTCCACATAAAGAATTGGCTGACAAAACATCAAGAAAAATAACAAGCCCAGACCTTGCAGCGACAGAAAAAAAAACAACTATGTCGACAAGATGGACTGTAGCAGTAGCTGTTGCTGCGGTTGCAAGTTGGAAAACGTTGATGCAAGAAAATCAGGAGGTAATGTCTGCTGGAAACGCCTCAAGACATTCACCATCCTGTTTCTAGTTTCATCAGTGGCGAGTTCTGTTCCATTGCACAAAACCTACAGAATATACAGATTGGAAAATAAACCATTCAGGAATTCATTCATGACAGTTGAACCAAAATTACATGGATATGTAGGAGAACAAAATAAAATAATGATCAGCAGAATCACAGATTTCAAAATCAACTGCTCATGCATTACCTCTGCAAAAATTGAAATAATTTTAGGAAGATACTGACTGTGAGGACCGAGAATTTCAGTATCCGACCTGCAAGCAGAAATGGTTATGATAAACCGCAAAAGTATTATCCTTGTCGGCAGATTTCCAGTCATGAGCCTACCTCTCCACCATTGAGCAAAGTTGTTCATGTACAACTTTAGCTTCAATCTTGTCATCTTTTATAGGCAAGCAGCTAAGCCAGGCTGGAACAACCTGAGAAGCATGAATTGATTCAGTTTGGAATAGTTCCATGCGCAGAGAAACTTTCTCCTGGCAAAGCTAACAGCAAAATCAACACCATGCAATACACTTAAGCTTAGGTTGTGTGATATGATGAATTTTTGTGGTCCATCATCTTCTCAATCCTCACTTTTTTTGTTTGGATGTGGCATAGAATAAGTTGGTTCTCCACCACTTCATACCTCGCAAGCAACAATTAGCACAAGCATCGTGAAAGGGTAATTCCACCAAAATTCATGGGACGAACTCGATGCACCACCTCATCTAGGATGGGTTGCTTCCTCAAACCAAAAACCCTCCCTAAGGTCTGTCTCACTAGTCATTCCCTCCCTCCTCGAGCGTCTCTTTTGGGTTTCATCTCTAGCCTACCCCAACTTGCTTGGGACAAAAGGCTATGTTGTTGTTGTTGTTGTTGTTGTTGTTGTATCTAATCCGCAGCAGGTCCTCCGGCCCCACCTCACCTTACTACTATCCTGATTTATGACCACCTAGGGTGCACCAAACGACTTCTGCGAAAATGTGTCACATTTGATTGACAATGGATATAGCCTACAAATTTTTATCATATCACAACTACACAATCATCTCAAGCAAGTTGTGGAAGCCACATTACTAGAAAATATTGGCCATAGCATCAGGAAAAGATGCTGAGATTAGAGCATACCTGAGCTGCATCAATACCATCGCGATGAAATTGGCATATTTTCCCAAGTGCCGAAACAGCATTATCATATGCCATGATATTGTCAGGAAGTCGTGCCTCAGGATGTCTTATAACATTGTTTAATTTCGATAGGGCCTCTGAAATCGGAAGCAACACATTGTTGGATAGGTTGGAAATGTCAGGTAAAATATAAGAAGTGTAAACACTTAAAATGTAAAGAACCTACCTCCTACAAGAGGGCGAAAAACATGACCACCAAACTCGGCACATACACCCACACCATAGACAGCAGCCTAAATGAGGAAATGATTCAGCATTATATCAGTGTTCAATTTTGACCTAGTAAAAAGGGTGAATGGATCAAGGGAACATGACAGAACAAAAAACAGACCTGCCGAACATCTGAATTTTCATCGTTTGATGCCTCCAAAAGGAAAGGAAGGTATGTGTCATAATATTTCAAAGCTGATTCACGGCATTGCTCTGCAACATCATCAAAAATACAGATAGCTATCCTTCTCTCTTCGGGTGTTTTATCCTTTCCCTGCAAAACCACAAGGTACCAAAATGAGATAGAATAAAACCCAAAAAAGATGGCACCACACAGCAAGTTAAGCATGGAAAAACACATCAGGGTAATACTGGTAAGGAAAAAACATTAGAACACAAGCATGACCAAATATCGGCATGCAATAACTGCAAAATTACTTGCAATAAAAAGAGCTCTTACAAATAACAAAGTAGGGCAGGGTTCAATTGACTTACCAACATTGGTGTAATGTACATAGAGAGCTCTTCAAAAAACGGCAAGAATGAAGCTTTGAAGGTTTTAATCAAAGTTCCTAGGCACTCGCTGACCTGGAAGTTTAAAGAACGTAAATTTAAAAAGAAAAAACAGGTTAGCCTTTTAACATAACTTGCAGAAGACAGTATCATGAAAACGACTCAGGAGAGAACAGACAAGTGATTTCTAGACAACTAAACCTGATCAAATACTTCCTCCTCCTGTTCATTCTCTTCTTTGAGTAGCTCTCCTTCATCAGCATCAAAATCTTCCGCTTTTGTTCTCTCAGAACGTTCTCTTTTTCGGGTGGCGCTGGCTATAATGACATTTTTAATCTCATCCGAGATAGCTCGTACTTGGGTTTGATCAAGCAGAAGACCGGAAAGCTGCTCAACGAGTAACATCAATGTTCAGTGACAGCCACAAAATATTTCAGAATAGAGAATATTTAAAAACTCGTGCAAGGAGAGACACAGCAAAACTACAGCACATTTTGTTCCATACTTCCATCCAAAGAGCACAGCTAAATATTTCCTCCCCACTTTTAGAAAATCAAGAGGTGATGACCACACATCCGCACAAAAACACAACCCAGTAGCATACACCCTATTACACAAATGAAACAGCTGAAAGCACAATCACGTGCATAAATCGTGAGCACTAAGATTAAATCCCTAGGTGGTGGAGTAGTAACTTCATGTTCCGCCACCACAAGGGGCGGTTGCCCTCTGTTCTATTTATAAGGTGCATCTAGGCTCAGCAAGGTCTTCAAATTCATTCTGGCAATTTATTTCTTCAATACAGACGAAGACATGTTCTGGTACAAGTACAAAGATCACTCATCTAAACTCCATATAACATCCTAAAGTTAGCTTCTTAGACCCCAATTGCCAACCTTTGGCAAGAGTACCAAGCTTTTGCCTGGCTGAGCAAGAGTACAAAAAACTGACCCCCTCAATGTAATGTTATTCAAACTGTTTTATTTATCATATTTCACTACATGAGACTACCATCAGCAATTCTGAATAAAAAATGGATGAACAATTTCAGACATACTGAACAATAGAAGATATTTTTAAACACTTTCTACAAAAGATCATTTTCAACAAATAGAGCAACATCTTTATTCCAGAAATGGTAATAACTTTCTGCTTCTAGAAGACAAGACAACAACATGTGCAGGGATAAAGACCATGGGCCCATGAAATTCTGCACATATCGTGATACAGAACTTTCAGGCTAAAAAAATACATGCCTAGCATTATTCGTAATGATTCATTCACACTTTGCACATAAAATATTGCAGATAAAAACATATACCAGTACTTTTTTGGTAACAAGGGGGTAGGCTAGAAAAAAATGAAGACCAAAACATCCAAGTGAAGCTTTAAAACCTCAATTATGGACGATATTTATAATTATCAAGCAGATGGTATGAAGTTACAAACCTGCATACACTCATTTAAAGAATCCAGCATGGATGAGCACATTTCTGTTTCTGGCTCCTGTACAAAGGATGCCAAAAAAAAAAAAGTTCAGGTGTCAGCATGGGTAAAAAAACAATAACCAAACTGCTGAGATATCTTCAAACAGACCTTATGCAGTGCTTCAACAAGTGCTGGAATTATGTAGTCAGACAACTGTTTAACATAAGACTGATCACGCCCTTGAGCCTGCCCCTTCTCAACAGCCAACTTTGCCGAGCGTAGGAGTTCGGGCATGGCTGCATTATGTCCGGAAATTGTAAAAAGAGCAAATCTATGTGCACAACTAAATCATACTGAAACTTTAAAATACCTGCAACAGCAGCTCTCCTGACTTCCTCATGGAAGTAAAACTTCAGCAGAGGGACTAACGTTGGGGCAACCTAAATGAAAAATTATAATCAATCACATGCTGGAAAGGTGCAGCCAGAACTTGAGGGACTTAGACATTAGATACGTTTATAATGTGATAATGAACCGATAATGAACCTGATCAATCCATGGGAAGAAACCCTCCTTAAGCTCATCAGCATAGCAGCACAGCATGTTGCAAGCTGTTGCTTTCTCTTCCAGCACACTAGTTCGGATTCCTATTCTTTTGTCACCAAGTGTGATTGTTTCAATGCTGTATAAAACACAGAAACTTAGGGTAAGTGTCAGAAAAAAAAATGCACATTTAAATGTATGATGCACTCCTTGACACACCAGAAAGGGAGGTGTAACAAAACCTTACCATGATATATAAATATAAATAATAAATAAATAAAAGCATGTGAGAAGTCCATAAATAACAAGGCATCTGCCGACTATAGGCATATTACATCTATACAAGTCCATGTATCGTTGAAACTGTACAAGAGCAAGAGAGGTATAGATAGTTCTATGTGGCAATAGTCTGCCATGATTCAGGACTAGGAACACAAACTCTAATCAAGTAGATAAAAACATTTCAAGAGAATGAATAACAGACCTATCATCATCTGATTCAATATCGTCATCTGATTCAGCTGAAGTAATTGTAACATCAGGCTTCAGCTGAGCAGACTGAAGCAGTGGAGGCATAACAACACTCATGTAAGGAAGGAAATCCTGCCCAAGACATTTGCATAGCCTGGCCCAAGCCTATAACATGTGGAGATAGGTGTTAGACATGTTTAAGGTGCTGTTAAGCCTAGATTCCATTGCTTTGAAGCAATACCTGCAACATGTAGCTGGTAATTGGATCATCAGTTTCCATTGGAGCTCCTTGCAAAGCCATAAGTACTTCCATAACCTAGCAGTGGTCAGATGATTAGAATAATAATAAAAATATCAAAGCTGATGAGGAAAGAACATATAGGATGTGTTTGGTTCACGGGATGAATGGGATGGAACAATTCCATCCTACTTTTGCCTATGTTTGGTTCAACAACCCATGGATGAGGGCACTCCAGAGTAAGAATATTCCTACAAGATTCAGGCTCCGCTCATTCTCGCAATGGGCAGAACGCGTTCGTTCCTTTTTCACGGCGTTTCTCACTGTTTCTCTCTCCAACCTTGCTCTCTATCTCTCTCCGATGCGGCAGCGGCGCGTGATGGGCGGTGACGGACGCGCCCACAAGGCGAGCGACAGCGGCCCGAGAAGCAGCGCTGGCGGCAGCCACGCACATGATGGGCTCGGACCGAGCAACAGTGGTGGACGGGCGCGCGAGGGCTGGTACCGAGCGAGAGCGAGAGCGGACACAAGGGATCTGACGGACGCTGGCGCGAGGAATCTGTAGCGGAGAAGAATGAAGAGAGAGGGGAGAAGGGCATATTTGTGTAACATGTTGGTCCCACAGTTAACGGGATTAACTGATCTGCTATCTGGTTCAGTCCCTTCAACCAAACACGAAAACAGAACCGCTCCATCCTTCCAACTAAACAATAAACTAGGCATGGAACCGTTCCATCCTACCTCGCTCTCCAACCAAACACACCCATAAAGAGCAAGAAGTGGAGTGGAACTGGAATCCTCAACAACAATTCTATCAACCAGAAAACCTATGCATTTCCTGAATTCTAACGCGTTAGTGGGAACAGGTTGGACAATGAAACTGACTAAACATGAACAGTGATGAAAAAAATCAATTACAAGTTATTTGCATCCAATTAGAAGTATGTTATTTGCAATCTGCAAGAAAACATGACAGTAGGAAGGAGAGGCCAACCTGCCTTGCGTCGTCTCTGAACTTGTCCTTACCCACAGCCATTCCTACCAAACTTATACACTCCATGGACTTGGCACGAAGCATCCTATTGGACTTGTCAGTTGCATGCATCAAGATAGATTTTAGGTATGGCATGACAGCATCATAGTATTTCTTGAAGTGATCCTGCAAGACAAACAAACAAAAAGTTATGTTGTAATGCCATAAACTAATTATCAGCTAGACAAACAAAAAAAAACGCTGTCATAATATTATGAAGTTGTTATTATCAGCAAGCTAAAAAGTACTGCAACCATAGGACAACCCTATGATGCATGATAACAGAGATCCATTACGCATAATAGCATAAAACTCCCATGTGAACAGTTACCTGTGATGAATCTGCTACTGACGCTAGAGCTGTCAATGCTCCCTCCTGCACCATTTGCTTGCCATTCTAGAATATGAACATGAGCAGTTAGCATGTTTACTAACCATGACAACAAATATATTTGAAGCAAGCTGAAACCTAATTCAACACACAAGTAAATGCATACCTGAAGAAGAACAAGTAATTTGTTCACGATTCCATCCAGGTAAGGCGTCAAAATTTCCGGTGTACAATTCTCACTAAAATTCAAAATCGCTGAAGCTGCATGTGCCTGTCAACAAGAATAGCAAATTAGCAATACAGTAGGTTGCCAGGTCCTAAGATGTATGACATGAATAGGTACACAGGCTGATTTAGAATGTAGGCATAAAGAATAGTATACACTTAAAACAACATTCAGTTCAGACGGCGGGCATCCATAAAGTGTTACCTTCCATTAATGCCTCACTTTTTGTGATGGTGATGAAACAATGAACTTAGTAGTTGCTTGGTAATTAAATTACTTTTTATTCAACAAACCTGTTAGGTTGAAGATCAAAAGGAATTCGTTTGACATTTAGATGTGTTAACTGCCCGCATCTATTTATAAAATGTAAAATGAACAAAAGTCATCTTGGTCCACATCAAGCTGTTTATTGATGCAACCTAGATTTTAGAACAAGTAGTGCAAGCAAAAGGATGTGAGATAAAGTTTTGCTGACATGACAACTAACCTGAACTCGTGGATTCTGGAAATCATCCATAGCATTTGCCAATGCAGGCAGCACCTGCTGGTGGTAATGAACTTGAAGATCTGGACCCAAATCCGTAGAGAGCTGACCAATGGCATTGATAGCAGCCCATCTCACACGCGGGTGGGGATGTTGAAATCCATTCAATATCATCGAAACGACTTGTTCCAAATTTTTAAGCATAACCTGCAAGGAAAAACAGCAGTCAATGAGAAGATGGTCTATTTCGGAAGATAGCATCCTGTGGCCCAATACTAGCCAACAGATCTTGCAGAATTTGCAATTTAGTATTTGTCAGCCCATTCTACAAGAGGAATCCATGGACAAAAATATTGTTCATAACATATTAACATCCCATTTCCTGATGCAACATGCTAAAAATATCAATTTTAACTATCCAAAACAGCAAAAACAAATATCCTTTTGGTTGGTCGACTGATTGCTGAATACTTGACATATACTTATAAAGTTAATCAGGCTAACCTTTGCACATCCTTCCGCAATCTGTGCAAGTGTAATGAGCGCAGCATGGTGTTTCTGCCACTCAGGAGCAGACAGGTACTGTGGTAGCAGCTCAGATGCAATTGGCACAATTGCGTTCCCACCAATGGCAATGGCAAGGCGGTCAAGGCACTCCTGTGCCACTCCATAGTTGTTCCCTTCCCCTGCGTCCTCATCTTCAGTTTCAGCAGAGTGCCAAGCAGGGTCATCCTCGACATCAAGCAGCATCTGCATCAGCACTGCAAACAGCCTACCAACAAACTGTGGGAGTCGCCGCATCATTCCTGGTGCACGCTCCCTAGCCTCGGCAAGGGTAATCACAAACTCGACCGCCAAGTGCCTGGTCCCATCTTCCAGCTGTGCAGCCTCAGCAACTTGCAACATTGCCCCCACCACATCAGCAATCTGTTTCCTCAGGAACCTTGGCTCAGCGCCAGCAAGCTCCACGAGCAGCTCCAGGGCCTCCTGCGCAGAGGCTTCCTGGCCAGAGTTTAGGCAATCAGTGAGCGCCCTCATCATTGCTGGGAGCAAATCCTGCATCTTATCCCGGTCGGAGTTCGTTGGCAGGCACTGGACGAGGTTGACTGCGGCACCCAGGGCCGCAATCCGAACATCAGGGGATGTGGGATGCGCCAGGGCTGAGGCGAGGAGGTTATGGATAGTCATCAGATGGTCGAGGAGCGATTCCGCGATGTAATCCGCCAGCCTCGCGAAGATGAGCAGCGCTGACTCCTGGAGGTTGGGCGCCTCGGGCCCCGACGCAGCGCGGAAAAGGAACGGCAGCAGCTCGGGCCAGGCGTTCTCGGGCAGCAGCGAGGCGGCGAGCTCAGAGACGGCGTCGCAGACCTTCTTGGCGATGGGCTTGGGAGGATCGGACTGGAGCGCTGAGAGGAGGTGCGCCTTGAGCGCGGCCTGTCCCTCGGGGGAGAGCTGCGGCCAGAGCGGCGCCGGGGGCGTTGCGTTGTTGGAGGATGCGTCGGAGGACGGGGTCGGGGAGAGGACCTTGCGGAGGAGGACCCCCGCCATGGCGCGCAGGTCGGCGGGGGTGCCGGGCGCCGCGAGCGAGGTGGCGAGCCGCAGCGCGAGGGGCTCCGGGTGCGAGGCCCGCAGGCGGTGGAAGGCGGCCTCGGCGGCGGAGCGGTCGGCGTTGGAGGACGACATGAGCGTCGAGAGCAGCGCGTCGAACGCCGCCGGGTCGCCCCCCAGCAGCGCGGCGGCCGCGGCCTGGTCCTCGGCGGACGCCATCGGATCGGAATCGAATCCGCGGGCGAGCGGGAGGGGGACGGCGGCGGTTAGGGTTTGAGCGTTGAACCGGTGGTGGTGG

>BAC-77

TAAATATAGATAAAAAATAAAACTAATTACACAGTTTAGACGAAATGGACGAGACGAATCTTTTAAGCCTAATTAGATCATGATTGGACACTAATTATCAAATAACAACAAAACTGGTTACATTGCCATTTTGCAAAATTTTATGGATCTAAACCGGCCCCAAGTCAATCTGCTCTTTGGTCATCGAGGACGAACAGCTTCAGAGAATACTACTACTGCTAGCATGACGATGCTACTCTAGGTTGTAGGTGAGCGCCTGCGTGCGTGAAGGGCGGAGGGCCGTGGGACGTGGGGTACAGCCGTAGTACTACATGCCTACAGGGGGTTGCTGGTGTCGGTGCCGCCGGAGTCAAACCGGAGCCATGCTTGCCAGCCCAGACCTGCTCTGAGCCTCCGCCCCGGTCGAGATCGTCAAGGGAAACGACGCGTCCGGCGGTCCGGGTCGGGTCCGTCCGGCTAGGATAGATAGATAGATACGAACACTAGCGGTGGCGGCAGCAGTCGTCGCTGTGAGCTGTCGCGGGCCACGCCGCACCCGGGCAGGTGGGGGGTGATGTGCTCCACCGCTCAGTGCCGGGTGCCACCGCCCGGTACTGACGTGTGGAGCGGTGGCACTCGCCGTAGTCCTCGACGTCTTGGGATCGGCAGATCCATGGACGGACCGAATAAAACAACAGCGCTGCGCGGTTCCTGTCAGCTCAGATCAGGAGTGGGCGGGCTACCATACCAGGTCGGGGCCGTTCTGCCCAGCCGGAACGTGCAAGGGCTCTTGAAAATCTTGCTGTCTTAAGGCCAGGCCAATTCTATCATTTTCGGCAGAGGACTACTACGGCCAATTATTTAAGCTCATTTTCCTTTTTTTTTTCTTACGATGATGAAACATAGGTCCGGTACGGTTGTATGCCTACACTTATGAGTGTTTATGATGGTCCTACACTTGTTGGTGGATCTGGCTAGAGTGTTGTTGATCAGGCCACTTTACAGGGCACTTACGTTTGGATCTTTGCGCTGAAATAGTTGGGGATTGCGCTAAGACTAATCATCGACGTAGATGGACGCTATCAATGGCCACGAATGCCGCCCTCCGCTTTACAGGATGGAAGCACTGTTTTCGCAGTACGCGAAATTAAAACCCCCCATCCACTTACTACACATCTCGAATCCATCCATTCATCGCTGGATAATGAATCCATTCTTCCATTCCGATCCATCCTAGAGGCTAGCAGCCTTACCCCACTTTCCCAAGAGGGGCTGCTGTTACACTCATGTCGCTTTAGTTTTACCACTGCCCGTCTGCGCCACACTCACCAGCCTCACCACGCGACACCCCGAATCTGCAAGCGACGGGCGTCGTCGCCTCGCATGCCTTCGCCTTTAAGCTTCCAGTGATGGGGTATGCGCAGGAAGCTTCTCCTTTGTGCTCCGTTTGTTCTTGGTGGTGGTAGTATACCAGCCGGCGCATCTTTTTTCCCCCTTTCTTTCGTCCCACATTTAATTCACGGCTGGAGTAGAGATGACATTGTCAATCAATCATCAGCGGCGCAGCAACCGGCTCGTTTTGACCCAAACCACGTGGCCAACGCCCACCGGCATGTATAGTATTACCGCTCACCTACTGCTACTGCCCCCGGCCGGCCGCAGCAGGCTATGCGACGACCCGACCGCTTGCTTTCTCCGCTTCCACATTTGCCCCATGAACTCGTCACTCACACTCACAGTTTTGTATACATGTGTAAAATTTGGCATGGCGTGACTTGGTGCCCGTTCCCGTCTCACCAAACCTCCAGATGAATGGTGTCCCTTGCCACCCGTAGGCAGGCAGTGGAGGCAGCACAATGCTGTCCAACGATTGCGGTGTTCTCTGTGGGGCAAGGGCGCAAGGCGGCAGGACGCCTGAGTTGTAAAGGGACCATGGCATGGGGACGGGGTGAGGAGAGTGGTGCCGACGGCATCTCATTCTCGTCTAGGGATCGAATCCGATTAAAGAGCCTGTCTATACTCTAGTCTTGGCGTGCTAGTGAAAGGCAAAAGAGAAACAACTCAAGACAGAAAGAAAGAAACAATAAGAAAGAATTGGGGAAAAAAAAGAGAGCATGTAGCCTTGTACTAGCTAGGCGCCACTCGCCCCTAGAAATTTGCCGGCCGTGTGTGACCACTCAGGACCACCTTTGCGTTCTAAGGGGAAATGCTGGGGGGGTCCTCTGATCTGATGCTTCTCGCAATTCGCAATTGCTTTTCCATAGGCCGCATTGGTTATATCCCAGAGGCGTTGGATTCTGTTTGTTCTGGGGTCGCAGGTGCGGTAGGTTCGGTGGACGCCGATATTGATGTCAGGCAGAATGGCAGATATATATAGTGCTCTACTGAGTCATCCGAAAAGAATGCCTTTTGGGATCTTGTTGATTAGCATTTGTTCTTTCCTAGATTTCAATTATTCTGAATTTGATAAGCATGTCTCAAGAAAGGCAGTGTTAGAGTATGGCAGGGGCTATTGGGCCTGGGCTGATGGTACATCCCATTAGTGTTAAGGTTAATTAGAGATAACGGTCGATTGCTTAGGAGTAAGTTAACCTCTCTATATAAGGAGAGGAGATATATCAATCTAATCAAGAGATTAGAAGGAAAAAACTTCTCTCTTGCCGGCCGTGGGCAAAGCCCCGCGGCCGGCGTTCCCAATAGGCCCCTGCCATACTCTAACAGTCAGGAACTGCGGTTTCTAAACTATTTATTTGATTTGCTTGGATATATGCCATTTGCAATTGCACAACCCCGAAGGTTCAGATGTTAGAAAGTACACTCCAGTGTCCAGGACAATGCCCGGTAGTTAGAGCTATCCTGAACTACAGCACTACGAGTCTGAATATTTATTGAGCTAGAAACTATGCATGGGGATAAATGATTCAAGTATTAAACACACCGTGTCGTGTGGAAGCGTGGGAATCTGACAGCTGCCAAGCTGAGTGGTTCCCCTTGCTATTTTAGTGCCTGTTGTTTCTCAACTTCTTTGGAACGATGCCATGTGGAAATTTCTCTCTCACAAGCATTCTCATGTGGAAACAGTGGTGCTGTTTGCCTGCTATGACTTTTGCAGGCCCTCTAATCATTTCAGCACCTCTTTGTTCAGGGGCTGATGTTGAGTTTAAACACGTCTTTTTTTAATATGCTACTAATACTGATCTCATGGTGGTTTCAGACATGGATGTACATTTCTGTCCCGCTGGTGCTCTACGTTGGTGAAAGGATGCTTAGAGCCTTGAGGTCAAATGCTTATACCGTAAAAATTCTCAAGGTGAGTACTACTGGTGGTAACTGCAATGGCAAGTTCATGCAGCTTGCAAGGGTCAAATCTTTTTTAATAATCTGTACCCTAGTGCTACTAGTTGGCTATCTCCACATGTTGATTACTTAATATTTCAGACTAGAACGGTTCTTTGATAACTTGCACAAATTGCACTCAAAGAAGAACAAAAGAGAACAACTTGCGCAGATTCAGTAATTAGTGATCCATTACAAGTTCTGATTCGGTGTAGTTTGATACATTATTTTAGCTTCAACGCACATTTTTTTTCCTTTTAAAAAAAGGGGATGTGTATGTGGATAAGCAATGGAGAATGTTGTGGCCCAGTCATGCTGTCACTACAACGACATCCACAAAAGAATATAGGCCCTGCTCGAACATTTTCTGTCCGTCAGAAATTGCTTGTCACTCTAATGATTTCCATGTCTCGAAGGTCCATGCTTTTGGTTGATTGTTTATTACTTTATTCTGTGTGCTCTTCTGCCAAGTCACAACGTCAAATACATACGTGTAATTACTGGTTTTAACATCTAAAGGACTTTTCTCATGCTAAGCAGTTTGATAGATGTGTTTCTGTTTTTCTTTTTTCACCACACTGTTAGATCTGAATGGAATAATCATCTAAGTATTAGGTGCTACAGTAACCAAGTCACGAAAAAAAGGTGCTGCAGTAACCATAACGAAGTGTCAAAACAAGAAAGAAGCATGTGCATACAGAAACTATACATTTAACATAATGTTTTTTTACAACTTTCTTTTTGCCAAATAAATCCATCGATCAATATTGTCTTATGTAAATTTCATTACTGCAGGTGTGTCTTCTACCTGGAAATGTATTGACCATAACAATGTCAAAGCCCTACGGATTTCGATACAGAAGTGGACAGTACATATTTCTTCAATGTCCAATGATTTCTCCATTTGAATGGTTTGTATGACTCCCGATTTGCATCTCCTCAATCTACTCTTAGTTCTCTGGTTAATTTAGTATTCACCTGCTGGGGTTTTGTGTTTGCAGGCACCCTTTCTCCATCACTTCAGCACCTGGAGATGACTACCTCAGTGTTCACATCCGAACAAATGGTGACTGGACACAAGAGCTCAAGCGCATATTTGTCGAGAACTATTTCTCACCACATCTTAATAGAAGAGCTTCATTTAGCGAGCTAGGTGCGGCAGAACCAAGAAGGTCCATCAAACTCTTTTCCCCGTGGCTGCTTCTTTACACACCTAAATAGAACTGAAACTGAACGTGTTTGTTTTCTATTCCACAGCTTGCCAAAATTACTTGTAGATGGTCCATATGGTGCCCCTGCACAGGATTTTAGAAACTACGATGTTCTACTTCTTGTTGGCCTTGGAATCGGGGCAACACCATTCATAAGCATTCTAAGGGATCTGCTTAATAACATTAAGATAGCTGACGAGTTGATGGTACTGCTTTATCTTGATTTGCTCTCATTTTAACCTTTTCCAGCAGATATGGTTATTCCCATTTATAGCTTACATAACTTTTTGCGATTATTAGGACTTGGCTATGGAGACTAGCAGGTCTGAAGACAGCGCCAACAGCTTTAGTGTCTCAACAGCGAGCAGCAACCGGAAGAGAGCATACAGAACAAGCCGTGCACATTTTTACTGGGTCACTCGAGAAGCCGGATCATTTGAATGGTTCAAAGGGGTGATGAATGAGGTTGCAGAAATGGACAAGAAGGTAGAATTTGCTTCATGCCACATTGCCATCCATCTTGATGTCTGATCGTCCCTTCAGTTTACAATATGCCCACAATATTTTCTATACTGTACTGACATTGAGCTCGTTGAAACCAGGGTGTCATAGAGCTGCACAATTACCTCACAAGTGTTTACGAGGAACGAGATGCACGGACAACTCTGCTGTCCATGGTGCAGGCTCTGAACCATGCCAAGCACGGCGTCGACATCGTTTCAGGAACCAGGGTACGCCAATTATCTGAAACTAGTAGCACAAGATAGTGTCCAAAGATCCTCTATTCTATTATTGCAAGTATAGATTGTAATTGAGCGTTTTATGTGCACTCGGCAGGTGAGGACACATTTCGCCAGACCCAACTGGAAGGAAGTCTTCACCAGGATCGCCTCCAAGCATCCGAATTCAACCGTTGGTAAACAGTCTGCTCAACCAACTCTAAAATATCTCCTCAAAATCCATAAGAATGCTTCTGATGCTATATCATGCCTGCAGGTGTGTTCTACTGCGGCGCACCGATGCTTGCCAAAGAGCTGAAGACTCTGTCGCACGAGATGAACCACAAGACGGGCACTCGCTTCCATTTCCACAAGGAGTACTTCTGAATTTGGACAGAGAGAGAATGGGCAACCGATGCTCGTTCTAATGTGATCTGATCTGATCTCGATAGCGTTATAGGTACCGGTAAAGAAGACGGGGAAAATTTTGCACAGATGCAAGACAAAGCCAAGCTGACCTCCTCCTGATCTGGAACTGTACATAGCATAAAAGAAAGCACAGGGTTAGTTTAGTACGTAGCACAATTCAAATATATATTGACAAAGAAAAGAGAGAGAAAGGGCAGGATAGAGGCTGTTAACTCGAAGATATTGATTGATGATAGACATGGTATACGTGTACACAGTATAGTTAGGTACAGTACTATAGGTAGTAGTTATATATATATGAATCAATGAATATACGAGGAGCCACATGCTGCCATTCTGAAGTCTGGACGTGCACTCTTCAGGCGTGTTCGTGACTGTCCGAGCCTGTTTGGCTGGTGGTTGCCCATGTGAGGAGTTAGTAGCTAAACGATCCATGCCGTAGCCTGAAGGCAAGGCAAGTTGGAGGACAGGAGGAGACGTCACACGTGCTCTTCCATGTGAATCTTCCCCGTCCGTGACGCGTGTGAGCGTCCACGTCTCTGCGGCCGAGCCGATTCCAGGCCTTGTCTAGTCCTAGTGCACTGCCCAGTGGCCACCGACGACCGACGGTTTCGCTTCGGCATGGGTTTGGCAACTTGGCAAGTCATCGCGGTGGAAGTTGGAAGCTCCCCGTGCTCGTCCTGTGGCCTTGGTGCGAATCCGTGCGTGGTGCGGCGCTCGCGCGGTCTCGCACCAAGGGTCTCACACCGAGGGTTCCAGGTAATTTTGCAATGCGTTGGGCCTGGCGTCAGTCAAGGGCCGGCTGGGATCGCTTGGACGAGTTCAGCCTGTGCCGGATCGGATCGGATGGACCGAAGCCGCTGGACTCAAAGCCCATCAACAGCATCACTACCAAGGCAGGCAGGGCCACACGCTTTGGACCGCCACACTAGGCCATGCAACATAGCCCAAGGAAAGGCGTGGGCACCTGGGCTGTTTGTTCGAGCCTACACTAGCACTAGAAACACGCGCGTCCTCATGGGACAGAACCAGTTGTGTAGGAGGAGCATAGGTCAATTATTGATTAATTTTTTAGCAGGCAGGGTGTCTAGTATTTTGAGGCAGTTTGTTATGTGTTTATGTTGCTACGGACCCTTTTTTTTGTGAAACAAGGTTTTATAATTTGGAGATTTGGCTACAGTAGGTTTTAGCGATTATTTATAGGCGTAATAGAATACGTAGTAGGTGTTATGGAGCATTCATTTTTTGTTTTGATGACAAAGAAGAGGCGGAAAATATTTAGATACGTTTTAGATTTGTTTTAGTCTTTTTACGAATTTTTTGGAATTTATTTGCAAACGAAAAGAGTCTACTTTGTGTGTTTGTAAAAATGACCATAAATTATCAAAAAAGATTTTAATATTTAGGGCTTTTTTTGGTAGAATTATTGGAGTCGGAGGTAGGATGGATAAGATATAGACTTTCTAAATTTATCAGCCAAATACGAACGAGAGGAATCTATGTTTTTTTATGACACTGAAGAGGCAAAAAAAATATCTAGGTACATTCTAGATTGCTTCTAGTTTATTTACAGGCAGGGCCACACACGCTTTGGACCGCCACACTAGGCCACACAACATGGCCCAAGGAAAGGCGTGGGGATCTGGGCTGTTTGTTCGAGCCTACACTGGCATCATCGATTCGTCGACATCGCGACGCGAGGCGTCGCTCCGGGCTCCAGCGGAGCAACCGAGCAACAAGCGAACGATGCTCTGCAAGACGTCGACGTCCAGCATGCGGGACGCCACGCCGGACCGGCGGAGCCGCATCATGAGCTCAACTTGTCTGTCCACCGATGGGCGATGGCCGGTGGGGCGACGGCGACGCGGGAGGGGAGCGATGCTCGGATACAGACATGCTGACAGCAGAGCTTGGCCGCTTGGCTTCACTATGCTCGTCTAGGCCATCTCGTTTGGGAGCCACATTGACAGACCGATTTTTTTTCCCAATTAGAAAAATCGTCAATCCCCTTGTTTTCTTTTTGCAACACACTGATAAAATGACAACCCATCAGTCTCAGTGTTTCACCTCGCACTCGGCTGCTCGGAGTTCCAATATTTAGGCACACCTGATCGGGTTCCTTGTCGATGGACATCACATTGTCACTTGGTCAGTTCATCACCAACTCCGATCCTGCCAATCTGATACCCAGCTTGCAGCGCAAGATTAATTTCCGCTCCGTGCTCGCAACAGCAGGTTCGGGAAACGAAGTTCTCCAAGCCAGGTTTTATTTCGTCCTTGATGACGGTGTTACAGAATGTCAAATTATGGGTGTTGTGACAAACTGGTGATGCGTACGGTGGATTCATCTGATCATGCAGTGAACTTGATCCCATCCCAATCAACGAACAACTGCGCGATGTGAGCTCGTGTGGTCTTGCCCTCTGATTCTCTGAATCTCAAAGATGCCTCCGTCTCCACCGGAATAAACCCGACTTTGAATGGCCATCAAGTACAATTCACCTACTACCATGTAATGTAGGAGTAACTAGCATATGACGGGCATATTCTCCTGATGCCACTCGCCGTAGTGCAGATATCGACATCACTGGAAATAAATCGCCTTATTTATTAGTTTCTAGCACAATAATGCTGATGAATGCTCCAAAGCCTGTCGTAACGTAATCACTCAATCATGGCTCCATACTAGTAGAACTGTGTCGACCACTATCACTTGTGTGATAGTTAGTACTACTGCAGAATTTCCATATCCATTGGAAAGCGAGGCCCGTTTGATTGCCTCTGTTTTTTTTAACACACATCACATCGAATGTTACGGCATATGCATGAAGTACTAAATATAGACTAATTACATGCATGTCGGCATAGATTAAGAGTAATTTACGAGACGAATCTATTAAGCCTAATTAGTCCGTGATTAGACACTAATTGTCAAATAAGACTAAAGTGCTACATTACCTGTTAAACTTTAACATGACAAACCAAACATACCATGTGCTATTGTGGCTTGTGGCGAGCTGCGTGCTTGCAACATCCCAGAGTGGCTAAATTGGTTGCAAATTTCCGGAACTCCGGGTATCAAGCAAAGTTATTAACATCCAGTATGGAATTAAAATTGAAATATTCTCAAACCACCTAAACACTTTTGTTAGACCTTTTTGCAAGGCTTTTTGCTAGCTGACGAGTAGGATTCAACTTGTTTATGGAAGCTTTGCGACAATATTGCCTTCTAAAGTTCAAAACAAATTCCGGCTGTTTACCTATACCTGATGTTGCATTTGGTTTACTCACTTTTATATTGTTTTATGTTATTATACCTAATATAATAATGAACAGAGAAAAAAAAGCAGGGATTATTATGTTGTCTGCCACTATCTAAACTCCCAAGGTACGGTGGTTCACTAAATTTGACGAATATTCAGTGGGTATTCAGCGAGTACAGACATCACATTTGTTTAACAAGTATTTATTGACATACTCTTAACAAGTATTTAGCGCTGCCATTGGTTAGATAGTTCTATTCATTCCAAGATACGTATATCTATGAATTATACTAAACTCAACTTCTACAATAGCTATAACTTATCTTGAGGCTTTGATTACTATTATTAGCGTAGGTGTGACATGGTCAGATCAGGTAATCAACTGAACACGCCACACTTTCCCCCTGTTCGGCTGAGCTTATCAGCACGGCTTATCAGCCGTGGAACAGTGTTTTTCTCTCACAGCAAATCAGCTACAGCCGACTTATAAGCCAAAAAAACAGCCTGCCGAACATGGCCTTTGTTAAAGTTAGACATAGCAAGGTTTGGTACCAACCAAAGAGGCTAAACTTTAAACTGTGACCTCGCAAGTCTAATCTTTTTCAGACCAGCGAGCAACCAGATTCGAGTACACGATTGCACAAGAGCCCTTTGCTCTGGCTATAACTTATGACAATCTTATGGCCAAAATCGTCATATCCTCCCCACACTACCCACATATAAATCAGAATATACAAGCATTATGACCACACAACATCCTATAATTACAGTTGCAATTCCAATCCTGCCCTCCTCCGCCGCCACCTCGGCGACGCTGTCAGGTCGCCGTCCCACAGCCTGACGACGCTACCGTCGAGAGCTGCCATGTCGATGTGGCGGTACCAACCCAGGTCGCTCCTCCGCCTCACCGCCAACTCCCTCTCTTCCCACGGCGCCGTGGCCGCCAAGGTCGTCGCTCTTCTTCTCCCGGCCGGCTCTGACACCGTCCTTCTGAGAACCACCTCCACCTCGCCGTCGTCGTCGTCATCCGCGTCCGCGCATTTCCTGACGCCGTCCACCACTCCGTCGTCGTTGTCTCCTGATGAGCCACCGTAGTACGCAGTGAACCGCACCTTGGATGGCGTGCCCGGCTCGCTCACGGACGACGACGGCTCGGCCGGAGCGAGAGCGACCGGCCGCCGCGGCTCGTCAGTAATGGCAGCCCGCCGAGACGGCGGCAGACGCGCCTGTGGCTCCTGATTGTCGTCGTCGACGAGGGCGTTGGCGCTGCGAACGCCGGCGGCTCTGGGCTTGGAGTTGGAAGCGCTGTTGGCGCGGACGCGCCAGAGGCCGACAGCGGCGGCGGCGAGGGCGGCGACGAGCCAGGCGCAGAGCGAGCCGGCGGCCGCGGCCGCCGCTGGGAGGGAGTAGAGGCGCAGCGCAATGGCCTCCGCGGGCACCATGCCGAGGAGCTCCATTCGCGGAGCCGCGGAGGTGTGATTGTGCTGGTGCCTGGTGGTGGTGGGGACTGGGGGAGGTACTCGGCGGTTGTTGAACAGGGGG

>BAC-84

AGGTAGTTACGCTTGAGGCTTTTGACCCCGCCCGTTCCCTTCCTCCTCGTCGCCGCCTCCCCTCCTCGTCGTAATCTCCGCTCCAGTCCCCCACCGCCCGGAAGCCGAGCCCGCCACCGCATTCCAGCGGGCATCGAGGCCACGGCCAGCACCCACCTCGCTCCATGGTGGTGCGCGATACCTAACCCTACCTTATCCTTGACCTTACCTCACAATGGCCGCCGCGGCGGAGCGCCGAAAGCTCAGCGGCTACCTACGCGCCGTGGTGTCCGTCCCCGGCGGAGACGTCGCGGCGGCGGCGTCGATCCCGCCGCTGTCACCGTGCACGCTCTCCGCGTGCGGGGCCGTGGCCCTAGCGCCGCTCCCCGACGACGTGGGGACCCAGCCGCGGTGGCCCAGGTGGCGCGCCCCCGGCGTCGTGCGCCTGCTCAGGACACTCGTGGCGAACCGCTGCGTCGAGGTGGAGGGCACGCTGCTGCGGGTCGTGACCAGGAGGGCCGGGGAAGGGGACGGCGACGGCGACGGCGCCGAGGTGGAGGCCAGGGCCGTCCTGCTGATCGACGTCTACCTGCCCGTCGCCGCGTGGTCCGGGTGGCAGTTCCCGCGCTCGCGCTCCGCTGCCGCCGCCGTCTTCAAGCATGTCAGGTGCGTCTCCTCCGTCTGTGTCTATCTGAGGGCGGGGCGGCATGTGCCCTGCATTGTGCACCCAAATTTTGGAGTTCTGACTGCGTAGGGGCTACTGATGTTTATATCATTTTTATTTACGGGGCTTATCAAGGTTATAGGTCTCTTAAATGTTTGATAAAAAAAAATCTGCAGTTGCTCTGAATCGATAATCTGTTTATGCTAGGAATTTAATTCTGGATCCTTCTCGATTGTATTCTATATGTTCCTTTCTGGGACTTTCTGTATAACGTAATTACTACTAGCCAAGTGCCTGTCGGTTGCAAAGGATTTCCTGATCTAGAGTTCCGGGCATATGTGTTTTCAAAACTTTTCAAAACAACTAACAGTTGAAATCTGGAGAGCTGGCCCTAGGAAATCTGAATAAATAAAATTTGCACCATTAAATGCTCACCTCTGAGGCTCTGAATATGAACAAACATGATAAAAGCATGAGCTTATACATGAGGTGACAATTGAATACTACTAACTAGTGGTGGACGTTCCAAGAATACAGGCATCTTCCTCACTAGATGACATGTGACTATCCCGCATGTCCCTCTCCTCAGCGAAGGCAACAGCAGAAGTTAGGGATGCAAGGGCCATTGGCTGGTGAGACTGGGGGACGTTAGCAATTGAGATGAGACCAGGAGGGCATGGGTCTGAATGGATTAGGCCAGCTAGTATGTAGGACTTATTGTTTATTGTGTGTATAATCAGATGGAATATTCAGAGGGGAAAAACTTCAAATCATAATGTATCTGTCCTTTCAACCGTTCCAAGCATTCCTGATATGCCTGAAACCTTGCAAAGGAAGGCTTTTGATATAATACCCTGTTTCTGGAATGATGCTGCTTATTATTATTTCTTTTTACTTTTTACTATTTGGAGTTTGGACTGCTATGGTCACTTTCTGGTCTTTTTCCAACAACAAAAACAACCAAGCCTTTTAGTTCCAAGCAAGTTGGGGTAGGCTAGAGCTGAAACCAAACACGAGCCATCACAGATCACGAAAAGAAAAGCAGTTTCTGGGGCATATGCCTGGGCAAAAATATTCTGGGACATTGGTTATGCTGCACGTGTACGTGGGGTTGACCTCTTATCTGGTTATCCCGTATCAGACACGTTTCGCTGTATATGTATCCGCCGATTATTGGCGGGGAAATAAATATAAGATAATTCCATGTGTGACCTGATACTTATCCGCGCACCCAAGGGAATTCGCTATCATGGAGGGAGGGAGAGGAGAGAGGAAGAGCAGGAGGAGAACGCAAGGCCTCACCTACCCTTGCTCCCATCCCCGTCAGCTCTGGCACCTGTGGCATCGCAGCTTTCTTCACTGTTCAACTGTATAGCTTGATCTAGAAGGGGGCAGCACTGGAACTTGATCCACATACAAGTAGACCTCTATATGGAAGGGGGTGGCACCGGTGCCCAATTCGCTGCTCCGTCTCAGTGAAGTTGGTACATCCAGTGAGGACAGTAGATTAGGGATTGAGGTGAGAGGGGAGGGGGTTGCTTTATGGGCTATTGAGTTGATTGGACTCTACTGAGTTAGTGGGCTGGCTGCTAGTAGTACTGTGGTCTATTAGTGCTCGTACTTTCCTCGTATTCCCTATTGTGGATGTACTCTCTTTTACATATGTCTGTGCCATACATTATTTATTTTCAAAAATATATTAAAATGTATCAGCATATTAGAGTTTTTTTTTTGGAACATTGGTGTATTTGTATCCATCCAATACTGATACACATATCCGTGTACTACCTCCATTCTCATATGTATGACGCTGGTTAGTTCAATTTCCAAATTTCAACTAACCGGCATCAAACCCAAAAAAAGGAGGGAGTACATAGGATATCAGTAAGTTATTCCCAAGCATTTTAATTTTGTAATGGTATTTTTTGTCCCCTGATTCTTGAGATCTTGTAAATGGCTATTAGTTGCTTCCCTTCTGACTATGTGATGAGATCTGGTAAATTGTAATAGGTTCCACATATTAAAGGTGTTTCCACTACCTGATTTGTTAATGCCAGTTCCTCTACCACAAATAGATATGGCTATTTTTTTCCATATGAAGTCCCTATTCAAATTTTCCATCCAGCCATGAATATTATGTGAATTTGAGCAAAACTGCAATTGTAGAGTTTGTTAATTTTTTAATTCGTTATTTCCAAAAATGTAGTTTCAGAGACCTAAGATATGTCAATCCTGACAAATATTCATTATGTCAGTAATATTTCCTGTGTTGTTGTCAACATTCCCGAGTTTAACCCCCCTTTTTTTGACTGGCAGTTGCAACTGGGATGCTAGGAAAGCTTTGCTTGATTTTGACTGGACTTCTCATGACAATACACACTGTGATGACCGATCCATTTGGAGCTGCACCGATTGTCACGTGCTTGGTTGCGAGGACCATAAGATAGCATCAATATCAAATAAAGAGAGGTCATTTGACCTGCATGAAATTTTCAAGACACTCCCTGGTGTTAGGATGGAGAAGAACATGCAGGTAGCAAGAATAATACCAGATGCAGGAGCACTGGAACTGGGTATTTGGTCTCTCCCTGATGATGTATTGCATAAAGTACTAATTCTACTTAAACCCAGAGATTTGATAAGAATGGCAGCAACTTGTCATCATCTAAGGACTCTTGCTGCCTCTGTTATGCCTTGCATGAAGCTTAAGCTCTTCCCCCATCAAGAGGCTGCTGTCGAATGGATGTTGAAGAGAGAGCAGAATGTGCATGTCCTAGCACATCCTTTGTACAAGGATTTCTGCACTGAGGATGGTTTCCCATTCCACATAAATGTTATCTCTGCTGAAATATCTACCGGTGATGCTCCAACCATAAATGATTTCTGTGGAGGTATGTTTTGTGATGAGCCTGGATTAGGGAAGACAGTAACCACACTCTCTCTGATTCTGAAAACCCAAGGAACAATGGCGTACCCTCCACATGGGGTGGATGTGAGTTGGTGCATGCATAAGCCAGATAAAAAATGTGGTTACTATGAATTAAGTGCCAGCTGCTCTTCTAATAGAAACAGCTCTTCGTCTGTGTCAAAAAAGCTTTTGGAGGAGGATGTGATAACAGATTATCCATGTCCAGATGATTCTGTCTGCAGTACCAGATCTTCAAGAAAGAGAGGTAGGTTACTGAGCCCTGATCCAACTAAGGTAATGTTGCATGCTGCGATTGAGAATTCCCCATCGTCATCACACAGCAAAGTGCATTCAATGCCAGCTACGCATATACTTAAGTTCACTAAAAACTCGAGACAAGTTAGGAAAAACCTCATGGATGCATACAGCGACGTTTCAGTTGGCAATAAAAGGAAGATTGGCACCAGCTCTGAGTTAAGTGAGACCTGGGTTCAGTGTGATGCTTGCAGAAAGTGGCGAAGGTTATCCAATGGAACAGTTCTTGATTCTACCACAGTGTGGTTTTGCACTATGAACACTGATCCTACACGACAGAAATGCACTGCCCCGGAGGAATCCTGGGATTTTAAGAGAAAGATAACCTATTTGCCAGGATTTTACAAGAAAAATTCTTTGCCTGGAAATGAAGAAAACGTGACATTTTTCACAAACATATTGAAAGATAATGTTACTATGATCAATTCAGAAACCAAGAAGGCTTTGTTATGGTTAGCAAAACTTTCTCCCACAAAACTTCTTGAGATGGAATTTGTTGGTTTGACTCGACCAGTTCTAGATACACGTGCAACTACTGGCAAGGGTGCTCGTCCATATTACAAGATATTCCAAGCATTTGGCCTTGTGAGAAAGATTGAGAAAGGTGTCACTCGCTGGTACTATCCCTCTATGCTTGATGATTTATCTTTTGATTCGGCTGCACTTGGAGCTGCTCTTGAAAAACCTCTGGATTCAACTAGATTTTATTTATCTACAGCCACCTTGATAGTGGTGCCTGCTAACTTAATTGATCACTGGACAACACAGATACAACGTCATGTGTCATCGGATACCCTTAATGTTTTTGTGTGGGGAGACCACAAGAAGCCATCTGCTCACAACCTTGCTTGGGACTATGACATTGTCATAACCACATTCAGCAGACTAAGTGCAGAATGGGGTCCAAAGAAGAGAAGCCCACTAAAGCAGATCCATTGGTTCAGGGTTATACTGGATGAAGGGCACACACTAGGTTCCAGCCTTGCCCTGACAAACAAGTTACAGATGGCTGTCGCTTTGGTTGCATCAAATAGGTGGATATTAACTGGTACACCTACACCAAATACACCAACTAGTCAGGTTGCTCATCTTCACCCCATGCTTAAGTTTCTTCATGATGAAACTTATGGTGAGAACTACCAGTCATGGGACTCTGGGATCCATAGGCCTTTTGAGGCACAAATGGAAGAGGGACGTGTTCGTCTTGTGCAGCTGCTTCAGAGGACTATGATTAGTGCAAGAAAAGCAGACCTGAAAAATATTCCTCCTTGCATAAAGAAGATAACCTACCTAGACTTCAATGAGGGGCATGCAAAAAGTTACAATGAACTGGTAGTTACTATACGCCGAAATATACTGATGGCTGATTGGAATGACCCTTCTCATGTAGAATCCCTTCTTAACCCGAAGCAGTGGAAATTCCGTGCTACTACTTTGAAGAATGTCCGTTTATCTTGCTGTGTTGCCGGGCACATCAAAGTAGCCGAGGCAGGTCAGGATATACAGGAAACAATGGATGAATTGGTGCAGCATGGCCTTGATCCTTCTTCAGATGAATATCAGTTGATAAGATATTCTCTTTTAAACGGTGCCAGTTGTGTCAGGTACAGGATCTTTTGTAATAAGATCATATATGTATCAATCACAGTATTTCCATTATTCAAATATTCATATTGCTTTCAGGTGTAGAGTTTGGTGTCGCTTGCCTGTTATCACACCCTGTCGACATCTTTTGTGCCTTGACTGTGTAGCTCTTGATAGTGAAAAATGCACGTTACCTGGTTGTGGTAATCATTATGAGATGCAGTCTCCTGAGACTCGTGCAAGGCCAGAAAATCCAAACCCAAAGTGGCCAGTGCCTAAGGATCTAATCGAGTTGCAGCCTTCATATAAGCAGGTCCGTGCTTGTGCTATATTGTCCATTTTTATCCACTTTGTGTGCTCTTGAAAATAAGTTTCTGTAATTTTGTAATTTTTAGGATGATTGGGATCCCGATTGGCAATCAACATCTAGCAGCAAAGTTGCTTATTTGATTGAGAAGCTAAGAAGTCTGCGAGAAACTGGGAATAACATAACCAATAGCGTTGGTCATGCTAATACACCATCTTATCAAACACAGGCAATGTTTGACAAAGTTATAATTTTTTCTCAGTTTCTAGAGCATATTCACGTGATTGAACAGCAGGTTAGTTTGAAATGCTTATACTCTTGGTCTTTCTTTATAATTATATGAAATTAGTTGACGATTGATCTCCTTTCTGCTCAGCTGACTGTTGCTGGAATAATATATGCGGGAATGTATAGTCCGATGCCTCTAGCTAGTAAGGTATCTCAATTGCTGATCTCTAAATTTCTGATCTCTACCATTCTAGTATCTCAATTGCCTCCTAGTTCCCACTACCCCTTCACATGTTACTCCGTACATTCCAAATTATAAGACGTTTTGGCTTTTCTAGATCCGTAGCTTTTGTTATGCACTTAGATATACACTGTCTAAAAGCAATGTTCAAGAAAAGCCAAAATGTCTTATAATTTGGAATGGAGTACAAAACTACCTTCCTAATTGGAAAAAATACAAGTAAACTCTCCTTGGCAAATATCTGAAACATATTTTAAGGTTTATCTCGATGTTACTGCAATTCTTTGCAGAGAAGTGCACTAATGAAGTTCCAGGAGGACCCAACGTGTATGGCTTTAGTCATGGATGGAACTGCTGCACTGGGGCTTGATTTGAGTTTTGTGACTCATGTTTTTCTCATGGAACCAATATGGGATAGGAGGTAAATATAGCTAAAATGCAGCTGCATTAGTTCCATTGTATTTACCAATCATTATGGTTCCATAGGGTAAATATAATGTGAAGTGTGAATTTATTGGCCAATGCTGTGCCCTTGTCATTTTGTCCTACTTCTGACAATGATATTTGCTATCACTGTCATTATCATGTATCATCTGTAACTGACACTTGCACTTTCTTTGTTCTGATCAGTATGGAGGAACAGGTTATTAGTCGGGCACATCGAATGGGGGCTACGCGTCCAATACATGTCGAAACTTTGGCTATGCGTGGTACTGTTGAAGAGCAAATGCTTAAGCTTCTGCAGGTTTGTGTGCATGACATGTTCATGACATTTTGATGCCAAATTATTGTCAAATTTTACATGTGGTTGAGTGCTTTATTTGTCTGCAGGATTCTAGCGCCTGCAGAAAAATAGTGAATAAAGGAACAGGCAGCACTGACAATGATGGAGGCCGGTCTCATCGGAGCCTCCATGATTTTGCTGAAAGCAGCTATCTGATGCAACTCAGCTCTGTGTAGACAGGATCTAAAGCAGCAGCCCATTTTGGCTGTCGGTAGCACTAACTAAATTCTTGATTTTTCTGTTTCTTCTTTGATCATTCCCTTTGCAATAGTAATAAAAATTGCTTGGAAGTCAGTAGTTCTTGATTCCAAACAAGTGTTGAGGATTCTTTCGTGTAAATAGCTCCAATTCCTTTTGTTAAACGATGTGTTCCCAGTTCAAGAAGCCTTGTTCCGAGCATTATCTGTGACGAAATCTCTATCGGCTGCGTCATATTAGCCAGAACTTCTGTGTCAGCTGCGAGACTTAGTTTTTAAACCGTGGAAGAACTTCTGTGTCAGCTGAGTCATATTAGCCGGAATTTGGACTCGAATCCTGCTCACGGCCATGTGGACTCAAATTCTGCTCACGGCCGTGACTGAACTTATGATTGGTGTATCGCGCATGTTGTTGTTAACCTTATGAACAATTTAAGTTGAAGCTAACGTCTGGAGTCTGGATAATTATGTAAGGTTTAGCAGACACCTTGTCTTATACTGTCACATCTACGGGTCCACCCCCATTTTGCTCTCGTCTGGTTAAGCCAATCAGTTGGGACTTCCGACTTCCAGTGTTGACCCCTCGAGACGTTTGCTTCAGATTTATGCATACAGAATCTCTGAGCTGCAGGTCTACTACAAACGTCAGAGGCAGCACCACTTAGCCCAGATTTGCCATTTGCATTTGCAATCATATCGGAAAATTTCCAAGACATTCTAAAATCGATGCTTGCTTATGCATTTACAAGATAGATTAGCCTGCTTCTACTAAGCATTTACATTACCTCTGCTCAAGCACTCTACTTTTCACACAAGGAGAAAAGAGAAGGGGGGAAAGAGTAAAGAATTATGTAAAACCGAAGATAAAAAATCCTTCCTGTTTTCCTGGGAGCGGAAACCACCTCTTCTCAAGGGGCTACGTAAAATGGTAGCCACGTTAGCAACCCAGCTTGACTGACGAGAGAGCCTTCACGTAGGTAGGGTACATGGCCTCAGCTCCGCTTGTAATCAAGTGTTTCTCCAGGTCAATCTAGTGTCAGTTAGCCGCAATGTCATTGTCGACACTGGCAGTTGATTTGTCAACCACACGGACATTGTACTGCTGGTATACCCTCGCCCGGTTCTCCTGCCACCACTGTTCTGCTTGTGTCTCACTGAATCTCTTCCGGCTATATTTACAGAAAAAGAAATGACAAGTCAAGAGCTAAACCACATGAGAATTCAACAGGAACACGATGTAAGTTGGACACTAGCTATAGAAGTTGCCTACTGAGCAGGATTCAAATGCATCTACCTGAACCGAACCCGCTTGAGATCTCTGGCGCCTCCAGTCAACGCAGTGAGAGTAATGTAAACACCTGGTTCATCTTGCTCTACCCATTCAGCATCATGGCAAGATTCAGCATCAGGCGGCCTACTTCCATTCTTGCCTACTTCTGGATGGCTCGTCTTATTCCTAACAGAACTTGGTCCATTAGAAGCAAGCAATCCATTATGACCATTTATGATTTGTTCTCCAGACCTGCTTGGGCTGCCCACACTCTCCGTAGCCATAGACGAAATGTCACTTGGAATGGAAATTCCAGGAAGAGGTGGCAACTTAGTGTTCTTGGCTGCTCCTCCAGGTAATCTCTCTGCCATCCCCTTCAACTGTTACATAAAGAATGGAGTTGGCCATCAGATGATAGCTGTCATAAGTGGCACGAGTTACAAAGCTCTTTTCCCCCTTTATTATTAGCCATTAGCCATGAATATACAGGGCCACATTCAAATGTGGAACCTTATAGCCAATAATTGATTCGAAATTGTTCAAGCCTTCAGATTTTCCATTAACAAACTGTTTTATTCAAGTTTCGTATCCTGGTTGTAACATACTAACATGGAGCTATTTAATATGGGACAACCTAGCTCAACGGACTTTATCCTAAATGCTAATATTGAAATTATTCTAATAAAATTACATGTGGGCATAAAAATAGAGACAAAATTGCAAGCCCATACAGTAAGCAGACTTACCTGTGCAGTGAGTGACTTGATTACTTCCTTTGCCGCCTTGCACTTTGCAGTTTCCTCACCGGCAATGGAAATAGCTTCCTTCAACTGCTTGGTAGTCCTGTCCAGCTCCACTTCTAGGAGTTGAGACTTCCTGGTAAGACTCTCCACCTGTAAGTCAAGAGCTAATTACATGAGCAGCAAGACCTCTCGTTCTGATGATACTTAGCTTTAAGGAAAAGGGAGGTTAAAATCTTGAAACTCCAAAGGACAATATGTTGTCTTCCATATTAGTCAAGTAGGCAGGTAACAATCTGCTAAAATTTAATGAACTTCCATTCTCCATACTCTGTTACTCTTACAGAACAAAAGGGTATATATGTCTACAGTGCAAGGTTGCAGGGGGTATGCAGAACAAAAAACGTGTAAGCTATCTTTTAGAACTTAGAACCAAAAGTTCAGTATAACAACACAAAAAGCACTGCTGTCTATCGAGCGTAGTGTCAGCTAAAACTCCACTAGATTTAAATTGTCAACTACTGAAAGCAAAATAAAATCACTATATCAATCAAATGACATTAAAAATGTGGAGCAAAATGTGTACCTGAGACCTCAAATTCAGAACCTCTTGGCTTAATGCATCATTTGTTGGCTTGCCATCATTGGGAACAACTCTTGGAGATGTTAGACCACCCAGAGTAGGTGTTGGTGTTGTAGATCGTGGAGGGCTTGCTCTTCTTGAAATAGGTGATGTTGCCCTAGAAACAATTCTAGATCCAGGAACTGATGCTGAGAAAAACTTCTTTGAAGATCCAAATACAGGATTGAAAGATCTTGAAATGTTTAGCCCACTCCAATGTGAACTCCCATTAGGCACAGGGGAAACACGGGTGCTATTAAATTCAAACTTCTTATTTTTCTTGGAATATCTGCTATCCAAATGCTTAAAAGATTCCATTGATGACAACCTTGATAGTTGAGCATTTGACCTTGTTTCCAGATCCTCCTCAATTGTATCACTGAATCCCGGTACAGTAGCAGCCCGCTTCGCAGAAGAATGCATATCTGTCTCAAGTCCCTTTGTCAGTTTGCTGTAGCAGGTATCACAGACACGGTAAGGCTTGTTTGTATTAGGTGCTAATGAAGCCTTCAGAGATTTTTTACTGCTGCAGGAATGACAAAATACTAGAGCACAGTTGTAGCAATTATGACGTTTTCTCCTCAAGTTGAATGGCTGACGGCAACCTGAGCACATTGATTGATCAACTCCAGATACCCATTTGTGAATGCATATTGCTGCAGTGAAATTAATTCCACAAACAACACTTCTGACTTGCTTATCTTTCAGTGCTTCAACCAATGTAGGAGTATTCCTATCATCTGTATCGCCATGACCTAACCGGCCATTTGCACCTTTTCCCCATGTGTACACCTCAGTCCTAGAAGTCAATACAGCCACATGATAAGCACCACATGAAATCTCCTCCACAAAGTTTTTGTGTAGCTTCCCTTCAACACGCACAGGAAGCATACCATCAGCTTGTGCATTCCCAAGTTGACCATAGACAGCACTGCCCATTGTATACACATGCCCAGAAGTTGTTAGGGCTACTGTCAAGCAATGCCCACAAGCAACCTGACAAAAATTGGGCTCCACCAAAGCAGCCACACAAGTTGGGACAAGTTTTGGTTCCTTGTCACCATGACCTAAGCGACCTTTATCACCATCACCCCATGTAAATATCTTACCAGAAGAACAATTGCTAGAACTTGAATTCCCAGCCATGACTTCTACAACTGCAGCAGTGTGCCAAACACCACAAGCTGCCCGCACCGTGCGTAGCCCTTTGAGAGATTCAACTTCCCTGGGGACCGAGATACTCTCACGATCTCCATGGCCCAGAACCCCAAAAGATCCATCACCAAATGTGAAAAGCTGTCCAGCAGAAGTTACTAGGGCAGTATGCCAAGGTCCACATGAAATTGATGAGACATGTACACCCTCTAATGGTCCATTCACGTGCTTTGGTACCCAGTGACTGACATCATTTCCATGACCCAAAAGCCCAAATTTAAATGTGCCATCCCCCCATGTGTAAAGATCTCCTGATAGTGTAACAGCACAAGTATGGTATTCGCCACATGCTACAAGCTCAATGTTCATATGAGAAAGAGCATCAATAAGTTTTGGCTGCGACACATCACAATCAACACCATGACCAAGCCGCCCGCCTGATTCCTCACCCCATGAGTATATCTCACCTTGTTTAGTAACAAGTGCAGCATGCCTTCCTCCACAAGATATATTCTGCACATCAAGTCGCACGGCAAATTCTAATGGTTTTGGTACAAGGCAGTCCATTTTTGCACCTGAGGAGCTTCCAACTCTTGAACCACCACCGCCAAGAATTCCTTCCCCGGTTCCTTCTCCCCATATGAAAACATCGCCTAGAGCATCACCGTCATCATGACCAGAACCATGACTAGATGAACTAACAGCACTTGAATAACTGACTCGGAAAGCATCCATTGGGACGCCTCTGGGGTGCCCATTTGTGTTATCCGAGTGTCCAGAAGACATAGAATGGACTGATCCAGCATTAGAATCTGATGGAAAGAAACCCCTGGGAGGAACTGCATACAACATGACATCAGAAAATGCCTTCTCCAGTCCATTCTTTGGTGGGCTCCCATATGGAGTACGGAGTCGGTAATTGTCACTGCCATCCTGATATAAGAGATCATTTTTAAGAATAAATGGCAATATCACAAAAGAGGACATATAATGCAAAGTAATGTGAAGGGAAGGAGAATCATTAAAAGTAATTAAGTACACTAGCAATTTGACACAAAAAAAAGTCTTCCCTTTCTGGCTTTGCACTCTAGTGTCATCAAATTTCTGGCAAGGCTTTCTGCTCTAACTGAACACATCAGTGAAAACAAGGTGCTTCCACAAGTATTTGAGTGGAGAAATTGCTGATTGAACTCGCAACATGAAAACAGGATGGTTTCTAACTATGAAGAGCCTTCCCAGCACAATTAAGGGTTGGAACTCGAATGTTTTATTCTAGTCTTCTAGTCATCTAGGTAGGATTTTAATACAATGGAGACAGTAATACCTTGTGGATGCTGTCATTGCTGCTGAAAGGTGAACTCAAAGGAGAACTCCGTCGTGTGTAAGTCCTCGGACTAGTTGCACCAGAGGAAAGGATATCACTTCTAGATTCAGTTCTCCATTTTCTTTGGTGACAACGTGAGATCAGTGTTTTCAGCCCAGCAAACCATACTTCAGCTTCATCTTTATCTTTGCATATCTGATATTAATGAACAAAACAAATCATGTCATCATGTGAATACAAAACTCTGAGTAAGAGGGGGGTGGGGTGTTGTAGCAACAGAGGCAACAAAGACAAAACATATAAAAATATGAAGCAGCGATAACTTACTATATCCAATGACCTATCATGTGAAATTAGAGAAAAAGACTGGCATTCCTTCTCAGGCCGTGGATACCTCTGAAAAATTGCCTGAATTATTCAAAAAAAGGGTCTAGTTAGTAAAGTCAGACAACCAAGTTCATCATAAATGGAAAATGGAAATACTTCTAAGAAAACATATGGCTTGTGTTCATTAACAATGGGATTCAATCGTATCCTGACACGAATTCTAAACTCCTTAGTGTAGGCGTCAAAAGTAGCAAGACATATATACATATAGAAGAGTTGTTCTAGTTATTTTTAATTTCACAAAATTATTTATCCATCCTAACATGAGAAATAAGGACTTCGGAAATGCTATTGTGTTCATCCATATGGGTTGGCAAGCAAACATCACCAGAAAATCAAGACATTTTTAATATGAAGCTTGTTGACCAAAGCAACTTATTATTAGGGAGAAATTGCTAGACAGGATAATCTTCTTGAAAATGAGTCTAGGCCTCCAAAGAACATTAACTTACAGTCCGTTGCCCAGGAATTATCCTGGATACATGGCTCAACCTTAGATGTTTCTCTTCTTTCCCTGAGAACCATATCAGTACAGATTCATCCTGAAGGAGCCAAAAAAAAGTATAAAATACACCATATAAGCCAAAGGATTATAAAGGCACAGAAACACTGTTAAGTTGGAGAGTCCAAATAAGGCTCACATTGGATAACCTAAATGGACAAAACTTTGGCTTTCCTCTACGCCCATACTTCAGCAAGTATGCTCCTTTCTTGAGAGCAGTGATGGCCTGCAAAGAAAGATGCTGTCAAGCCTCAAATATCAACTTATCAAGTCATCAATATATGGAAAAAAAACAAGTACTAGGAGCATGCAACTTCCATACTTCACATTTTGAAGATATTTAACAGACCTGTTGTAAATCACTGGTTTTATAGGATGGCTAGGATCCGCGAGAGGAAGACAAGGGACATCAACCAAATAAAATGCATCAAGGATGGGACAGATCGACTTCTGGTGAAGGATGAGTAGATCAAGAACAGATGGAGAGAGTACTTCGACAAGTTGTTTAATGGGGAGAATGAGGGTCCTACCCTTGAGTTGGACGACTCATTTGACGATACCAACAGACGCTTTGTGAGGAGAATTCAGGAGACAGAGATCGGGGAGGCTTTGAAGAGGATGAAGGGAGGTAAAGCGATGGGCCCTGATGGTATCCCCATTGAGGTGTGGAGATGCCTAGACGTCAGAGCGATAGTATGGTTAACTAAGCTTTTTAACCTTATTTTTCGGTCTAACAAGATGCCTGAAGAATGGAGAAGTATATTAGTACCTATCTTCAAGAACAAGGGAGATGTCCAAAGTTGTACTAACTACCGTGGGATTAAGTTGATGAGCCATACGATGAAGCTCTGGGAGAGGGTTATTGAGCATCGCCTAAGGAGAGTGACACGTGTGA

>BAC-2

ATCTATATAATGTAGTTCAGAGTAAAAATTTACTAGTTAAGTACGCGTTCAATAAACTAAACATAAAATATAATGAAATATAAGTACACATGTTGTAAAACCTAAAATTTCATTAAATGTATTATAATATGGTAGGGAAACAGTTTTAGATGGACGAAAAAGTCTATTCTAATTGTGTTTTGTGTGATTTTGTTGTCCAAAAGACACTAAAAACTATATATGACAATAATTTTTTATCATATCTTTCTAAGATAAATATAGATTATTAAATTATAGATTAAGTAAAAAATTATTGTGATGTTAGGAGGGGGCATGACTCCTGCTGCCCCTGGCTCCACCCCTGTTTCTCCGTCCACTTTTTATATGCCGATGAAATAGTTCATCCTCTTTTCGATCATGCTAATCTCAACATTACATTTTTCCTTCCCAACTGGAAAAACCACAATGAAAAGTTTGGCCACTAGGTCGACCCTTTTTTTTCTATCCACCTAAAATGTTGAGTCCTCACAAACTTATTCATAACTTTCCACATAGGACGTGTTTGGCTAAGGTGCAGCTAAGGTTCCACAAGCCACACGCTACCACATGCAGCCGCTGTGGCGCCTAAGGTGCGGTGTTTAAAATTGGTGCCACAGATGTGACGTGGTTTTGAACGGGAAAAAGTAGTGGTTGCCACACATGAAGGCCGAACAAAACAACAGCTTTTCGATTTCATGGCGCGTTGTAAGTTAGGCATGGCAATGCGTGTTGCGAAACCAAACTCCTTAATATGAGTAAGGTTGAGATGGAATGGACTCTTATGTCAAAGGTCCAATCAAACGGTAACACGTGTGCGAGGGTGACAATTGAGATCAGCATGTGTTTTCTATACTACCGTACAAAAGATGAAAGTTAATTAATTACTCTCTTCATTTCAAATTATGAGACATTTTGATACGCAGCTTTTGCTATACACTTAGGTCTAGATACGCAATAAAAGCAAATACATCTAGAAAAAATAAAATATCTCGTAGTAATTTAGAATGGTCCGAGTAAATTAAAATATCTTGGAATGGACTCTTATGTCAAGGTCCAATCAAACGGTAACACGGGTGCGGGAGTAACGCTGTAGTGCAGCCGACTAACCCACAGCACGATGTGTACTAACCGGTCAGTCACAAGGTTCAGCAAACTATCATCCCGTCGGCTAAAACGGGGGGAAGCAGAAAGCACGCCGTCAATACCCAAGGTCAGGCTGGTCGGTGGGCAGCAGCCAACCAGCACGCCAGCCCGCAGACCGGCACCGCAGCGCCTCCACTCCCCGACGTGTGCCGCGCACGCGGAAAACGAGAACGCGCAGCGCCCCGAGCCGAGCCCCCTTGCTTCTCCTTTTTATACCCCTCGCCACCATTCCCTTCTCCACACTACTGCCAGGCGCCAGCTCCGAAGAGGGAGGAGGAGGACGCCAGTTGCCACTGCCGCGCCGCTCCCGCCTCCCTCTCTCCATCCGCCGAACTGGACGCCACCATGATCTCCGCTCGCGCCGCCACCGTCGCCGCCGCGTCCCCCGCCTCCCCGGTACGTTCTACGAGCGGCCTAACCACCCTTCTACGCCACCTTCACTCCGCTAGTGATTCCCGGTGCTTCGCGCCTCGCGCGGTTTTGGCATCGCGAGTTGGATGCCACCACCTGACCATTTGAGCTTGTGTGTTGTGTCTGTTTATTCTCCGCACCCGCAGTGGAAGCGGGGAGGACGGAGCGAGGGCGGCGGCAGCTGCGACGGATGCAGTACCTACAGGAAGACCGTGCGGAGGAGGGCAGCGACGGCGAAGGTGCGCGCCTTGCCGCCGAAGCGGGTGGAGGCGGTCGCCATAGGCTCCGCCGCGGAGACGGAGACGGAGGAGGTGGTGGTGGAGGTGGCGGCCGCCACCGAGGAGCTGCCCGTCATGCCCTGGGCCACCTCCGTCGCAAGGTGAGCCGTCGAATCGCGCGTGGGGGGCTCTGGCTGTCTCTACCTCTAGCATATCCAAACTAGTTCTAATTCTACCATTGCTTGGTTGCGTCTAGTCAAATCTAATTGCTTGTTCTATTATTAGTGTGCTCGTGTAGCTGCATTCTTCTGTCGTGGTGCCTTCACCGAAGACAACTGTACCATTTAGTTTGCCTTTTAGTTTAGTTTCAATACTAATACTAATTTATCGAAATGTGCTTCAGTTTAATTAAGAAGCTCCAGCCTGATATAGTGTATATATTTGTTAAATCACCATATGTCACATGGGAGTTTCTAGCAAGTTTCAGTTTGGGAATGTTTTTTTTGGCAGTGTTGTAGAATTCTGAATTTGGATTTTTTTTTGGCATCGTTGTAGAATTCTGAATCGGGTTGGAGTCAAATGAACTCATGCTGAGTTTAGGTCCAGAGAGTTGTTGAACAAAAAGCTGGGAATTGTCTGCTTTGCTTTAGATTTGTCCTGTTTCGTGCAAGTATATTCTAAGCTGGCCCACAGAGATACGAATTTGTCTGGTTTCTTCCACTACCCCTTTGGCAGTGAAATATGATGGAAATTGGTACTAAATTGCAACCATATTGTGGTAAGAGCGTGGTAATATACTACCAAATCGAATTTGTCACATAATCTCCTGTGAATGATAACCTTCGCTGTGCACAATGATAGCTTGTCAAGCATCAATATGGCCATGTCACCCAAAGAACATAATCTGGGCTCTAGAATGTTCATAGCTGGATGCAAAGCTTTTCTTTGAATATACGACCATAATTCTTTCCACTAATTAAGTTTCCTTCACTTGGTTGAAAAATATGCTGAGTTGTCTTATTTCTACATCAGCGGTTACACCCTTTTGAGGGATCCACATCACAACAAGGGTCTTGCTTTCACGGAGAAGGAGAGGGATGCACACTACTTGCGTGGACTGCTTCCTCCAGCAGTTGTCTCTCAGGAACTCCAAGTATGGGGCACAATAAACAGTACCTGTTCTTGAGCTGACCTTTCTTTCTTTCTTTTTTATTTACTGAAGATCACTCTAATGTATATGTAGATTAAGAAGTTCATGAACAACCTGCGGCAGTACCAGCTCCCTATTCAGTGCTATATGGCCATGATGAACCTTCAGGCAATCCCAGTTTATCCTTTCTACTCTTATATGAAAGCTTGCGACGGCCAGCATAATTGATATGAGATGTTCAGTTAGAATGAATGGGTAGTTGCATCATTGTGCTTAACACCTCCTGTTTTCAGGAGACGGACGAGAGGCTTTTCTACAAGCTTTTGATTGAAAATGTGGTGGAGCTGCTTCCTTATGTTTACACACCAACTGTAGGTGAGGCCTGCCAGAAGTATGGGTCCATCTTTGGACGACCACAGGGTCTGTATGTCAGCCTGAAGGACAAGTATGTGCGCCTATATTTGCTTGAACTTTACAATAGTATATAGCTGTCTCTACCATCTACCTATTTGATCTGGACTGACCTCGAGGTGCATTCCAGGGGGAGGGTCCTAGAAGTTCTAAGGAACTGGCCACATAGGAATGTTCAAGTTATCTGTGTTACTGATGGTGAGCGAATCTTGGGACTTGGAGATTTGGGTTGTCAGGTATGATTGTCTTATTAGTTGCTTATTACATGAGAACCTTTGCTTATATTATACTGAATCCTTCTATAGGGAATGGGAATTCCTGTAGGCAAACTTGCTCTATACACTGCTCTTGGAGGAGTTGATCCAACAGCTGTAAGCGTTCATATCTTTTCTGTCCCCTTTTTGGTTGCATTTCATGTAATGGTCATGTATGTTTGGTACCAACAAACAACTAAGTTATCACAATTGGCGAATTCTAAATTACGTTGCAGTGTTTGCCTATCACAATTGATGTTGGCACAAACAATGAGAAACTGCTTAATGATGAGTTCTACATTGGACTCCGGCAAAAACGTGCAAGGGGCGAGGTATGTAGTTCTGTCGAGATTGCTAGGTGTTAGGAATTGGCAAAATATGTGACACTTGGGATCTTATCTGTGCATTTTCCTTAATGGCAGGAGTATGATGAGCTTATGGAAGAGTTCATGGCTGCTGTTAAGACATTCTACGGTGAGAAAGTCCTCATTCAGGTGAGTTAGTCTCAACACTGTACTTTTGCTATGCTGGATCAATTTACTCTTTATCCCAAAATGTAACACTGTAAATTTTGGACAGTTTGAGGACTTTGCCAATCATAATGCCTTTGATTTGCTTGAAAAATATAGCAAGAGCCATCTTGTTTTCAATGATGATATCCAGGTAAATAGAACCACCCATGGTATTTTTATTTTTACTGATACCAAGGGGCAATCTTAATTAATTGAGATCACCATCCTTCAGTTCTTTGCCTTTTGTGATTGGTACCAATTTGGAAATATGAATGTTTGCAGGGCACAGCATCAGTGGTCCTTGCAGGTTTGTTAGCAGCACTCAAGATGGTTGGTGGGACCCTGGCAGAGCAGACTTATTTGTTCCTTGGTGCTGGGGAGGTTAGTTAAAACTTCTCCTGGTGAACTGTTATTTCCTTTGTCACCCAACTGTCGAAGCATACTATGTACTAGTATCTTTTGAATTGAGGTTGTGACCATTAGTAAGATTCTATTTTATCAAGGAAAGTAAACCCATCAATAGGATTCTGATAATGTATTTGATTTCAGGCTGGAACTGGTATTGCAGAACTCATTGCTCTTGAGATGTCGAAACAGGTAACTAGCTTCATTGTTCTAGTATCTGAATGTGTGGTAGGTTTCCTTTGTCCTAGTTCTTACTCTAACATGTACTCGTCGGGTCGATTTATAGACGAAGGCCCCAATTGAAGAGTGCCGCAAGAAGGTTTGGCTGGTGGACTCAAAGGTGTGTAACCAAAGGAAAATGCGCTGTTGCTAGTGCAATGTTTGCTCACCTGTTTATCTGACGTCCTAACATGATATTTTGTATACAGGGTTTGATTGTTGACTCTCGTAAAAACTCCCTTGCGCCATTCAAAAAACCTTGGGCACATGAGCATGAGCCCTTGACAACCTTGTATGATGCTGTTCAGGTAGTCACGCTCATGTGCATTTAAGATTGTACTTCAGTTCATTTAGCAGCTGCTGCCTTCATGTCCTGTTTGTGCTGTTTTCATAAAAAAAGAATTTACTAACCTTTCATCATATCCTCCAGTCCATCAAACCTACAGTTCTGATTGGGACATCTGGAGTTGGAAGAACATTCACAAAAGAAATTGTTGAGGCCATGGCTTCCATCAATGAGGTAATTTTTTTTCTGTTTTCAGACATTGTTGGTGGTATATTGTGAGGATGGAATGCTGACCTCTGATATTGCTAATTATAATTGCAGAGGCCTATCATCTTTTCACTGTCAAATCCAACCTCGCATTCTGAATGTACTGCTGAACAAGCATATACCTGGACTCAGGTGAGCTGTCTAATCTTTTTTTTTTTTGAAAGATCCGGTTGCCCGGCTTTATATTAATAAGCAAGAAATACAGTTGCGAGGCTACACGCGCGAGGAGCGCTTCCTCACTTGGCAGACCACCCCGTGAGGATGTACAGATCAAAAACTAATTACTCGCTAAAATGATGAGCAATCCTTAAAGATCTTAGGCCTCAATGATGAGCAATCCTTAAAGATCTTAGGCCTCTTCCCCCAGCCGAGACCCACAAAGAACATTCATCCTTTATCATTGTGAAGACCGCCTGCTCTGACCTTCTATTGCCGTTGAAAACTACTGCGTTTCTTTGCTTCCAAATCTGCCACAAGCTTAGGATTGCAAGTGAGTGTGCCGCCTTTGTGCCGCTTTCGGTCATCTGAAGAAACCAATCCCCTTGTGCAGAGTTTGATCTGCAAATTGGTCCACTCGAGTCAAAACATTTGGCATTTTGGGCACTGTCTAACAACCATTTTGAATGTAGTCATCACTAGAAGATTATCCGTGCTCAGTTTCCTGTTTATTAAACTGTGATCTAAATTAGACAAAAAAAAAAGAAGTGGCTGATTTCTGGAAAGGAACTTGTGGGGTCTATACTCTGTAGTTTGCAACTCATCTCTCTTTATTTGCACTCTATGCTCAGGGGCGTGCAGTGTTTGCCAGTGGCAGTCCATTTGCCCCTGTGGAGTACGATGGGAAGACCTTTGTACCTGGGCAGGTAGAACTTGTTTCTAATATTAAATCCCAGGAGTACTAGGCAATGCAATCTTGCTGGTGTAAACACCCGCGAAACATAAGTTGTTTATTGCTAACTCAACTGTGCTATTTACAACAGTCGAACAATGCCTACATTTTCCCTGGACTCGGCCTCGGTCTTGTTATTTCTGGAGCCGTCCGTGTCCACGAGGACATGCTTCTTGCCGCCTGTAAGTAGCTCCGAACGGAAACCGTTGGGCATAAAAAATCGTTTATAATACCTTGATGAAACTAGGGACCAATCTCATGTTTCATTCATTCAAACAGCGGCTGCACTAGCTGATCAGGCCACAGAGGAGAACTTTGTCACGGGATCGATCTTCCCACCCTTCACCAACATTAGAAAGATCTCTGCGTACATTGCTGCAGCCGTGGCTACAAAAGCTTATGAACTCGGTACGCACGTCCTCTATTTTGCTTTGCACACACCATTCCACGTCTTGGCATTCACCACCAAGTCTCATACCTGTCTGATCTGACATCATCAACTTCCCTCTGCAGGTTTGGCGACCCGTCTGCCTCCCCCCAAAGATCTGGTGGCATATGCAGAGAGCTGCATGTACTCTCCTGTCTACCGTAACTACCAGTAGTGCTGCTGGGATCAATTTTGGAGTAATATAAAATCTATCAACGCAGATGATACTGCAATGTTGTAGTCATTGTCGTTTATAGTCCCTGCTGCTAACCACCCTTGTTGTTGGTGCTGCTGGCAGACACTTTACCTGTCTATGCATATATGATATGTTGTAACGTTATGAAAGCAAACAATCTTGTGTCCCAATGTTTGTTATTCTTTCCCTCGCTTATGATGGTCTGTTAAAGTGACTGAAAGAGTCAGATAGCCGTGCAAATCAAAATGGCCAAATTTAAGCGAAACCAAAGAATTCTGCAAGTTAGCTCAAAGCCTCAAGCCATCAGCACAAACGGGTTGCAAGTGATTTCTCTCTTTTCTTAGAGTTCAAGGGCTTTAATCCCTAATCTTAGCAAAAGCAAGTTCTCAGCAACCACATAAGTACACAACACACTCACATCCAACCAGAAAGGAAAAGGCACTTCTACCTAGAGACTCAACGATAACACGAGCTAAAGATCACCAGGACAGGTAGAAAGCTCTTCTCAGACGCCGGAGTAGGAACGCGCAACCAACAGTGGAGGCGTCCAGCTTCGTAGGTTGCAGCGCTGCTTTTAGCTCGGTTGTAGACCTTTTTATGTAATATTGAAGCTCGTCAGAAGCGTGTCGTTTGTATGATTAAACTCTTCTTCTTAATTACAATGATGCGCGAATCTTTTGCTTATTCGAGAAAAAAAAATATCAAAATAACTGCTAGGCATCTACTTCACACCAGAGGCTTCCTTCAACGTAGAAGATTGATCCAGTTGTTTGTTCTTTCATCTTTCTAGCTTCTCCACCCAAGTGCCATCCTTCACCGCCATCTAGGAGTCCAGCTGGTGCAGCAAGGTCCAAGAACCAACATCATCTTTTATCAGCATCTCGGTCTCTCTTTATCCATCGACCATCGGGCAGCATGGCCGGGGCTCTTGCCAACAATTTCGAAGTTCGAATAACCTCCTCTAGCCTCGGTTGCTCGGTCACACTACGTGGACGTCCACGTGGTCGTGGTCGTGAGTCAACAAATAATAGTCAAAATGTCTACATGATCTCTGGCTCTAACATATCGAAGCTTGACTACATGACTCGTTCAACTATAAATTTTAGTACGATAGCCCTCAAAATATCTAATACATTTTAAATGACCCGAAAATGTTTGTGTTGAGCTGTTTTGACACCATATTCCACTACGCGGGTTGGCCTAATTAGTAAACACATTTGTAAGTGCCTAAGTGGGCGTCTTCTCTTTTTTCCTTCTTACTCTTCGTGTTCACCTCTCCCTCTATTCTCTCCACTCCACTCTTTTTAGTTTTAGACTACCCCACCTAGGAGCAGCCCATGGTTGCAATCTCTTGTCATTTGCTCCACCTCCCGGTCCTCCTCAATGGCATTATCCTTTTCCTCTTGCTTTTTTTCTCCCATGACTACCTCATCAGCAAGTTCCCTTCACTCTTTGCCCACACCCGCCTTAGCAAGCGACCAGGGCCACCCATCTTATAGATAGGTTCATGTGCCAACATGGGTAGGCCACTGTTGGAATTATTTGAACATTATTAGAAATTTTAAGGGCCATAACATGGTTTTCTGTACCGTGTGTTTGATTTGGGGACGTCTTAGGTCCTACGATGAAATGGTCCAATTCTGTATTTGGTTGAGGGATGGATTCATCTATGTTCTCTATTTGGTTAACGTGATAGAGACAAATGGGATGGATACAGTGTTCACATATTTAGTCAAAAGTCAATTACGGATCCACTTGCCAACAGTGGGCCCACTCGTCAGCCTCTCCTTCCCAATCCCACATGCGTCGCCCACCATGCCTACCCACCTGGTGTTGCCCCCACAACACCTCCCGCATCGTGCCCGCACGAGCCAACCCTAATTCCAAGGTATAGGTGTGCCGCGTATCTGAGCACCGACCCTAATTCCAAGGTACGAGTGCGTCACACATCTTAGTAATATATTCCATATCACTCGTCCTCCATCCAAACACTAGTGAAAATAGGATCTGTAAGGCTAGCGTCCGAACGTCCGGCCCCAGGCCCGCGTCCGGACGCAGCTCCCGTGCTCAATGCCCGCATCGCATCTCACGCACCCCGTCCCTCCCATCCAGTGCAAGCGTCCGAAACCACGCCCTGCGCCCATCCCGTGCCTACACGGCGAAACCGCGCCCCGCGCCCATCCCGCGCTTGCATGGCGGAACCACGACCCCGCGTCCACCTGCCCTGACTGTGGAGGTGTCTGCCAGCCTCAAGCACCAGCACGCTAACCACGGCCGGCGCCCTCAACAGCAGCAGTAGGCGGACGGCGGCCTGCAACACCTGATCTACTTTTGAACATCCAGATGAAACATTTGCAACGTAAGTCCGAGACAGATGAAACACTTGAAACATACGTGTGAAGCCATTGCAACACGTGCAACACCAAGATCTCCTCTTGAAACATCCAGATAAAACATTTAAAACAAATATCTAAAACACAAACAATTAAAACATATGCTTACAACATGCATATATTGCCATTGCAACATATGCAACATCTCAGATCAACTTTGCAACATCTAGATAAAACACTTGAAACAAAAGTCTGAAATGCTTGAAACACAACATACGCCCGCGCGACCACGGCCTACCTATGTGGAGAGCTGTGGAACCGGCGGCGACCCCCTCCTGGTGGCGCTAGGGTGGTGGTGGCAGCGCGGTGCACGCCATGGCCGCGATGGACGAGGTGGATGGGGGCGCGGCGAGGGACGCCCCGCATGGTGTGGCGCGAGATTGGGACAGGCACAACCTCGGGATGGGGTGCGGCAGGGGATGGTTGCGGAGGGCGAGGCAAAGTGGGGTGAGCAGAGGCGCGTCGCGGACGCGGCGGGCAGATGGCGCATGGATGCGGCCACTATGGACGGAGGCACGGTGGACATGGGCCCCGTTCGGCTTTGCTGATAAGTCGGCTGAAAAATACTTTTGGTTGATTTATTGTGAGAGAAAAATATTGTTCCAGCTGGAAAAAACAAGCTGAAAAGTACGGATTATAAGCCGAATGAACAGGGCCATGGCCGCTGTGGCAGACGGCGCGGTGAAAGCAACCGCGATGGTCGGGGAGTGGGGAATGTTCTGGACGACACGCAGATAGATGAGTTGGGGAGCGGAATCAGTTGAAGACTAGCTCCATGTTCGCTTGGCTGAAAAGTCGTAGCTAAAAGTATTGTTCGTTGATTTGTTATGAGAGAAAAATACTGTTCGTTAACTGAAAAAGTACGGCTTATAAGACCAGCGAACTGGGTGCAAAGATGCCACAAGGACATTGATGTTGACCCCGATGGATGCTCACGCCCAGAACGAGGCGTCCGAGCGGAGATGCTCGGACGGAGCATTAGCGAAATATAGGATAGGATGGGTCTGTCCCGTCCTCCTTCGTTATCCCAAGAATCTAACAAATGGTACGTGAGAAGGTGAAATGAACGAACACCAATAGTTTGGGGTTGTTGCTGCCAGTGAAGCCTGAGATAAAAGGAGATGCACTTATACATGAAAACAACGTTGCTGAGCAGTGAGCAAAGCCATCTCTTCTTGGAGACGAGCATCTCACGCCATTTCGTTGGATAAAGGAATAATAGAGCGTTGAGCTGCTTCCTACCGAAGATCGTCGGATAAAGCTGGTTCCAGCCGAAGCCTCGCAAAGTTTTGGGATATGCCGCACCACGTGGACAGGACAGATTTCCACCACAACAAAATTGCCCCTGGTGTATCAAAACCAAAAAAAGGATGAGTCGACAAGAGAATTATCCGGATGCCTTATGGCCCTCACGTCGGAGGCTATTTGCCAGAATAACACAAAGGGGGCCTCAACAGCACCAAGGATAAGCTCGAACTTCACAGCACGATAGAGAAGGCCATTCTTGATCTCCAATTCTCCTCTTCATCTTACATGGAGAATGGCATCTACAGAACTGTTATACCTATATTGATACTGTGATACACTGGTAATCTGGTATCGATATTAGTACACACTACACGCACAGTGCCGTCCTCCACCTAGTGTTATTGCAGGTTACATATCTTCCGTACAGATGGCTGACAGAAGCAGTGAATAAAAGGGTTTAGTAGGGCATCCTTTAGGCCTTGCTGTCGTTGTAGGAGTTCAGATCTGCATGAGTGTCCTTGTAACGCTGGGACAGGTATGCAGAACAGGTGATCGGAGGGAACCTGAACAGTGCAATTGTAAAGATTTGGAATTTAATAGAACGAACATATATATCCTGATGAGCAGCAGATGGCTAGCTGCCTGGCTTTAGCCATCTGAGGGGGCAAGAGCTTACTTTGGAGGATTTGATTCGGATTTGCAGGTCGGCAGGCACTCAACTACGCAGTCATGGCTTGGTTCCACAAAGTAGGCTATCTGTTCCAGACAAGGCAAACACCGCCATTGAGATCAACTAATTCAGCACATATTACCAGATATATGCTTCGTTTCAATGAAACTGGAACAGGGAAACAACTTAAGGACTGGTGTTTTTCTTTCCTTTTGTCCTTTTCGTCTTGCAATACTTCAGGTTTGGGCTGATCTGGCTGGTTGAAAGTACTGCTGGCTGGTTTTTTTGAGAGAAAAAGACTGCTGGCTGGCTGGCTGATTTGGCTGATAATCTCAAGCGAACAGTCTACCAACTAAAAATGAATTTATCTACATAACAGAAGGAACATACCGAGTAGCGTTCTCGCCCATCTAGGACCACTCGATGTAAGGTTGACCTACATGTATGAGAAATAATTGTGTTATATCATAAGAAACAGTAAATACTAGTAAACAAAGCATAACAGTGTTTAACTGCTATTTTCTTCCTCAAACTGACAGCTTGGTGTATCCAGATCCCAGTAAGAAGTACGAACTTGTTATTGTAACAGAGGCAGGTTGGCCTACCTGAAAATGCTGTTACTCCACCTTTCCAGCATATCACCAAGATTGACGATAAATCCACTGAAAATTCATGGTCAAACATGTTACGAGATACCAAACACGACTCAGTGGAAACATAAAGTAATTTAGATTAATAGGCTTTACCCTTTCACTGGAGCTACATATTCCCATACTTGAGGCTGAGCATTCTTGTCCTTACATATCTGCAGAATTCAAAAGAGTGAAACCCAGTTGAAACAAATCCAAATAAAAACATTAGAGAAGGTATTGTTTCAAGGCGTATTACTTGGAGACCAACTACATCATCGGTTGCTAGGAGAGTTATCAGGCCATAATCTGAATGGGCACCCGCTCCATAAACACCCTTTGCAGGATTTGACACTTTACCTGATGATAGAAAGAACATCAAACAACAGAATTCGTTATGTTCCTTGCGGGTAAATACCATTTGACATGAAGGCATGACCTGTTTTTTGTCCACCTTCATAGTGCAAGAGCCTTAAAGTGGCTATAGGCTCGCCAAGCATTTCAGGTCTATCAAAGAAGTCCACATCTAGATCTAGAGCAAGAGCAATGATCCTTGCAACTGATTTTGCTACTCTCCTGAAATTTATAGAATGCAGATCAGATCAGAACAGTAAAAGCTCTGAAACAAGAAACTATAAACTTCATTACCATACTTCAATTGTCTATATATGAGTACATTACCATACTTCATTACCATACATTAATAATCCACTTTATCAAATTTAGAACTAAATTAGAACCAATTAGTATAATAGATGTGTGCTTCTATAAGTATCATTTTTGAAGTCAAAGAACAATGAACTCACAACGCCTCCCTGTGATATTGCTCCATCACTTCTCTCCATTTTGGTAATACTTCTGAAAGGAACAATGTGGAAAGTATATATAAAAACAGGTAAACACAATGTAATGTCATGATAATCACCAAATTAATCGTTAAAGGGATCAGAATTTAAATGATGATACTAGAGCAAAGAAGGATATACTCTTATGTGGCAAAAACCCAACAAAGAGGAGTTGGACTATAAACAATCTACAGTTATTCCCCGCGTTTCAAACATATGTTAAACCAGTGTATGAGGTTGTAAGTACAAAAACTGAATGATTCATGAACAGGAAAGAGCTTCACCTTTAGAAGGCCACTGATTTGGACCATAAAATGGCCTGTTTGCTTCTGGATCATCTGCAGGTACCTCAACTCCAATATAATATCCTTCCTTGTAGTCACCTGAAACACATTATAGAGATGATAAGAATGTGTAAAATATGTAACTGAAAATAGTACAATACATAAACGAAGGACATCCAGATTAAAGTGTGATTTTCTCCGTGCAGTTGCCTTTAGATTTATTTTTTTTAAAAATGTCCTATGCTTTTGAAATTAATACTGTTATAACCTGACAAGATTTTCATCACTAGCAGTTCTCTACCATAGATGGCCCATGAAAATTAGATTTATGTGCCACAACAAGCATGGAGTGGAGTTTGAATAACTTACCATTCACTTGATTTTCAGGATCAAGAATTTCATCTAGCATGGGTGTGTACCCTCGATTCTTCTCGTCTCGGAGAAGCTTCATTTTCTCACTGTGAGGAAGGTCAAAGAACTTCTTGCTCTGGGCAAAAACTTCATCCATGAACTCTTGGCTTATCCCATGATCCACAACATAGAAGAAGCCTGAATCCAGGCATGCCTACCAAATAAAAACATTGTTTTCAGGAAGAAGAATATGATACAGAGCTGTTATAAGAACAGAGCCAGCAAAGATATTTAATACTCAAGCACTCAGCTGTCAAACATGCAAACTTCAATATCATGCACACTAGATTTTTAGTGGTCCAAGACTAAATTATAGCCAGTCATGAGGCAACAAACTAAAAATGGCTCAGGAAATATCAGAAAATCTAGAACAGAAACAAGTGGATGCATGCCATATATCACTGTCTTACTATTTACCAATTCCAAAATAACATGCAAAAAATGGTAACATACATTTATATGGATTGTGGCATTGTGCCATAGAGGCAAGATGTACTTTTGGCATAATCAAGGAAATAAAAAATGTACACTAGGACAAATAAACTATCCAAGTACCAACAAGAATCCACCTAATAAAGAGAACCCTGCTGACTACGCTAACAACAGACAGCTAAAGAAATTGCAAGATGGCAGGTCCGCAGGTCACTCCCGTCAGCGTAATCTAATCGAACCTTTAGTTTCGAAAGGGATGAGATTTGAAGGCACAATTAAACAATATACTAGGTGAAAGCAATTGCTGATTAATGGTAGTGCAATTAGTGTAATATATATATTGAAAAGGAGTGAAATTCATGTTCTTGTGGAACTTGTGCACTGATAGCCACCCAATTCTATTAACCTGCCTTACGAATAGAAAAAAAAAGAGGACATAATAACACACTCTCTCATGAAAAGAAACAACAATACTGCAGTGATGGAACTCTGGAATTGCCTTCTATACAGTTATAAATAACCAAAAAGACAAGATGATTCTCGAGAAAGGAAGTTCCAAGCGTCTGCTATTCAGACACCTGAGCGACTAAAATTACGATAATCAAGAAAGAACCGCAGACCTGCTTGAGGAGCGCGACCGATTTCTGGACGTCCGGGTCCGCCAGGCTGATGCAATTCAGGTTAGTCCTCGAGATCGGGCTCTCCATGCTCGGCGCTTCTCTCTCCTCTCGACTCTCTCCCTCTGAGGCTCTGATCTCTCTTGGATTTGCCGCTCTTCTTTTGCTCCCGCCGCCGCCGCCTACCCTCTTCCCTGGTAGCCGCCTTCCCCTTTTCTTGATACGCTCAGGTTCAGGCAGAGCAGGAGTTGCCGGATTGGAATGGTCTGACGAGACGATTTATAAAGGTGGACGAGCCTGGAGAGGAAGGTAGGTGGAAAAAGTTGCAAAAGCCAAGGCCAAGACCGAAATCCAAAAGCGCATTGGCAATGGGACCCAATCTCTTGGTGGCCACCGACTGCCTGGGTCTATGGGCCAAGAAAAAAAAGAAATCAATTTGTTGTATTTATTTATGTTTGTGTTTTCTGATTTTCTATCTTTGCTTAACACTTTATTTATGATGTTGTCAAAAAAACATTTTATTTATGTTTTAGCTTTACTGTGTCGTGAGAATATGATCAACGGAATTGATATACACTGGGGCATCTAGACGGGGACGGACGCACCCGACTCGTGCCGAACCAACTCACCCGTCTCTCTCGCTCCTTCTTATCCTTTCCTCGAGCCGCACTCCCGCACTGCTCGCGCTCCCATTCTACTGT

>BAC-33

TTTTATTATTTTCTATGATCAATTCAGCTTAAACTTGTAAAATGAATAATTATAAATAGTAGGATTTATATTTTGTGCGGAATTAGATTCTTACATTTTTTTTAACATTCTTATCTAAGCTGCAAGCCACATGTTGGAAAAAAAATTCGTCGACCCGATACAACAGAGGGCAATTTGTGTGGAAAAAATTCATCGACCTGATACAACAGAGGGCAATTTGTGACTCCGTGCAACTTACGGGCATATATCTAGTCTACATAAAATATTTTAGCATATTAAATCCTTCATAAGTAAGTGAATTTTATTTGAACTTGTAAACTTGGAAATCTTTTTTCTGAAATCGTCGTCGAAGTTCAAATTTTGTGAACCATAACTTACAAACATACAGTAAAAAACCTGGATGTTAAGTGCTCTACATTCGAACCTGAAACTCTTTCGACGATACAGTCATCCTAAACCTTAAAACTCTTGCAAGCATTGAAGCATTCAATCAAAACTGGAAATCTATTGCCAGAACCTGTGGTGAACCTTTAGCGAAAAATGTCAGGCAACATTCACTGAACATAATAGAAAACCATAAAAATGCAAACAAAACTGAGCACGCAGATCTTTAAGTTCAAACATCACTTATTCATGATAAAGCCGCATACTGCTGCACCAACAACTGTTCCTGGTAAAACACTTGGGACACAACCCGTCCACGAAACTAAACTGAATTACAAGACAAACCACAGATACTTAAAACCCTCCATGGCAGAGACCAGAGGCACAAAGACAACCCAGGTACACAGCAGCAGCTTCGACGACCAATGCCCTACTGACCACCGCGGAGACGGAGCACCAGGTGGAGGGTGCTCTCCTTCTGGATGTTGTAGTCAGCGAGAGTGCGCCCATCCTCCAGCTGCTTGCCGGCGAAGATCAGACGCTGCTGGTCTGGGGGAATGCCCTCCTTGTCCTGGATCTTGGCCTTCACATTGTCAATGGTGTCCGAGGACTCCACCTCAAGGGTGATGGTCTTGCCAGTAAGGGTCTTCACGAAAATCTGCATGCCTCCCCTGAGGCGGAGGACAAGGTGAAGGGTGGATTCCTTCTGAATGTTGTAGTCAGCGAGGGTCCTGCCATCCTCCAGCTGCTTGCCGGCAAAGATCAGACGCTGCTGGTCCGGGGGAATGCCCTCCTTATCCTGGATCTTGGCCTTGACGTTGTCTATGGTGTCTGAGGACTCCACCTCAAGGGTAATGGTCTTGCCGGTCAAAGTCTTCACAAAAATCTGCATGCCTCCCCTCAGGCGGAGCACCAAGTGGAGGGTGCTCTCCTTCTGGATGTTGTAGTCGGCGAGCGTGCGGCCATCCTCAAGCTGCTTGCCAGCGAAGATAAGCCTCTGCTGGTCCGGGGGGATGCCTTCCTTGTCCTGGATCTTGGCCTTGACATTGTCGATGGTGTCCGAGGACTCCACCTCAAGGGTGATCGTCTTGCCGGTGAGCGTCTTGACGAAGATCTGCATGCCTCCCCTGAGGCGGAGCACCAGGTGGAGGGTGCTCTCCTTCTGGATATTGTAGTCAGCAAGCGTGCGCCCGTCCTCGAGCTGCTTGCCGGCGAAGATGAGCCGCTGCTGGTCTGGGGGGATGCCCTCCTTGTCCTGGATCTTGGCTTTGACGTTGTCGATTGTGTCAGAAGACTCCACCTCAAGGGTGATGGTCTTGCCGGTGAGAGTCTTGACAAAGATCTGCATCTGTAGAACAAAAAACAAGCAAGATAAGTATACAGCAGTAGACCGTGGAGGAGGAACAGTCAACAACAAACAGATATGCAGGTTAATCATATAGGTACTACAGAACCAGCAAATAGAAGCAGCATGCCACCATCCAAGTAAATCAAAATAAGTAACAACAGCATACATGAGCATGAAGTGGATAGATAACCATCAGTAGCAGTATATATTCATCTATCTAGGCAGGCCCGTCTTAAGGGGGAGGGCAGGCAGTGCGACGCCCAAGGGCCCATGACGATAGGGGCCCATGAGTATACGTATATGTAGTACATGTCTTTGGCCTATATGGTCACGTCCAGTTCAGTCTGCTGCGCGGCACACCCTTCGTATTCGCAACTCCGCGTCATCGCGTGCGTGCTGTTGCACCGCGGCGGCTGTTCCGCCGCTCCCGAGTCCTGAAATCCCAATACCTGTAAGCTTCCCATGATCCCAGCAGGCGGCCACAACCCCAGCGGCGCTACAGTCTCTCCGTCCCTGTCCCTTCACTGTGGTAAGTGATTATGGTTCTCGTTCATCAGTATTATTCAGTGTTTGTTTGATCTTATAACTTTAGTTTTTTTGTAACCTTGCAATCCAATCAGGCATCAAGTGATAATGAAGCGAATAGTTGATCCATTAATTTAATTATTTATTAATTCATGAAGGCACATAGGGACATGCCATAGGCTGCAGGTAATTTGTTTTAAATTGAAGGTATTTCATATTACTACGATTTCACTGTTATTTTAGCTTCTTTGATGTACTGTCGTTTTTTAAAGAACTAAATACAATTTACAAGTTAACTCTTATTAATATATTACTGCACAGTTCAACCGTCATATTAAAACATTATTGCGGGGCCCTTAGTGTTTTGCTTGCCCAAGGGCCCTACAAATTGTTGAGACGGCCCTGTATCTAGGCATGAATACCAAACAAATCACCATGATAATAACGCTCATGAAAAAGCTACAGCCTGAACGAGTACTGTTGAAGCACATCTTCATCCATCAACCGTAACTACAGACATACTTGGTGACATGCAGGTACAAATCTAATAATAAGCAGCAGCTATTTGAGAGGAGCTATTCTACCGTAATGTAAAGCTACAACTCAACCTGACTCAGGACAAAGTCAATCAGACAAATCATGCGCACAAACCTAACTAGTACCATAGAACCAGGTGAAATCTAATAATAAGACCAGCAGCTATTTGAGAGGAGCTATTCTACCCTAATCTAAAGCTACAACTCAGCCTGACTTAGGACAAAGTCATCAAACAGACAAATCATGAGCACAAACCTATCTAGGAGACTAGGACCATAGAACCAGATCTATCCCTACGCAGCAGCCAAAGAAATCATGAGAACGATCCTAGGAACCGCGTAGAAGCCAGCCATCTACGAACAGAAACTCGCAATCTAGATGAGAAGCATCTATTCACAGAAACTCGCCAATCTAGATGAGAAGGGGATTACTACGCACAGAAAAGATGAGAGGGGGAGGAGAATCGCCGTACCTTGAAGCGGGGGATGGATTGACGAGGCTCACGAGAAGAAGATCGATTGGGATTTGGGATGGGGGATGGGTGCTCCGAACAACACAAGGGAGATGAGATTGGGGAAAGAGGAGGAGGGGTGGCTATTTATGTTGGTGGAGAAGGAATCACCGTACCGTGGGCCCGTGGCGGTTCCGTTCCGTGCGAGAGAAGTCCGCCTAGGAAATTTGTAGGAGCCCGCCAGCGCAAGAGTCCAACGCGAACGCGGAATGGAAGGCGAGCCGGCCGGCCGTAGAGACGGAGGAGCGGAGCTCCCGAGAAGGAACCAGTGACGGAGTGGACACGGAGACGTCGGACCGTCTGTTTCGGTCTGTTGGTTCGCTGGTTGGTGGCCGTTAGACTCGACGCTGTTAACGCGTTCTCCCGTGATTCCGTCCATGTTTACCGACACTGAGACGTGTGGCCCAGGAGTCAGAAAGCCTTTTTTTCTCTTTTTTCTTTTTTGAAACGAGAAAACCTCTTTTTCATCCGACCGTGATCACTGATCAGGATGAATATGATTGCGATTAAACAAGTGTATGATTTAATCGGCATTTATGCCGTATTCCACGGAGAGAGCTCCCCGCTCTCAGTCACTTTGATAATAATACTCAAACACTTTGGGCACAAGAACGTAAAATAGGGGGATTGCTGCACCTTGTTACCCTGCCCATGCGGGGGCTCGCACCTTCCGGACGTCGCCCTCGTCGTTCTACCACACGCGGAACCGCTTGCGCCCCTCCGTTTCACACGTGCATGCACGCGGACCGTCTGCGCCTGCTCGACGGAGCTCCAGCAGCTACAGTGGGCGGCTATGGCAGATAGGTCCCATTGTAACATGTGCAACACTAGATTTATTTTTACAATATACATCCAGAACTGCTGAAACACTTGCAAAAACACTTTAAAGTCACTGCAAACATATGCAACAACAAAATAAAAACATTTAACATCATACGTCTGAAAACAGATGAAACATATTTGGAACAGACGCTTGCAACATACACATATGCAACATCCGGATCTATTTTTGCAACATCCATATGAAACACTTGCAATATACTTTTAAAACATCTGAAACACTTGAAACATACGCTTGCAACATGTGCTTTCAACGCAACATCTACTTGCTGCTTGAATGAATGGAGGCTCGTTGACGCGGTGCTCGATGCCACGGAGTTGCGCGTGGGGATGGTAGCGGGCGGCGGGTGCAGTGCCCGTGGCAAGCGGCTGCGCGACCCGCAACGATAGGGTGAGCGGGTGCGTTCCCCCGCGGCGAGGTGCCCCTTGTGGAGGCGGCTGCGCTGCCCACGGTGAGTGGTTGCGCTGCACGCGGCGAGCGGGCCCGCTAGAGAAGGCAAGCAACGAAAGGGCAGCGCGGTGGAATCGGACACGACGATCGAATGGTGTGGTGGAGAACGCCGCAACAGGCGAGGAGGAAGAGGAATGTGTCCAATTTTTTTTACATGGAGAAGGTGTGAAGAGGGCATGCCTATTGAGCCGGTCCACAGACGCAGGAAGAGGCGTCCGGACAAATAGACGCTCTACACGGCTACACAGAGCATTACCGGTAAGACCAGCGGAAGAAAACACCGAAACTAAAGGTGGGAAACCATAGTATCCCAAAACACAGGAATGAAAATTTTGGGCTTGTCTAACTCTTCGTAGCCGCTGCTTGATTGAAAAATCAGTTTGTAAGTACACTCTACTCTCTACTAGCTGATGCTTGCTTGCCTCTTTCTAGCCATTTCTCATTTCTGCATACCTTTGGAGAGTAAACACACCATCCATCCATGTCCGCTGCCTTTATCATCTCTTCTATTTGTAGATCAGGCAATCCCTCGAGAGCTGTGACACACTTCTTGATGCTGTAAGGATCTTCCATTTTATTCTCTTCTATAGTCGCAAATCGCTCAATATCTTTCTTTTTGAGCATTAGGTACCTCTCCTGGAAATCATTAGTTGATTTAGTGATTTTAGTCTAGCTGTTTTTCTGTTTTTTTGCTCGCCTAGCATCAACCGACTCTGGAGTTGAAATGCTAGATGGCCAGGAAGTGTGACGTTGAGTTTCTAGGAACTGCTTTGGAACAAATGTCTCAAAATTTGAGGAATTTAGTGGATGTGCTTCTTCTGGTGATGCATTAAAAGTAGAGTCTTTTACTTGATGAAGGACTGAACTCATCATCACCAAACCAATTAGAGTATCATCCCTAGTCCCCTCGACATCAAAATGCAAGGCTGATGCACCATCAGCATCAGGTACATTGCAGTGGCAGTGAATGCAATGCAGGAATTTCTGAACATACTTTTGCCTTGCTCGCATGGTCCATTCCATGGCAACTTCTTCCTTCAGCATAACGACCTGCATAAATTATGTACGCAATATATATTTTTAAATTATAAGTACAACAATGTGTATAGATAAATATAGAGAGCAAGGTCCATGAACTGAAACTCACCATCATATATATAAAGAGTATAAGTCATCGAATTATGGAATGATTTATCTCGCCATCGAAGAAGAGCGTCTTTGTTTCTACGTTTCCCTAGTCCCTTCCAAACACTCTCTGGTGCTGTCACCATCATCCTACTGGAGTCCTAGCCAAAACCACTTTCTTCTCTCAAATCCTTTATAATCCGATACCGTTACTTCCTTTTGTTTTACTTGAGCAACGGTGGAGGCTAAAGCAAACCTTGAATTGAATTGACCAATCATACTAGTCCATGCCTCCTTGCTCCTTGCATTGTGTGTCGGAAATCTATGTAAATCATGTTGTTCTAAAGGTTAGGAAAACACCCTGATTATAGATTTGTAGTACTTTATGGAGAGGCAAAATATGTATTGGAAAGTGGAAACAGGGCATGATTGGGTGCCCTGCTGGTGGCATCCGAAAGTCCATCTGGCACAGTTTCGTGCACTTTGGCCTTGTTTGATTCCCTGCCTCGCACCATTTGCCTGCTTTCTTCTATTCCTTTCCTCCCTTCATCCCACACGCCTTGGTCATCTCGATTTGGTCTTCCCTTATAAATTGGGATGGCTTGATTCACGCATGTGCAGGAGCCCATGTGCCAAACACCCAAAACTCTGATGTATGCATGCAACTAAACATGATCTTGTCTCGTACTGGACCAACAACAAAGACAACAAAAAATAGCAAAGTGCATAGTCTCATCGGGCCTGTTTGATATGGAGCCCAGGGGTGTCCCAGCGCCCTCTAGCTGCAACTCTCGCTGTTTGATTCACTTGGCCTAGTTTTCGCCCGGCCCGCGGCGTGCAAATTCCCCTCTCTCTCTTGGCTCCCAAGAAACGAGATGAAGGTCCGTTTCCTGGTGGCCTGGCTCGCTCAAGCGCGTAAGCGGCTCGCTTGAGTGCTGGAGCGTGTGCTCATGTCACCTCCGGTCACCTCCTGCAGCCGCGCACACCTCCGCAAGCCATTCGCGCTCGCTGCCGCCGCTGCAACTCCAATCGCATTGCCGCGTGCCAGCGGAGCGGTGGAAGCCGATGGTGTCCCTGGCATGGAGCCTCTTCTCCTTCACGGACTGGCTCCACACTTTCCCGGCGGCGTCCTCGAAGTTAAGGAGCACGCCCTTGCACTCGCGCGCCATCTTGTCGAAGAGGTGCTCACGCACCGCGGCCGCCATCGTGGCCGCGTCCGTGGCTGCTCGCCCATTCCTAGTGAGCGTGGGTGTCGTGGCTGCCGCTGCAGCAACAGAGGACGACGGCGAGGACCCTACTGCCTCTCTGCGTCCACCAATGGCACCGAGCCTCTGTAACCCCTTGGTGTTACCGCGTAAAACAACTCACTAAATCATTCATAAGCATCATGTTGATGTGTTATTGCATGTGATCAATTATGTAGATAAAATCTTGTAACCCAAAATAATCAATAAAATGTTAAAACAAAAAGTAGTCCCATGTTGCATTTTATATCCAGTAAGGTTGAAAACCAAATTTTCATGAACAAAAATGTTATAGAACGTATATATGGCATATTAATAAAGTTGGAAGTACGCACTTCGTAGATGGCAATGCAACTTTTGTTTTAGCAAAATAATAATGTGAGCTAATATTTCTAGTAGGTTGGAAATTGAGCTTGGAACCTAACTCAGACCTAGACTTAGAATAATTTTCAACCTTGAAAATAGCGTGACAATGCAATATTGGGGAATAAATTTCGTAAAAATTTGTTAATAATTAGTATCGCGTTTTAGCTCGGAGGGATAGTTTGATGTACCCTTGTAACATCCGGTCTCGGCATACACAGCCCAGCCCTACTCTGCTGAGGAGTGGTCCCACCTGCGTAACAACCCGCTTGAGCTTTCGTCCTCGCTTCGCTCAAAACAGTTAACCGAAGGGTACTACGCGTCCCTGACCCTGGTATTTAAGTCGGTCAAACTCCCTCAGGATTCACAGTATGCTACTGAACACCTGTTGCACAGTGTTTCGTGATGCTACAACAATCGGTGAACAGTACCCACGCTGCAATAAACCGAATTTGGCCTGGTCCAATTCGGCGCTATAGCGACAGCCTGCTACAGTGTCCAATCTATAGTACCCTGCTGTTACAGTATCCGGAGTTCGAATGTACAGTAACCTGCAGCTACAGTATCTGGCCCGCTGCCCAATCTACAGTACCATCATCCCATTCCGCGGATTGGGCCCACTTGACCCATTTTGGGGCTGTTACAACCCTCTTAGGCTTGGTCAAGGCAGTTGAACCCAAATCGAGCTAGCTTAGTTTTTACACGTGCTTTAAAATCCGTGCACGCACACTGTCTCGGGCTGTTGGCTGGGAGTGCACGGTCACCACACAAGAGGGCTCGGCGTCGCGTACCTGGCCACACGCCCGCACGTGCGTGTTGCCAGAGTCGGTCAGTCGGGCCTGTCCCTGGCCTGGCTGTGGCTTGGCGTGGCCGGCCACAATGGGCCATGGGTCACCTGCTGGCCATGCCGTGGGCTCGGTGCCCTGGCTACCACCGCAGCCGCTGCCCCCCTGCCTAGCTCTGCACCGCCGCTGCTGCCATTACTTTGACCTGCGCGCGTGTAGGCTTGGCTTGTTGCACGGAGTCGTCGCCTTGACGTCGTATCCTCCTACGACCTGTGCCCCGTCTTGATGAGCGTCGCGCCAAGCACAGTCCTGCCCGACTTCCTTCCCTTGTCCTTCTCCCTTTGTCGTTTCTGTCCGGCGCCACTCGTCCGCATTTCACAAGTGAGAGATCATCGTGAGCCGATTCTTCACGACCGCGTCATCGCCAGTAGGTGCGCTAGCTCCCCAACCCCATCTCGCCTTGATCCACATATGGCCAAGCGCCGATTTTGGCGTTTCCTCACCGCCGGTGCCGTTAAGGCCGAGATTGGCCATGTGGACAGTTGCCCCCTCTCCATAGTCGCTCGCCTGCCTTCGACTGCACCAATAGCTTCGCAGCAACCTCCACTTTGCCATGCGTACCCCAGCTGAGCCGCCACCGTTGTCCCAGCACCGCTGACGCGGCCGTGCCGCCATGGTTTGTCCCCGAGATCGCCGCGCGCATGTGGCCAGCTTGCCGCCAGCCTTTCCCACTCCAACCCCTGTAACCGCCGAGTTCATGGTAAGGTATTAGTGCTCTTGCACAAATCGGTTGGTCTCTAGCCGGCACCAGCTCATTGGAACGGCTACGCCACCGTAATGGGCGGCTGCGCACCGTGGCCACATGCTTATGCGTGTCCCGCTGCCCCCTCACCGCCGCTTAGCGTAGGTGTTTAGGTCATAGGCTAGGTTCGGCTCGTTGGTCAGGTCCGTGCGCGCCTCGGGTGGCCGGCGTAGCCGCCCAGTGCCACCGCTCGCAGCTTTGGCGAGCGCGGGGGCGTCGAGGACTGGCTGGGTGTGAAGACTAAATAGGGCGAGGGTCTTTGTGCATAAGCACTGACTACGGGAATAGTATCATGGACCGCGGGGTAATTCGTTTAGAATCTGGGGTCATTTCTGTAAAAGCGTCAAGCGCGCGGGCTCTCTCCCGCCGTGGGCCGGCCTACGTTGTGTGGGCCACGTCTGCGGGACGCGTGCGCTACTCGCGAGTGGGCCGAGCGAATGGGCTGCGCAAGGAATTGGTTTTTCTTTTTCCAGGGAATTAGCAAATGCTTTTCGAAATTAGTTTTGAGCCGATCTTTGGTAAATTATATCAAATCACGTAGGTGCCCAAAAATTGTGAAATAAATTTTGTTGAGTTCATAAAAATGTTGTCTATCTGGTAGTATGATTGGTTTATATATATCTATGTTGATGCTGAGATATATTACAACGATTGAAAGTACTTAATATTGTTAAGTTAAAACTTGTAGGAATTATTGTGGCAAATTTGTGATTGCTTTAACTCTAAAATTTTTATGGTAGCTTCATGGCGTTATTTTGTGATTGCTGTAAATTTTGCTGCCCCAGAGTAGATTGTTGCTAATGTAGCTAAATATCCACTAAAACTTAACATGAATAAACATATAATATTGATATATAAACTTAAGCACTTGCATGGTGAACTTGAGCTTTGCTCGAAGAAGACGATGATAGTTTTCTGTCTTAGTCATTAGAGCTAGCTAAGTAGCTTAACATGTGTGTCCTATTTTAAGAGTTGATGTTGCTTAAATACTAAATGATTGCATCATTATCGCATGCATATAGAGAACGAGTTGGTGGAGTACGTGATCCCCGATGAGCAGGAGTACAACGAGGTGATCGAAGAGTATGAGGAGGAGGTACTCGTACAGGAGGAGTTTCCAGAGCTACCCGTGACTGGCACCGGTAACGTTGTGCCTGCCCAAGGCAAGCCCGATGCATGACCTTTATTTTGCTAATCACTATATATTATATTTATTATGCATTTACATTGCAGGAATTATATGAAAACCACATGCATATATATATATATATATATATATATATATATATATATATATATATATATATATAT

>BAC-74

TATTAATTGAGTCCACCAGTTGATATCATTATCAGTAACTGCATTGAATAATATTAATCTTTAGTAATGTATCTTCAATTTCTGTGTTTCTTGGGTTCTTGCTCTTATCAATTTCCATCGATTCCTTATAAATGTGTGGTGGGTTTAGCAGATCCCATATCTTTCGTTGTAATGTGGACAATGATATGCTTTGGTTATACTTTTTCCCATAGGTATGCAAATCTTATGTACTGTGATCTTTTCGACTTTCTAGATGCTATTGTGGGAGTCAATTGGTGTGTGTTATTTCAGGAGTGGATCCCTTAGTGCCTGGTTACAGAACCTACTGGTGAGTAGAATTTGACACTTCTCTTTTCAGCTTCTCACATCAACAACCCACCTTGCATTTCTGCTTATTGCAGACTTCAGGTCTCAGCATATCTATTGTGGACACCACAATCATCATAATGTCAACAATCCTGTCTATTGCTTTTCATGAATTTGGACATGCCATTGCAGCTGCAAGGTCTTTATGTCTTACCTTCTCATGTCTATTTTTTTTTCTCTTAGATGTTGTGTGCTTTTGTTTTCACTCGATACTCAAAACATCTGTAAGCGAGGATAATAAGCGGCAAATAGTGGATTAATCATGAGGGAATAGATTACTGGATTATATAGGCATTGCTGCTCTGGGTTTTAGTTTATAGAAATAGCACCAATCACAAAGATCCCTGCGACCTTGCCTAACACAAAATCGATCTATATATGTGGCATGGGCCTATGGACAGCGCATGCACAAGACTAAGGCCGACGGCCTAAACCAACTATCTGTCTACAGCTTGCTCATGAATTATTTTTGCGTCCTGTTCTGACACATTATTTGCTACATTTGCAACAGTGAGGGACTACAGATTGAGTATGTTACCATATTTATCGCTGTACTTTTTCCTGGGGCATTTGTTGCTCTGAACTATGACCTACTACAAAATCTGCCTCTCTTTTCTATGCTCCGGATTTATTGTGCAGGAATTTGGCATAATGTTATGGTAAGCCATTCTGTTGTGCATTGATTATTTCCATAAGATAGGAGTAAGTCTATTTTATTGGTTTATGTCCTCTTTTTATTTTCGTAATTTCGTAATCCATTGTCACCTACTTTCAATATTTTGGTACATCTTGCTACCTCATACAGAAGTACAGTGCTTAAATCAAAAGAAAAACGGGTAATTATTTGTTTACTTGCAATAATAAAGGCAACAAACAAGAAATAAGAACAATATGCTTTATGCAGTTTGGCATAAGATGCAATTGGTCTCACTGAATATTCATTTTAAACAGTGTGAGCAGAGATATATTCAGAAAATGGAAACTAACCTCCTAAACCAAACAAGCATGTCCTTATCTTTGCTTAATGTAATTTAGAATTTTTTCCCTACAAGTTCACTTTAAAAAGAGTTATAGTTGATATTGAGGGGGTACACCACTGAGTATCTTGCCTACACATACACTTTGAGGGTATACTCCAGTGTGTATCATGCCTACACGTACATTTGAAGGGATGCATTTTGTCTGGTTAGGACATCAGAAACTGAAATCAATATTATAGGACATGGTTTTTCCTTCTTGTTTTTTATTCATTTATTTGACTGTAGGCCTGTAGCCTAAATCAGGGCTCAAGGTATATAACAACCAAATAGAAACAATTGCTGGTTTAGAAAATGCTGTCAAACCCTGGTTAGAGAAGTGCAAATATAGTTCTAATGCAGTCTTAGACGTAGTTCAAGTGTAATGTTATATACTTGTATACTGAGCTTGTTACTCACTAATTGTGATGTTTTATAAAAGTTTTCTTTTGGTTATGATTTCATGTTTAATAGCTCTGTACAGCATGTGTAATGATAACATTATTACTTCCGGTGATGTTATATCCTCTCTATGTGAGTGGTGATGGCCTCATGGTAAGTCTACTTCTATGTGTTTACATTATTTTAATCTTGGCAGTCTTTACCATCTAAAAGTCTGTTCTGACAGGTCATGGATATCCCTGAAATATCTCCTATGTCAGAATACTTGTCTTCTCATGATGTTATCCTTTCTGTGGATGGTCTGAAAATAACAAGAACTGATGAATGGATCAAGATTCTGAACCAAGGTTCTACAGCAAAATCTAGTGATCCTGAGTTCCTTGAAGGCTCTCATAGATATGTTGCCACTAGTTCTGCCAAGGGTTATTGCGTGCCCGATTCATGGATAGATGCAAGCAAAAATCTCTGGCAGATAAGAGACAAGCTGCCCTGCCCAGATGAACTGATAGCTTTCGAAAAAGTTATCTGCAACAGCTCAACCATTTTGACCGATAAGACTAGCATAGGTAGTGACCAGAAGGAAGTTGAGGGAAAATATTGTTTGATCGCGAAAGATGTAGTAAAGCTTAGAAGATGTGGAAACGGATGGCACAGGACTGAAGGTGATGAAAGTAGTTGTGCATGTTTTGAGGTAAGAGCCCCTCATTTGATTTGATTAGTACCACATTTGAATGATGAATTAAGAGTTTTTAGGAACAATCTTTAGGAAGTCCTTCCTTCTTTTAATAGAAGTAATCTTTTTTATGATTCATATATTATGCTTAATATGTGTCTATTCGGTCAATGGTGATAGGATGAGCATTGCTTGGTACCTGTTCTCACCCCAGGCATTTCATGGATCGAGGTCTCCTACACCAGGCCATACTCTTTGGGATGTTTACAAAAAGCAGGAAATATGTTACCATCACATACGACAAATAGTAACCTTGGACAGAGTCCTTGTGAAGGATCTTTTGTTTATGTGGGTGATCTGTCATCTTCAGCACGTTCTGTTAGGTTATCCCAGTACCGACCTCGATGGGCACTTCTACTTTTCATTGCAGACATCCCATATACCTTCGAAAATGGTTTAAGTAGCTTGCTTCATGCATCTGCGGCATTGGCTGTAATCAATTGCCTACCAGTGAGTGTTTACTGAAGTAGTGACAGTTCCTATCACCCAATTGTGACATTTTTACATGTCTTTGATTATAAGAGGATAGCCTTAGAAGAATTTGGTGTCAATCTTTAGAGCAAAGAAACTTTCCAATTTAACAAGTAATTTTAAGGACAAATATATAAGATATGATAGTAGAATTTTTTTATGCTAAGATGGCTATATTCTTCTTCCTTTCTGTTTTTGGGCCTAGGTTTGTTTCTATTGGGTTATGCTAACTGGTAGGTCTGGATTTCCGTCTTTCTTTGATATGATTTCTGAGTGTTGGTTCAGTCATCTGCTTTTCTATGTTCGTAACATTCTGATCTCCGGCTATCTTTTTACGTTTGTAGCATTGCGCTTCTATGTTCAGAGTGCTTTTCTAGCAGCTAACTCTATCACAATGGATAGGTGTATTTTCTGGATGGTGAAGCGATTTTGGAGACTACCTTAAGCTATGTAGCTTGGTTTACCCCAAGATTTCAACGCAGAATTCTGAAAGTATGCCGCTTTGTGTGGACGGTTTTATCAGTTATTACGTTCTCAAGAATTTGTTATTCCACAGTGCTACATGGCGTTGTATGACAAATTACCCATGGAATTTTGTCTTGAACAAAGAGAAAAGATCATTTTGGACATGCCAATTGTATGTCGTATCCTATATGAGATCCTCCCTCCATTCCAAATTATAATTTGTTCTAGCTTTTCTAGATACATATATACCTTTTATTATGTAACTTAGACATTGCTTATATCTAGGTGCATAGCAAAACAATGTTCTTAAAACAGCCTAAACAAATTATAATTTGGAATGGAGGGAGTGTTACACAAACGATTATAAGAATATTAACATAAGGTTCATGTTCATTTCCTTCAAAGGTGATATGCTTGGTCATATTTTATTTTTTATTTTGTCCGATGGCACTAAAAGCAATCTCAGTAGGAAAATCTAACAATTTCTTTGCTGGGTAGAGAAATTACGTGACACTGCCTCACTGAGATCGAGACATGGCCTCATCAAATCGCTAAACCATACAAAACTCAGCCAAGCCGATAGTTCACGCAAAAGTTCAACACCATATTCATTTACAACTCATTATTCCACTCCAGAAAAAAACACCTTCGTCCTTCTAAATATATACCTTAATGTGCAGTACGCAGTTAGATAAATAAAGTACCATTGTGTCTTCAGAACTAGAAAAACAAAGCATCAAAAGCATGCACCCATGATATGGCACCCATATTATTGCCTTGAGCTCCAAAATTTCGGAGCCAAGAAAAAAAAATGATAATACGAGTTAACACTGGATACTTGAACAGGTACACCATGCATTAGGGCAACACAGGCACCAACAGTAACAACAATATTGCTTGCATTTTTAAATCAGACAGTTATACACTCTTACTCTTCATCTTAGTGTGGCTGTTAATATTGTGGGAACAAAGTAATCATGGCCAAGCATTTGTGTATTCAGTCCTTAGATAAGTTCTTCCATAATGTACAAATAGTTGAGATTCCAGGCTCTACTAACGCTGCCAAAACACTTCCCGCCTTAATTCCAGGCCTCAAAACAAAAAAGCAACATCACACTAGACCTCACCACCCTATAGCTGGTTTGCTTCAATGGACCGGAGGACGTTCCTCGTTAAAATGTCGGAACCATGTGTCGCTGGAGCTGGGGCTTACTGAAACTTCACCTTCTTAGTTGATCAGAGTACAAACTGAAAAAGAAACATGAAAAGGGGGTACAAGCAAAACCATAGGAGCAAATCAAATATCAATCAAGCCATCGCGCCATCACTAAGCATTATGCCTCTAGGTGTGAATGCCAGTGCCATTTGGGTAGTACCCATTTACCAAGCTGTTCAGTGGATTCACAGGTCCAGTTGGGTATAATTTCAGTTTCGGGACTTTATTGATAGGACATGCTTTTGTTGTCGGATTGCTGCGTTGAAGCAAAGGTTCTAGCTGTCCAGATGCCGCGGCTATCAGCCCTGTCAGCAGAGATGAATTGTTGATTCAGATGAGGCACTTAGGCATGCTCAAAATGCAGTAGCAGTCTTATTAGCATAGTGGATATCAGGAAAAAAAAAACTCATGGTATTTTTGCAAAGTAGGGTCGTGTTACCTAAGAAAAGCGGAGATGGCTTTCCTGGTCTTGACTTGAATTCAGGATGGAATTGTGCACCGATGAAGAACTTGTGAGAGGGAAGCTCAATGATCTGCATCCAACAGAGCACCACTAAGTGATTGAGAACGTTTCTTATTAAGTTCCTAGAAAGTACATTCAAGTCTACAGCATCTGAGAAATTTTCCAAGCCTACCCCCATGCGTTTTCCACTTTCATCTTTGCCGACGAATTGAAGCCCGGCCCTCTCAAAGTCTGGGACCATATCAGGGTTCACCTGGAAGAAAAAACAAAATTGTCACTATGTAAAAAGATTAACAGTGACATACATTATTCGATATGGCTAACAGACAATTCAATCAGGGTACCTCATATCTGTGGCGATGTCTTTCATCTACAGAGCTGGCATTGCCATACCTGAGATATATTTACAACAGTAATATTTCGCCACATCAACTGTACAATCATGCATTAGACTGAAGAATATGAGGATTAGTGTTTTCTTACAGTTTTGCAGATTTGCAGCCATTGACATGGAAATAGGTTCTCCTCGATCCAAGCCTCATTGTCGCTCCCATTTGGGTTTTTGAGCCCTGTGGAAGTGAGAAGTTTACAGTTCAAAATGAATGGTTAATGAAGGATAATAATACCAAGTAAGCATGACAGTGAACATAACCTACCTCTGGCATGAAGATGACACAGGGTGATGTTGTAGCTGGATCGAATTCCGTGCTATTGGCACCTCGTAGTTTCATCACCGAACGGGCAAACTCAATCACTGCAATTTGCATTCCCAAGCAAATGCCAAGATAAGGAACATTGTTCTCGCGTGCATATTTTGCGGCAAGAATTTTGCCCTGGACTCCCCTATCTCCAAATCCTCCAGGAACGAGTACACCATCTGCACCCTGAGGTGGAATTCCGTGAGACGGCTGTACAGAAGTACAGAACTTTCAAGAATTGCATCATGCTACCATTTGATGTATGCACTTACCTTCAGCAATTTCCACGCTTTTTTATGGGCTTCAGGGGTCTACACAAAACAACGCATCTCCAAAGCATCAGCAAAAGCTCCATTGAGGAATAGAACTGACTCAGTGCCCATGATAAAACTGACCTCTTTAGCTGAAGAATCTTCCAGATCGCAGGAAGGAACCCACTCGACTACAAGTTTTCTTTCCATAGCAACTGATGCATGCAAAAGAGCCTGTAATAAGGTTAATTCGACAAATTAGTCAGCAGGAAGAGTACGATTGTCAGGGGGTGCAACTTTGTTGTTTGTTCTGCTAGCAAGAAAGAGTATTCCCGGCACAAATCCATTGAATTAAGAGCACAACCACAATTTAAACAAGCTTCAAACAGAAAACAGCACAATGTAAGAAAACTGAAACTCTTCAAGCAGGTCCCAGCCTCTTCTCAATTCTCATTTGCACATGTTTATGGTACATTTATAAGGCAGTGCCATCTCAACGTTGCTCTGGCCTTTATTTTCAATAAGATAGGGTAGTCATGGTTGAGGAACTGAAAAAAACATGCGATTCATTTTGGTCTAGTATGTTTTTTTTTCACCGATTTTCATAATCAAACATTGGGCTGATGGGAGGTGTGGATGTACACCCCCACCACCCATGTTCGTTTTCAGCTCAAATGTATCCACCTATTTCTTCTTATTTATATTACCACCCAGTTGCTCCTAGTTGGTCAAGTTTTCATTTTAAAATTCAAATAGTAATAATGTCACCTTTATGACAGACAGGTAGGAATCCGACAGGCCAGTGTATTTTCCAACCATGGCAATCTTGACCTGAAAAGTTAATGTAATAGTGCTCACTTTAACCAACTGTTTGCTCAAGAGCATAAGGGATAATATTTTACCCCCTCCCTCCTGGAAACAATAGGAAGAACGATGATTCAAACCTACCGGAGTCTTCAATTTGTCGAACTTGGTGGCTCTTTTAGTCCACTTCCCTAACTCAGGTTCCCGAGGTACTTTCCCATCACTGCATACATAGCAGTAATAAAAAGTGAACACATGAGCAGTGCTCCTTCAATGTTCAAACTACTATATGAACAATTGATGTGATCATGCAAAAGTACATACTACTGAAGCTCTAAAACTTTCAGAATAGCTTCATGGGCCTTCTGGTCCTATAAAAAAGTTCCATTGTGGATGGTTAAATAAGATGCGAATTTCGAATTTATGGTAAACATTCCATAGGAAAAGAAATACCCTACCCTTAGCAACAAGGGGATGTGCCAAATGTTCGTCACGTCATGGAGGTTCACAATATTTGAGATCTGCAACATAAGTTGCATTGTCAGGACGAGAGCAAGGGTAAAAACTCAAAAGAAGCCTTGTAATGGGAAGCATTATCATTACCGGAACATGACAAAATTGTGAAAGCTTGACCTTCACATGTTTCTCCAATGGCTGCCGAAAACACAGAAATGACCATTAGTGATTCAGTTTTAAGCACTGACAAACAGTAACATAGAGGCTTTGAAGGACTGGAAACTGGAGAAAGGTGACTGTATAGTGTACCTCAGTACTGCGACATGCTAAAACATCCGGTGCCAGCCCAAGTCCTCTAAGCCCTCGAACACTATGCTGTGTAGGCTTAGTTTTCTACACAAATGTAACAGGCTGTCAATTACATATTTACATGAAAGTGATCAACAGAGTTTTTCTATT

>BAC-31

CCCCCCCACCCCGCAAAAAAAATGGAAATCAAAACAATGAAGCACCTTGTAAACCTTAAAATATTAGGTTCATTGATAATACTATCAACAATAACACTATGATGAAGCATGTGCTAAAAATCAGTACTAGATTGATCTGATACTTTGCATATAAATGTAAAAAGGCTCAAATCTATCCAAAACTTGGTGTTGTATGGCAAATTGGCAATCAAGGAGTGACAATATAGGCAGCAAGTTCCTAGCTGCTGGCTAAGATTGACAGAAAGGGCGCAGCACAAAAATAATGTAATCATAACAGAAAGCAATCAAACCAATATGAAGTGGGCAAGTCTAAACAAAGTTATTTAACTGGCCACATTCATACATGATTACAAAGAAGAGAATTGGGAATTTTGCTGTAGGAAAAAGCTGTAACGTATGTAAGTGGATGTTAGCTTAAAGTTAACCTACAGATAGTAACAAGCCCCATCCAAAATCTGTGATGTCTGAATTTCCCTATGGAAAAAAAACAAAACATATGCATGGATGTTAGCTTAAAGGTTATCTTATCTTAGAAAATATAAACCGCAACCAAAAATCTGTGATGCAATGGTTCTGAGATGTGTGAGGGAGAAAGATGGGTAAACATTACTCGTAGACAACATGTACATGTGAGTTTACTTGGGCCAACACATCGTTCAAGCAACAATCCAAGCATATAACCATCTCAAGTTGGAGGTAGTGTATAACTCAACTGGAACAGATCTGTACGTGAGCAATTGCAACTATAACAGGGAGCAAATGAGTAATCAGACGCTTACATGGCGGCACTTGCCCTCGATGAATCACTAGTCGTGACGTGAGACGCGTCCCCGATCGACCACCCCGCGCACAGGCCGGAGTCGCCGTCAACCATGACGCCCACGTACGCCCGCGAGACTTGTCGCTGCCACCGGACTGCGAGATGTCGAGAAGTAGTAGGGTTTGTGTTTGTGCGGCGCCCCGGCGCAGCAGGTGCCCGGAGTAGGGCTGGCGGCGGCGTGAGATCGGACGGTTGGTGTGTGCGGATCAGGGCAGTTATGGTCAGAAAAGATCTATATCTACTATATATATATACCACCTAAAAAATATCGTTGGCTTTCATCCAACATATCTTGGTGCACGCGCCCAAGGCAAACGTCTGATCGTGCCTGGTTCGGTCTCCCCTGTCTCCGACCGCAAGATGCCTTCGGTATGTACTAGTTTTTTTTTTCCACACGTCCTCGGTCATGTATTGCCATGATGTATATATGTGTTATGAAATGAAAAGGTTCCTTGCTTCTTGATTGGCACACATGATCCGCCCATCTTTAAGTTTGTCTTAAAATTAAAACTTGAATGGCAGCAGCAGCCTGCCAGGTGAGGCAAGTCCGTGCGGCCGTACGTGTACTCTAGCTGCATATATAGCATACACGCCGCATATATACTTCGGAGATATCCTGTTACATGGCCATGTCACATGTGCTGACTATACTTCAGTTTAATTTGCATACGGGCGCATGCATAGTGGCAGCCATGAGCTTTCTGGTCTGTACTTTGGAAGCATATCTCTTGCATCTAATATAATCTCTCTACACCTAAAAAGACTCTAAAGTCATCATCTATCCGGTGATACCAAAACCTTGATCCCTGCTCACGCATCCGCCTTAGATACAAATTATACTCCACTCGAAGATCACATGCCCACATCTTATCTGGTTTCAATACGAAGACCGATCCACATGTCCACATCTGATACGAAGGCCTCGGAATTCACGCATCCACGAGGTTCAATTTAAAAAATTATGACCCCAGTGCAACCGTTTTGTGTTACGGCAGTTCAGTTTCTTTCGGTCGTTTTTTCTTCTGTCCATTTTTTCCTTTTCCTTTTTTCTGTTTTGGTCCGCTTCTTTCCATGGCTTTTCTTTATTTATTTTTCCCAAGTTTTCGTCTCTTATGAATTTAATTTCTGTGATTTATTATGTTTTTTTAAGATTCCATCCGTTTAGAATACTTCCTTAAAAGAACAGCATTGACGACACAAGACAGAGCATACATCAAGTGTAAAAAAATTATGAGAGTCTGCACATGGTGTTAAGTAGATCGATAGTAGAGCAATTAACGAATTCTCATGAGATCTAGTTACTTCTTGTCAGGCTGATCTCTACCAACCATTGTGCGGTCGCTAGCATCGATAGCACAATCATGTTTTCTATATGCACACCACTCTTCGCGATTGCTAATTAAGGACACCATCGAGATTGCGAGTTAGCTCAACCGCTGCCGTCAGGCTATGTCCGGGCCATCGTAGGAGCCATGTATGCATGGGCACTATGCTGCCCACCCTGTTGCAGTGCAGTATAGACCTTGGTCTGTATGGTGGCCGTCGCGCCACCGACAAAGACTGCTTGGCTGTGACTGGTGGCACCGCACGTGCGTGCTGCCATGGCTGCCACGCCGCTGGTTTAAGCCGCCTCGCAATGGCCGTTATGCCCAATAAATTATATTCACGTAGCAACGCACGGACATTTTTTTGCTAGTATCTAATAAAGACCAAAATACCAGCCTTAACCGTCCGAGTCTGACTCACGAGTCACGAGAGTACCGTTCCCGCCCCCTCTCACCTCCACAAATTTAACTCCCTTCTTTCCTGACGCATCTTAGCCTGTTGTAACAGAACAAGAATCCTGATACACACACCATGGGGAAAATCAAAATCCCACGCCTGCTAATAGGGCGATACGAGCTCGGCAAGCTGCTCGGCAAAGGCAGCTTTGCCGAGGTGTACCACGTGCACAACCTGGGCACAATCAAGATCATGGACGAGGATCACTTGTACAAGTTAGGCGCCATGCAGCAACAGATCATGCACGAGATCCATATCATGCGCCAGGTGTGCGGTGTGCCAACCCTCACGTCGTGCACATCCATGAGGTCATGGCGACCAAGAAGAGCATCTTCGTCATTATGGAGTTCGGCAGCGACGACTCCCTCAATGTCCCCACCTCGTCCACCGCGTCGGCCACGGCATCGGTGAGGCCTCAGCCCACTGCGTACGTCTTCCAGCAGCTCGTGTCTGCGCTCGACTATGCCACTCGCTTGGCGTGTACCACTGCGACATCAAGCCCGACAACATCCTCGTCGACGCCACTGGCAACATCAAGGTTGCCGATTTCGGGCTCTCGGCCCTCGGCAGCACGGCACAGCGAGAGGCGCTGCTCCACACCGTCTCAGGATAGATGCCCATGTTCATCGCGCCTGAGGTGTTCTTGTGCTGCGGTTACGATGGCGCCAAGGCCGACGTCTGGGCCTGCGGTGTTGTCCTCTTCGCGCTCGTGGCAGGCCGCTTCCCCTTCAATCACAAGGACACGAGCTTGTACCACATGATCCGCCGTTGTGACTACTATTGCCCGTCGTGGTTCTGTACTGGCCTTGTCGCCCATCGGCCTCATCCGCCGCATCCTCGGCCCTTACCGGGCGCGCCGTATCGCCATACCGCAGGTCAAGAAAAATCTTTGGTTCAAGAAGGACTTGAAAGAGATCCCAGAGAGCCTCAGCGAGACCGAGTTGCGCGACTCTAACTCCATTTCTGACGACGAGTTGATGGCGTCATCTACGTCATTGGGGATACGACCTCCCCGATGGTGTGCCCCATGCATACCTCGGTGTCCGTGCCGTTGCTGACCACGCTCGAGAGTACCGGTAATGCCGCCGTCCAAGCCTAGCCACACATGCGCCGCCCTATGAGTCTGAATGCGTTTGACATCATCGCGTCCTCGCCGAGCCTCGACCTGTCCGGGTTTGTTCTAGGACCCGAGCAAGCAGATGCGGTTCGTGTCCACCGTGCCCATGTCAAGATCATCTCCCATTCTGCAAGCTAGAGGAAATCGTAGGGCACATCAGCTTCACGGTGCGCACCAAGGAGTACCAGGTGAGCATAAGGAGACGAGGAACGGGAACCAGAAGAAGAAAATAAGACTAGCAGCGCCTTCCTCTTATTCCACACACACTCACACAACTCAGCTTATCAGTTCCCAAAAACCATAAACAAATCCACGAGAGATGGCTGGGGTGACGCCCACGAGGAAGTCGTCGAGGTGCACGCGCTAGGGTAGGGTGTAACAAGTAAACGGAATGAACTACTATCCTTTTGCCTCAATATACATACAACCTGAACAAAAGGATGAAACATGACAACTCATTCAGACGAAGAATTGCTAACCCTGCTGCCAGCATCTGGGCACTTCCTTACCGTAGCAGGGACTCGCTGATTATTACCACCAACGCGAGGAAAGCACACAAGATTACCATAGCAGGAACTAGCAGATTAGCACCAACGGCACAGTGCTCGTGACGTAATCGCCTCTCGAGACTTGTAAGTTACAACAGTCGAAGTATGATAAACTGTACAGCGACAGCACCAAAGTCACTTACATGAGCATAACAAGGTCCATGAGAGCCTTGAACCAACTTGTAAGTTACAAGAGTCGAAGTCACATAAATAACCCATCAATAACACAAGTCTAACTTGCATATATCCTAGACAGGTCTGTCAAATACTAGTAAGCTAGATAAACAGCAGCAGCACGGCAACAGCACCAAAGTTTGTTAGATACTAGCTAGATGAGCAGAACTAGGTCAATTAGATACTCCAGCAGTGTGCTGTCTAATTAAGACGCCGCCCAATATGCTTGCCTAGATCAAGAACCCTCGCATCAAGTTCGTCATGATACCAATATGGCTCAGGCACCGGCCACCTGTCATCAAATATGTTTTTGTCCCAATGTAAGTTGGAAGTTGAATAAAATACTCTAAGCAAACAGAAACAAGTGGCAATGCGAAAACAGAAAGAAATACCTGTTCTTCTGAATCCCCTTCTTTGTCCCACCAATAACATTGACAGGCATAGGATGGTTATATCCACTGTCATCAAACAAAAAAAAATCAAAGTATGTAAAATAAGTGTCATGCCTACCAGTCAGCATCAAAATGAGAATAATACCATTCTGATGGTAAAGAATCCACTCCATTGAGAACTTCAACAATCTTCTGCCTTGGTAAGAAAGGAGTTACACCACTTCCATCGTAGAAGTTCATGCCAACAAAACTCCCATCAAGATTAATAAGCGGGCCTCCAATCCCAGCCTAGTTTTTTTTAAAAAAAAGGCCCAAAGTTACAGGAAAACTTGTAATAATTCCTAATGAGTTCAGTACATGCAATATGATTAAACTGGTTACCATATATAAGAACAGTAAGAGAGATTCACCACAGTACCTTCTTGATTTTACAAGTAGACAGCTTAACATCTTTGCAATCAAGTTTGCAATCCTTGTTTCCATGCTTCACTTCACCACTTGCGGCCATCAATATACCAAATTTAGCATCACGCCCTATAGCTACAACCTTTTCAGATGTGTTTTGTGCACGAGTGAAGATGTCATCCGGACAAATAGCATTGAAATTCTTCTTGAGACTGACAATAGCAATGTTATAGTCTAAATTACAAAATTCCAATGTCCCACCAGCACGTTGATTCGGTGGGAGAAACACCTCAATCTGCGATGAGAAAGTGAGCTCAGCAAACCATCACTAAACTTAAAATACCAAAAGTAATGATAAGGACTAAGGAGCACCAACCGTCAAGCTATTATCAATGCTATCTGCATTACAACAATCTCTAACTAAACTGGCTGAAGTCAGGATGACAGTGTGCTTAGGTTTGCTTCCATGCTTTATAAGCAAGCCTGTGCACGCAAAAGATCTCACATACCCTACACATTATAAATTTAATCATTAGTTAGTGAAGATAAAATACTAGCACAGAAAAGTAAGGAGCACACAGCTGGAAAAATCACCATTGAATGAAACAAGTGAGACAACACGACGGGATACATCTGTCACAACTTCTATTGGAAGTTGATTCCAGACACACTCCTCAGAGCCACCGGGTGGATCACCATAGGGAAACCCTTTCCAAGGAAGTAATTCACCAAAGCATTCTTCAAAATGATTAAGCAATTGCCCATTGACTAGTTAAAGAACGAAGATCAGAAGTGAACTACAAAAAGGCCAAGGGAGATGACAGCAATATCAAAAGCAACACTTACATTCGAGCACAAGGGGTGGTGGAATGGGATAGCCATGGGACTTTAACCTGCAAATAGTTTGCATAAACCCTGATACAATATTCAGATCATTATTAGTCCTTAGATGATATGTATGATACCTGTAACATAGTATTCTGCGCACAATGATACATAGGCAACATTTTAGAAGATGGCTTGCAGTATTGTTCACACCTGAAGGAACTACACTTAATACATCTTTAGGCAACGTATATCCACAGAAGTCTAGTTTTTTAGGACTGCAATAAAACATAGTTGACTTTTTATGACAAAATAAAAAACAGGAACTACACAAGGGGAAAAACCCAATCATACCAACATGTGAATCTAATCTTGTCTTGTGAAAGAAATAAAGGTATGAAATAATGACTAGGAATAAGGCGTAAATATTCAACAAAAGATGAGAGGATTGAACATATTCATGTCACAAATATAGGTTGCATTGTCCATATTGCCACAAAGAATTGTTTAATTCTTCCTAATTTAAGCCACAACATTAAGCATGGGCACTAAGTTAGCATCATAAGAACTGGCAATGGTACTGGTTTCATTAGAGAGGAGCAAGAAGAACCAAATTATTTTCAATCCAAAGGTTACCATGTGAAAACTCCTAAGTCTCCAATGCTGTTAGTAAAAATGGCTTACCTTAAAATAGGAAGTGGATTAGGAGACAGGGTTTGGAACAAATATGAATCAAGTTAACTGAATATAGTCAATCAATATTGTAAGTGTAGAAGACATACTTGATTATCCGAAAATGCTTCAAGCGTTTGCAAAGTAAGTCCATTGGAAGGAAGGTGTATCTCAAATTTGCATCAGCAGGGTCATAAATATCTAAGCTCATCCCGACAATTTTATTATCCCACCCCAGAAGTGGCCCTCCAAGTACAGCCTAGAACAAAAGCAAAGTGGTAGGTTTAGAAAGGATTTACTTCAATGCACACGGTGATACTTTAGACCAGCAATAACATAACGCAGTTATGTGTTCCATGTTTTCCATCATGCCGTAAAGTTACAAATAAATAAATAGACAGTATACCTTAGAGATATCTTGACTATCAGAAACCCAAGAGTTGAGGTGTTCGAGGGGGCGTTTCTGATACAGAGATCCACGCATGGCCATCAAACTGCCAGAGTTGAAGGCACGCCCAGCAGCTTTCAAACTATCACCAGGGAAAGGTTTTGCCTTCTGATCCAGATCTATAGGACGGACTCCTAGGAAGCCCAAGCATGTGACGATAGCAATGTCTTTATCATATAGTCCTAAGAACCCATCGGTAGTTGTATTATTAGGAAGGCGCACTGCAACCTACAGAGATAAAAGCAGTGCACAAGCACAAGCTCAGGATGTTCACAATTAGCTTGAATATACAAAGAAAAATCAAGGAACACAATGATTAGCAGCACTAACCCTCAACTTATCATCTTTATTTCTATTCTCGTTGAATTGTGTAACCAAATGTGCTGATGTCACAATTCTTGTTAGGCTTTGTCTAGTTCTTCCGTGAGGTATAGGTATGCCCGAGCATGCAAATAACATCTTATCTCCTGGACAATATCATTATTTAAATAGCAAGTGATGACTAAATGCAATTTATTTAGGAAAATTGAAAAGTATAGTAGCATAATCACCATCAAACAAAGCGATCGAGACGACCAAGGAAGACAAATCCACAGTATAATTAGTACTGATTGGATAAGTTATTTGACCAGGTTTGACTATTTCACCAAATGGCTTCTCCTCAAAAGCAACGTTTGAACCTTCATGAAAAGGTTCGCCGAATCGTTGCAATGCAATAGCAGATATATTCTCCTCAAAATTATGATAAGCCTCATAAGCTGCATGATCTGATTAATAAACATGATACACCACAACTGAGTGCTAGAGCAATATGCGCCACAAATGAAAGAACCAGCAAATTCAAATCAACTACTCACGTTCTAGCATCCTTTTCATGGTGCGGTTCAACGCTCTTTTGTTACCACTTGGGACATAACTCCGACTTGAAACCAGTGATGACTCAAGTTTGTTCCTTCTGTTTATGGCTCTCTGCTCCCTACTCACACCTCTTGTGCAGTCAATCCCTCCTTTGTTCCTCGTGGTCTTTCCAGTCCTGAAGCAGAAAATAAATGCAAATGGTTAGTACAGTAACTAAGACCCTTTTTTAAGCTTATACCCCCCCCCCCCACACACACACAACACCAAAAAAATAGAAATCAAAACAATGAAGCACCTTGTCAACCTTAAAATATTAGGTTCATTGATAATACTATCAACAATTACATTATGACGAAGCATGTACTCAAAATCGGTACTAGATTGACCTGATTCTTTGCATATAAATGTAAAAAGGCTCCAATCTATCCAAAACTTGATGTTGTATGGAAAATTGGCAATCAAGAAGTGACAACATAGGCAGCAAGTTCCTAGCAGCTGGCTAAGATTGACAGATAGGGCACAGCACAAAAAAAATCTACACATAACACAAAGCAATCAAACTAATATGAAGTGGGCAAGTCTGGACAAAGTTATTTAACCGGCCACATTCATACATGGTTAGAAAGAAGAGAATTCGGAATTTTGCTGTAGGAAAAAGCTGTAACGTATGTAGGTGGATGTTAGCTTGTATATGATGGATAAACATTACTCGTAGACAACATGTACATCATGTGATTTTTACTTGGGCCAACACATCGTTGAAGCAACAATCCAAGCATAAGCAACTCAAGTAGGATGAACAGATCCGTACGTGAGCAATTGCAACTATAACAGTGAGCGTCCCTGACCACCCTGATGAATCACTAGTCGCGAGACGTACACGTCCCCGACCACCCCGCACGCAGGCCGGCCAGAGTCGCCGTAAACCGTGACGCACACGTACGCCTGCGAGACTTGTCGCTGTCACCGGACTGTGAGATGTCGATTGAGATGTAGGGTTTGTGCTTGTGCGGCGCCCAGGCTGGCGCAGCAGGTGCAGCCGCGAGTGGGCTTCCCTTCCCAGTCGGGGCCAGGGGGAGCAGCGACTGCTCTACGCTCTGCTAGGGTTAGGAGGAGGTCCTGGAACCAGATGTAGCCCGGAATAGGTCGGCACTGTCTTGCCCGTATTGGCAAGGGCCCAACCTTCCGACGCCAAAACTGTCGCTGCCTGCCATGGGCGGCCAGTGCACGGAGATGTTTCCTCGCGCGAGGCGAAGCAGGGGGCCGGTGCCGGCGGTGGCGTGGGATCGGACGGTTGGCGCGTGCGGATAGTCATGGTCAGCCCTAATATAACAAGGATAAGAGTTATGTTCTTTATTTTGCATTGCTTTCTTTATTTATGTACGAGTTAGAATAGCGCACGTCTAGACAAATATGGAGTATGAATTCGAATCTAATAGATTCCTCAGATCTGAGTACTATAAATTTATACCTCTATATCTATACCTAATAATAAAGAGGCAAAATTTCCATCAAGATTTTTTTCCACCAAGATTTTTCCACTCCAGTATGCTTGTATATATTTTTTTCAATTCCAGTCCGGGTTGTCCTCTAGAGTTCGTGTCTGAATCCAATTCCAAACGCAGAGGAAAGCACCTAATAAACATCCAAAATTAGATGCAAACTTGAAGATATATATTAGATATAAGACAGAAAAAACAGATACGAAGAAAGAAGACATGATGGACACGAAGAAATAAGAACTATAAAAAGATCCAGCAAACACAGCAAAAACGCCGCGGGGGTTGTATTGTATAGGAGGCGGCTCCTCAATAGCTCGATGGCAACACCAGCGCCTCTCATGACGTGCGGGAGCCCCGCAAATGAGTTGAGTGTTGGTGCAGCCAGCCTGCCCCGGACGTCTCCAAGACTTCGCGCCCGATGTTGCTCGCGGATCTAACGAGAAGCGCGTCGCATAGGGAGATCTTGGTGCGGAGCTTTCGCGGGGAGGTCGACAATGCCGCGACCGGGGCAGGGAAGCACGCTCGCTGGACGCAGGCGTAAGCTTGGAGCGGGAGGTGGAGGTGGAGGATGGAGGGCGCAACGACGCAGGAACAAGAGGAGGTGTGTAATCAGAACAGGCACACCTCTACTGCTGGAGGCTCTCCGCCACGTTCGGAAACCCTTCAACGACGTCGATGCCTAGGGACCCGGCGGTGACCACGATGTTCGGCGCCCCTTCCACGGTCTCCGTGTAGGTGAGCTCCGGGCTCTGGAGTCTGGATCATATATTGGATGGATAGAGAAGGGTTGAGTTTTGAGTGTTCTGGAAGATGGAACGAGTGACGAAGTGAGCGTCTCGCCCGGCAGCTCCTTTTCCTATCGATGCCATTGATGCAAGGATTTGTTGACCTTAAGCAACGAAACACAATGGAGAATCCAACTCCCTACTCTTATCAATTGTTGTAGAAGTAATGGAGACAGCAGAGCGACCGAGGGAGCTCAGCTCGGATTTGTGCTGCAGAACTTTGTCGGCCATGGAAAGGAGGGAGATGAGCAGTGAGGGACGATAAGGATCACCAAGTTTTTTAAGCTGTGTGCACTGTAGCCGCTGGAGTCTTTCTTCGGTTGGCAAAGCTTCAATTAGTTTTCCTTTCTTTTCTATTTTCCCTTTCTTTTCTATATAATACATATTTTAGTTTTATAAATATAACTGATGAGTAGTTTCATGATTATCCTGTTTCAATACTTTGATTACTAGCACAATACAAAATATGTCGTAGAACAACTTATCTGCTTGAACAATGATTATATAGTACTAAACATTTCTAATTACCCTGGCAATGACAGATCTACCTACCAAACTGAATAATGATTTTTTCTACTTGAACAAGTTACTATTTAATATTACATATATAACCAAATATTAAAGTCAAAAGTGTGTTCTGCAACTATTGTATTTTACTTTCCATAGTGTGCTCTTGTGCACTTTCATTTCTCAAGCTACGTAGTGAGTCTGATCCCAATATATATTAGAAAAGATACCTCAGACCACCATTTGTACGCCGGCGGCACGCCAAGTCACGAAGCGGCAACTGGTCCCCGAGATGGATGGCGGGTCAGACGCCACCACAATCCTGGCAAACCGACGCTGTGTCGTGACCATTAGGGATATTTGGTTCTACACTTCTATTGAAGGTAGAGGCAGGCTACTGCGCTGCTGGTACAATGTTTTACTGAGGTGGCTTTAGCCACAGTCGTAGACACAACCTGCAACTTTAGTGGGACGATGAGGCAATTGTGCCAGCAACTACAGGAGGAAACAAACAGTGAGGTAGCATGACGAGGCAAGATAAGCGGGACAACAAGGCATTGACCAGTATGCGGCAAGGTAATAGGCGGAATGGCTATGTGGAGCTGCAAGGATGGCAAGGATACAGTGAGATAGGACAACAAGGCACGCGGGACAACGAGGCTAAGGAGGTGGGAGCGAGGGTGCCACTGGGGCAGGAGGCGAAGGAGGTAGTGGTGGAGTGGTTGGGTATATGGACAAGAAGATAAGATTATATGTACGGACCGTACATTTTACATCCAACTGTTAAAAAAA

>BAC-5

TTCAAGCAGAAGACGGCATACGAGATTCGCCTTAGTCTCGTGGGCTCGGAGATGTGTATAAGAGACAGGTGGTACATATTTATGTTAATGCCTAATCAGTTAGTGTTGTTTGATATGCAGAAATACAGGCTCTATCTCAAACGGTTAAGTGTTGTGGCGTCACAACAAGCTAGCATTGTTGCCGCTTTTGGAGGTAGAGACCCCTCATATTTCTTCACATGGGAGCCTTTGAAGGCCTTTAGAGTTTTGAACCTTTCGCCCCATGTTCCGCTCTATCATCCTTCCGCCCGCATGGCTTGCTAGGGGATTTTTATTTTTAGTCGTTTCTTAATTAATATTTTAATAATAACCACACCCAAAAAGATTAAGCAAAGCTGACCCCTTTGGTCGCGCCACTCGCACTGGCGCGACAAGGGACGCCACCGTGGCGGCGACCGCTGACCCAAGCTGGCGTGCCATTTGATGGGGCCCACCTTGTCACGCCAGCGACTCGAGCGCGACAAGGGGACATAGGGTTTCATGGGCCCGGCCCACTTTGTTTTATCTCTCTCCCTCTCCCTTCCTTCCACATCCCACAAGCGCCTGGGAGACTCGGGCACCGCCGCCGCCTCCCTGCCTCCCCGTGCCGTGGCCGGCCGCCACCGCCTCCCCCGCCGGCGGCTAGTGGGCCGCCTTGCTCCGGCCACCCTCCTACACGCTAAGTACAACTTCTTCCCCCTCGATTTCTTGATTTGGTTTCTTTAGCTCCATGACTAGGGTTACGAAATTTCGATATTGTGAATGTGTCTTTGTGTTCGTTGTTAGTATATTAGATGTGTACATAAGGTTTAGGATATACTCTGCCCCCGGATTCGACGTAAACTGTGTGTTGCATGAACCTAGGCAGTTGATACCCGAAGGATTTGGATAGGTTTGTGTGTTGCCTTTGTTGTCATCACTGCGAAGGTTGTGATATGTTAGTGTAGTAGTGAATGTACACGATTTGATTTGGAAATATGCAATTGATGTAGGTGTTGATGTGTTGCTTTCATATTTTGTAGATGGATCGTTTAGTGCGATTGTTTTATTTTATGGTGGCAAAGTTAAAGGAAATGAGGAGTTTGAAAGCTTGAATGAACAAGTTCAGTTCTTTAGTACACCTCCTAGTTTTGATGCTTTGCTTACCCAATGCCGGGAGAAATTTGGGTGGTCAATAAGTTTAAAAGGGTCGATTTGACTGTGGGAAGGAAAGAGCACACTATGCGTTAATGGCTTTGTCATATGAGGAAGAATGGAAGAACTACAAAGAAGTTGTGAAGAGTAGTAGTGTTAGGTGCTTGGAATTTGTTATGGACAAGGGGTCTAGCCCATTTGTGTTGTGTTTGGATGATAATGTGGATGTTGAGCCCGTAGATAACCTTACCTAAGATAAATTGTTGCAGTCGGTTATTGTTAGGGAAGTTGATAAGTCCTTTGAGCTCGGAGCCGCCGCCGAGCCAGACGCAGGCTCTGCAGCAGCAGGCACGAGGGGCGCGACTGGCCAAGCAGCTGGGAGGAGTGCTGCCGCTTGGGGTGCGAGCCGCTAGGAGGGTGCCGCTGCGGGGCGCAAGCCTCTACCGTGGGGGCTCGCAGAGGCGAGGTCACGGTCTTTGAGTCGATGCGGAAGAAAAACAGTATGAAGAACAAGAGAGAAAAATAGAGATGAAAAAGGAAAGCTAAGGGTATTATGATAATTTTATTCTCTTAACCATGCATGAAGCTGTTGTTGAAGATGTTTAGTCAAACATTTTTCCCAAAGGGCTTTGCTACGGACAGTTCAGCTTCACGAAAAAGCTGCTCATAAAGTTGTTTTCTAAAATTTGAACCTTAGCAATAGTAGTATATCCGTACAAACGAGCCATGTAAACTTGTTCTCTAGTATAATAGGAGTAGACGAGTAGTCGAGTTGAGTATCTACAGCATTGTTCATCTTTTGCCTGTCCAACACCTACTGCTGAGCTTGCGTGAGTTGATCCATCAATGCATAGATAGGCATCATCTCGGGAAAAAGCGTGAGAAAAGCTGTGCCACGGGAGGCTCTTCCCTGAATTTTGCTTTCTTTTTCCCCAAAAATTCATGCCCAAGTACTCATCACTTCTTGGGTCGTAATTACTATTTTGCTAGGTTCAATTATCATAGCTGTTCGGAATCCACAAACCATCCCGACCGATGGTCAGAATTTCTTCGAATATGTTCTTGAGTTTATTCGAGACTTGAGCAAAACTCAGATAGGAGAAGAATATGGTCCCTGGGTTCCCTTTATTGGAACTATGTTCCTTTTTATTTTTGTTTCGAATTGGTCAGGTGCCTGTAGCCACCTGCTCTCTCTTGCCTTTTTGTTTTGACTCATCCTGTCATCTTTGCATCGTGTCTGTGCCAGGCCATGCACTCGTTACTGTCTCGCACGCCCGCGGTGGTTTAGGCCGCCGATGCAGATTTGTCAACGCGCGATTGCTGAGAACTTTTTCATGGCGTCCACCGTGCTTGCAGGTGGTCGGCGATCACAACGCGCGTCCCGAAGCGCACCGACAACGAGATCAAGAACCACTGGAACACGCACCTCAAGAAGCGGCTGGCCAAGATGGGCATCGACCCGGTCACGCACAAGCCGCGCTCCGACGCGCTCGGGACAGGCGAAGGAGGGCCCGGCGGCGGTGCCGCGGGCGCGCAGCACGCCGAGGGCCACACGGCGCAGTGGGAGAGCGCAAGGCTCCAGGCGCGCGCGCGCGAGGCCAGGCGCCTGCGCGCGCTCGCTGCCTCCACCTCCGCGTCCGTCTCTGCGCCGCCGCAGATGCCCGGACCCGCCGCGGCGCACCAGCTCGACTCGCCGACGTCCATGTTGAGCTTCTCCGAGAGCACGGCGCAGGCCTCAGTCCTGGAGGCGCTCCGCGCCGATGTTGCGGCAGCGCGCGCCGCCATGCAGCCCATGCAGGCGCACGAGGAGGCGCGCAATGATCATCTTCAGCAGCAGTGGGGAGATCACCTTGTCGATGTCACTGACGCAGACATCGCCGCCTCCGCCTCCGCTTCCGTCTCGACGCCGACGTCCATGTTGAGCTTCTCCGAGAGCGCGGCGCAGGCCTCGGTCCTGGAGGAGCTCCGCGCCGCGGTTGCGGCCGCGCGTGCCGCCATGCCACAGCCCACGCAGGTGCACGAGGAGGCGCGCAAGGATCATCTTCAGCAGCAGTGGGGAGATCACGTTGTCGATGTCACTGACGCAGACATCGCCGCCTCCGCCTCCGCGTCCGTCTCGACGCCGACGTCCATGTTGAGCTTCTCCGAGAGCGCGGCGCAGGCCTCGGTCCTGGAGGAGCTCCGCGCCGCGGTTGCGGCCGCGCGCGCCTCCATGCCGCAGCCCATGCAGGCACACGAGGAGGCGCCCAAGGATCATCTTCAGCAGAAGTGGGGAGATCACGTTGTCGATGTCGTTGACGCAGACATCGCCGCGGCGGCGTTCGCGGGCATGCTTCTCGACAGCTCCTTGATCCAGCAGCAGACGGAGGAGGAGAAGAAGTACTGGGAGAGCATAATGAACCTGGTAAACTCGTCGTCAGCGTCATTGCAGACGTCAGTGGTGATGCCCGCGCTCGAGGCGTCCTCCTCGTCGGCGTCGTTGCAGACGTCAGTTGTCGCGATGCCGGCGGCCGAGGCGTACTCGTCGTCGGTCTCGTTGTCGACGTTAGTCGCTGACTGTGCCCGCGCCCGAGAGGCATACTCCTCGTCGGCGTCGTTGCAGACGTCGGTCGCGATGCCGGCGCCCGAGGCGTACTCGTCGTCGGCCTCGTTGTCGACGTTAGTCGCTGACTGTGCCCACGCCCGAGAGGCGTACTCCTCGTCGGCGTCGTTGCAGACGTTAGTTGCTGACTGTGCCCGCGCCCGAGAGGCATACTCGTCGTCGGCCTCGTTCTCGACGTTCGTCGCTGACTGTGCCCACGCCCGAGAGGCGTAGTACTCGTCGTCGGCCTCGTTCTCGACGTTTGTCGCTGACTGTGCCCGCGCCCGAGGCCTAGCTACTCTCCGGCGCTACCCGTGAGCCTTGGACATGTTCGCCGGCATGCAGCCCGGCCGGGAATTTAGAATCTCTTACTCGTTCATCAGTTGCCAAAGTTTCCTGTAGAACACATGTTGCCAAAGCGACTCGTCTCGTCTTTGCCCTGTATGTAATTCGACAACCATTTTCATTAGCATGCAGTATATTGGAGCAGTGGAGCTAATCGAAATGAGAAGAACTCTTATTTCAGTTATTTGTTTTTTTCATCTGAACCCATATATATTAGAATCCAACTGTTCAATTTTGTATGTTGTGGCACTATACTACTGATAATGGATGTACTTCGCACTGTATATTGGTCTTTGACACTTTTTTTTAGATTATTGGCCTTTGACTCTTTGTATTCTGTAATTCTTAAAGGAGGTCTTTTTTTTAGAACGCTGAAACAATGGAGCAGAGGGATATGCCTGCATTTCGTCATCAATAGAGCCGTTGCAACACATTGGCCCGCTCTTCAAACAGGTCAAGCCCATGACAGGCTTGGTCCAAATTTTATCCAGCCCGCTTGGTTTTTCCCATTTGGAGTCGAGCGGACCAGCTCTGCTCAGTCTTCGGGAAGAGAAAAAGGGTAAGGAAAATAAGAAGGCTCTCGCGCAGGTTCGTTGTTCCTTTTATCCGCGGAGAAAGAACCTTGGCCCGACACCATGCTCCCAATGACCGTGCACCCCACGACCACGCCGGCCTTGTCCCCGCGCGCCCATGTCTCCCAAGAGCAGAAGGCGGCCACTTCGCTATTGTTAGCACCCAAAATTATCCGAAGTGCATAATGGACTCCATGATCATATTTCTATCATCAAGACAGCCACAAAGCATACTTAGATTATCGCATTTGGAGCCCGGGTGAAGAAAACGGCTTTTCGAGGCGGGTGGGGGGGCTGACCTAGCCTGAATCGTCTTAGCCAGACTCTCAAAGTCGGCTAAGCCCATTTTCTCAATTTTCTCAAATTAAAGAATGGATTATACGTTGCTACTCTTGCTCGGAAGGAGAAATTTAATGAGTACTGTCTTGAGATTAAAGCTGGTTCAGATAAAGCGAGTGTCCAGCTGACAAGACCGAACAAATCAATGTCCATTCAGAATCGATTCCATCTCTGTTGTTGATTCCAGAGTGAAGATTTGAGTCTTGAATCGATACTGTTGATGGGCACAGATGGGTGGATGGGAATCTTGTAACGCACAGATGTGTTGTTTGAGCCTCTACCGACCTTTGTATGGCTAGCTTAGTACTTGTGCGGGCACACAAAAAAGTACCGTTAGCCTTTTTAATATGTCGATTGGAACATAATAATGATGCCCCCTATGGTCTTTTTGTTTTGCAGTAGCCAACATCATAGTATTATATTCTAATATAAGTTGTACTCAATCACCTCAAAGACCGTCTATAGTAAAAAACGGCATCATGTTTTTTTTACCCAAGGAATAGTAGGTAAAAGGGGAAAATGCAAGCCGTTTTTAGAGTAGATACAGGTAATGCTATGAGTTTTAAGCTGATGGCAATCTGATACTCATTTTTTATATTACATGGTACGTTCACAGAATCACACAGGTCATACAATATTTGAAGCTCACAGAAAACTAATGGCAAACGCAGATATTCCACCGAACTTTTTCACACAAGAGACCCAGCACATCTATAACCAACACACAAACTCATGATCATGATCTGCAAGCAAGAAGGATTGCAGAAGCAAAGAGATTTTATCTTTAGCCAGCACCAGCTGTGCACGCGGTGTCACTCAAACCGTCAAGGCAGAGTAGCTACCTGGGAGGTCTTGCAGTTCTCCAGGATGGCCTTGGACTTGGTGTAGAGCGACGTGAGCTGCCCTTCCCGCTGCAAGAAGCCGCGGCTGATCACCCGGATATCCTGCTTCAGCTCATCCAACTTCTGCCTCTCTGTGCCCAGCTCCCCGGCCAGCTCCTGCTTCTCAGTGGCGAGGAGATGGGCCTCCTGCCTGGCGGTGTAGAGATCCTTCTCGAACGCTGCACACTTCGCCCTCACGTCATCCAGCTGTGCCTGCTTCTCGGCCACGAGGCTGTCAGCCTCCCGCTTGGACTCAGAGAGCTCCTGTTGCAGGGACAAGTTCTGGTCCCTCAGCACGTCGCGCTCTTTCTCGAGATTGGCGCATTGTGCCTTTGATTCCTCAAGCTGTTGCCGATCAGAGATGCACTCCTTTTCTAACCTATGGCATCTGGTATCAGTAGGTTCCTCCAATGGCACAGCCTGCAAGCAGAGAGCAAAAAAGGACAGAGAAATAGGCAAGTCGTTTCTCAGTTCCAACATATTCCTTATATGGAAACAGAAAGCAAATTTGAACTGAAACAGAAGGGTAAACTAGCTTCTCAATCTTTTTATGAAATATAAGCAATTAATTATAGCTTCTCGATCTTCTTAAGCTACAGGTAACAGGCCCAGTAATATATGGAGAAGGAAAAGGAACCAGGTGACGGTCCTTAGTGGCTAGAATTAAGTATCTTGACTCAAGTATGTTGCATCTTCTTGAAGCAAATTTATCAAAACCATCCAGACCTCAGATGGTTTACCATGATCAAAAACTTGGATCGATATTATCCATTTTTATAAGACACATTTCCCCTGATGAACTCTGCCAACTAGCTATTACAGACTCTAGACACTGAAGCAGTCTCTTGTTGGATTGAGAGCTTACTAACTAGCCTATGCTTGCTTGGCAAGTAGCGGCAGATAAATAGGTAAGATGCAAAAGATCTAACTGTTGATTATATTGCCTGTTACATAGTGAGCTAGCACATTCAAATCCTAGCTAGATTGGTGGGAGATATGGGTGTTAATACCAAACCCTACCCAAGTTCAAGCCCTATTGAATACGAAGTTATCTAGTTCCTGCTTGGTAGTCTGTTACTCTACTTTTGTAACAATTTATGTCCAATCACTTTCTTGTTCTATGAATATGGACAACCTAAAATATTTGTAAACAAACAGATTAGAGCAATGATGCAGGAACAGAGAAAAACTTACTGATGTATCTTGTATAAAGGATGCCTCGCGTTTCAGACCAGGTGTCATCTCAAGCATGTCAAGATCAGTAACACAACCTTTGTTGAGGCGTGTCCATAGATCTTCATGAGCGATATTATCCAAATTTTCAGGTAATGGGCTGAACTCAACATTGTTTGAATAACGTGAGCGACAGGCAACAAAGCCATCTGGTACAGATTTCGAATCTGGAGTTTTCAGTGCATCCAGCTGCAACAGGCAAACATATACTTTCATTATCACCAGGGACTTGCATATTAAAAGTAAGATGGAAGAGGGTGGATATGGTGACAGACATGGACCATCAGTGACATGTGCTGTGGACAGCTTTTAGCCTTCCATACAAGACTGAAATTACTATTTGTTACCATGTTTCTTCTCTATCAAATATACCTGGATTGAATCGGTAAAGTGGTCTGAAGATATGGCATCTAGCTTCTTCTGTTGCTCAGTCAAATGTTGTTCTAGAGTCACCCGTAACTTTTTTTCCTCTTCAAGTTCCATGGCCAGTCGATCACGCTCAAGTTCAGACTACATGAAAAGAACACAGGTAGCTTTATTATGTAGTTATTAAAGTTGTGGTTTATTTATACAAGTTAAAAAAATAAGTAGTTCTTTGATGCGCCTTCAGTGTGAAGAATAATCATAATTTGATGAGAAACAAAGTTTTATTATAGCCATTAGTGTTTACACACCTTGTGCATGTCATTCCGTAGTTTTAGTACAACCTGCTCCAACCCTTCAGAATGGGAGCCCTGAAATTCATGAAACATAAATCAAGACTCAAATTCATACTCTTATGGAGAGCAATACCAACCTAACAGTAACTACCTGCAGTTTTTTACGAAGTTCCTCTATCTCCAGCTTTTGCCTCTTCAGCAAAGCAGCATCTGTTAGAATCTGTCACAAAAGTGGCCAAGTTAGGTTCTCTTGCTACTTATAAACTATATGCTAACAACTAAAATTGAATAGACTCCTTACTGCAAATATAGAAGCAGGAGCAATTTTTGGATTGGCATCAGAGCATTTACAAGAACGCATTCCAGACATTTGGAATAAAATTTTCACTTTCAATAAACTAAAAAAGAAATTCTACCAATGTACCTATGACCACTCACAATGCATTTACATGAAAATAATCACTAACATAGCTCAAGCAGTTTTTAGAAGATTTAACACATACCTCATTTACTTGGGCACAATTGCTGACACATTTTGCTCTACTTGCGAATTGAAGAGTTCCTCTAGTTTCCTCAATGTGAATCTGTAGATAAATGTCAATTTTATTCAGATTGTGCAACAATAAAGAAAACACTAGTCTGTACAGCAGTTTGCCCAGATGCAACAAATGCAAGTGTATAGACAAACCTCTTCAGGTGCAGCAGTGCAGATGATAGATGTCTTTGCATTGCCTCCAAGTGCAGGTTGGAGAATCCGTGTCAACTTACTATCACGATAGGGAATGTGCCCTCTGCATAAAAATATTCACCTAAATACAACTAAACATAGATCTTACAAAGTTTCATATGATTGTTGCATCCTGTACTAGAGTAAAAGTAATATGAATAATTTCTTTTTCTTTCATAAGGTAGTAACTTTTTTCATTAAGAATACGACAATGATCAAAAACTCTCCATAAGGGATAACTCTACACAACTCAAGGCAATAACAAGATCACTGAAGAAATAAATAAATGAGCAGATTGACTGCAGTACAGAAGTGCACACTGTCAAGAACAAAAAAAGTGTTTCTCTAGCTAGTGGAAGTGGAGAAGGTATCAGTTTCTTCACTAAAGACACTATTATAATGATTCAAAAGTCACTAAAGATGATAAAGAAAGCTCCCACTTGTACTTGTTTGGATAATGAATTTGAGTTTAATACACTAACAAAATCATCCTAACACTTGAGCTGTTGACAGAAAGTGGACTCAGTATTGATCATTATGAGACCCTAAGGCAGTAATAGGATCAATTAAAAACAATATATAAACAAGCGAGAATATGGCTTATGGCACGGAAGTAATGGATTAATTATGGAGGATGAAAGGTTACACAAACATAGTCAAGAATGACCTAGGGTTTGGCTTATAGAAACAGAACACTTAACGGTAGTCCTTGCCTATAACGAATCAATCATAACCATCCTAATTACAAGTGGGCGCATGGCCCATAGGGACATCAGCCGAGCAGCCCATTGGGCCAGCTATTCTGTGTCATACTGTCATCTAACATATACATCTCTAGATTGATTTTCCATCAAATTTAGCACACTCCACCTAAAGGAAGTTATAGATTTGGGAAACACATGAGGAAGAAGAGGTACCTTTGTTTTCCGTTCTCACTCAACTTATTGATGACATTTCCAAGAATCATCAAGCTCTTGTTAATATACTTCCCCTCATTTAAACGTACCCCTTCTGCCCCTGTCTTCATGATTCTTTCTGACCCAGCAAGATCCACCAAATTCTAGATGAAAAACAAAAGTGTAAATCACAATATTTTCTACACATTTACTTTAACAACATACATGATACCTGAGTATAGAGTGAGCAGTACATACCAAAACAGATACGCGGATAGCATCCCCAGAGTCTGTCTGGTCCTTACCACTACTTTCAATTACCTGCCAATATAAAAAGAACATTCTTGATTTAGTTATCGCTTCCATTCAGCTACAGAAAATAGTCTTGGAACGGCTACAATACAGAACTATTTTGGAATTCAGAATTCTGTACCATTCTGAAAATAGTGTGTGACCGACTGCTCCGCACATTCATGTTGGTCTCTCCAAAATGCCTATTCGCTAAAAACAAAAAGAAATAGTAACAAACGGTTACACATGTTGCGCATATGGTGAAAAATAAACCGTAGGGACAGAAATGTGCCTTCTCCAAGCTGGAGGAGCTCAAGCACTTGCTCTGCGCTGTTCACAATCTCCTCCCGCAAACCGGCCACATACACTCCACGCTACAGGCAACAATAAATTTTCATTATTACAAAAACCTGTCCAGATCCAGAAATCAGAGAAAAAATATCCAAATGAGTACTTACCTCTAAGCTCTCATGAATCCGCAGCTTTTGGCCCTCAATTGTCAAGAGATCGTTGATCTCCTCGTTGTAGATCTCCATGTAGGACACCCGGATGAGGAACTCGCGGTCGTCAGCCTGACCCAGGCAGACCCAAACCCAACAAATCACTCACGTGCATCAGAAACAATCGCAGCAACCAATCTTGTTCAGGGAGCAGTTAATCGAGCAACCAACGCACCTGGCGCACGGTGTCGAAGACATCGCGGACCGCGCGGGGAATGATACCGGGGTCGGCGTCGGAGCCGTTCATGGTGAACGTCTTCCCGCTGCTGGTCTGGCCATAGGCGAAGGCGGTGCCGTTGAACCCGCCAACGACGGCGCCGATGAGTTCCCGGACGAGCGCGCCGTAGATCCGCTCGTTGGTCGCCGCGCCGTCGAACACGTGGTCTGCGGAGCCAACCGACCAAGCAGCCCAAGGAAGGAGGCGCGAGCGGGCGGTGTTAGGGGTCGAGAGCGAAAGTGAAGGAGAGGGAGGTCGTAAGAGGAGGGGGGATCCGTACCGAAGGCGAAGGAGGCACCGGGGACGGGGGCGGCGGCGCGGTGGAGGAGGGAAACGCGGGTGTCGTCGATACGCCACTCGCGGTCGCCGCCCCCGCCGGCGCCGGCCGGGGA

>BAC-35

GATAAAGAGTTGTGAACACCAAAAGTAAATCCTGACTCGACTACCACCCTTGTTGCACGGCACAGTTTTTAGGGGCTAGGGTGGGGAATTTTGCTATCATTTAGTATACATACAGAGTCGTCTCCATTTTCATCCGGACTACTCAATATATTAAAAAGAAGGGTATAACGAACATTTAAAAAAAAAAACTACTGATGCTACATTACAAACACTAGTCTGCTCTACATGATTGCACGAAAAAAATGAAACGGTATTATGCCAAACATTTGACCATATAAGGCCCTTCTAGCTCATAACCCAATTTGCGGTAGTAGTGGCGAGTACCAACCCCCGAAATGACAGCCAATTTCTTTGAACGGTGCTCCTTTTGAGCAATCCTTTCTGCTTCTTCCATTAATAGAGTTCCATACCCCTGTGGATGTACACGGACACAAAACAACGACAAAAGCTGTATAAGAACCTTCTTGACAATACAAAGAATGAACAAGGAGAAAAATAATATTGACACATTAATAAAGACTAAATGCAGTCATGTGTGTCTTCCCGTCACTGACACTAATTTGCCTCTTCAGTTTCACTAGATTGAACACATTCACATTTTTACTAAAAGGATTTTTCACGTCATTTAAGTTGGGCCTAAGGCTTAAGCCTGAAAAAAAAAATGAAAACATCTGTTGTAATAAAGGATGAATGGCTAAGTCAAAATGCTCATGATGCACCACAATTATTCCATTACAGAACAAATGGCTTAGAGCATCACAATTAGCAACCTAGGAATCAGCCCAAAAAAAAAAGATTAGGAAATTTAAGGAATCTGCCAGAAAGCACTAATTTTTATTTTCAGAGTTGCCCCCTCACTTAGCCCAGTGTGTCGGAGCATGTTCAAGCCTTAGTGGTGTTTCATCTCTGCTGTGACCCAAATGACTGGATTTACTAACCACTGAGCTTTTATCTACTCCAATTAACATGTATTGCACGATTTTAGATTTTACTAGCAGTTTTAAAAACTTGACACTATTAATAATGGTAATTACAAGTGCCAAGCATGCCAGGAAGTCTAAACATAAATTTCAAACATGGTAGTGCCAACTGCTGAGGATCAAAACCTCGGGTGGTGATCATACATTCATACAGTCTACATGTAAATAAGTGAATCACCTGGTGCTGTAGCTTGTCTACATCACGGCCATGCACAGGGACTGCAGTTCCATATACATGAAGCTCACGAACAATTGAACACCTCCCTACAAGTTCAGGGCATGTAACATTACGGCCACATTTACGCAAGCGCAACAGGCCGATCAGGATATCCTGTAAAATTCATGAAAAGTAAATTGTGAGTATATTGCTAAGGTCAAGAAGCTACGACATTATGGTTAGCTAGACCCATTGCTAATCTCCATTGAACAAAAGGAATATCAAGAGAACCAATTGAAAGACAGAATAAAAAGATCTCAAACTATGGAGAACAGGAGTAGACTATAATCACATCAGAAATACGCCTATACAGGAAGGCACCATATTCCAGAATTCGCATCCCTATGATCAAATTGGCTGGTCAGCTATAAGTACCTGTCGTGTATCCTCATAAGAGAGGAACGTCTCCCAACCCTCATTTGCAGCATAGTCACGTCTAACAAGCTCTACTTCATCTGGCCTGATCTTGTGATGGATATCCTACATACAGATGTACAGCTAGAATAATAAAAGGACATCCATTTTAGGCTGTGAAAGTGGTTTTACTGTATTTCGTATTTACCTGAATCCCTGCCTCACGTGTTCGGACATCTCGGCATTTTAAGCCCAGATCTTCCATTCGAGCCAAAGCAAGCTCGCGAAGGTTACCTTTCTCAACACCAGAAGTGACAAGAGGCATAGGAATATCTCTCTGGACCCGATACACTCGTGTCCATGGTGGGACCATAGACAGAATTCTTGCCACGATATCCACCAACAGTTCAGGTGGATAATTTCTATATCTGCAGCACAGCAAAAGTTACCGGGTTAACCCTGGATTTATGAGCCCTTGTCATTAGTACAGAAGTTATTATCCTAAAATTTAGAAATAACCACAAGAATGATAATATTTAGCAAGACACTGAGTAATACTAGATTAGAGAAGTACCTGCCAGTTTTCCAGAGCTCATAAAGACCAGTTCCACGAATAACAAGTGTTGGATAAATCTTCAGACCATCAGCCCGGAATGCTGGACTTTCAAAAAATTCTCTGAAACTTTCCAAGTCTCTTTCAACTCCAACATTAGGTAAATCTGGCATCATGTGGGCAACCACCTGTCAAAAGGGTGAATCAAAACAAATAGAACAATCATAAACAAACAATGAAAGCAATCAGACAAAGCTATGTGACTAAAAAAACAATAACCTTAAAGCCAGCATCTTTTGCCAAACAGAAGCAATCAGCAACAGCAGCCACCGTGTGTCCTCTGTTTGTGTCACGGGCAACATCCTCATATGTACTCTGGACACCAATTTCCAAGCGGGTACAACCGTAAAATAGCATCTGGCGCAGATGAGGCCCCAAGCAATAATCAGGTCTCCTAGAATTCCAAGAGGAAAAAACATTTCATTGGGATTAAATTCCTAGGGAAGTGGGAAAGACAACACATTCCTTTTGAATGAAAGTAATAAGAAGCACCATCCAATTATGCAATGATACGTACGTCTCAATCGTCATACCAATACATTTGACAGCACTATGTTCCGAATAACAAATAGCCTCCTCAACATTTGCTGAAGTGTGACCCGATAAAGCATCATGAAGATTTCTGATGAAATAATCGCGATAATCAGCTGGTAAGGACATGAAAGTCCCACCCATCAAGATGAACTCGACCTGCAACATAAATTAAAATCATCTTCAACTGCTGGTCAAAATTTAATACAGCATGCCGTGAATATAAGAATCAATAAAATTGAGCCGAGTGCTGTCTTGGACTCTAGACAAACCTTATCCACACTATGCCCTAGCCTCTTGAGCTGATCTATCCTGCTCCTGGCTTGCACATATGGATTATACCTAAAATATAAATGTAGATCAGGAAAATTATCTACCAAACGTGTATCAATAAATCAATTTAAATAATCACAGTGCACGAACACATATATCTTTAAACATAATCCATGGCTACAAGATTCTCCAAAGATCCCTAGTTGGCATTGGTGTATCTTCATTATTAACAAGCAACAAATAGAAAACTTTACTTTTACCTTTTTTAGGGGAAATCGCTACTGTGCATTTCATCATAAAAAAAGTATGAAGAGAAAAGGAAGATAAAATCTGTATAGCTAAAAGGTAGGGGGGGAAAAAAAGGCATGCAACGGGCAGCCAAGAAAGAGAAAAAGAGCACACAAGCTAGTTAGATAACAGGTTTAACAAGCAACAGGCAGTCAAATAGAGTACTTTATTCATAGTTCTTAAACGTTTGGTCATCATTTGCACATATTTTCATTAAAATGTCCGGTATCTAATTTAGCCAGTCCATAATGGGTTTTATGAACCAAGACATGAAGTACTGTTTGTTTCGCTGGTTTATTGTGGCTGAAAGTACTGTTCGCTGGTTTGTTGTGAGAGAAAAACACCGCTGGATGGCTGCTGATTCTGCTGATCCAGCGAAACGCAACTGTAAGTCTGTAACACAAACATAAGTCACTGCACTCCTAGGTACAGAAAGGCTTAAATAAAAGCAGTTAAAGGGAGCATGGACACCAATGTACTCCAGTGAAATCACCTTGCCCGAATGGCACGCATGCTGGTGGGCTCGTATCCAGTGTAGGATTGCGTGCTGTACTCGAAGTCGGAGTCGGGACCGCCCGGGCAGTAGACGCAGATGTTCCCGGTGGTGGCGATGTGGGGGCAACGGTGCGGCTTCGACATGACGGCGACGACGGCGATGCCCGACGCCGTGCGCACGGGCTTGGCGCGCAACCGCGGCAGCAGCGCGGCGCGGTCGGCCTCGGGCACGGCGGCGATCATCTCCACCAGCTTGGGCGCCCGCGCCAGACCGTATCTCCGGCACGCGGCGGACTTGAGCGCGTTGAGGTCCACATCCTCCCCGCGCCGCGAGAGCTCCCCCATCGCCGAGACGATCTCGGCGATGGCCCGCACCCTGGCCTCCTCCTCCGAGAGCCCCGCAGGGAGGGCCACGCCCCCTCGCCCCGGCGCCGGCTTCCGGCGGCGCGGCTGCTCTGGTGCCGCCACGGCGGCGGCGGCGGCGGTTGCCATCGGGAGAGAAGGGTTTCCGCGAGGGGTTTTGGGCTCGGGTCGAGAACGAGTTGGATTCAGTCGGGTTGGTGTTGGGCTCGGTCGGGTCGTTGTTCCTCTCGGCCCTCGGGTACGGTTCAAAAGCTCTCGGCTCTGTACAGCCGTCAGATCAAGAATGAACGGTAAGGATTAAAAACGTTGCTTAGCAAAAGTTGTACTAGCACGAGTCTCTGAAAACATATTTCTCCCTTCCTCCCTTAGTTCAAAAAAAAAAAGAAGAAGAAGCCTCCTCCTCCTCCTCTTCTATCCCCGATCTTCTTGCGGCGGAAGCAAAGCGAGGGCGGCGAGATGGACGGCGGCGGAGAGGACGGTAAGCAGCAGCCGCACCTGGTACTGGCGCACAAGCTGTTCCTTCTGTCGCGCTCCGACGTCGACGACCTCACCAAGGTCGACCTACGCGCTGACGTGCTCGCGGCAGTCAAATCCGATGGTATACCAGCGCCCCCTCCTTTCGTCGTGTCTGCTTCTAATTGTGGGATGGGTTTGGATTTGATCTGTACTGGCTGTGGTTGGTGCAGACATGGCGGCTCTGTACGAGTCGTTGGCGGCGGACGGCGTGCTGGAGATGGACGCGGCGCTGCTGGCCGAGATGCGCGCCAGGATAGACGAGGAGATCCGGAAGCTCGACGAGAAGTGAGTAGATCTCGCCGCTTGTGACCTTTTCCTGCTCGATCAAAAGCTGGCTCCTAGTGTCGTATGCTGCGATGGTTGATGCATTTAGCTTTGTGACCGCACTGTTCCTAATTTGCTCGGTTCAGTGATAATTTAGCCTTGAAAATCACTGGGTTTGTCAGTTATGCACTGCTAGGCCCTATTACTTTCTTTGGGTTGGATTTGATTTCGTTTAGCTGAGTAGTTGTTCCAGTTGTCTCTGAACGTTGTACTTAGTAACTCTTGCACAGGCCGGAATATAATTTCTTCCTTTTTCTAAAAAATTGTGTTTAGCCATAGCAGTTGTGCTTGTCAGAACTTTATCAATGGCTTGTGTGTCAGCATCAAAAACATGGCATTTGTGCTTGATACTTGATAGGGTAGTGTTCATCCATCCATCTAGATGGAAGGGGGATGATTAATTTTTGTGCTTGATTCAGTAGTGTCATCCATCCATCTAGATGGAAGGGGGTGATCAGTGATTGTAGATAGAGAACTCATGTTCACACATCCAACCATCTAGATCATGAGAATGGTGGATGGTTGTAAATAGAGGTTGTTTTCTTCACAAACAAAATCAATGATATACTCTCAAGGAAGGCTGACATTTTGTTGAAGTAAAACAAAAATTGTAATCATGGTACCTATATTCTAAGGCTTTGTTTGCTAGGGCTCCTCCGTTTTCAGTTCCAAACGGCTTCTACTACTAAACCATTTGGTAGGGCTCCTCTGCCGGAGCCGGAGAGGAGCCCTACCAAACAGGGCCTAAAGTTCATATCCTCTCTTTTGGTGCATCTGAAATTCTGTGTAACCATTATGCTTGAGTGTCAGTATTCAGTCAGTAACTTCTCTTGGTTGATTTTTCTTATTTATCAAGGATTGCCGATGCTGAAGAGAATTTGGGTGAGAGTGAAGTGCGTGAGGCCCATCTCGCCAAATCCTTATATTTCATAAGGGTTGGGGAGAAGGTATATATCTTATGTGTTCTACATTTCTTAAGATTGTTAGTATGTGCTCCAGACATGCTTACCTTGCCCAAATAATATAAATTTCAGGAGAAAGCACTGGAGCAGCTTAAAGTTACTGAAGGAAAAACTGTAGCTGTTGGGCAAAAGATGGACCTTGTTTTCTACACACTACAAATTGGACTTTTCTATATGGACTTTGATCTCATCTCAAAGTCCATTGACAAAGCGAAAAAGTAAATCCAAATTCTTTTGGTTTATCTCTTTTTCAAATTGGATTTGCATTGTGAAGCTGATACAGTCTTCTTCTGGTTTGAAGCTTGTTCGAGGAAGGTGGTGACTGGGAGAGGAAGAACAGATTGAAAGTATACGAAGGATTGTACTGCATGGCAACTAGAAACTTCAAGAAAGCTGCTAGTTTATTCTTGGATTCAATTTCAACTTTCACAACGTATGAGCTGTTCACATATGATACATTCATCTTCTACACAGTCCTCACGAGTGTTATCTCTCTGGATCGTGTATCTCTAAAACAAAAGGTACTCAGCTTGTCTTCTTCAAGTTCTCTTCATTGCCCATTGCCTTGGATTGGATGTATAAACAACTAGGGTCTTGGCAGGTTGTAGATGCACCTGAGATCTTAGCTGTAATTGGCAAAGTACCTCACCTCTCGGAGTTTCTCAATTCCCTCTACAATTGCCAGTACAAGTCATTTTTTATTGCATTCTGTAAGTAACAGAATCATTAAATTTTCCATCTTTCATTTACTTATGATTAAAAAAATGCTCATGGATTATTATGGCGGGTCTAATCTTTGGGTTTCTCATCACTGGGTCATTTTTTTTTCAGCTGGCTTGACTGAACCGATCAAGTTAGACCGTTACTTGCAGCCTCATTTTCGCTACTACATGCGTGAAGTGCGCACTGTTGTCTACTCACAATTTCTGGAATCATACAAGAGTGTGACAATGGAAGCCATGGCTGCTGCATTTGGTGTCACAGTTGACTTTATAGACCAGTAAGTGCAGTGTCAGCCATGCTTATATCCAAGTTTTAAATGTTACTCACAAGCTTAACTTTTATTACCTATTTCTGTCACTCCAATACAGGGAATTGTCACGCTTCATTGCTGCTGGGAAGCTCCACTGCAAGATAGACAAAGTTGCTGGTGTCTTGGAAACGAACCGACCTGATGCCAGGAATGCCTTCTACCAGGCAACCATCAAGCAAGGAGACTTCCTGCTGAACCGCATCCAGAAGCTATCACGAGTTATTGACCTGTAGGCGAGAGCCCTTCATGCAGGAGCTCCCGGTGCATGACTTATTAGGAATTATTTGCCTTGGCTCCTGGAGTGTACAAGCAGTGCTCGACTTTCAATCGGTCTCAATTGAAGAAGCTTTTAGGGATATTTGTGTTCTGATAAATTGTGTTTTGTACTTTTGTAGCCTAACTACATGGACCACAAAAAAACACGGGCATTGCTTGTAGACGTTTCTTCATTGATGAATTCATCCCTTGTTTTTTCTGGCTAACTTATTGTTTTGCTACTAAGAGCCTTTGCTAGCAGAAGACGTGACTTTTTTTTAAACCTCAGAAGACGTGACTTATGTTACATCAAGCAACGATCCTCTAGAACGATGCGGTTCTGTTTCAGCCGAATAGAATGCTGTTCTGTTTCAGCCGAAGGAACAGAAAAGCAGATCGTGTGCTCCGTACTCCGGAGTAGGGATGAAAACTGACGGAAAAATTCTATTTCTACTTCCGTTTCTATATTTTTTTTGGCGAAAACGAGAGCGGGTTCAGAAAATGTGGATTCGAAAACGGAATCGGAATATGCGGACATACGGAAACGAACAAATACGGATGGAGAGCCGGAAAATTAAATTAAGATCACATAATATAATATTCTCAAAGTATACATATATAAAGTCATGTAATAATATATACAAAGTTTAATATTCATTTACATGCTATATACATGAACATAACATGCATATTTGGTGACTCGTAGTTATATTAACATAAGGACGTGTAATTGTTGAACATGTTGAGCATAGATTGAAAACAAGATAAAAACAAGATAAATACGAGTCTATTCCATATTAAAATGGGATTACTCGAAAACGAACGGAAAACACCATTTTCTACTTCCGCAAAATACGAAAATGGGATCTCGCAAATACGAAAACGGACAGAAAAATATAGAAAACGGAACGGGACGGTACGGGAAAAATTCCGTCCGTTTTCATCTCTACTCCGGAGTATAGGAATAGACGAATAGTACAAAGGAATCAAAAGTGGACGGATAGCCATGTCCCAATTCCCAGCATCTTTGCATACAAAACCCCACAAACAAGAACACAACGCTCACTGTCATTTCGGTATATAAACCACCCAAACTGCGTATCTACAATCAAAATCGACGTTGTTTATTGGACTATATGGTAATTCTGATTAATGTTACTGCACGTGCCAAATCTCTCAAATTCCTCTGTATGTGTACATTTTGAACATACAGTTCTGACAAAACCAAACACATCCTACCAGGGGCAAACCAACGCAAAAAACATTCCCAAAGCTAAAATCACAACCCAAGTTCCAGGAGAACTCGCACAAGAGTCAAAGAGTTACACAGCTGCGGCTGATATGGTGGGCTATTCTAGCCAGCAGCTTGACTGGGTTTGCCATTGGGTGCACCGTGCACCTAAGGGATTATTTTCGCCAGGTTTTGAGGCAGCCATTTCCTCCAAGCGCTTCCATAGGGCTTCTCGCTTCGAATCAGATTCCTCCTGCTTGAGCTCCTCATCATTGAACCTCTGAACGCACTCCCCGAAAAATGAAGAATCATGATCAGAGAAGATCTTGCGTACGTTCAGTGTCAGGCTACGGACAGCTTGGTTCCAGTGATCTCTGGAGTTTCTCTCGAGTGCTGGATAGATGATAGGTAATAGCACCTTGTAGTTTTGCTTAATTAGGTTTTCAATATGGTCATTGTTCCAGAGAAACAGAGCCCGCTCTGCCACCTATGAACAGAAAAGGAGAAAAACATAAGTCATAAATACTGAGTATACTAAAAAAAACTGGATAAACCTAATGCTCAGCATTTCACCTGGGCAGCCAGCTAAAGCAGTGTTCATATTCTATTATCGATAGGCTACAAACTGCTCAAGTGGACATATATGACTAAACAAAACCACCAGATACCATCAGCGATGAAAAAACTACATTCCATTTTACAAAATCAGCAGGATCCAAGCAGCACAAATACACAGTTATTAACTAGAATGATTAGCTAACAAGAGGGTAGCAAATAATCATGCTTCGAATATGTAAGGTCATGTCTAAAACCTGTTCTAAGACGACCAGAAAAGGAAAGACAGTCAGGAATACATGAAGAGAATTCTACACATAGCAAGGACAGGAGCGATAGTCAAAAAACACAGGTTTGAGAGTCATAGATGAAGAGGTGTCACAGATAGGCAGAGGTACAAGCAAATGCAAGAATATACAGACTTTAACGGACAGAAATTGATAAACAGGTTACATTCCAATGGAGCAGTTCACTAATAAAACTAAAAGAATGGGGTTTGCACCACAATTCATAATATGGGAAATTATAATGGCTTTTGCTTGCTATCCAATTTGGGCACCATGAATACCACTTTTTGATACATGACATTCCTGATGACAACTTCTACTGTGACTTTTGTTTCAGTAGATACAAAACACAAATTTTGTACGCCACTAATCCAATTACTGAGAAAGAAGGCATATAAAGGTATAAAACCTCTTAAGGTCTATATGCAGCAGATCAGTTTCTAAGTATTTAAGGCAGGATAAAGTTTTTTTAAGGCCAAGGAATGAATTAAATGTAATTACAACATAAAGGTGAAATGGCTTAGAACCAATGATTTATTTACTATTCTTATCACAGATAGCATATATATGAAACAAGTGAAAATACATGTACATAAATTATACACTAAAAGATACCCAGTAGTCCAGAGACAAGGTATCTTAACAGAAAAACAGGAGACACTATGAGCATGTACATAAATTATACATAAAAAGACACCCAATGGTCCACATAGAAGGTATCTTAACAGCAAAGCAGGAGAGACCATGAGCATTGATCTTTTCCACACTTAGTAGTAGATTTCTAGCAAGGTAAATCTGTGCAAGCAATCAATGTGGACATGTAGGCAAATAACTGGCAAGGATTTGCACTAAAAGTCTTTTTCTTTTCCTTTTGTGTGTGTGGGTGGGTGGTTGGTGTGCGTGTGTGTGGGGGGGTGATATTTGTTTTGCTACATGACATTCCTGATGACAGCTTCTACAGAAATGTTATACAGAAGTATGTTAAACCAAAAAAAGTTTGTTAAATCCCCCAATGGCCCAATCTGATTGCTACAAAAGAGGGACATAAAGATTTAGGTACCATGCAACAGATCAATTTCGAGATTACTTTAGTCTTTAAGGTACAAGAAAAAGAAAAGGAAAGGAAAAAACAAAAATGTACGTGTTATAGATGTTTATAAGGGTCTGATTGGTTAGCTTCTAAA

>BAC-34

TAATTATGGTAATTTTGTGTCTCAGATAAAATATGTCAGTGGTTCCTATGACACTGGAGAAGGATTTGAGAAACTGAACAAGGCAATATCAGAGTATGAGGCATCAAACAAATCAGGAAGCTATCGCAGGCTCTTTTATTTGGCATTGCCTCCATCTGTCTACCCTTCAGTGTGCAAAATGATCAGAACATATTGCATGAATCCATGTAAAAGTTTCTTTCCTATGCTGAGTTTTTTTGTTCTGATAAGCTAATGCAAATCCTTAGTATGAACAAACAAGTATGGTGGAGTTAGTGGAAGCTTAAGTTTTATTAAGGTTCTATTATCTTTGCTTTTCTTGAAGCGAAATAAACTTTTACATTGTTATTCAAAACTTCCATTTGTTCAGTAGTAACGAAAGAGCATGTTTATTTTTAAGCTTGTGTTCCTACCTTCTGACTACATTCAAAATATGAAACTGATTTGGAAAAGATTGGTGCAAAGGACATTCATGCTGTTCATCAAATATTCTTTAATAGTTAGTTCTTCACAAAGGTAGAGGAAATAAACCAATACCAGTTCTCTTTTTAATATATATAGAATTATAGATTTAAAAATTCGAGCACCATTAGTACTGTCAAGTCTGGGCTTCACATTAGTGTTATGCTTATGCGTGCAGCTTCTCAACCTGGATGGACCAGAGTCATTGTTGAGAAGCCCTTTGGAAAGGACTTGGATTCCGCTGAAGAATTAAGTGCCCAACTTGGGGAGCTATTCGAAGAACACCAACTATTCAGAATAGACCATTACCTGGGAAAAGAGTTGGTCCAAAACTTGGTAATGATGTAACTTTTTTCTCAACTTGCTTTACATTTCACAAATACTTGTCTGACTATTTTTTAACCAATATCGCGTTACAGCTTGTCCTTCGTTTTGCCAACCGCTTGTTCTTGCCTCTTTGGAACCGCGACAATATTGATAATATACAGGTAAGATGTTTTGTATTCGGTAGCTAGCAGTTGGCAACTTTATTTTGCTGGTATAAGCTCCATGCACATAAATTGCAGTCCCTTTATCATTCAAGGTACTCTGTAGTTCTGTTCATAAGTTATTTGACTTGATCCGTTTGTGATTATAGTAGGGAGCTGTTGTATTCTACTGTAACAATTTACTACAGTTTGAAATGAGCCAAACTGAACCTAAGATAACTGAAAGCATTACAGTATCAATGACATGGCATGCTAATTACTCAAACACTGCAGATTGTATTCAGGGAGGACTTTGGAACTGAAGGGCGTGGAGGATATTTCGACCAATATGGGTATGTACAGGCATTTCTTTTCCTCGCAAAAGTAGAAGCACTAGTGTACCATATTTCAGTAACATTTTTATGAAATTTTTCTAGTCTAAAATGATACCCTTACAAGCTAAAGTATAAATCTTCTGCGTAATGTATATATAAACCATGGTATTGATGATAAATTTAGAAATACTTGAATTCCTGGTTGGTTCTTAACAATGAATAGATGTATTTTGCATTTTTTGATCTGAATTGCCAAAAAAAAAAAGATCATATCACTCTCTCGTGCAATGGTTGAACAGATGAGTTGATGGTAATCAGCACCAAATGATTATGCCTATTTCTGTCACATTGATTAAACATTTTTGTGTTTCTTTTTATACCTTCTTTTTGTTTCTTTCCCTTTGCTCTATAAATATCTGACTCTGCTCCTGTTGTTGCAGAATCATTCGTGATATCATTCAGAATCATTTACTGCAGGTAAAATATAAATAAGATATATTTCCATATGCTATGATTCTGTGTTCTGCAATCATTTTCATAATCGAGTCCAGATTTTAGAGGTTTATTAGTATTTTAGTGACTATGGACAATAAAGGCATCTTTTTCCCTACCTTCTTCCCACTATTTATATTTATGGATACATGCTAAATCTTGTGGATTAACTAGCCATGACAGAATTTCCTGAAGAGTAGTAGTGAGTGAATGCCTTTGTGCATTAACAATCTTAGTCATAAATTCTTACAATCTGCAGGTTTTCTGTTTGGTTGCGATGGAAAAGCCTGTCTCCCTTAAGCCTGAGCACATCAGAGATGAGAAAGTCAAGGTCCAACTCTCTTTACTCTATTGATGTTACTACTATATATTGCAGCTTCGCCAATTTTCTTGCTGCGGTTTGATTTGCTCTCTGAAATGTGGCAGAGTGCACATCTTATGTTTTTTTTTTGTTTCTTTAGTGCTCAGAGGAAGTTCCGTGTCTTAATCTCAGGTTCTGCAATCTGTGAACCCTATTAAGCCTGAAGAGGTAGTCCTCGGGCAATACGATGGCTACAAGGATGACCCTACAGTGCCCGATGACTCAAATACCCCAACTTTTGCATCTGTTGTTCTTCGGGTACACAATGAAAGATGGGAAGGTATATCTTTCCTCCACATGACAGTATCTGTACATATTACAGTTGAAATGGATATACTTGAAATACTTGCTCTCTGTGTTTTTTTTTTCATTCAGCGTGCCTTTTCTCAACCATAATATCTTTACTTTCTTTATCAACCCTCTATTCATGCCGTGCAGGTGTTCCTTTCATTCTTAAAGCTGGTAAAGCATTGAACTCAAGGAAAGCAGAAGTTCGGGTGCAATTCAAGGATGTTCCTGGTGACATTTTTAGAAGTATGTGCATAGAAGTTAATATTAATGGTGTAGTTTACTCTTAGCATAGATTTACCTGTGTAGGAGGGGAGTGCCTTTACATAACCAGACAGTACAAAAGCAAGATGTTCTTTCATAAGTTAGAGATCATGGGATCTGAAACTACCAAAGGATTGGCTAAACGTTCATTGACAATGCAGAATGGGGTAGGCCAGGTCCATGCATTGATACTGGAAAGCCATGACAGTGGTCCCTTTAGATTGAATGAAATGCAATCGTGCAGAGCACGTGCATTGCCGTTTAACTTCTCACTGAATTTTGTCTACATTAGCTACCATATTGGTAAAATTAGTAAATTGTTAAATTAACTCAAGCTGGACTAGACTAAATGTGTTTCTCACTGAAGTTCAGCATGCAATTGTTAGCCGTAGGGTGGATGGATGAGACTCTTTGTTCATTGAAAACCAAAACAATACTAAGGATGACGATTTTCCTCACCTTATAGCTTCATGGAATGCCTCTCTTATTGATGACCTGTTGCAACGAATATTTTATATTGGTTCATTATGTTGTTCTGTTATGTCTATGTAAGAATTAGAGATCTTGGTAGTGGAGCTAAATATAAATATAAATATAACCTGGTGAAGGTAAGAAGCAAAGGAGAAATGAGTTTGTTATACGCCTCCAACCATCAGAAGCCATGTACATGAAACTAACTGTAAGTCCTGCTACTTCTCTAGGGTTTACTTTCTTCTTTTCATCAGTAAAACGTCATGAAATTTCTTTGGAAACTGGACGTCGATATCAGATTTGAATGTGTAGCAGAATCTCATATTAATCTTTGAAAATTAAATTGCTGCAGGTTAAGAAGCCTGGGTTGGAAATGGCTACTGAACAAAGTGAACTTGATCTGTCATATGGGATGCGGTACCAAAATATCAAAATTCCTGAGGCATATGAACGCCTTATCTTGGATACGTAAGTGTCATCCTTTTCATCATTCTAACATATCATTCATTACAAAGAAATTCTCTCATCCTGAAATCGTGGATAACTCTGTATATATCATGTCTTGTGCTGATAACATTGCAAAACTTCTTCTATGCAAAATTTTGCTTTTGTTTACTGCACCTGCACTGATCCATATCCATGTTTCTACAGAATAAGAGGAGACCAGCAGCACTTCGTCCGCAGAGATGAGCTAAAGGTCTGTGCCACCCTGCATATTTGTGCCCTCTTGTTGGATCCTTCATCAGCAATAACTCTTTTGTTTCTGTTTTGCTAACACCAGGATCCTTTATCTGCAAGAACTCTTTCATTTCTGTTTTGCTCCATCCTCTAACGCTGCACCTTTCACTTTTTCAGGCTGCTTGGCAGATTTTCACTCCTTTGCTGCACGACATTGACGACGGCAAGCTGAAGGCCCTTCGATATGAACCTGGCAGCCGAGGCCCCAAGGAAGCCGACGAACTGAGCGCGAGAGTTGGATATGTGCAGACCCACGGTTACGTATGGGTACCGCCGACCCTTGCATAGAAGAGAATGGCCGGTTCCATGGCCTTATAGGAATTGTGTGTATCTACTATCTAGGATGAGTGCCCTACAATAAAGCTGCGCATATATATTGCCCATTTTCGCTATATCGCACGGCGTTGTAATAATGTTCTGCTGAATATCCCAACTACGATATGAAATAAAAATGCATGTTCCATTCAGTGTGCCATGACCAGCGACGAAATGCTGTTTGATCCTGCAATAGCAATACCGAGGAGTTACAAAGAAAAAAAAACATGTTCTGGCAGACTTTAAGCAGATACTCTACTATCTCTGCTCTAAATTGATTAAACTTTAGCTTTGTTCTAATCACTTTTTTTTCAAATTTTGATCAGGTTTTTAGAATGATACATTAACATCTGCCAGGTTCAAATAAAATATACAGATTTTAACAAAACTAGTTTGATATATTTTTATGTCCCTTTACGTGTCTCCCTATTTATCGTCTCTTTTGGGTTTCATCTCTAGCCTACCCTAACTTGCTTGGGACAAAAGAGTATGTTGTTGTTGTTGTTGTTAAAAAAGTTCGATAACAAATTATCGACAGTGCTAGCATCCGGACGTCTGGTCCAGACGCCTGCGCTTGCACTCTGTTTCGCGCGCCTCACGCGGGCAAGTGCCGCCCTGAACGTCACTGGCATCAAGAAGAGAAGGGAGGCAGCGCCCTCCTGCGCCTCTGGTTCGGATGTCCAGCCGGTGCCGGGAGGAGGAGACACCAAGGCGAGACAGCAGAAGGCGAGGAGGGAGATGCAACACTCAATCTACTTTTGAAACATTCAGATGCAACGCTTATAACATACGTTTGAAGATAGATGAAACACTTGAAACATACATCTGAGACGCTTATAAAAACACCTGAAAAAACTTGAAATCATTGTAAAACATACGTAACATTCAGATAAAATACTTACAACATATGTGTGAAACATATGCAACATCGAAATAAATACACTTGTAACATACGTATGAAAAAAACAGATAAAACATTATGAATAAAAGGTTGCAACATATGCAACATCTCGATCTACTTTTGTAATATCCATATGCAACACTTCGCAACTATACCTATGAAACACTTGAAACATAGGCTTGCAACATGCTCCTTCAGCGCAACATCTTCATGCTTCTTGGCTTCTTGCGAATGGAGGCTCGCCGGCGCGTGGAGTTCACTGATATAGAGCTTGCTGGTGGCTCGGACCTTGCCGCTTCGGTGGAAAAGGCCACAACAGGTCTGGTAGAGAAGACCGCGGCAAGCCCACGCACTGCAGAAGTCCGCGGCGGGCAGGAGGTGCAGTGGAGAGGGAGGAAGACCGGCCACCGCGCTTGGGTGCGATGGAGATGCCGGTCGACAGAGCGGAGGCATGATGAAGAGGGGGCGCGATGGAGAGCGCGGTCGCCGAACACTGCTGCGGCGCTGCGGTGAGTTGGGTTATCTTTAAAACAGTCGTGTGATGAGTGGACAAGTATATAAGAGATGGTCGTTTAGCTGGCCTCGCGGCCGAGCATGCCCGCTTCCCAGAAGTACTGATAAATTACTGTGAGGGCTTGTTTGGATGCATGTATATCCACCTCAATCCACATGTGTTGGAGTTCAATTCCACTCCCATCACCTCAATATTTGTGGGTTCAGATGAATATATACGCATCCAACGAGGCCTGAAAAGGGTATTTAGTTAGGTTAAGATCATGGTATATTGTACATTTGGGTTTTAGAAGCATCTAGCCATCAAATTTACTCTATTCGTTTAAATTTATAAGTCGTACCGTTTCAACGAAATAACGTCAGCCGTTTTTTCCACCCAACCGACGAGGACGGTCCTCCGTGGCTGTCAGGGAAAGGACTAGGGGACTTTTTGCGGGGGACAGGGGTCAGAACGAAACGAAACCCAAATATCTGGACGCAGACACTGGGCCGCGCTGCTTCTCCGTCCCACTGAATCGTCGCCGCCGGCTAGCCGCAGGAGCTCGCCGCCGCACCGAGGCCGAAACCGCCGTCGCGTGTTTGTAAAGCTCTCGAAAAAGGGGCGCGCAGCATGAGAGCGGCCGTGAACACACCACCTCCGCACTGCTACTCGCGCCGCCGCGCCAGCGCCACGCTGCTGGATGCGCGCCATGTGTTCGACCACGCGCCGCAGCGGCGCCTGCCAACCCTGGCCGCGGAACTCCGCGCGCGGCCCGTGGCCGCGGCCGCCCGGAGGCCCGCGCGGCGCCCGGCCGTGTCTGCGGCTGCCCCCACCTGTGGCGCTGCTTCAGGTACTGAAATTGGGTCCTCTTCAGATAAGCTTGCTGGATGCACTTACCTTTTGGAGCCGCCTCATTTGTGCACTGGAGTATCATTTGGTATTTGGCCAGTGGTAGCATCATTGGATTGTTTGCGTGTGTTGTTCAATGGCCCTTTAGCTTTGATGAGATATTGGGGGTTTAGGACAGTAGTATCTTTTTGTGGCACAAAAGGACAGTGGATGTACAAGAATGGGAGCAAGCTTCAGTCTAGCTCGCAACTAAAAGATTAATAAATGCTACTTATTGTTACTTAACCAGGCTACCCCAGGAGTCCAGAGTGTGTTGAATCTTCCTTATCAGTTGAGTTGGGTTGCTCGATGTTTTTGGGTTTGTGCGGCACACCCCTCTATGTTGGGTTTTCGTGTCCTGTTTTCTTAAAAAAAAAACTGGGCCAGCACATGTTTTCGTTCTCTTTCAAAGAAAACTTACGTCAAAGCTTTTCCTTTTGAAAAAATATTTGGTTTGTTTGAAATGGTAAGAATTGAAGAGACAGGGAGAAATAAGAGAGAATAGGATTATCAAATTTCGGTGTTCAGAAGTTTGCCCTCCGTGTAATGTAGGTTTGTTTGCTCACAGGTGCACCAAATGAATTCAACTCATCTATTTCGTCATGTTTTATTGTTAGCCAGTTGACAGTCTTGCTTGGTTCCCTTAAACCTTAACCCCCTGCAACTAACTGTTCTTGCTGTTTTGGTAATCTGTACCTGCCATCAATCCTGGAATTGGACTTATTAACACTTAGCCTTTATTGAGTCATGTGATTTAGCTTTATAGGATTAACTATGAACCTTTGATTTAAGGAGAGCGGGGAATTAAATTTATGGCAACTTTTGGTTTCATTGTCATCTGGGTTGCATCAAAATAGATGGAACAAAATAGCATATATGATGGTTGACCTCAAAGTGAAACTTGACTGACTTGCCTGATAAATGCAACACGCTTTGAACTTGGAAAATGCTTAGGTACATGTACAGAAAAAATGAGGCGGGAAAAGTAGTTTTCTTTGTTTTTTATTGTTCTTATCTGTTTGGAACATCAATACATGATATACATCATAGCATTTTGCATGCTTGTTGGGAGATTGTATCAATTTGCACGCTGGTTGGGAGGTTGTAGAGGTTTGCACGGTGTCGTTGTTCACTTGCTAGAGTTTGCTTCTTTGTAGTGAACATCATTGCTTGAGAGGCTGGGATCTCTTTGGCTAGCTCTCTCTAATATTTACTTGTCCCAAGCAATGTCTTCTTGCTGAAATTGGGTAAGGTATAAACCCTTGCTTGCAATATTCTTCCCCTGCTTCATTCAAGAAGTGAAGTAAGTTCTTCATATAAATGAAGCATAGTATTGTCATGTATTGCGCATTCTGAGGTTAGCCATTCTTGTTGGGTACTACCTACATGCTTTCCAAGATTAACTTCTCAGCTTTATTAAGATATGTCATACTTATCCAAATTACAACATCATTGCCAAGCTTTCTAACCATTGAGCAGTTCCTCGTTTGTTTAGGACATTCCTGCAAATTCCAGTGGCCTCTTCTTCAACTTCTCGCGTTCGTTTAGCTCCGTCTGCATTCTTGCCATCAAGACGGAACTTTGAAGGCTATATTCCTCGTAGCTGCTCCAGTTCATCGCTCAAGATCTATAGCCGGTCATCTCTGTTAAATCTATCACCATCTTCAGCTCTCATGGTGTCCTCCCAGCTCACCTCCTCTGACGTAACCCAACGTTCAGAGGAATGGTTTGCTCTTCGCAAGGACAAGCTCACCACGAGCACCTTCAGTACTGCTTTGGGTTTCTGGGCTGGCAATAGAAGAGCAGAGCTGTGGAATGAGAAAGTCTTTGGAGCAACAGAGATCAAACTGGCGGATACTGCTAGGTCTGCCATGGACTGGGGGACGCATCATGAAAGCGTAGCCATAGAGCAGTACACAAGCATCACAGGAAGACTGGTGGGCACCCTTGGCTTCGCGGTGCACACTGAGGCCAACTCCGGATGGCTCGGAGCTTCGCCTGACGGAGTCCTTGGGTGCGAACCGGACGGCGGGATCCTGGAAGTCAAGTGTCCATTCAACAAGGGCAAGCCCGAGCTTGCTCTGCCTTGGCGCGCCATGCCGTACTACTACATGCCACAGGTGCAGGGCCTGATGGAAATCATGGGCAGGGACTGGGTGGAGCTCTACTGCTGGACACCCAACGGGAGCAGCCTGTTCCGGGTGCCGCGGGACCGCGCATACTGGGAGCTCATCCACGAGGTGCTGCGCGACTTCTGGTGGGGTAACGTGATGCCCGCGCGGGAGCTGGCGCTCCTGGGGAAGGAAGACGAGGCGAGATCCTTCGAGCCTCAGCCCAAGCACCGGCTCACGAACCTGGTGCTGTATAGGAGCAGGAAACTGGCCTCCGAGGCCAAGCTATTGTGTATTGATGTTGGCGGCCATGTAGAATTCTTCAAATGAGAAGTTCTGCCGGTGATTGTACACTATGTTATGACTATTCTTTAATTTAACTCATGAATTCGTTACCTTCCAAGCGTCCTGTTCCTTTCTTTTTTGTTATGTTTAATAAAGAGTATATTCTTCCAATCACATGTCTTTTTTTGACAACGACCACTCACATGTCTTGTGATCGTTTTGCGCGTTCATCTGATTTGATGTATTTGCATTCGCTGAAACTATGGAATCTGGAAAGGTTTCAGATTAGAATTAGAAGGTTAGCTCAGAAAATGGATTTGGTGGATCGGCCAAATACAAGTCCAAATGTAATTATTTGTGGTTCTCCTTCTAGCATATGGACACCAAGCTGTGTCGTGGCACCAGGGATTTCCTGCCTTGTGCCCATTCTAAATTTCAATGCTTTTGGATTGGCCTTTGCTTTCCCTTGTACGCCAAGGAGAAGTGACCGAATCAGACAGCGATTGCCTGCTTCCCGGATATTCTCATTCCCTCCGTCCTGGAAGTCCAAAACGGCAAAAACAGACTGCTCCTCTCATCCATCCACCTGCCTGTTGCTTCCGACTTCCGAGCTCTCAGGAATCCTTGTCTCGCCTTGCCTGTGGGACAAGCAAGCCTGTCTGCAGGACTGCAAATCAGCCACGCCTCATGTGGCCGGCACCTGGAGGCAGGACAGGGCCTAACATAACCCGGTGCCCCGGTCAAGAACATAATACGCAGGAGGCGGACCTGCTCAAAACTCCAAGCCAAGGCCGGTAAACCATGCGCTTTGTAAGAACGACCAGCACTAGTTTGGTTCAGCATGTTTGCAGTTCTATAATTAGAACAGCAGGGAGCACTGCCCTCCTATCAGTTTTGTGGATCTTAAGCCATGTGGTCACGAACGACCAGCAGTCCAGAAAGTTCGTTGGTTCAGCAGATTTGTGGTGTCTGTCTATAATTAGAACAGCAGTGCTCACGCTGCTGATCCACAATCCATGACCAGAAAGGCACCCATTCTATTCCAGATGTCCTGCATATATTGCGTTGTTTAAGCGACCATGCGTCTTCTGTCCAGAAAGCGACCATGCGTCTTTTCTGTCCAGAAGAACGGGACCAGACACTTGGTTCTGGGAGGCAGCACTCAGTAGCAGGAAAACATGACCGCAGAGAGCAAAAAACTATAAGGGGGGTGTAGTCTCTCGCATGCTTTGTTTCAGCTTTCATCAGCACTAGCAAAGGATGGCCATTTATTTCCCTCACCAGCACAAAAAAGACAGACACATGTCCCATACTTGCCAGAACGGGATGACAGATAATAGCCACCAGAAAGAAAAAAAAATGAAATACACCTGGTTAAAACAGGCACCTGAGCATCTGCAAGCGCGAAAGAATCTAGCTAAGTTAGATGTTTTGTCTTTATTTATTCTAATAAATTAGGTGGTACAAAGTTTCTACAACCCAGACAGTAAATATTACATTTGTCAATTGTCAAGTAACAGCACGCAGCACACAGGTAGATTGGTATCATTGGACAAATAGATACGTCCGATTTTATTTTGGGGCCAATGAAATCAATTACTTCTGATGTTCCAGGAACATGCAGCATCAAGCTACCGGCTGGAAAAAAAAACTATACTGTTGTACTTCAACACTCCTGAGCAGAGCACTTCATTATTCCAAAAGGAAATATCCCTGAAACAACAGTACAAGCAAAGACTCAGCAAAAAACTGAGGCCCGATTTGGAGGGCATTAACTGAGCTAGTTTCATGCATTAATTGAGCTAGTACAGAATTTCTGCAAGGACCAAAAGAATATACTAGAAGCATAACATCACCTACCGCAATTGTCAGTTGACACCATTCACTGCTGACACCTTGATAGGGAAACCATACTTTCGTCTTCTGGTTTTCTGGGTTATACTGTTTTTTTTTTTTTTTGCTTTTTCTTGCAAGCACGCTGCTGCACCTACACCGATAGCAAACTGGTATACCTTTTTCTTTTTTTTCTTTTCCTTGTTAGAAATACTCAGTCAAGATGACTGATGGATGGGAGCATCAAACAGAAGAAATGCTATGTTAAAACTGTATCCATCCAATGGATATAACAGAATCGAACATCTATTTTTCTGTGGGGGGTGAATCTTCATACTTGACACAGTCACGGCAAAGGTTCTCCAGAAGAAATGTCTCCTGAAACTCACTGTCTATACATCTCCCACATCGCAAACATCTCGAGATGCAAACAACACACCCTCTGCAAGTACTAGGTCCTCTGGTCTTGCATTCCTTTGATGGGCAATCATAAACAAGCTTGTAGTTCGGACAAAGAGGACACTTCTCAATATCAAGAGCATATTCATCATGAAGATCTGGTACGAAAGAATCGGGAACATATCCACCATGAAGATCTGGTAAAAGACGATCAGCATGAAAGATCCGAGGCTCCTGTTTGTGTAATGCCAGCTCCTTGTCTATTTTCATCAAGGACAACAACTCTTCATATTGCGCTTCTGATGCAGTGACACGATTTGTAACTCTGAGTCTCTTGATACCAATATCTGCTTTCATATTAAATGACCTCAGATTGTCAATAAGACCTTGATGAGTAATCCGGAAAGCACCAAAAATGCCCAACTGTATCAAAAAATAAATAAAGAGACAAGCAGCAATTAAGTAAATGGAGAATTGTGGATTGCCAAAACTGGTAGCACAAGTTTTTCATACCAGTTCAACTGCTATGCAACAAAACTGAAATACTGTAAAGCAAATAAAAATTCATGTGAAAAAAAATCCTAAGCTTTAGTTGAAAAATAACTAACCCTTCTTTGAATTCTGCTGTAAAAACATGCAATATTATCAAGGTAAGTGCAAGTAAGTAGTCCTATCTAACTAGTTTGCATTCAAGTTTGTGGTCTGGCAAATATTATATAGACAAATGAATGAAATTAGCTAAATGAAAAAATATGTAAAATCTAAGTTTCCAGTTCATCTCATAGATCCAAATACAGGGTAGCCAAGGTAGCAGCCTTGTTCCACAAACATATATTAGCACCCAATAACATTGGCTGGATGCTCAGAGC

>BAC-29

TTTTAATGATACGGCGACCACCGAGATCTACACAGAGTAGATCGTCGGCAGCGTCAGATGTGTATAAGAGACAGATTCACCCCGTATAAACTAAATTAAATTACTCCTATGTTGCCTCATATCCAGACAGTATTTATTAATATTAAATTAATTAATTAATTAATGTAATCCCATTCCGATTTGGTTGCCCATTGTTTTTGCCCGAAAGATACTGCTCCTCCATCACCGGCATCCCGCATTCTCGACAATTAAAACCCCCGCCCCCCACCTCCTCCCATTCCTCTTTTCTTTCCTCCTCAAATCCGCCCGCCATTGCTTTCGCCGACGCCGCTCGTCGCTCGCTCACTGGCGCTTCCTTCCTTCCACCAGCCATTTCGTCTCCGTCGGCCATGCTCTCCTCCTCCCCCGCGGCCGCCAGAACCGCTGTGAGCCCGCGAACCGACGTATAGCGCCGCCGTGCTGCACCGCGACGGCCCACCGGCGAGGAGCCGCCGACCTATGCCCCTCGACCAGTCCGGCATTGGCTGACCCCCAAATGCCTCCCGCCGCTAACTAGCCGCAGCCTGTACATACACAGACAGTCATACACACTGTGCCGGAGGTTGAAGCGAGAAAATCGATCCCGGACGCTTCGCCGCCGCCGTCTCGTCGCCGCCGGAGAGGAGTTTTAGCCTCAAAGGCGTGATTTTTTTGCCTTGTTATTTTGTAGTTTTTTTTTCTTTCGTGATTTTCGGATCGTGTTCTCGCGTCGTGCCGGAGACATGGGAGTTGTGGACTTCTCGGTCCTGGGCGCTCTGCAGAAGGTCAGATCCTTCGTCGCCGGCGCCGCGCCGGCTGAAGCCGCTGACGGCCGCCCGCCCGCCACGCCGAGGTCGCTCAGCGGGGGCCCCTCCCCAGAGAACTCGCCGCCGCCCGCGGCGACGAGATCCGGAGGCAGGCGCGCCATCGCCCTGCGCCGGCAGATCTCCTCGCCGCAGCTGCTCCACTGCCGTGCTGTCAGGTACTGTGTGCTACTCCTCCGTTTCCGTCGTACAGTACTTACGTTTCAAGTTTCCATTTTGACTTGCTATAGAGGAAAAGGGAAAGTGTTTTTTTTTTCAATCTGAAGTGAACGGATTGGTTAAGATTGCGTGGCGCCACCTGCTACGATCAAGCAATTATTAAGTTAGCTAAAAGTCTCTGCCTTTTTTATTTGAGGTAGAACTTGGTCTTGAATAAGACAGAATGAAAACTTGTGACTGGCAATTAAGTGGTTGCGGGAGCAAGTAATTCTCACGGAAGTTTGGGCTTTTCATCTTATTTTTACATTTAGTCAACTATTGAGCTGAGTTTTCCTTGAATTGGTTTGTATAAATGCAGGCGAGCAGATGATGAGGACGATGATGAGCCCGGTGTTCAGTTTTTCACCCCTGGGAATGACTTCATACATGACTTTTCAGATATAGATTCTGTTAGTGTTAGTACCCCTAATGAGATAAACCGGTCCCTGACCCCAAGCCCCTTGGAGAGCCCAACTTGGACGGTGAAGCAGAACGACAGCTCACCGATATCCAGGAAGAATGGCTGTTACAGCCCGGATTCTCCCGGATATGGTACAAAGGCGAGTTTAGGATCTGATGGTTCACTGCAACAGATGAATGGTAGCCTCACTGACAGTGGTGGAGAAGGAAGCAAAACTCAATATCCTGTTGATTTCAGTGCCAATATTTGGTCCCCACCACCACCAGAAGATGAGGGGGATGATATCGAATCAAGGTTATTTGGATTCGATGATGACGATGATGAGGTTGGGGATTCAAGCAGGCTTCTTGTTTCTGGTAGCTTTAGTGCTAACAAAATAGCTGGTATTGACGAAGTTACAAACACTGCTCAGAAAGAGGGTCTGAAGACTGCAGTGCTCAGTCATTTCCGAGCTCTTGTGGCTCAATTACTGAAGGCAGAAGGCATCGATATGGGAAACGATGATGGGTCCAAAAATTGGCTTGATATTGTGTCATCCTTAACTTGGCAAGCCGCAAGCTATGTGAGGCCAGATACCAAGAAAGGAGGCAGCATGGATCCTACTGATTATGTGAAGGTCAAATGTATAGCATCAGGGGATCCAAGAGATAGGTTTGTTTTTTTTTCAATAGTATTTTAGTTGACTAAAATTTACATGGATGTTCTCACCATCATGTGGAACTGATGAGTAATTTCTTTTGTACAGTAATTTTGTTCAAGGAGTTGTTTGCTCCAAGAATGTAAAACACAAACGCATGGTCTCTGAGCACAGGAATGCAAAATTGCTCATTTTAGGGGGCGCACTTGAGTACCACAAGGTTCCTAATAAACTAGCGTCTATTAACAGGATACTTGAACAGGTCTGTGCCTAGCATACTCTTGCTGTACATTTGTTTGTCCAATTTATGCTTTTACTGAAGAAATGCCCTTAATACAGGAGAAGGAGCACATGAAAATGATTGTTGGAAAGATCGAGTCCCGACGACCTAATGTTGTGCTAGTTGAGAAAAGTGTCTCATCTTCTGCTCAGGAGCTCTTCTCAAAAGATATTTCGTTAGTTCTGAATGTTAAGAGGACACTTTTGGACAGGATATCAAGGTGCACAGGGGCACAAATTGCATCAGTTGACAGTATTGCTTCAGCACGGCTAGGTCAATGTGAAGTGTTCAAGGTGCAAAAAGTTACAGAATTTCCATCAGCTAAACAGACAGATAGGAGATCAAGCAAGACACTGATGTTCTTTGAAGGCTGCCCTTGGCGTTTGGGTTGCACGGTAATAATCCCTTTGCTTTTCTATGAGCACCTGATTGGAATGAACAACTCATGTCATCAGCTAACATTAAATACTGTAGATGTTCAATCTGTTATGAAGAGTATTAGCACTATATTTAGCTGATATCCAATTTCTAGTTTTAGTTTTCCAACTCAATTATAACTGTGTCAATTTATGTAGGTTCTATTGAGAGGGTCATGTCGGGAGGAGCTTAAGAGGATTAAGCGTGCTGTACAACTTGCAGTCTTTGCTGCTTATCACCTTTCTCTTGAAACATCATTCTTTGCAGATGAAGGTGCAACACTTCCTAAATTTCCCTTGAGACATGTGGTAGTTGAGCCAGGTATAAGAAACTGCACAAATAACAATTCTGCTGCATGGGCCACTGTTGGTATGCCTCCTCATGGACGTACATCAGAACAGGACAAACTCTCACAGACTGCTATGGTCAACATGATGTTTGAATATACGTCTGTATCACCTAGTTCATTACCATTGAATGAGGAAGGCCATGGGTTTGTGGGTGCATGTGAGCATAAAGAAACTGAATATTCTGTTGATCATAAGAACCCTTGTGAACATTGTGTATCCTGTGCAACTGGTTCATGCAATGGGCATGAAACATCTTTATGTTCACTGGATCATGACTCAAGGATGCAGAATCAGAATTTGCAGAACTCTGCAAAGCTTACATCAAATGCTCACCAAGATGAGCCTCTGGCAAAGAAATGTCAACAGGTAGATCACTGGAATAAAAAATCACATTATGATCACTCAGCAGACCAGCATGACCTGAATGAATTTTCTGGTGAATACTTTCCTGGTACTGACAATCATCAGAGCATCTTAGTTTCGCTGTCCAGTACTTGTATCCCAAAAGGCTTGGTATGCGAGCGTTCCCATCTGTTCCGCATCAAGTTTTATGGTAGCTTTGATAAGCCACTTGGGAGATACCTCCGGGAAGACTTATTTGATCAGGTACATCCCATTTTCATCTTGAGTATGTACTGTCATGTTGGACCTTCTCATAATAATTTTCTAAACCTAATGGTTAACACTTTTCGTATATTCATCAGGCATATTGCTGTCAGTCATGCAAAGAGCCATCAGAATCACATATCAGGTGTTATACTCACCAGCATGGTAGCTTAACAATTAGTGTTCACAGACTTCGATCTCGAAAGTTGCCTGGTGAGCGTGATGGAAGGATATGGATGTGGCACAGGTGCCTCAAGTGCAGACCTAAGGATGGTGTTCCACCTGCCACACGAAGGATAATCATGTCTGATGCTGCCTGGGGTCTGTCATTTGGGAAATTCCTAGAGCTAAGTTTCTCAAACCATGCCACTGCCAACCGAGTTGCAAGCTGTGGGCATTCACTCCAGAGGGACTGCCTTCGTTTTTATGGGTATGTGTCTGCTATATTCTTTGCAATATCATATTGTTCCGTGCTCCAGTGAGATTTCAACAGTTCAAATAAAATTCCAGGTATGGAAACATGGTAGCATTCTTCAGATATTCTCCTGTTGACATCCTCTCAGTTAACCTTCCTCCATCAGTACTGGACTTCAACTCTCATAGTCCACAGGAGTGGCTAAAAAGGGTGGCAGTTGAGGTGTGCTCTTTTATTAAATTTGAAACTATGTTCATCCTCTGCATCCTACACATCAGTTTTAGCATGAACAACATGTGCTCTTGTATTTGTTTCTATAGATATTTGGCAAAATGGAAGCCTTGCATGTGGAGGTGTCTGAATTTCTTCACCGTACTGAAAAGAACATTGTAACTGAGGATGAACCTGTGAAGGAATGTGTTCAAAGGCAGATTATCGAGATGAAGGATTTGCTTAAAATGGAAAGAAATGAATATGAGGTGGCACAATCAGACTTTCCTGCCATATCGTTAACTTTTATCAAGAGTTACAGCGATTTTATGTTGATTTATTGCCCCTTTTCTTGCAGATATTGCTTTTACCAGTCATGAGGGAAAGCAATCACCCCATGCGGACATCAATTGATATTCTGGAGCTCAACCGCTTGAGGCGTGGTCTTCTTCTTGATGCCTACATTTGGGATCGGAGGCTATGTCATGTAGACTCAGTTCTTAAAACACATGGCCATGTTTCGAAAACCAATCCAGACAACCTTGATGTTCTCTTATACACCAGATTGAAGGAATGGAAAGCTGATTTGCTTTCTGGGGATACTGAGATTGGAAAATCTTTAGGAAGTCCGAGGAAATCTTTGTTATCCAGACAGGGCCATTTGAATGATAATGAATACAGTGTTGCTGATACAAATTTAGAGATGTGTTTGGAGGGTCATCCTGTGGATGATGCAGAGGATCTTGACAAAGTCTATAACAAATTCAATGGAGAAAAAAAGTGGCCTATTGCTGAATCTACTGATGGTTTGGAACCTGTTGAGAGGTTACCCTCACTTGCATCGATTTTTTCTGATAAAATCGATTTGGCATGGACTGGGTCTTGTGATTTGCATTATGATCTTCCACAAGCTTTCACCAAAATTGATGACAATGTATCCTTTAATTTGGGCAGTCCAAACTACAACAATATAGTAACCCCTGTTAGAATTCACTCGTTTAATTCTACACGGGGGTTGCGGCAGAGAGAAAGAACTGGATTAGCTCCAGCTTCTTTGCATCTACCATCATTCAAATCTGCTGAGTACTTTGGGGGTATGACAAGCATCCTGAAGGACCCAATGCCAAATATACGAAGGGCCTGTTCTCAAAGGTCTCCTGGGGTGATAGAGAAATTAAATGTTGTTCTTGCACGCACACCCACATATATCTCATCTGCTTCAAATATGATCGATGATGGGGCACGACTACTATTACCCCAAATTGGATATGAAGATGTTGTCGTGGCAGTATATGATGATGAGCCCACCAGTATTATATCATATGCCATGACATCAGAAGAATACGTACAACAAGTAGCACACAGACTGAATTCCAGTTTGAGTTTTTCTCATCTGCCAAATACTACTGAGGTCAGCAGCCATGGACTTGAGGTGTCCTCACCCTCTCAACAAGACCATTTGCATTCGAAAGGAACTCATTTTAAGTTTTCCTTTGATGATGACTCACCGATTTCTCCAGATAAAACAAAGTTCTCTGTGATCTGCTATTTTGAAAAGCATTTTGCTGCGCTTAGAAAGAAATGCTGTCCCAAAGACATTGATTACATACGTTCTCTAAGTCGCTGCAAGAGATGGAATGCACAGGGTGGAAAAAGCAATGTTTACTTTGCAAAGACGATGGATGAAAGGTTCATAATCAAACAAGTCACGAGAACAGAGCTGGAGTCTTTTGTAGAATTTGCTCCTCAGTACTTCAAGTATTTAATGGAATCCTTGACTTCTGGTAGCCCAACTTGCCTGGCCAAAATAGTAGGGTTATATCAGGTTTGTTTTTGTTCACTCGTTGCATCATGTTTTATACAACTGTAGAGTGTAAAAGTAAGACATCACCCTGAATTCTATTTTCCCCTTTTCACTTGAGTTCAGGTTAGTGTTAAGAGCATGAAAGCTGGGAAGGAAGTGAGGATGGATCTGATGGTGATGGAGAATATTTTCTTTGAAAGGAAGATATCTCGGGTGTATGACCTAAAGGGTTCATTGCGCTCACGCTACACAGCCGGGGACAGTAAAGTCCTCTTGGATTCAAACCTCATAGAGGCATTGCATACTAAGCCTATATTTTTGGGGAGCAAGGCAAAACGAAGATTGGAAAGAGCTGTCTGGAATGATACTTCATTTCTTGCGGTATGCAACTACATCTTTGTGCTTACTGTTTAACCAATATATTTAAATACTTAATTAATTTGCCAAGCTACTACATAAGAACTAAATTATGGACTATAATTTATTTATAGGTTATTATTGTTACATAATTGTTTCAGGGATATTGCCGGATGCTAACTCTTTTTCCATGTTTTACCTGGTATTTGTCACCCACGAAATGGGTGCCCTGCATTTAGTTTGGAGTCATGTTATGAGTACCGCTTCCATCATGTTATAGGTTTCCAGATTGCCACAACAATGTATTTTGCAATATGTTATATCTTGCATGTGTTTCCCCCCAGTTCTGTTGAAGTAGTTCACCCTGAGTGACTATGTTAATCTACCTCATTCCTCACCTCCATGTATACCTATTTCAGATGGAATTTGCACTATTGATATAGTTTTTGGCAGTGATTGCCCTACAGAGCATTTTATTTTAAAACTAAAGTGGCATTTAGTATCCACTGAATTGAATAGGGGTCCTGTCCCTGCAATGGATGTGAAAACCCGGAGTTGTAATTCTGTCTGCTATGCTTTGCAGTTGGCGGATGTCATGGATTACTCTCTCCTTGTTGGAATCGACGAGGAGAAGAAAGAGCTCGTCATTGGCATCATCGACTACTTGCGCCAATACACCTGGGACAAGCAGCTGGAGACCTGGGTGAAGGCCTCGGGCATTCTTGGGGGCCCCAAGAATGAATCCCCTACCGTCATATCTCCAATGCAGTACAAGAAGAGATTCAGGAAAGCCATGTCCAAGTACTTCCTCACTGTCCCTGACCAGTGGTCCTCGTGACGACTGACTAACATGCTCTATCGATCATCCGCCATAACCAGAATCGTTGCCGTGCCATAGACCATAGTACATGTCCATTATTCTTTCTGTGCCTAGAGTGAGAGAAATAGTCAATTCATTTTGTGTCCACCTGTGCCATCTGTAGTAGTCATAGACATACGGGAGGGATCATCCTTCAGCTGGATGCCAAGCATGGTCACTGCAGTGTCCAGCTGCAGATAGAGGCTTGGTCCATGACTCCATCTCCATGTACATTACGGCCAGCATATATAGCTGAAAAAGAAAAAAAAATGTCGATGAAATGCAGACAATTCTTTTGATGCCCCTGTGGCATGTATGCAGACTGTTGCTACTTTGTATAGAAGAGACATGCAACTTGAAAAACTTGTCTTTCCAAGCTGTCGCTTGCAGACCTGGACGTGAATGAAATGGGCTCCGAAAAAAGAGGGGAGGGACTTCTTGGAACTACCCATACTGATGGACAAGTATTGAAGTCGCTGTATTGAGCCACCCAGGACGAAACTCTTCAATAGTAGTGAAGTACGTATATATCAGCGCACCAGCACTGGACCATCTTTCTTCGATTCGGTTGTGGCATTCATGGGAGATCCTATGCGTTCGCTTTCTGATCTTGACGGTTCTTGTCAAATGCTCCTAGCTAGTTGCACACAACAGTTAGACATCTTCTTCTGGACAAAGACATCGTCGTCGTAGGCCTTGTTTAGATCCCCAAATTCCACTATGCAAAAAGAAGATTCCCCATCACATCAAAATGTCGGCATATATATGGAGTACTAAATGTAGATGAAATCAAAAACTAATTGCACAGTTTTGTTGTACTTTACGAGACGAATATTTTAAGCCTAATTAATCAATATTTGGACAATTATTACCAAATAAAAATAAAATTGCTACAGTGTGATGGGACATTTACTATGCAAAGTTTGCATCCCAAATTTGGGATCTAAACAAGGCCGTAGTAGTTGTGAGTTGTGACGCCAGGTCTGCTCTTTGGCATGTTAACGTAGTGCTGCCACAATTAATAAAGGGCCTATTTGGTTTCCAGAGTTAAAGTTTAACTCTTATCACATCAATGTTTCCTCAAATGCATGGAGTACTAAATGTAGATTAATTACATGCATGTCGGCACAGATTAGGAGTAATTTACGAGACGAATCTATTAAGCCTAATTAGTCCAAGATTGAAAACTTTTTGTCAAATAAGACGAAAGTACTACAGTGTTTATTAAACTTTAACTCCTCAAATAAAACACCAACTAAACACACACAGACGTACGTACACTCGCTAGGGCCTAGGGGACTAGGGGTCACAGGCCGGCGTCGGCGTGAGACTGTAATAGGGAGGGGTTGGTGCTATCTGCAACGGCAAGCTCCGACCCGAACGTGAAGTGGAGTGGTCAGCTACTAACCACGTGTGTCAGAGCCCGGCAAGCTGCACCCACAAGTCCAACCAAACCAGCCGGCGACCTTGGCTACTTCCAATACATCGCCTACAATTATGCATCAAAAGCAGCAGGATGTCTAGCAGCTTGGAGCCAGCCTGCCCTTGGTCCTTGGACGTCTTCACTCTTGAGGTAACAATGTCTGACGTCTTCAGAGCATCGATTCATGCTTGTAAAATGCATGTTTGCTGTGTTTAGATGTGTAAAAAGATATTAAAAAAAGTACTGTAGCACGTTTTGGCTTTATTTGGCAAATAGTGTCCAACTGTTGACTAATTAGATTCAAAACGTTCGCCTCGCCAAGTACAACCAAGCTGTACAATTAGTTTTTGATTTCATCTACATTTAGTACTTCATGCATGTATCGCAAGTTTGATATGACGGGGAATCTTTTTTTCGCATAGTGTTTTTTTCGTGGAACTAAGCAGGGCCGTTGTAAGAACGATCTATTGCTTGTTTTGTCCCACTAGCGTAGCTTTGCAACAAGACAAGTATAAGTGTGTGTCTCTCTCTGTATATACATACATGACAGCGCTATTCTACACCGTAAGCCAAAACTGATATGAAAAAATACTGAGTAGTATCAGTCAGTACTACAACCAGAACCATGAAAATACTGAAACGCGAACACTGATCGATACTGAAATTGTTCAATATAACAGTTTAGTGTAGAATAGCGCGTATATGACTGATGATCTCTCTCTTTATTTGTGTTTCATGTATTCACAGGGCTCATGCGTGCTCCGTGCTGGGCTTTTTGTCAGGTAAACAGTCCTTGGATTTGGGCCTCCAGTTCGTTGTCTGTTCGACCTCGCATTAATTCACTTGAACCCACTGCAAAATCCGAATATATATGGTTGGAACCACATGAGTCATATTCTGAAAAACCTTATTTTGCGATGAATTCTCACGTACAACAATTTCACATCTACCTTCTGTAGCACGCGCTGCTTTACACTGTACGTCCACTGTTATATACTATTCAGCTACTGTTATTTTCCGCTATTTGTGAACAGGCGTAGCGGAGCGCGCCCGGTTGTGTTAGTATATATAATGAAGATATTATTGTAGATCTAACTCGAAATTTTCTGTATGCATAATATAATGCGCATAGCTTCCACTTTTCTGTGGTTGTAGGGAGTAGTTTGTGCGGTCATTGGTCATAAATTTGCTTTCAGATAGATATATATAGAGAGAGAAAGGATCATCTGTCTCTTGAAGTTGGATAAAATAAAGTACCCACAACATCTATATACACAATATAAGAAGGATCATTTGTGTCTCGAAATTGACTTGTCTAACTTCTTTTTGATCTCACCTCTCTTGTGCATCAAGAGACAATATTGTGCGTAGTTACTCAGCTAGCTTAAGCAAGGTTCCTAAAACCCTAGCTATGGATGCCACTATCTCTGGCTATAGCTATTTGAGGATGATCCAGCTATTTGCTTCAATGTACAATTTAGCCTCTATAGCCCAATTTAGCCGGTCGTTTAGCTGTTTCAAGAGGTAGATCACTAAATGCTTTAGTCGGCGATTTAAAACACTAAGCTTTAGCCTTGCCATGCCTAGTGTCCAATATAAGTATTACACTAGTTAGTTACACAAGTATATCTTGGAACGGAGGGAGTACTACTAGAGAGAAACACTAGCTTTAAGCATAAATGGAAGATACGTCAAATAAAAAACCAATAGCAGTTTTAGAACTCCCCGCAATATAATATATTGACCATTTGCCTGTGTGCTTGTAGAAATGGATACATACTGTCTCTTATACACATCTGACGCTGCCGACGATCTACTCTGTGTAGAT

>BAC-81

GGCGGGATCTGTCCCGTAAACATGGGCGCACAGCGCGCAGACGCTACAGAGCGAGCGGGTGATGCGGACAGGGGCGGCACAGTCTGTTCCGCGAGCAGGCGATGCGGGTGGGTCCGTTCGTTCGGATGGACGACCGAATACGAGCATTACCGAAATTTATTCTGCAAATCAACATGCATATTTGTTACTACGAGCTTCCAAAATTTTGATTTAGCGCTTGATGATGGGAAACCCAAATAAAATATACAGTAGTTCATTAAAAGTGGGAAATATGGGAGCCCAAAAATATCTTCTCGCTTCTAGAAGTTCCGAGAGAAGTCGTAGAGATAGATTGAAACATGGGAAACCCAAATAAAATACTTGATGTTCATAAAAATATCACTACAAGCTTCCAAAAATTTGATTTACCTCAACGAAAATGGAAAAAAATATACCTAGAATTTTTTTTTGGAAAAATCGACAACGAAAGCATGAGATGCAATTGTCATGAATGCACTCCGTCACATGCTTGCACGATGGGTACTTTTTAAAAACATTATACATTGATTAACGTTTTGGGATTTGTCTGGATATTTTTCCCATTTTCATAGAGCTATACCTTTTTTTTTGAAGTTTCTAAAAAAGTTTTCAGAAGACCTAAATTTTTTTATTTGGATTTTTCATATCCTAATCTATCTCTATAATTTTAAGAATTTTTAAAAGCTTAGAGACATTTTATATGATTTTAAAACCTTATAAAGTATTTATCGATTTTTTAAACTAAAGAAAGGACAAGACCGTCTTTAAAACCACTCAAACTAAGTAGGGAGGTCATTTGAACTGTCTTGAAAGTTTAGGGGGTAAGAGGTTTGGTTTGGTGTTTGGGAAGAAATCAGACTATGGGGATAGTTCATGGAGGTAGAATAATTTTTTTCCTCACCGGAAAACAATAGATGAAGAACTAATGGTGTGGGCCTTTCGTGGTTGGGCCTGTTGCAATTGTCCATCAACTGGGCCAAACCTGAGGCAAAGAATTGTCCATCAAATAAATCAACGCTATCGCTAATTCTCCTATTCATCTTCCGCCGCCAGCAAGGTTCATGGCTAACCGGGGCAGCTGCTCGAGGCCTGTTTCATGTTCAAAAAAAAAAAAGAAAAGAAAAAAAAGAGGGAGCGTTTCTCAGCGCCGTGCCGCCGTGGCTAGCTGGGGCAGCTGCTCGAGGCCCGAGCCTGCCGCATGGGGAGCTCCGACTGTCACCGGTTCATGGCTAACCGGCAGGAGGAAGAATTTCGTCTACAAATGTTTACCTGCACCAGTGCATGCAATCCCCAGCAGCGACCTCTCCAAGTCACCCTACACAAGAAAGTCAACGCCTGCGATTGGCTGGCCGACAACAACCTCCGCCGTCGCTTTCCTGCGGGTACGTCGGTCATCACATTCTGTATTGTTATTGTTGCCTGTCGTATTGTTTCGGATCCAAACGCGTTATGCTGTTTCCGCGACGTCGTCTTTGCATAAATAGATCCACCCACCAGGTTCATCTTCCTACCTTTCTTGAACCAACCACTCGGATCAAAGCCATGCTTCATTGCCCTTAACAGGTGATTGATTTGGTTCCTTCTATCTGCCTGTCGCTCGGCTTATATATAATTCCTCAATAGTTTGTTCTACTAGGGGAAAATGTTATGTTGTTTCATCTTTTATGATTCTAGGATCTGGTTTCGTAGTGCTTCACTTCTTATAGTTCTTGAAAACATGTATATATTCCTTTTCTCTTCCTTGGGTGCTTCAATTCTCAAGGTTTCCGAAGAAAAGACGTTTATTGGCCTTTGATCTTTAGTAGGATTTAGAGATGTGTGTGTGCCTTGCTTAGCATGTGATTGTTGAAACACGATTCTATAGATCCATGCAGGGTTTATGAATTTGACAGCCCACGCCCAGGAATAGTTGAAAGTTGTCTAAAAATTTGGGAAGATGCGACGCGTTTCCGAATTAATGGAGAACCAGTCAGCTCACCCTCAGCATGGTATGGCCGATCAAGGGTTTCGCCTGTTGGCTACTACTCCACTGAGTTCTTCAGTCAACACAGCACCGATTATTATACCGATCTGGGCACCAATTCAGATGCCGGCTTTGCCATCTAATTCAGCAGACGATGGGTACATCTGGAAGAACTTGGGTATGCAGGAGATTCCGGGTTCAGGCCCATTCATCTGCTACGAATGCTCGCAGGCAGACTGCACGGTCAAGAAATCGGTGGTGCTATCTGCAGATGGAAAGATTTCGGAGATAGTTTACAAGGGACGTCACAACCACCCCCGTCCACCTTCATCAGAAATGTGCCCCAGAGATGTTCCAACTGGATACATATCAGACTCTCAATACTATCATGTGCCATCTGAGATGTACATAGCAGGGACTTCAATTCCTGAGACTGAGGAGGGAGGTGAACAGGAACAACTTGGCAGTTCTAGTGACAGCGAGGAGGAGGATGGTGGCGAACAAAGAGCCGATGGCCATGTTGCCGGTGCCAGCACAACGGAAAGGTTCTGACTAGACAAAAAACTTTGGTTACAAACTAATTTCTTAATTCTGTCGACACTTAATTAACTTACTTTCACTCCACAGACATGTGGCAGCTCCAGCTAAAAGGAGAACAAGGAGCGGGACTTCTCACCAAAAGTATGCATCAATTTTGAACTAAACCTGCTGCTTCTGTCTTCTTGTAATCTTTCCATTTCCCTTATTGCAAAGTTAAAGTGATCTATAATTTTGTGTCAAAAAAAGAAGTGACTTATAAATTGAAATAGAGGGAGAATAGATCTCTATCGAAACAAAAAAAAATCATGCAAGTTTTTTTTTTTTTTGCAACCTGGTGTGTACCCGTTTTTCATTAAGCAAGTAAAATTTTTGAAATGAAGATTGATGGGCATGATATGCAATTAATTAAGTCTCCCATTGTAGGGCCATGATATGCAATTCCTTTATGCATTCTTAATTAATGCTTTATGAAATGATGAGTAAGCTAAAAACAAGTAATTGGAGCATATGAGAATTTGACGATTGCTTCAGCAGGGGCTTAATGACCAATGAATGTAATCGGTGCTAAAAAAACTCTGATATTCGTCATGCTTTCGATGCATATGAATGGGGGTTCATCCCTGAAATTCTAAAAAATTAAACAAGTAGGATGCTCAGAGTTTTACAGTAAGTGACAAGAGCCATATTATCTTGGATTTACTCTGTAACCTGGATCATAATTTGTCTTGGTCTTGCTATGAATCCTGTTTCTGTGGTTCAGTTTACATTTTTTAACCACTGATCTAACAGCAAACATTTCGTTTGTTTCATAACACTTTTTCTCAAACATTTGTTTCATAATTCTGATCTTAATACATGACAGTGATTTTTCTTCTGAACCTTACACTAACTGGTATTCATAATGTCATTTCTCTGACACCGGTGAGAAAAAGAAGACCAGAGTCCAAAGTGTGGGAGGAATTTACAAAAGTTTTTAGAGATGGAAAACTTCAAGCAGCAGTCTGTAAACATTGTGAAAGCTCCCTTAGTGCAAAAACTACAGGAGGAACAAGCCATCTGAGAAGACATCTGAAAACATGTCCTGCACGACCTGCAACGGGTCGGCTGCAGCTGCAACGACCGTCTTCACATCCCATTTTATCTGTTGAGAATAATTCGAATTTAGATCAAGACAAGTCTCTTGGATTGCTTGCTAAGGCTCTATTCTGTAACCTTTGCTCCTTTTCATTGACAAGCAATACAAATCATAGGCAACTTTTGGCTGCCATCTGTCCTACTTATGACGTGGTATCGCAATCTGCTATTCAAGAAAAGTTTCTCAGTATTTTTCAGAATGAAAAGCTGAAGCTGAAGGAGGAAATATCACTTGCACCTGGGGGAGCTTTCCTGACAGTAGCAAAGTGGGTTCTTGGAACTAAATACTTCATATGTGTGATGGCACATTTTATTGACAAAGAATGGAACATGATTAGAAGAATCATTAGATGTAGCTTTGCAGGGTGTAAGGTTGACACTGCAAGTGCTTATATTAGCATGTTTCCTGATTTTCAATCGTACCGTAATGTGAGGATTTGGGACCGAAAACAAGAAGAAGAAGAAGAAGAAGAAGAAGAAGAAGAAGAAGAAGAAGAGGATTTCCAACCTGACACAGTAACAATAATAAAAGAAGTAGTCCAAAGTTGGAGTCTTGATGGGAAGCTTTTAGGAATTTCATTCCCCCCGTCATTATTTCATACAGATATATCGGGCTTGCAGAAGAATCTTGCAGAACATAATTATCTCGTCAGAAACAACTTACTAAGTTTACCTTGCATAGTAGAATCACTTAGTGAGATTTTTTGTTGCAATGATTTCACTGTCAGAAAACTATGTGACGACTGGTTTACATACATGACGTGCTCCCCACTGCGTTCGGAAAAATACAAAGAAATCCTTTTGCAACTGCAAATAAGCCGACCCACTTTTGGTTCCCAGAAATGGTACTTGGCTTTCTACTTCCTGGAGGCTGCTCTGCAATATAACAAGGTGTTTCCAAACCCTGAGCAGATAGATTCGGGATTTTATGCATCTGAGTCTAAGCCATCCGATAAGGAAGTACAGGATACAGAGGATTTTTGTAATATTGCAAGGGTGTTTTATGACGCTATTCAAGTGGCTTCCAGCCCATGCAATGCGACTCTGAATTCAAATTTTCACACAATTTGGAACTTGAAGATAGCTCTGACGAGGTCATCTGGAAAGGCACAGAAACTTTTTCATCATGATTCAATGAAGAAAAGGTTTGATGAGTTTTGGGAGAAATGGTTCCTGTGGCTATGTTTGGCCGTCGTTCTAGACCCAAGATATAAAATCAGGTTTCTTGATCACTCCTTGAAAGAAGCTTTTGGTAGTGATGCTAAAAAGTACATGCTCGAAGTGCGTGCAAAGATTTGTGAGCTTTTTTTTCTGTACTCTATTCACGCTGATCATCAGGGTGGTGAGTGCTCAAATGACAGCAACACTGATGTACCTGTACATGAGGGGCTGGGAGAGCTTAACCGCTACCTTGAAGGGGAATGTCTTCCTGAAAATGTTCCTTTCGATATTCTGAAATGGTGGAAGGGCAATGCTTCGATGTATCCAACTCTTGCATTGTTGGCACGCGACATCTTAAGTATACCTGCATGTGTGGTCTCCTCTGAATCTGCATTCGATGAAACTGATGAACGGGTGAGCCTTTTCAATCGAAAACTGAGTCCTGAAGTAGTTGAAGCCCTCATCTGCACCCAGGATTGGATCAAATCTTCAGGTACAAGTACCACTAGCTCCTTATCTTTCGTAGTTGTACGGATCTCTTTCATTTATGGTGGACAGAAACCAAGAACCTCTAACTGTAATGTTGTTCTTCTTTAATTTCTCATCTCAACAGAAACAAATGACCAAGTCTGTGGAAACACAAACATGCCAGCTTAATACACGGCAAGGTATGCTTGCCATCTTTGTCTTCTGTCCTCTTCCATGTATAGGTTCTAAAGTCGATTAAAATTATAAACCTCTCACAGATCACTTTTTTCTTTTGTTTTTGTCACAACTGATAACCCATTTTCTCGTCCCTCAATTGGCACCCCATAAGTAAACATGTACATGCACTGAACATAAGACATACAACTCAATTTCCACTACTTTTTCTGTTTTCTCAAAGGAAACCCCCAGTATATTGGAAGTCAGCCACCAATTGGCAGTTGGATCAGTTGCTAGCCAGATCTTTTAGGAATTTTTTGTTCTTCTATCTAGTACAGAACTACAGATCTTAAGTCACTTTGCCATCTTTTTCATTTGTATCTGCAAAATAATTCTCAGCATTGAAACAGAGACAAAAAACCTGATACTTGAATTGGGAAAATAATGAATCTGATTAGTCCCAAATCAATTACATTATTCGAAACATTCAATTTCATTGTTCAACATTGCATGAAGTTTCAATTGGTCTCCTGCATTTCCTCTTTTTCATAATTAATGTAATAGAAATAATTAGGGTGCATACCATTCTCTATCTAGTCACACTCCGATACCCATATTAGAATCTCAATCTAGTCCCATATACTTGTCTCACAGATGGTTAAAAAAACATGTGGGTTGTGAGGGGTTGTGAAATTTTCCTTCCTTTTGTTAAATGTTTAAATATAGATCTGATGTTGTTTTGATTAATTAGTCTTTTGATTTCTGTTTAAACAGCTGCTTTGGCCTTTTCAGTAGTTCAACAAGAAGTGAACTGTGGTTTCGACCTCATCTGCGCCTTACCTGTCTAATTATAATGCATCAGCATTAGGTGACGCCGCGACAGAAGCCTAACTGCCTATGGTTTACAGTTACTTAATTGTTTTGCTACACTCACCGTGAAAATATGAACAATACTATCCTGTTTAGTAATTGTGCAGTATGGAGATGTACTAGTGTGATATATACTCCTATGTACTAGTAAAAGATATGGAGAATATTTGTCCATTAAGCCATCACTAAAAATATGGAATGAATACTACGTGAATGGTTGAAATATATATACGAACTTAACTCCCTTTAGTTTTATGTGTTTGTTCGACAGCTCATAAGGCTCTAAATGAACAAGTTTCATATTTGAGCTTTTCCTAAAATGCTGAAAGTATTATTTGACAGATAGCAAATCAAATATGACAGTTGCGATACATCTCTTATAATATAATTGCACAAGAAAATTGATATTTGTCATATGATTTGATATACTGTTGGGTTATCTTGTACAAGGATTTAAAATGAATTGTAACTGAAGTCAGCTGTGCAGTAAAGAATACACGGCACGGATCCATTCATATCCATAGAATTACACTTGCTAGTGATAGATTCATAAAATGTCGATACCCAAGCACCTCTAGATTTAGGTCTTGTTTGGATCCACTAGTGCCAATATGCTTGTTTGGATCCACCAGTGCTAATAGTTAGCTAGCGAATATCTAAAAAGCTACTAACTATTAGTAATTATTAGTAGGGGTTGTTTGGATCCACCAGATAATAAGTGGTTGGATAGTTGGTTGGAGATGTATATAAACTATTAGCATCCCTTCATCAACTAATGTACTAGTTGTTAGCACCTCCACTTGCTAATAGTTAGCAGGCCTCTTTGGATCCCCACCAGCTAATTTCAGCTGCTAACTATTAGAACTAATGGATCCAAATAGGCTCTTAGATGTGTGAAGGCACCAGAGTTAACAGTTAAATTTGGATTCCTCCATATACTTCTATTGTTGATCCACTGAAACACTGCCATGAAAATTTTAGCAAAGGTAATCTTAGAACACGATTTGCAAGGTAGGAAAGTCCCATATATAATATTTTTAGTTAGAATAAACTTGGAATGTCTCTCGACAACAACGCTGATCAAGAACACTGATTCAAAAGACTCAAACATAGCACAGCATAAGAACCACTGAAAGACTCAAGCAAGTAGTTTTACTCATTACTGGTTGAAAGCAAATGCATAGATCTCAAATCATCCACTAAACAAAAGAAGAACATCGCAATGTTTGGATATTCAAACATAAGTTCCATCTTCCACTTTACCCATAACAGGCACTAGAAGCTGTGTCACAGTTAACTGGATATTTGTCTTGCCGCCAGAACTCCAGTTCATACCTGGCAAGATCTCTCTTCATCCTCTTGCCACAGAGATGTGGAATAAAATCAAGCAGCTGATGGATTCATAGATCAAGATCAAGCACAGAAGAACAAAGAATAACTTGCTTGCCCATTTGATAAACTAGCCTGCAAAAAAAAACATCAAATGACTGTATATCTATTTAATAAATGCATTTAGCCTTTCTTGGACACTTGGAAGCAGTTGCGAAGCTTCACATAAATGTTAGAGGGGGGCACTAGTGTTAAAATAGCTAGGTTAGCAATGTTTAAGCGATTAAGTATAAAACTTCAAAGTAATGGAAAATCAGGGAGGGCCATTGCCCTTTTGGCCTCAATGAAGCTCCGGCAGTGTTGGACAAGCTACTTGAGTAGTAATATAACACAAACACTGTAAAAAATATGAAATTAGGTACGCACAGAGAAGGTATGACTCACAGCTGCTCTCTGTTCCATGGGCGAAAATATTCTCATCTGGTGTTACCATTAGCCCTAAACAAGTGTGCCAGCTCATGGACTCTCACGTCATCCTTAGAGCATTTGATCTTCACCATTCAAAAATGTTTTCAAGCAAATGATCTTCACTTTGGTTTGACACTGCTTTCCAGTTGCTTCATGGAGTTGAAATTCTTTTTGCAGAACAGCAAGACTGTTATTAGACATGTGCCTCGATAGGCAAATACTCAAGCTCAAGACCTAGCATACACAAAAAATGTAAAAGCTCAAACCAGTTTTAGTTCAAGAAAAAGCCTCTCCTAGTTAGGTGAATGCTGCAGCAGGAAAATTAATGCATCAAAGCCAGCATCCAATCATCCATACACCATTCACCAAGGGACAAGGTCTTCAACGTTCAGGTTGCTAAAACTTGGGGCACCTTATAGTCAGAATCTTCATAGCGTAAAAGTGGGTTTTCAGCAAATAACAACATTAACCAAATAATCACTCATATGGTGCATGCAACCAAATCCGTCAATACATAGAGAAGAAAAGTAAATAACACAATGGCATATTGCCAAGGAAATATCAGCTTTGCTAAATGGGATGCATATTTTATTTAAAATGGGAATTGTCCATGGGCTAGTGTCAAGGCACAAGATTTATTAGACTTAATTCTGTGCTAATACATAACAGCAATCATTTCAGGGAGATACGTTATTGCAAAATTGCAATAAAAGTAATCAACATTGCTTTTCTTGGTGAAACAAACTTTTGCTAGACAATCAAATGAGTTTTTGAAGTTGATGGAAAAAGAATAATACTCTTTATCTGGACACTTTCCTATAAATCAGCCGTGACTAATTCAGGTGACAACGCCAAAGGGTAAAATGTGGATTGGTCATCCTGTGATCTGGTGAAAGTTAAATAGGGCTTCATTGACAAAGGAAATGAGACTATTCCCCTAAAAAATTGATTGGGGAAAACAAAAATACATACTAAAACTTAGACATGGAGATCAGCAAGAGATGGTGATACATATACCTCTCCAGCATCAGCTAACACCTCCAGACATGTAGCACTCGAAAGGCTCTCAAGAACATCACGGCCACCATTAATTTTGTTAAAATTGTATCCAGATTTCACCAAACACATGATGGTTACCATCTGTACTGGATTTGCAGACATCACCACTGAAACCATACTCATCACAGTCGTTTGCAATCTCACTATATTCATAGGTGTTGTCATCACTATCATGTCCCTACCAATACCATAAGCATAATCATCCTTGTAACCAAGCCCATATCCTTTTAAAGGGACACCAAATCCATTCCAGTCTTGTACTTCTTATTGTTATCATCTTCGTCAGTGGTTCCACCAGGTTCGTCCTTGTTGAAGTCTTCGAAGTCATCACTGAAGGCATAACCATCAAATACGATAGTGCTTGTGACAAGAGACCCCATGTTCTCAAATGATGGAGCCTGGGTTATTCGTGAAATGCAGAGCTTCCGTACAAGGTTCAAAGCAACAATGAACACATCTGACACTTGAACATGGTCAAAATCTTTCAGGCAGGCAGACGAAATCTCATGGTCAGTCATCAAGCAATCTTTAAGATCCAGTTCTTCCAAAGACGGGCAATGAGACAAAAGCTGCCTCAGGATGTAGTCAACGAGTAGGGCATAGCACAGTTTAAGATTTTGAGGTGGCAACTGGCAAGAGACGAAAGTTGCATGCTCAAGCACTATGATAGCCCTCATTGCGATGCGCCATCAACATTGCTCGACGGAACAGGAGGCGCACCGCGCACGAAGTCGGGAAACCATCTGGCGTCACGCCGTAGTCATCCTGGCCGATGCGGAGGTCATCGCGCGGCTCCACCGCCCACGACAGCACGCAGTCCCACGCTTTTCAGGAAGGACATGATGTCGTGCAGAGGTGCAACCGGAAGGGCACTGAGGCGTTCGGCGGCCGTGGCACGCTCCAGCGTTCCTGTTGACCGGCACCCAGCACTAGTGGTCTTCGGGGGCATTTCGTCGAGCAGGTGGCCTGCACGGCCACGATGACCAGGAAGGCACATGAACACCGGTACAAAGACCAGAGCTTTACCGCCGGAATTCCTGCAAGAAAAATCCCGCCAGAAGGGAATCATAAACCAAAGGCAAGATTGACTGGGTGCAAATTGGATTCTTGCAGGATAACTTTCAGCCGATGATGTCAAATTTTGCAGGAAAAAATGTGCGGAAGAAAACTGCATAATCAACCCAGGGCAAGGAATGGATCGAATTTATACACGAATATAAGAGTTTCTTGTGAGATCTTGAAGGAGTGCGAGCTGAGTTAACCTCGCACAGTCCCGCAATCCCGATTCGAATGTTCGAAATCAGGCTGGTTCTTACCATTTTGGACCAGGGAATGGGCTTCCAGGCATTTTGCCGCATTGGGCTGTCCACACCTCCACCCATGATTTCCGGTTTTTTTTTTCGGAAAATAAGATTATATTTTGCTGGCATGACAAAAACAGAACCATTTGATAATTTGAAAGACGGGACTTGCTAGCGCCCAGACATCCCTAACGTCACTGGCATCAAGAAGAGGGGGAGAGGGGCGGCGGATGCCTAACCGGCGTTGGGAAGAGGAGACACTAAGGTAAGGCAACAGAAGACGAGGAGAGAGATACAGCACCCGATCTACTTTTGAAATATCAGATAAAGCACTTGCAACATACGTCTGAAGACACATGAAACATTTAAAACATGCATCTGAGACACTTGTAAAAACACACTTGTAAAAACATCTCAAAAACACTTGAAACTATTGTAAAACATACGCGACATCCAGATAAAAACACTTGCAACGTATGTATAAAACACATATAACATATAAACAAACAAACTTACAACATACGTCTAGAAAAACAAATAAAATATTAAAAACAGATGCTTGTAACATACGTGTACAACTATTGCAACATATATACAAACATCCTGATCTATTTTTGCAACATCCATATGAAACACTTACAACATATCTATGAAACATCTGAAACACTTGAAACATAAGGCTAGCAACATGTGTTTTTCACCCTTCTTCCTTACGACGCAGCGCAGAGCGGGGGAACGGCTGGTTCCGGCCAGCCAACGCCCGAGGATGGTGGCGTGGCCTGGCAGCGGCCAGCTGCACCTACGCCTAGCCTGGGCCCAACTAGCGAGGGCCCCTATCCTGGTCGCCTGGCCTAGGCGCGGCGGAGGACGGAGCACAGCATAGCGTCATGGGGGATGGCGGCGGCGGCGCGTAATAGAGGTGCCACTGGGGATGGTGGTGGCGGAGCGAGGTGGGCGGACGGCAGCACGACTGACGAGCGCGGAGTGGGGAGATTTGATAATATAGACGCCTTTCTCTTTTGCCTCATCAAGGGACGAGTTTCCAAAAGGAAATTATTGTTCTCACGTTCTAAATCATAAGTCGTTTTCTAACTTCTAAGTCAGACCTGTCCAATTTTGATTAAGTTTATCATCTCTAAAATAAATTAAATCCTATAGTTATACTCAGTCAAACTATTTTATCGACTAATTTTATAGGAAAGAGCACAACATTTATACACCAAAAAAATCATGACATATCTAATAGACTAATAACACTATTTTTGTGTCATAATATTAGTACACTTTTTCTTGAAAGTAGGCCAAACTCAAAATAGTTTGACTTTGGACAATTCTAGACGTTGATTTATTTTGAAATGACAGGGGCATATAATATCAATAAAGTGCATTATTAAAGCATATTTCATGATATATCTAATGAATTTTCTTTGATGTTGTAGATGTTGTGTATTTTTTTATGAACTTAACAAAACTAAAATGACTTATGATTTAGAGCGGTACGAGTATGTACTTTTACAATAGCCATAACCTTATCATACTGCTACAGTTTTGCCTAAAGGCATGCCTAACAGGTATAGTTAAGGTATGTTCAAAGAATAAGTTTATAGCAGGTTTGAGACAGTGGCGGAGGAGAAAAAGATTTTAAGTTTTAGGTATGACAAAGTAATTAACCAACCAAATTTCACTGTCTTTTACCCATCTTTATTTGTTAATCTTCACAATCTCACTGTGCTTCATAACTCAAGTAACTAACCAGCTAAATTAATTAATAACCAAGAAATTGAACAAACAACAATGAGAAAAAAAAAGTTTCAGGTTTCAGAGTTTGCCTTACCGTGTGTTCTGCCATCGACCGCTTGTGGTGTCGTACTCAGGATATTAGGTTAGGATACAAGCTTGCTGGTTCAACCATTTCAAAAGTTAGAGGAGAGGTCATTTCCGAGATCGCAAATTCAGGATAATAAATTACATGAAATCTTGTAATAAACAAAAACAAGCAAATAGCCGGTCGTCTCGCCAACTGCAAGATTCGATATAGAAGACAGAAACACAGCACAACACCTTTCTCAGCTGCAAGCCAGCAGACCATTGAAACACTAAACAGATAGCAGATTTACTCATTATTGATTGAAGGCGAAATTAACAGAATGCATTATATGTAAGTCTTAATTCATTAACTAGAGAAGATAGTATCGTTCCAACATTGGGGTAAACATACATAAGTTCCATCTTACGACAATGTTACCCAAACGAGGCATCAAGAGCTGAGTGCCAGAGTCACAGTTTCACAAGCAAGATCAGTGCTTCGTGAGCTCAGTGAAGCCTTACAATCACATGTAACAAAAAACTATTAAACTCATCGCTTAACATCAGATAAGTGAAGCTGGATAACTGGACAACACATTAGTCGTCCCCCCAGAACTCTAGTTCATGCCTGGCAAGATCCTTCATCGCCTTCTTGCCACGGAGATCTGTTAGAAATTATGAAATATCAAGGCAAATTTGGCTGTGGGACACTCGAAAAACATGTAATTGGCTGCGGCACACCGCCAGCTGCGATATTGGCTGCGGCACACTCGAAAATCAGGTTCGCTTGCTGTAACAGACCACCCCCATTAAAATATAATTTTCGCTGGTTTTGAGATAGAGAAGGTAGGGGAAATGACGTGTTTGCCCTCATCTTCTTCCTCGTGTTGGTCCTTCGCGAACAGCCATGGCAGCCATGGGAGGCTGCACTGCTCCTCGCGCACAGGGGAGAGAAGGGGGCGAGCATCGGCTGGTCGGCCAACCTACGGCACGTGGCGGCGCCAGGCAGCACCCGTGGCAGCCTGCGGCGGCGCCTGGCCGCACCCGTGTGGGACAGCAGCTGCGTCTGCTCGCAGGAGCAGCGCAGGAGCGCTATAGGGACGCGAAGGGGTGGTGGGGCCAGGTGATGGGGCACGGCCCGGGGCTCCGACATCGGCCCGGATATGGTGGTGCGATGGTTGGCTGTGGCGGCGGCGCACTGCAGTGCCGGCAACGGCGCTGTGCAGTGGCGCAGGGAGGTGGCAGCGGTGGTTGCGCACGGTGGGGGCGGCGGCAATGGTGATGCTCACCGAAGCACGCCGGTGGGAAGGTGGAGGCGCGGCGCCGGCAGTGCGAAGAAGAAGATGAGGGCAAATACGTCATTTCTCCTACCTTCTCTCTCACAACCAGCGAAAATTATATTTTAATAGGGGCGATGTGTTAAAGCAAACAAACCTGATTTTCAAGTGTGTCGTAGCCAATATCGCAGTTGGCGGTGTGCCGCAGCCAATTACATGTTTTTCGAGTGTCCCACACCCAAATTTGCCAAATATCAAATAGTGGATGAGGGCATAGTTTACAACTTATAAGAGTACAGAAGAAGAACAGTGCTTTAGTAAACAGAATGGCTTGCTTACCCAATTGGTACAAGAATATACTCGAAAAAATATCATGTAGCTATACTTCAATTGAGCCATGGTATAAAGCATTTCAAGGACAAAAAAAGTAGTAGTAATATAGCACTGGCACTGTAAATAATTATGAAAATCAGTCGTGCCAAGAGAGAAGGTATTGAAAGCATCTAGGCCCCGAGGTTTGGTTTCGGCAATTGATGACAACGCTTTATTACCATGACTAACGTGTGTTTTGCAGAAACTTTGGTAAGTTAGGTCATGGAGGTTATAACTGCTTTGGGCACCATGGTATTCATGCCCCTTGATGGAAATCATTTCGGTTTTCAAAGGAGTGACGTCAAGATTAAGGACGGTTAGTTCCGGATGTCGAGGTGTTGAGATACATTCGGAATAGTCGTTAGGATCTTCGTTTTTCTCTTTTG

>BAC-43

GAGATGTGTATAAGAGACAGATCTAGTATAGAAAGGTCTAAGCTTCTAGTGGAATCGAAACCTCATGAAAATAGCCGCTAGAGTAAACCAAAGAATTTTATTAAGCACATCAGCATGATTATAGATACAATTTTCTCAAACAGTTTCATTTCAAAATAGTTGATGCAGTGCTAAATTAACTATATAGTGCCTATGAGGCATGTTCTTTTGATTATTATTAAATCAAGTACTACTAAATAAAAAATAGTTATTGCATTCCAATTATGACTGTAAAAATTGAAATACATTCATTAATCATGTCAAAAAAATGACAGCGAACATAACCATTTCTTGGCAGAACCTAGTGTTGCACTTGATTAGGATGCTGCAACCCTTAGTCGTGCCTCACGGAGCAGGAATGCAACCTGGAAAACAACTATCAATGGTAAGAAATTGACTATTGCTTTACAAAGAATGACCATTGACAGACATATAATGATCATTCTTTGGCTCATCGATATTGCTTTACAAAGAATGACCATTGACTATTCGCAGTTTTCAAACGTTGTCTTCATTATTGACTGCAAATAATGGTGATGTCGATGAGCCAGAGTTTTACAAGCGGTAATTTTTTCACCTAGTTGACTAATTAACTAACCATATTAGTACCAGATATATTCAGCCCAAGAGGCGAAATTGGTGGGAAAGGGGGAGCATTAGGAGGTTAGATTGCAACCACCGCTGCAGAGCATGTCTGCTGAAAGGAAACTCTCAAGGAAGCACTAGGAATGCGAGCTGAAGCCTCACACCTCAAGTGGGGAGCCTAGCCTCCTGCTCATGGGAGCCTCATGACCGATGAGCATGTATGTGGTGGAGGCCGATGAGCTCTGCCTATTTGCGCACTTGCTGGATCTGACAGAGAAAAGTGAACATGAGATGCAGATCTGAGAGAAAAGTGGGAGAGAGAAGGGTGAGAAGAATGGTTGTAGGGTTACCTGCTTCACGTCAGTGCCCTCAAAGGGGCTGTTGCATGCCCGCACCGCCGTATGTGGGCTCTCCATGTTGGGCGTCCTCTTCAACAGGGACGAGGGAGGATGGCACGAGCTTCGAACGACACCGAGCTCATGTGGCTCTCATGATCGAGCGATGTAGGCACCAAGAGGCATCCACGGGAGCATAGCGAAGGGCGGCGGTAAGGAGAAGATAGGGGAATGAAAGGGGAACTAGGGTTCTGGCTTGGATAGGGGCTCATTGCAAAAGTGTTGGATGGTATTAGGAGCGGATTTAGGGGGGTGGGTGGGCCGCAGTGCCCCCTCCCCCCCCCCGGTGGGCCACAAATACATGGAGTAGGTCAGCCCAACTCGTCTCGCAACCCAGAAGCCCCCATTGAAGCCCAATTCGAAGCCTCCTTCTGTTTTTAACCCACCTTGGGCCAATTCTGGATCTGCCCTGGATGGCGTGTTGATTCCCTAATAGTTAGAGGGTCTTTTGTAAAACATGTAGCGACATGTGTTGGTACACAGAGGAGCCACCAATACTCCGCATTCTCTGTTGCAAGGAGGAGGGAGTGAGTTCTAAAACACCTAATTAAGCAGACACGTGATCTTAAAATTGGGGTGCACTAGAGACTCGCTGGAAGGCACATGTGGTTGTGGGTGATATGTCTACAATTTTCCTTCTAAAGACTAGTGCTACATACATCCTAACCATATATGCATCACAATTCGCAGATTCTTCTGGAGGCAACATTTGCCGTGCAAGGCAGGGAGAAAATTCTTTATTTATATTGTTGAGATGATGTTTTTAAGAGTAGTTTTTTATTTGTCGCTAGAACTTTTTTCTTCACTGTTTACCACTAGAAAATATTTCCTTTCTTGTTTGCCACCCGCTCAAATTTCTAGAACACAAATAAAGAAATTTCTTCAAGTCTGCGGGCCGCTGTGGCGGAAGATTGAACAATGATGGTGCCTCCACAGCTACCGCTGTCCCCTTTTGACTGGTTTATTTCGTCCGACGTCGGATGTTGTTGGTGGCTGCCTGCCTGGTGACTTGTATAGTGAACTGTACTTGCTCTTTTGCTTGCGCCTGTATAGAAGGAGCGAGGCATAGGGCAGGAGACCCATAAGACATAGTCACATAGCCATAAGTTGTTTAGCTAACCTTCAGTTTGCAAGACTTGCTTATTACTTTGAAGGCAACTTTTTTTTTCGTGATCGGGTCTTTTACCCGTTTTTCATTAAGAGGAAAGACCATAAAAAAAGGCCGGAACATAGATACAAAGGACTCCCAAGTAATCATAAGATTACCAGCGGAATCCCAAAGACACAAACACAAAAAACCAAAACAAAACACAATCGAACTTTGAAGGCAACTTCATTGGAAATGTTTCCAGTCGAGCTTTGGTTGTTGAACCAGTCAGCATGCCTTAGGTTGAGTTCATGGTTTCATCTCTGAAAGATCTGTAGTTCATGAGCTACACCCCTAACTATGCTCAACCTCTCACCAGTTGGCTCTTCTCCAAGTTCAATTTGTCACTCCATGTTTGAAAGCTAGTTAATCTGTCTGATTCAGCTACACGGCGGCACAGCAGCAACGAAGTGAAGGACTGCAAGCAGAGCAGGCAGTGAATTAACTGGAGAGGGATGCATGCACGCACGAACGTATCGCTCGGTGACTCGGGCATGGAAACGATGGCACCGAGAGGGTGCAAGGTGCGGAGCTACGGATCTTGCTTTGTAGCCGTACTAGGGAGCGGAGCACACCCAGCATGGGGACCTCCAGCAACCCGCGCAGCCGCGCCGCCATGTGCGAGGAGTAGAAGGCCAGTATATCGGGCTGCCGGCATCGCCTCAGGGGGAGCTCCGCGATACCGCCATGGATGCTCGGAGGAGCCTTGTACCCCTCGCGTTGACCGACGGTGGATAGATGGCTTGTGCCATGTGGTCGCTGCTGCTGGTCTCACGGATCTAATCCGAGGACGCCTAATTCTCAAGAAGTTTGCTTGTTTATTGTATACTCTTC

>BAC-19

AAATTAGCATAATTGGATACGTTGAAATACAGTTTCATAATCTACAGTTTTTAATAGTAGATGCTAATGAAAATTGGTCAAACTTAGTCTAGTTTGACTTATGACAGAATTGGATCTTCTAAAATTTCAGGACAGAAAGAGTAGCCAGCTAATAGTTAACAACTACTAACTATTAGCTAACTAATTATTAGCATGATCCATTTGGATCCACCTACTAATAGCAGCTCCAAACCTAATAGTGGAACTAATAATTATCAGCCTCTTAACTAATAGTTAATTGGTCAATTAACATATCTGTTTGGACCCAATAGAACTAATTTTAGTTGCTAACTATTAGCATTAGTGGATCCACCCTTAGGCTCATATGCAATGCAGCCAAAAACAAACAGAAAGAGTTGCTTCTTCGGAGATACTAATACACAAGATAAAAATGCCATGCTATTAAAAGCTGCAGTTTCACGTCCATTGGAGTTTCCTGAAAGGCATCACCAATTACCCCTTTTCCAACTACGTGGAAGACGCTTGTGTGTACATAACATTACACACGATTTCCACCACTGGTTCTCTATAGCTTACACACAAGCAATTTTATTGCTCAAACATTCTTCCAAGAGGTGCTGCCTTTCCACCTTAAATCCTGTAAAGAAAATAACATCCCTGAACACCTCCAATAAATAAGCAATCATTTTAAATCCTTTAGACAGTGACCAAAGCACATAGGAGAAAACAAGCACATGAAGGGATGAGCAAAACGGCGTAAAGAAAGCTTGTTGGGTCCTTCTCGTCTTCCCCCAGACAGCAGACAGCTGAGCTAGAGCTGGACTGGCAGTCTGGCTCTGCCCTCCCAGTCCTAGGGCAATAGAAACCAAATTTATTAGTTGTCCTCTTGTTTTTTTTCTTTTCTAATTAACTGAACAATACAGTGGTAAATAAAATTAGCTGTGACACTTGCTACAGCAGTTACAGGATCACAGCCTTCTTTCTGTCGTTCTTGGCCACAAGCCCACAAGTCACGCACCCCACTGAAACCCCTCTTGTGCTTTTTTGCTTTCCGTGTGCTCAGCTCAGCATTCACAGTTCTTAAGCAATGCTTTTCTTTTCGGGATAACTTTTTGCTCTAAACTTTTTTGTACCATGATTTCTTTGATGAATTGACAGTGGTGCCCCTCCTCTTACACCGCCTATATATCCACCCTCAATCCCGCTCCATTGCTCTGTAGCTCTTGCAGTCTTGCCTCTGTTTCCCTCCGCTGCTCTCTCCGAGCCTTGCCCTCCTCACGCTCTCTATGCTCCTCTTCCGTTCATAGCCTCGGCCTTCGTCTCCCTCCATGGATCCGCGCTAAATGCATTGCGAGGTTCCAAACTGGTAGCATTTCTTCCTGCTCCATCTGCCGCAAGATGTAAGCAGGCTCTCACTTGACACTACTCCGAGAGGCGGCCATTCAGAATATACCCTCAGATCTTCTTGTCTGAGAAAAGCGACGCCCTTTTGGCCCGAGCAATGGAGGAGAGCTTCGTGCCCCTGCGGGGCATCAAGAACGACCTCCACGGGAGGCTCGCCTGCTACAAGCAGGATTGGACCGGAGGGTTCCGTGCCGGTATCAGGTATGAACTGAACTGAAGGCTAATCTAATCTTTCTTCGCAATCTTGCATTGTGCTGTTTAGGATTAGAAATTCGGTATCACCATGGTGTCAGAATCTTGAAACCGTCTGCGACGTAGATAGTCAGTCCTTTTGTTGTTGTTCTGAAGTTTGTTCTTGCCTGGCCCAAGATCTTGAATGAAAAAGGAGTGATTTTTTGGGGGTGTTTCAGGATCCTGGCGCCGACCACCTACATATTCTTCGCGTCCGCGATACCGGTGATATCGTTCGGGGAGCAATTGGAGAGGAACACTGGTAAATGGGGATTCAAAATTTGTACTTGCTGGTGTTAGTTTCAGACTTTCAGTACACAGTGGGTGAGGAGAGGAGCTTATTTGTTTGTTGGGGCATCAAAACTTGTGTGCAGATGGAGTCCTCACAGCAGTGCAGACGTTGGCATCCACTGCCGTGTGCGGCATAATCCACTCCATTGTGGGAGGGCAGCCCCTGCTGATCCTGGGAGTTGCCGAACCCACTGTGCTCATGTACACATTCATGTTCAACTTTGCCAAGGACAGGCCCGACCTCGGACGGAATCTGTTCTTGGCCTGGACCGGTTGGTAGGTCATCCTTGCGGCGCTGATTGAGAATTTTATTTCAGATTGGTTGGGTTTGTTCTAATTGGTTCCCTCTCTCTCTCTCTCTCTCTCTCTCTCTCTCTCTCTCTCTCTCTCTCTCTCTCTCTCTTTTGTGTGTCATGTATATGTGCAGGGTTTGTGTGTGGACTGCCATCCTACTGTTCTTGCTGGCCATACTAGGCGCATGCTCCATCATCAACCGCTTCACCCGCATCGCTGGCGAGCTGTTTGGGCTTCTGATTGCTATGCTCTTCATGCAGCAGGCTATCAAGGTTGGTGAAAACTGCAGGCCTTTTCAAGCCATTGCTGCTTTCCAGGCTTGAACCCCTGTACTGATGCAGAAACTTTTTTAAGACATGCTGCTGCTGGACCATAGAGCTTTTATACTAAACACCAGTTGCCTTGCCTCTCGAAATTGTGTAGTACTCCAGTCAATCTGAATGCAACTAACTGGGGAGAATCTAGTAGGTTGGTCAAAGGCATGTGAAAGTGCTGAAAAGCACATGGAAGCCAATGAAGATGCCTCCAGCTTCTGTTTTTGTGCCATTGGTCACTAGTGCAACCAAATACTTCAATGTTCTCAGTTTGGAACAAAATAAAAAAGTAGATAAAATCAAGAATGCTTGTTTACAAAAACAAACGGTGTTTTCCTGGAAAAAGGTTCCTCAAAAATATTTTTTTTAAAAAAGAACTACCATAAATTCATGTGTACTGAAGTAATTTGCAAACCGACTTCTCTTTGGCCATCTGGATGTGTAAGCTCACAAATCTGTATGATGGACACAGGGACTTGTCGACGAGTTCCGCATTCCTGAGAGGGAAAACCGAAAGGCACTAGAGTTTGTTCCATCATGGCGGTTTGCCAATGGAATGTTTGCAATTGTGTTGTCGTTTGGCCTTTTACTCACTGCCCTGAGGAGCAGGAAGGCACGGTCATGGCGCTATGGAACAGGTATGCATCCGAAAACTGTTCTTGTCCTGCAGATGCTATGAGCTATAATACTATATATAACTCTCTTTCTCTCTCTCTCTCTCTCTCTCTCTCTCTCTCTCCCCACCCTTTGGGTTGCTTATGGCTGTGAAGCACCTCACTTAGTCCAATACCAAATTTCTGATGATAATACTCTTGTACCTAGGTTGGCTGCGTGGCTTCATCGCTGACTATGGTGTTCCACTGATGGTGCTAGTTTGGACTGGAGTGTCTTACATACCTTATGGAAATGTACCAAAAGGAATTCCACGACGCCTTTTCAGCCCTAATCCATGGTCCCCCGGTGCATATGACAATTGGACAGTCATCAAGGTATTACAACATTCACAAAAAGATTCTAAAACATTATTTTTATTCATGTGTAACATCATTACCTATCAATTAAGTTTTTCCACATCGATCACATGAGCAGGATATGACACAAGTTCCACTCCTCTATATCATTGGTGCTTTCATACCAGCAACGATGATTGCTGTTCTCTACTACTTCGATCATAGTGTTGCATCTCAGCTTGCTCAGCAGAAAGAGTTCAATTTGAGGAAACCTCCATCCTTCCACTACGATTTGCTTCTCTTAGGCTTCCTGGTATGTGCACAATTCTTCAGTACCAAGTTTTGGTGTTTTCTATACATTTTGATAACATCCATGTTTTGAACTGTAGACGTTAATGTGTGGCCTTATTGGTATCCCTCCATCAAATGGTGTCATTCCACAATCACCCATGCATACAAAGAGTTTGGCTACTCTCAAGCACCAGGTGAGTTATGAAAGTTTTAAATAGTACTTCAAAATACCATGTGAATATCTGATTTCTCACTCACATGGCAAACATTTCTGTTCAGCTGCTTCGTAACCGTCTAGTAGCCACCGCACGAAAGAGCATGAGCCAGAATGCAAGCTTGAGCCAACTGTATGGTAGCATGCAGGATGCTTATCAGCAGATGCAGACACCACTCGTTTATCAGCAACAGTCTGTCAGAAGAGTATGTGTTCTATTACCCAAAACTTAGTACTTCACAACACTTTATTCAGACGATTTCTTTATATAACATTTTCTATACTTTTTTTTTACAAAAATAGGGCTTAAATGAGCTCAAGGACTCAACAGTCCAGCTAGCTTCGAGTATGGGTAACATTGATGCACCAGTTGATGAGACAGTCTTTGACATAGAGAAAGAAATTGATGATCTGCTGCCCATCGAGGTCAAGGAGCAGCGCTTGAGCAACTTGCTACAGGCTTCCATGGTGGGGGGTTGTGTCGCTGCTATGCCGCTACTCAAGAAAATCCCTACATCTGTCCTCTGGGGCTATTTTGCCTTCATGGCCATTGAGAGCTTGCCTGGTAACCAGTTTTGGGAGAGGATCTTGCTGCTCTTCACTGCTCCCAGCAGAAGATACAAGTAAGTTTATTTTATCTCAAAAGATTAGTTTGGCTTTGGCACCTCTGGTTCTGTTTTTGCATATCCACAAACTGCGCAAAGGCATGATCCAATGGGTTCAAGAAACAATATATTATGCTAAATACTGTTAATGTAGCTGCTTTGGAAGACTGAAATTCTTACCATGTACTGTAAATATTTCCCAGGGTGCTTGAAGAGTACCACACCACGTTCGTTGAAACAGTGCCATTCAAGACGATAGCCATGTTCACATTGTTCCAGACAGCGTACCTCTTGGTCTGCTTTGGAATCACATGGATTCCAATAGCTGGAGTTCTTTTCCCCCTCATGATCATGCTCCTGGTTCCAGTAAGGCAGTACATCCTCCCCAAGCTCTTCAAAGGTGCACATCTAACTGACCTAGATGCAGCTGAATACGAGGAATCACCAGCTATACCATTCAGCCTTGCTGCGGTAAGTTCAGAAAAACATCATCTATTCTGAAAAATATATGAGTCTTGCTCATCAACATGGTATTTTGGACTAGAACATTTTTAGTCATGTTGCATTTCCCTGAAACTCAAATCTCATGTATTTCAGCAAGACATCGATGTCGCATTGGGACGCACCCAGAGTGCAGAAATCCTAGATGACATGATCACGAGAAGCCGGGGTGAGATCAAACGCCTCAACAGCCCGAAGATTACCAGCTCAGGTGGCACACCTGTGGCAGAACTGAAAGGCATCCGCAGCCCATCCATTTCTGAAAAAGCATACAGCCCTCGTCTCACCGAGCTCCGGCATGAGCGCAGCCCTCTGGGAGGGAGAAGCAGCCCTAGGACGCCATCCAAATTGGGTGAAGGTTCAACTCCAAAGTGAATAAAGCAATGACGCATTGCTTAAGCTCTGCAACTACTGGCCATCAGTTATATGAGTGATTTTAAAGTGGTTTCAGTGTTAGTGTCCTTATATGAAGATGTCATCTGATGAGATTACTATTTATGTGTATCTCTAGCCTCTTTTTCTTCTTTTTTTGTGTTCTCCTGATTAAAGGTGTGCTATACCTGTAATCTCTGAACTAGCAGAATATTAGAGTAACTACTTGAAAAATCCTGTCGCTAGCATATTATCATTCTTTCATTGTTTATAATCATTAATATGTGCATGAAATTGGTGGAAATTATGCACATGCATTTCCATAGAACTCAAGCACGTATCTGGATTCTGGAATACTCTAGTTGTCACAATATTCTATTGACGATGGTGCTGAAGCATGGAACTTTAGACCCTGCTTCCCTTCTCAATTTGCACATATTTTGTTAGTTACAACAAGTGTTTTTTGCTTTTTTTTTACTTGAATTCGTAACTATAGAACTATGCTATAATAATAGTCAATCAATTGATGCAACAGATCGGTGGATGTAAATGTAGCTGAATTGGTGATGCGATTTTGAGATTACTTTTGCTTCCAAAATGTATACTGAGTGTATGATTCAAAAATCGATTCAGAATGTAAAGTTCTCCTATAGTGCTCTGTGATGATGCACGTCTCATGAAATTCAGTGAACTGAAAGTTCAGAAAAGGGGAAACGATGTAATAATATACACTTTATCTATAGATTTTTTTATAACTTGAGAGCTTCATCGAGAGATGTGCATGCAAAAAACAAGATAAGGTCAGAAATGACTGATTCAGACAGAAAGATGATTGCATAAGATACCAATCGTCTGAGTCTTCCCACTCTACTTATCTGTAGAAGCATGCCATGCTGATTCCAGCAGTAATTTGTTAGGCACTATCACAAGCTCTAACACATGGTATGACTGTATTGGAGACCTGCATCTCAATGTTATGATGCCAAGTACATCTGCTGAATTTACAGCCTTAAAATTATACTTGCTCATGTTACTATAGAGGGGAAGGATCTTTGTGGTGGAGTTTCATCTGATGCAAGGAACAAAGGGAACAACTGCAACACATGCCTGCAGTAGGTAGAAAGTGTAGAGACTTAGAGGGTGGCACCACAGCACAGCACAGCACAGTTACATTCCATCCCTCTTTCAACTGCAGCCATGATTCCAGCTTTAGAAGACACCCCTGCATTGGCCCTGAAGCATTGACCTGCTGAAGCCAACATGTCATAACCACCACAGAATTCCCTCAGGATGCAGGCTCACCTCATAGGAGCTGCTGCTGCTGGGCTAAACGACACGCCCGTTGTCAGCAATTATGGCCATTCGTCCTCCTTTTCGGCATCGATGATCTTCCTTGAGAGCCCGTGCCTTAGGACTGATGATGGAGTAGCTGATACAGGCGATGAAACTGTGCTGGAAAGATGAACAAGGAGTGATCTCAGCCACAAGGATCTGGGGAATGATGCGAGGGTTGAAGAGAAAGTAGTGGCTCGAATTCAAAGGCATGGCGGCAAAAGCACGATAATCTTCAGGTCAGGTCCATGGATGAGTCCTGGATGAAAATTCAGTGAATTGAGTGTAGCTCCAGGAACGGTGTGTTTGTTCCCCATCAATTGACCTGCAGACAGCAAGTGGTGTGCCGTGCTCTGACACGCAATTCTTGAAATGGCCCCCAGTTCCCAGGGCCACATCACATTCCCTCTCCTCCACAGCCACTGAATTCTTCATCATCCCCTTCAGTTACCTTTCATGCATCCCTCTACCATCTCTTTCGATGGGACGCCATTTGTTCTGGTCTCACTTTTCCGGATTCTAAAGAGCAATGTGACAATTTCATAGGCATGTAGATTGCGCCAGTTAAAAGATTGAGACATCAGATCCACGCACATACATCATCCAGCATCTGCAGATGTGACCATGAATTATTGGAATGCAATGTGTTGCTCCACTGAAACTTGGACGGATTTTCCGGAGGAAATGAACGGCTGAAACACTTCTGACATCTGTGGAACTGGACAACACTTCAATGAAAGAATGAAGTCAACTGCTCCCTTCAGTCTTAAGCACAATGCAAGCAATCGTAATAACAAATCATACTTCTGCCAAGGAGACATGTTCTATAATTGCAAAGCTTTCGCTGCAAAAAATGTTCCCATGCTGCAGAGCATCAGGTGGGATCCCCAAAACATAAAATACTTTCGGTGGTGAAGAAGTTCAAACGGAAAACCATTGCCACAAATATGAGCAGTGTGCACTTCAGCAAAATCATGGCTGTGGTTCAGACACAGAGATGGAACCAAGAAGCATGGTAAAACTACACCAATTATTACAGTTTATCAATCCTCGCATGAAAGCTGTGGAGTATCAATACATCAACAGCAAAAAGGAAAAGGTATTATCCATAACCACTAGGGAAGAGAGGAGTATTCTCAATCGCTGAAGATTTCTGTCTCCTGGCTATGAGTAGGATTACATTTATAAGGTATCCAAAATAATTTGCAGCCAGCATTTATGGGACAAATGCATAAAAGAAAAGACAGCCAAACTCATGGTTACTGAAAATCATTGCTGTAACAGCTCGAGTACTCAGCAATACGAAGGAAACATTTGCAGGTCCTTGTACAATAGAGGTGGAATAATTCAAAGGCAACAAAGTAAATTGTTTTTACTATTATTCATACAGCACACTGTAAGTCTAGTTTCAACTTCCGTTTTTAACCTGAAAATTTGCGTCCTGTAATCTTCTCAGACTCATACAGAATATGATTGATAAGGCCCCTTGCAGCACTGCATAGCAAGGAATAATTTCATAAGATATGCTTCGACCATGATAAAGTACTGGAAGATAAGACGGTAAAACTGAAAAGAAAATTAAAATTCTACACGGGACAGTAACTTACTCATCCTTCATTTTCTGTATCTTCTCTGGATCAGTTTCATGCATATTTTTCTTGAATTGTTGCCTCACCATGGTGACTAAAAGCTCTGTATTGTGTTTCTGGATTACAAAAGGCTGACTCAGTCTATTATTATAAAAAACTTTTCACAATTTCAAGGTGGGAGCAATGGTAAATTTTCAGACAAAAAATATGCATACCTGATGCCCAATATACTTCGCCCTCCGTAAGCATTCACGATATAACTACAGAATGAAAAAAGAGAAAATAGTAAACAGAAAAAAAAAAAGAAATGTTTTTTCCTCATCAGCTATGATTTTGTTTGTTTCATTCATGACAAATTTTCATAAAAAGTAACAAAGAACTAGGCTACAGGATGCATATTAGATGTGTCTTCTGGAAAAGCTGAAGCTAGAATTTAGGGTAACTGATCCAAATGAATGTAGCCTGATGACTGACTATTCATTAACAGTACAACAGTACAAGTTGATTTGAAAGTTGGTCGCCACACATAATACCAAGTAGAACACAGCAAAAAGCAAAAGGTGCAAAAGGAGCCTTACCCTGAGCACATTATCAGCAAGTTCTGGAGGCAAGGCTTTCTGGAGTGAAGGCATCTTCTTTAATGTTGTGCTAGTCACTAAAAAGGTATAAGAAATTGCTTTCATCAATAATATGCACATAGAGGATCGTTAGAACAAGAAAAGAAGTCAACATGTGTATTTATATATGTTTGTGGCCAACAACAATAAGAAAATGTGGCCAGAAAACTTTGGTCAAATAATGTTTAAAGCGCACAGCAAAATTGTAAAACATACTACCACAAGAGGACAGGAAAGAATATAAAACTCACCTGTTGATGTACACATACCACCTATTTTATCAACAAAATGAAAGAGCTGGGATGGAAACTTGTACTAAATGCTCACAAAGTTACATCCATCCAAAGGCCCATAGTTGCTTGTGTCAAAATATGATATTTCCAAGAGATAGCAGAGGACCCATCCAACCAATACATGGGCCTTCCATGCCCCCCTATTGATCAATGCAAGTAGACTGCAATTTCTCAACATCCATGAAGTCACCTAGGCCTCAGGTCAGGCAACTACTGAACGAAAAATAGGCAGCCCAATAGAAGGCATCATCGGACTTATTCAAGAAGATGCCTTCCTTTATTTGTGTTGATGAATTGTTAACACCAAAAATAAGCAGCTACCATGACTACATAACCAAAAAACGAATTACTGAACCAAAAAAAAAGGATTCTCAAATACGCAGGGGAGCTGCGCATCATTGCATTACGAAGAAGAGAAAAAGTACAACAGATCCCAACACCCACACACTCCACATAACTCACAATGGCATAACACAGTCCCCTACACACAAACAATTACATCACATGTGCGCTACTAATAAGAAATGAACTCTACCAAAAGGACCACACTAACTAGTTCGATGCAGCCTCAAGCTGTGCCAAGCCTCTAGCCCCCGCTAAAACCCAGAGCTTCCCTTCCTATGGGATCTGACGGAGGACTAGTTACACACTAGGGCAGCTCCCTTGAAAACACACTCAGTTCCTGTGAAGCCATAACTCCAAGTGTGATCAGAGTGTTCAGACCACCACCAGAAGCACCAAGAACGAAAGTGTTTCACCTTATGCCACCATCCACAGAAACATCATCACCAGGTTGTGGAGAGAAGTCAGGAGACCAAACTGATAGAAGTGGAGTAACCAGATCTGCCCGAGAAGACACAGCTTGTCAGAATAAGCTGAATGGTGTCATCCTTTTGATCGCACAGAGAGAAATGATTTGGGCGAGGTAAGCTCCTTTTACTGAGTCCATCCGCAGTCCAACATCTGCTGTGAGCAGCCAGCCATAAGAAGACTTGCCATTTGTTCAGGGCCTGATATTTCGAAATCAAATCCCATAGTTCAAAGGTACAACCTGTGAAGAAAGCTGTTTAAGAAGATTTGGATGAGTACTGAGTACTGACCAAACTCTCTCAACCTCCAATGGTGATCATCTTGCATACCTAGCTGAAGCTTTACTTCAGTCACCAAGTCCCAAACATCCAGGAATCCAGAGTTCTATTGGAACACAAACTAAGGCCAAAAGGGGCAGGTTTTTTCAAGAATATGCCTCTCTTCCTTAATTTGTGTCGTGTAGAATTATTGACACTGAACGCACAGGCGCACAGCAAGCATGGCTACTGAACTAAGATTCAGGAGAAAAGGGAAGGCTCTTTCAAGAATATGCCTCTCTTCCTTAATTTGTGTCAAACTGTTGACATTGACACTGAATATGCTGAACCGAAAATTCAGGAGAAAAGGGAAAGAGAGGGAAGGCAGGCAATAAAAAATAATTAATAGTGTTTTCAAGACAAGGACTGAATAGCAAAGAATAATCAAGCACAGCTGTTACTGTATCGAGGGATGCTGTTCAAATTTCCAAATAACCATTGATAGACCATGTTATACTGGGTGATCCTATGCACTTGTAAAACTAATACAAAAAGATCATCAGAAGTGGTCCATGTATTGATCAATGCAAGTCGTCCCAAGAAAAACCTGCGATCAACAACAGCAGCAGTTCCATTCCAATTCCCAAGTGAAACCGAAGTACTAGAGTCAGCACCGTAACACGAACTAACTGGCTGTGGCATTTGACATTTC

>BAC-26

ATACTTCATATATGTATTGATATTTTGATGTGACGAATAATTTTTTTTACATAGTGAAATTTGGGGGTGAATTTAGGTATCTAAACGAGTCCTCGGATAGACTGGCCTAAAAAGCAGGCCCCGGAGTACTGCTATCTGCTGGCACTGGTAGAGGCGTAGAGCAGCAGCGGCACGGGAAATGAAGAGGGTTTTCCGCCGCGCGGTTTCGGGGCGTGAGGGTGAGGCGGAAACCGTCGTCTCCTCAACGCCGGCCGTGAGGAGGGTTCCGTCGCTCTGGTGTGCCGGCGGCAGCGACCCCCTCCCCTTCGCTCGGAAGGTTCTCCGATCTCGCTTTGATTTGTTTTTTTTTTGAGAGATCACGGTTGATTTGTCTCTCTCCCTCTGCATCTGGCCTGTTTCAGTTTTGCCATGGTTTTGAAACATCAATCGAGATCGCGGCGTAGATGAGTCTTTTTTTTCTTTCTGCTTTCCTGTTACTGATTTTTGCGGTAGATTTTTGGATTTTGCATTTGCCCGATGATGACGACGATGACCCTCTCTGACCTGTTGAATGGATGGATGGATGGATGGATGGATAGATCGGATCGGATTTGAACTCCACGGGTCACTCCTTTGTGTTTGCAAGGTTGGAGCTCCCTTATGGCTAATGCAGTGTCGGCTCCTCCGTCTTGTGGTTGTGGGTGTCGAAGCCTGCTCGGGGACCGGATGCTCACTCACTCTACTGACAAGTGGGACTGCCCTTGTGGCAGGCTTATCGCCTCCTCCTCTTCTTCCTAAATCCTAAACCCTAAACCCTACACTGCATTAGCTTCTGATGGAAATCAGACGAGCTTGTCAATCTGAGGGCTATTTTGCAAAGAAAACATTTCTGCGTGTCCATTGGATCTGACTTGAAGATCTCTTGCAAAGCAAACATTTCTGTGTGTCAATTGGAATTGACTTGCGGACCTCTTCTTGAGTGTGCAAATTTCAGGGTGCTGAGGTTTACTACCTTGTTGTAAGAAATGATTTTGCTGGATGAAGTATATATCTTTGAATGAACACATCACTCATCCCTGTGCCTCATGTATATTATGGCTACTACTTGCATAGTTCTAATTCTAATTCTACTATTATAGTTGCTCGTCTTGCTTATCCACTGTTTTTGTTGAGCTTTAATTAATTAATAACCATAAAAACTGTACTACACATATTGATACTGTTACGGTTGTATCAATTTATTTTAGGCCATTTTCAAGATTCACTGTCTTGTTGCTTGATTTGCTTTTGCTCCGATGTTGATGAGACTTGTGGATTAATGCATAAATGAGCTGAAATGGTTTGTGCTGTTTTGCCATCTCCACAGTCCATATTCTCGGTGCTTAGGCATGCCGAAGTATTTGATGAGATGAAGTAGCCAAACTGACGACTGGCCCATTGTTTGCTTTTCTTCCTCGTCCTCCTTTCCTCTAGTTGTCAGTTGTCACTGCTGGCATAATCGCAGTCATGTTCTTCTACCGATCATCCCTGGGGCATGCTCCACTTCTAATGCTGGAGCCTGCTGCATTAGCCTTTGATGAAACATGGCATATCATGCCTTTCGCATGGTCTGAGGCCATTTGGCAGTGTAGACCATTGGTTAGTTTGATTTTCTTTCCCTTTTTGTGATGAACTTATCTGTCCGAGGCCTTAATGCCCTCCTTCATTCGGTTCTATCATGGAGAGCTAGTTGTCATTCTCAAGTGGAACTTGGACTTCTGTTGTAAGATGTGAATTGCAGGCATTTTGTGATCCTTTTTTTTAAAAAAAATATGTTTGTGGTTTCACCTCTCCTGTCACCTTTCTTTTTTATTTGTTTTCTTAAACTTAATATAACATATATTCATATTGCACCCATTTCCGCACCCCCATTTGTGAAGTTGTTGCTGCACCAAGTTCCTAGCTTGAAGATATGGTGACTGCCCATCTTTTAGACAAAGAATGGCAGCATTACCTTTTATAGTTTCTAAAAAAATGTGCTGCCCAATGATGAAAACCGAATATGCAAAGATGGCATATGATTTCCTCCCATCCTCCAACTCCTCCTCTTCCTTCTCCTAGCGCTCTCTCTCTCTCTCTCTCTCTCTGTCTGCCTGTGGCCCTGTTCATCTGGCTTATAATCCGTCTTATTCATGTGTGTTCACTGTTTAGAGGGAGAGTGTTGGCCCTGTTCGCTTGAACTACTTATCAGCTTCTGCAACAGCGACTGCCTATTGTCAACAAATGGCTGTCTGGAGGCGTCTTGTGCCATCAATGAATGCTGTTTCTTTGAGACCTGAGGGCAAGCTTTTTACTTATCTTACTCATATATATCCATTTTGAATTTCTTCTTTATATAAACAGATTTGAAGTTTCATCTGCCAGGTTTGCCTCTGTTAGTTTGATGTGGGGTTCTCTTGTATTCCTAAAATTATTTTAGATTGATATTGGTTTTGTACTCCCTTTTATTCCATATATGAGGAACGGGTTTGCCCAGAAGTTCATATGGTATTATAGTACATTTTGTTTCTTGTTTTGTTTCTTTGGTAGTATTTGTGTTTCATAATTCAGCTGCGGAAACCAGCAGCTTTAAGCCTGATGGTGCTCATATCATAAGGGCATCTCGTTGTAATCTGTGTTCTGTATGAGGTCCAAGTGAGGTAATTGTTACAAGTTGTAGTTCCTAAAGTGTGCAGTGCATTGTTGGTTCCTCTGTTGTGTTCATACTGAAGCCTGGTTAGGGGCAGAGAGGGATACACTTACTGACAGATGGTCACAGCCGTGCAGTGTTCCCATCTTGCTCTCCTGCCTCTTCCTAGGTTTTGTGCTTGCCCGATCATGATTACGGTGAACCCATCTCCTAATGAATGGATGGATAAATAGATCAGCTTGGATTTGAGCTCCATGGGGTGACCCTATATGTTTTTGCAAGGTTGGACCTCCTGAAATGGCTATTGCAGTGGTGGGGCTTCTCGGTGTTCTTTGCGCCATAGAGCCCGCAAAAAGTAAGCCTATGGTTTGCGGACATGCCCTCTTGCATCCCTTTGCTTGGGAGGTTTTCTGATCTCGCTTCCTGTGTTTGATTTTTTTTTGAGAGAGATCACGGTTGATTTGTCTCTCTCCCTCTGCGTCTGGCCTGTTTTAGTTTTGCCATGGTTTTGAAACATCGATCGAGATCGCGGTGTAGATGAGGTTTTTTCTGCTTTCCTGTTACTGATTTTTGTGGCAGATATTTGGATTTTGCATTTGCCCAATGATGATGGCGATGACCCTCTCTGACCTGTTGAATGGATGGATGGATAGATAGATCAGATCGGATTTGAACTCCACGGGGTCACTCCTTTGCGTTTGCAATTCGGAGCTCCCTTGAATGGCTAATGCAGTGTCGGCTCCTCCGTCCTCTAGGTGTCGAAGCATGCTCGGGGCTCGGGTCAGTTGCTCGCTCACTCTACTGACAAGTGGGACTGCCCTTGTGGCAGACTTATGGCCTCCTCTTCTCCCTCCCCCCATCTACTGGCACTGCAAGTGCAAATCCATGGGACTTGGATTGATCTCTCTTTCTCCTTGTGCTGGCAAGGAACCACAGCTAGCTAGCTTGGAGGCCGTCTTCTTCTTCTTCTTCTTCTTCTTCTTCAAGCCCTTGAATGTGCTCTGGTGCCAGCGTTGAAGCCCACTGCATTAGCTTCTGATGGAAATCAAACAAACTTGTCAATCTGAGGGCTATTTTGCAAAGAAAACATTTCTGCGTGTCCATTGGATCTGACTTGAGGATCTCTTGCAAAGCAAACATTTCTGTGTGTCAATTGGAATTGACTTGCGGACCTCTTTTTGAGTGTGCAAATTTTAAAACATTCAGGGTGCTGATGTTTACTACATTGTTGTAAGAAATGGTTTTGCTGGATGAAGTATATATCTTTGAATAAACACATCACTCATCCCTGTGCCTCATGTATATTATCGCTACTACTTGCATAGTTCTAATTCTAATTCTACTATTATAGTTGCTCGTCTTGCTTCTTGGTGCTAACACCTATTATCCACTGTTTTTGTTGAGCTTTAATGAATTAATAACCATAAAAACTGTGCTGCACATATTGATACTGTTACGGTTGTATCAATTTATTTTAGGCCATTTTCAATATTCACTGTCTTGTTGCTTGATTTGCTTTTGCTCCAATGATGATGAGACTTGTGGATTGTTGCATAAATGAGCTGAAATGGTTCTCAGCTTGGTGCGATCAACCTATGCAGTTGCAAGGTTGTAGCTCCTCAAATTACTTGTGCTGCTTTGCAATGTTCGACAAGGCGCTATTCCGCGCTCGCCTGGGCGCGCTTATGCGCTGGGCGACGGGGTACTGCCTCGCCTAGGGGGGGTAGGCGGAACTTAGGCTGCTCTCGTCGACCGTGGCGCCCAGACTTGTCGTCCCCCTTGTCCCTCTGTGCAGATCCTCGACGAAGGGGACTGCGGGCGGCGTCGAAGATGGCAAACGCGGTTGCGCGGCGGCGGAGACAGCATGCTCGGCTGAGAGGAGGCTGGCGGCGCCGGAGAAGGAAGGGAGGAGGCTGGCCGTGACGGAGAGGAAGGGAGGAGGCTGGCGGTGCCGGAGAAGGAAGGGAGGAGGCTGGCCACGACAGAGAGGAAGGGGGGAGGCTGGCCGCAGTGGAGAAGCAAGGGAGGAGTCGCGCGGCCACGGAGATGGGGAGGAGTCGCGCAGCCACGAAGATGGGAAGGAGTCGGCGAGCCGCGCAGTGGTGGCGTGCAGCTGTGGGAGGAGGGTGGATGCCTGGATGGATAAGGTCCTAGTGGATAAGGTGGGCTCTGCTTTGATGGGCCTTTCTGTTAAATAAGGTCCATATATCTCTAATTTCTTCTCTTTTTTAAGGTAATTTATAAATGTGTAAAACGCCTAGGCCCGCCTAGACGCTAGGCAATGGGTCATTGCCTAGAAACCGCCTAGCGCCTAGAAAAACATTCCTGTTTTGCCATCTCCACAGTCCATATTCTCGGTGCTTAGGCATGTCGAAGTATTTGATGAGATGAAGTAGCCAAACTGACGACTGGCCCATTGTTTGCTTTTCTTCCTCGTCCTCCTTTCCTCTAGTTGTCAGTTGTCACTGCTGGCATAATCGCAGTCATGTTCTTCTACCGATCATCCCTGGGGCATGCTCCACTTCTAATGCTGGAGCCTGCTGCATTAGCCTTTGATGAAACATGGCATATCATGCCTTTCGCATGGTCTGAGGCCATTTGGCAGTGTAGACCATTGGTTAGTTTGATTTTCTTTCCCTTTTTGTGATGAACTTATCTGTCCGAGGCCTTAATGCCCTCCTTCATTCGGTTCTATCATGGAGAGCTAGGTGTCATTCTCAAGTGGAACTTGGTCTTCTGTTTTAAGATGTGAATTGCAGGCATTTTGTGATCCTTTTTTTTAAAAAAATATGTTTGTGGTTTCACCTCTCTTGTCACCTTTCTTTTTTATTTGTTTTCTTAAACTTAATATAATCATATATTCATATTGCACACATTTTCGCACCCCCAATTTGTGAAGTTGTTGCTGCACCAAGTTCCTAGCTTGAAGATATGGTGACTGCCCATCTTTTAGACAAAGAATGGCAGCATTACCTTTTATAGTTTCTAAAAAAATGTGCTGCCCAATGATGAAAACCGAATATGCAAAGATGGCATATGATTTCCTCCCATCCTCCAACTCCTCCTCTTCCTTCTCCTAGCGCTCTCTCTCTCTCTCTCTCTCTCTGTCTGCCTGTGGCCCTGTTCATCTGGCTTATAATCCGTCTTATTCATGTGTGTTCACTGTTTAGAGGGAGAGTGTTGGCCCTGTTCGCTTGAACTACTTATCAGCTTCTGCAACAGCGACTGCCTATTGTCAACAAATGGCTGTCTGGAGGCGTCTTGTGCCATCAATGAATGCTGTTTCTTTGAGACCTGAGGGCAAGCTTTTTACTTATCTTACTCATATATATCCATTTTGAATTTCTTCTTTATATAAACAGATTTGAAGTTTCATCTGCCAGGTTTGCCTCTGTTAGTTTGATGTGGGGTTCTCTTGTATTCCTAAAATTATTTTAGATTGATATTGGTTTTGTACTCCCTTTTATTCCATATATGAGGAACGGGTTTGCCCAGAAGTTCATATGGTATTATAGTACCTTTCGTTTCTTGTTTTGTTTCTTTGGTAGTATTTGTGTTTCATAGTTCAGCTGCGGAAACCAGCAGCTTTAAGCCTGATGGTGCTCATATCATAAGGGCATCTCGTTGTAATCTGTGTTCTGTATGAGGTCCAAGTGAGGTAATTGTTACAAGTTGTAGTTCCTAAAGTGTGCAGTGCATTGTTGGTTTCTCTGTTGTGTTCATACTGAAGCCTGGTTAGGGGCAGAGAGAGATACACTCACTGACAGATGGTCACAGCCGTGCAGTGTCCCCATTTTGTGCTCCTGCCTCTTCCTGAGTTTTGTGCTTGCCCAATGACAATTACGGTGAACCCATCACCTAATGAATGGATGGATAAATAGATCAGCTTGGATTTGAGCTCCATGGGGTGACCCAATATGTTTTTGCAAGGTCGGATCTCCTGAAATGGCTATTGCAGTGGTGGGGCTTCTCGGTGTTCTTTGCGCCGTATGGCCCTCAAAAAGTAAGCCTATTTGTGGGTTTGCGGACATGCCCGCTCGCATCCCTAGGGAATTGGATTACACTCCTGCTTCTGCTTTGTGCCAGCACTGAAGCCCTCTGGGTCACCTTCTGATGGAGATCAAATCAGGTTTGCATGCGTTGTGATTGTCTACGTTGTACTGTGAAATTCGCCAAAAAAAAAATCTGAGGGCTATTTGATAACATGAATATTTCAGTGTTTTGATTGGAAATGATTTGACGTCCTCTTATTGGAAATGATTTGATGTCCTCTTATTTGTGTTAACAATCTCTTAAACAATGAGGCTCTTCCTTTTTTAAAAGCAAGCATTCAGGGTTTTGATGTTTTGCGATCCCATTTTGTGATTATTGTTTTTGTTTTGGCCTGATGATGATGAGACTCTGGATGGATGGATAAATGTACATGGATTTGAGCTTGCTGGGCAAGGGCAGCCTATGCATTTACAAAATTGTAGTTTCTGAAATGGCTTGTTCTGTGTTGGCTTCTCCATGATCTGGGCGCTGAAGCATGCTGAGTTATCGGATGACCTGATATGGTGAAATTGTCTGACACTGACACATGCTCAACCAAGTGGTTGGCACGTTGTCTCTGCCCCCCTTCCCTCTCTGTCTCTTTGTGCCACTGCTGGCATGCTCTTGTCGTCGCCGTCGTCCTCGTCCTCGTCATCTTCTATTCGTCTGATCTCTCAGGACATGCTCCAGTGCCAGTGTTGGATCCCGCAGCATTAGCCTTTGATGAAACATTGGGTTTCATGCTTGTCACATAATCTGACTATCTGAGGGCTATTTAGTAATCTAAACCATTGATGATTTGGTATTTTTGTTTCTTTCCCTTTTTATGATGTTTTCATCTACCTGAGTAAAGATCTTCCTTTGGTCTGGTTTATTGTGGAAACCTAGATGCCATTCTCCAGTGGAAGTTGGTCATCTGTTGTAAGATGTAACTTGGTAGGTGTTTTGTGATCGATTTTCTTTCCATGTGGTCGTGGTTATGCCCCTCTTCAATGGAGCTTCCCCTTTTCTTATGTTGCTTTGTAAATCTTCATTGTGCAAAATTAACTCCGTATTTACTCTCCCATTTGTGGAGTCGTCAGTTGCGCTTTCTAATTTGAAGAAATTGTGATTTCCATCTTTTGGCTGGTCAATGGCAGATCGGCAATGCTGCCTTTTAAAAAAAAGTGTTGCTCAATGATGAAAACTAACACAATAGGAAACGCGCGCACTGCCCGCCACCCCCCCACAAAAATCCGTCCTCCCAACCTGGCAGTGCAGCAGCAGCTGAGCAGTAATTTCATACTGAATTTCACCACTCACCATGCTGAATGCCATACTTGTGAGATCTGAGGGCATGCTGTTTCTGTTTCTTATTGTATATATTCATTTTGAGTTTCTTCTGTTAAGGAAATATATTTGCTAGTGTATCTTTGATGGAAGGTTCTCTTGCATTCCTGCCTCTTTAATAACATTTATATGAATGTGTAGATAGACCAAGTCCTGTGGGTTCGCCCTTTGCATTTGCATGGCTGGAGCTCCTGAACTGGCTAGTGCCTCTCTTGGCTTCTCCATGGCCTGCGAGCTGAAGCATGATGAAGTGCTGAACTTGCTGACAGGTAGGGTGGCCTATTATTGTCTCTACTTTATGTCTTTGGTGGCCTCCTCCATCCCGCTTGTTGCGGTGATCACTGCAGTTGTCATTGTCCTGATCTACAGCTAATGTTACCTCATGAGTCGAGGCCTGCTGCGCCTTTGGGCTTTGATGTAACATGATGGCATATGCCATCACATAGTGCGTGAGGGCTATTTGGTAATATATACCTCCTGCATTTCAAATCAGTAGCCTTCTTTAGTCAGGATTCTTAGGAAGAGCTGATTGGTATGTTGCTTTCCTACTGGAAGTTGGTTCTCTTTTTGTAAAATGTGATTTTGCATTTTGTGTTCAAATTTATTGTTATATGCCTATGTTTCCCCCTCATCTCTTATCAGAGTCCCTTTTTTTCGGTGTTGCTTTGTAAAATTTCATATTATATTGTAAGTAACCATTTTTCACTCCCAGTTGTGACCCTTTTAGCTGCATCAGGTTTCTAGTGCGATAAATTGTGAATGCTGCACAGACATGACGAATGTTTTTGAGCAAACGTTATATTGTTGTGCCCAAGTTGTTGTCCTTGCTTAGCTGTTTCCTTTTTCTTGCTGTGAAATTTTGTATAAAAAGAATTATAACCATGAAATTTCACAGCTCTGGCATCAGTTTTTGCAACCAAACAAGGCTTGGAGCTTTCTCTTGGATGGTGGATGATAGAAGCTCTGTTTCTTCCTAGAAACAACTGCCCAGTGGTGGAAACAGCAGCAGCCACAAGAACAGCACCAATCCTTTTCAGTACTGCTCAGTTTTACTGAGGGCGTGCCCAACGCTCTATGGTGGCGTGTGGTTTCGTGTACCACGTAGGCTCGGATGCAAAAAAAAAGTGAGCAGACAGTGGCTTGGCAGGCAGTCAGATTAGCCTGTAGAAGAACCCAACGCCTCACCTGAGGAATAGCAGAAGCGTCTACCGATTTCCGCGTTTGTGAGCTTGTGGCAAAGCATGAAGGTACAGTGGACCACACCAACACCTCTCACATAACAGGGTCAGGAGATGGCAGGGTAGTGGGGCCATGAAAAAATTGGTAGATGTACCCTGTGTTGGAAAGAAGTTGGCTTCGTTACATTCACTCCTACGTGTATCATTGCTTTTTGTTGGAGTTATTAACAGTGTATGCCTGCTTTGGGGGTGCTCTGATAAAGGGCCAGTACATGTGTATGCCTTGTGCCATCACTGAATGCTGCATCTACAAGGTCTGAGGGCATAGTATTTTATTCCCACCTAGATAAGCCCAAACCTGTTCCTTATTCATTCATATGTTTTTGTTTTCTTTATCTTTCCACGTCTATGACAAATTGTGATATTATTTTGAATTAGTTTCTTCAAAGAACACAGATTATTCGTACAGTCTTTGGCAGATGACTCATTTGCTTTATTTCGTGGTTTTTATATGCACTTGAAAGTTGGCAATGTTTTTTTGTTAAGTTCTTGTGCATGTGAGCATATAGTTGGACTTGGACTACACATGAGAAGCGTACCTTTGTCCATATTGTTCTTCCTATTTCTCCCATCCAGCATGGAATCTATCCATTGACCATGTGGCATTTCGACCTCCAAATACTGCTGGCACAAAAGAAGTATTTCATAGTGTTCTTTTTTTTTGTTTAATAAACTGTCATGACTGTTTTCTTTCCATGGCAAGTACATAAGCAGTAATACTTTTTCTGGGATTCTTCTTGAATTCCATGACCCTTATTTTAACTGATTCTTAATCGCATCCATAGGTGGTAACACTGTCTTCTTATTGTGTCCCAAGGCTCAGTATCTGCTGTGCAAAATCATGAAGCTGAGTGTGGATTGTCATGCAATAGATAATACAAAATCTGTGGGACAAGGTTTCATGATATTTCCTATAATTTTCAGTGTTTTCCCCTTTTTTTCAGGGGTGTGGGTCCTGCATCCATCTCATGTCATATTGTCTCCACCATGGTTTTTCGTGCAGCATACTTGAATTGTAGCATATTTATAAACTTCTCATATTTTGAAACTGTCATTTATGGCTATCATTCAAAGTTTCAAACTGGGTTATTACATACTCCCTCTTTGAAATTACAAGGCTGTTTTTTTCCATAGAGCTTGTTAATTAATACACAATTTCTGTGAGAATTGATGTCATTAGATTTGTATAAAAAAATACTTATCATTATAATTCTGTACCACATAAATGAAATACTTCATTTGTGGAGTAATCGTCCGTCAATGGCGTGTCCAAATGAAACGAAATAGGCCTTGTAAAAAGAATGGTTGGAGTAACACACAACTACACAAGTTTGTGTGCAGATCCAACCTATTTTGCTATTGGTCGTCACAGTATTTCTTATGTAAATATTTTGCTTTGCATCAATATCATTCAAATTTGTTTTTGGTCCTCTTCTGAGAGCATTTGTAAATAATCAATTTGTGACATTTGTATTTGATCTGTACTATCTGTTTCCTCTCTTACCATTGTTTCTGAGAAATGGGTACATCTATGATTATTTCGTTACTATGTTCATATGGCTAATCCTGGGTTTGCACCATGAAAATGGTACTACATTCCCAAATGTCGGTTGTTATAATTTTTTGCGCTGAGCCAATTCTCATACTTTGACCAAGTCTTCTAATTTATTATGAAAAATATATTTAGATAAGTTAAAGTTTATAAATACTTTATCTGTTTCAATTATAAGTCGTTTTAACTTATTTGGTACATCTACTTTGCTATGCATCTAAATATAATAATATAA

>BAC-11

GTTTATAAGCAAGTTCTATAATTAGTTTTTTTATTAACATCCGAACACCCCTTCGCATAAAATATCCACACCCCGAAGCTTTAGCCCACTGTCTTCTTCCTCCTTCGACCTCAGTCCTTACTTGCCAGATTGTCCGTGCCACGTTGCCACTGCGCAGCCAATCAGACGAGAAGTCGCTCCGAGACGAAACTGTCCCCTCCCCGCTCCCCTCAATTCGCTACTCGCTAGTCAACTCAAACCCGGGGGTCCGAGCCCGTCAGGCCATCGCGATCGCGAAATGGGTGGCGCTAGCGCCGGCGGGGCGACGGAGGCGGCGTCCTCGCCTCTCCTCACGCCCCGCTCCGCTCCACGCCCCGCGGTGGGGGCGGAGGTGCGGCGGCAGGTGGGGCTCGCGGCGCCGCTGGTGGCGTGCAGCCTGCTGCAGTACAGCCTGCAGGTGGTCTCAGTCATGTTCGCCGGACACCTAGGGGAGCTCTCCCTCTCCGGAGCCTCCGTCGCCGCCTCCTTCGCCAACGTCACCGGCTTCAGCGTCCTGGTACGTGCAGTGGTAACCTCTCTTCACTCAGCCATTTGTGCAACTCGGGTCTCGGTATGTGCTACTGCTTGTGCATAAGTCTAAAAAGAGCAGGGAGGGGGATGTGAAATTGTGAATCGGAACATGTACACTCTAGGAGCTAGTTGTTTAAGGAAATTATAGCCAAAATTAGTACGGATTATGTAAATGCTTGCGCATTACTCCAATTAGTTTCTCACATGCTTCATAATTATACCTACTACTAGTAGAACTCTAGAAGTAAGAGCGGCATATGAGTTTTGACTTATTGCACAGTGGTCAGTGCTATACTGCGTGATGCCTACTGTGCTACCAGTAAATTAATTGGAATTCATGCTCCTCTTTTCCATGTCAAGCTGGGTATGGGAAGCGCATTGGATACCTTTTGTGGACAATCACACGGAGCAAGACAATATGACATGCTGGGGACACACACGCAAAGGGCGATAATTGTTCTTATGCTTACGGGTGTTCCTCTGGCCTTTGTTTTGGCCTTCGCTGGCCAAATCCTTATCGCCCTCGGTCAAAATCCAGAGATATCATTTGAAGCTGGACTGTATGCTCAGTGGTTGATTCCTGGCCTTTTCGCATATGGTTTGCTTCAGTGCCTTACCAGATTCCTGCAGACCCAAAATATTGTCCAGATATTGGTAGCTTGCTCTGGACTTACTTTGCTACTTCATGTTATGCTATGCTGGTTGCTGGTTCAATGTTTTGGCCTTGGCCACAAAGGCGCAGCTCTGGCAACCTCAATATCTTACTGGTTCAATGTGGCATTGCTAGCTGTGTATGTGAAAGTCTCTGAAGATGGCAGAAGAAGTTGGCACGGATGGTCAAGGGAGGCACTGAAGTTAAAGGATGCCAAAGTATATCTAAAGCTAGCAATTCCATCTACCTTTATGACCTGGTAAGCCTGATATGGGGCACCATGTGAAAAACTTGTATTTCAATAAGTCTTGAATGTTTAACTTATGGTTTGGCAAAGAAGTAATGTGTACCTTTACCTACTTTCTGCATGGAACTGGTTATTTGACTTGCAAGATAGCTCAGCTTGGAGTATTGGGCATTTGAGATGGTGGTTCTCCTAGCAGGATTTCTTCCAGATCCAAAACTGGAAACTTCAATTTTATCAGTCAGGTAAGCAATTGAAACTTGAAATGCCTCCTCCCTTTCAGTAATCGGAAATTCTATTAAACTGATCAATGTTTTAGATCTGATGTTGTTCTGTAACATGGCTGATCACTCTGTGGTTGCATTGGTTTCCTTTCTCATGTCTTGAGATAGCCATTTCTTGTGCATCAACCATGCATCAGTAATGGTGCTTTCAGTGCTTGATGTGAAGTAACTTTGACTCTTGCAGCCTAAACACAATGTGGATGGTCTATACAATTCCAAGTGGCCTCAGCAGTGCAATAAGGTCAGAGGAGCATAAATTCGTAGTGCCCACTCCTTTTTGTTCTGTGATAAAAGAAACTTGTGGTTAACTAATTTTGGCCCATGATTCTTTGTTTCTTTGCAGTATTAGAGTGTCCAATGAACTAGGTGCTGGGAACCCACATGCAGCACGCCTATCAGTTTATGTTTCAGGAATCATGTGCCTAGCTGAGGGCCTTTTTGTAGCTATCATCACAGTATTAGTGCGAGATGTCTGGGGTTATTTGTACAGCAATGAAGATGATGTAGTGAAGCATGTATCGATTATGATGCCAATTCTTGCTACTTCTGACTTCATGGATGGAATACAGTGCACACTATCAGGTTTGGGATAATTGCATTGTCTAGTATGCAGCATTTGCATCATATCGGACTTCTGCTCCATGTTGGGAAATTCATATGCAGATACTGTAATCAGAATATTATTATTGTAAAGTTGCTTAGCTTTTATTTTGTTTGATCACTTGGTCACTTGGTCTTGGTGTTGGTTTGCTTGCCTCATTATGATGCCATCAATCCATGTAGATTAATTGTCTTTGATATGCCGCCTGAGCAGGTTAATGTAAAAATGATCACATATCCAACGCGCTCACTGCTCTCATAAGTCATAAGAGAGGGAAAAAATTCGATATGTTTGGTATTGCATGCTACAAGTAAGAGGGTTTCACTAGTAAGCCATACAGTCCAAATTAGGATCATTGCTGTTCCAAACCTATTGTTTTTTCTAGGTATCATATATCCCTTTACCAAAGAAGAGCGGCGCAAGTATCATGTAAAAAAAAACCTATTGTAATTCCTATTTTCCCATGGTAATCCCGGGATTACCTCTGAACCTTGTATTTTCAAAAACTCTACTTCCTCTTAATGAAACACGTACCTCATGGTATGGTCGAGAAAAAAGAAGAGCGGCACAAGTATTATGTACAAGAGTGGTAGTTCCTGAATCAGACTTGTCAGTGATTCTATTGTGACACTCCACAATCATAATATCAGTAATCCTTTGTCTAACTGATCCAGGCTGACATATGTTCATTATCTGCCTGTTGATTTTAAGGCGCAGCTCGAGGATGTGGCTGGCAGAAAGTATGCTCGGTTATCAACCTGTTTGCTTACTATGCTATTGGTCTCCCTTCAGCTGTTACTTTTGCATTTATTCTGAAGATTGGTGGTAAGGTAGGTTTGACTGTTTTACTTTTATGTAATTATGATAAGATATATATTGATATGTGTTCCCTTCATGTGCCAGGGCCTTTGGTTAGGAATCATATGTGCGATGGCAGCGCAAATATTTGCTTTGGTTGTGATGATGCTTCGAACCAACTGGAATGAAGAGGTACACCCTAAACTTCTTATGCTACAATCAACGGAATGCATGCTCTCTGTTTCTACTGATGGAATTGGATCTTAGTTGTGAGAGGGAACAGCCCGGGCATGGGACTGTACACATGGAGCAGGTTTGCATCAATGGGGAACAGAATGCAAAATTAATCGCTATAGATTGCTGGGATTTCTTCTCTCTAAGAAATAATATTGCAATTATTCTTTCTCTAAGCTACTTTCAAAAACTAATCATGGCAACCATTAGTCATCTGTATATCACCTTGGCATGATAAGGAGGCCTATTTAGAACAAATTGAATCTATATTGCAATATGCATGTGGCACCTCAGTTCTAAAATTTGCTTGAAGCATTGCCCTCACTCCACTGACGATAGGCACAGGCCTACTCGTGCATAGCACTTTGCTTCAATTTTCATCATCACTAGTTTAACCTGGAGTTAACTACATTTGTCCAAGAGACATATACTAGTATGATTCACTTTAAATTCCTGGGACATGCGACTAACTACCACTACTACACGTGGGCGGCCACCTCTAATCAAAGTGGGTATTATAATACCCTATAAGGCTATAAGGCGTCAGTTACCAATCATGCTTTCCTGTGCCGTGTTGATGTGAATGCTGTATATCACTATGTTCTCTAATTGCTAGAACTAACCACTCTTTCGCATCTTTTACTGTTCTAGGCTGAAAAGGCCCGAGCTAGAGTTCAGTGTTCAGATGGCAGCAGTACATTGATCTGAATGTCATCAGTTGCGATTATACCTCCCTTAAAGGTAGCTGTTTTCTTTTATATAATTGATACTTTTCAGCATTTGAGAATTTTTTCTGTATAGTCAATACGGTGAAAACACTTCAACATGAAATATTACATTGCTAGCCAAGCAGCGTGACTAACTCATGTTACACATCTATTTAGCATAAGTTCACAATACGCAGTTCATAAAAGGTGTGCACTGCAGAGGAACTTTCCAAACAAAGTTTGCCAATTTTGGCTTTGGTGGGAGTGGATGGAAGAAAATCCGGGAAAACTTACTTTCAAAAAAAAATCCGGGAAAACAGAAATTGAAGTCATAAAAACATGTAAAATATCACTGCTGCAAGGCTTTTATTTGTTTCCCCCCTCTTTCCCAGATTGTATAGTTATGGTCCACGATTATTTAAAGGTAGACAGTACACATCATTAATTTACTATTATGATGCATTGTTTTACCTAACCCATTGTCCTTTCCTTGAGGAGGTTACTCTGCTAGTGTATAGCTTCGATCTTAAAGAAAGCATTCAAGCATCTGATGTAACACTCCATCATGCTTTAGTTGTTGATGCGGATCTCTCGGTTATACATACAGAATGAAAAGGAGAAATATTCAGGCAAAAAAGGAGAAGGTGCACCATTTCACTATAATATGTCCACATTCTTTGGGAGTTATATTGTGAACTACTGTTTATTTTAGTAAAAGGTGGCATTTTCATGTTTCAACAACGCACTGAAATCGCCCACACAGTGTATTCCCACGCATTTTACCGAACCAAAACAAGAATAAGAAAAGGATGTGTTACCTGACCAAAGAATTTTGAAGAGAGTTCTTGAAACTTCACTGTGTTGAGTGATAATGATAGGGCCACAGTACTGGTATGAAGTACTAGTATGAACAATGTAACCTTAAATATATATTTGTCTCACACGTTCCCCCTGCTGCACAGAAGTCTCCAGCACGTAAAGCAGTATCCAAGTCTGAGCTTCCAATGGATAGCATCATTACCGACCATGGACTAGCATACAGGACCAAAAACAACGTGCACAAATTACCTGCCGTAATTGATGGCCTCCAAAAGCTAATACTTAGGTCTATCTCAAAATTTTGTCAAAGGGTGAAGCTGACAAAAGCAAAGACAATGATCTCTGACCAAGAAAGGGTGAATTTGTATTTTTAACGACTGCATTCATTTTCTGTTGAACAGCTGCATTTCGTTCTGAGGACCATTGTGCTTTTGGCCAAACAGACGACGATGCTTTTGCTGATGTTCTCCACGGCTGTCATTTATTCATCCTAATCCGTAACCAAGAATTTCAGATTCAATTTGTAGTGAAATAATGAAGCTCCAGCCTTTATGCATAAAAAGTTAGATCATACATTATAGCTGAAACTCAATTACTTGCTAATATTAGCCCCTTTTTTATTCTTGAATGTACAGGTGACTGGAATAGTTGACCTTGTAGTTGTAGGTCTATATGAGCCCTTGGGTTCGAGGCACTAGGCAGCAGCTCCAAGCTTGACGCCCTGGATCTGGTAGGACTGAACTATCATCCTAAAAGCAGGGTACTTGGCTTACATGTGATGCTCTTGGAGTACGTTTCTTAGTGGCTTCCGCAGCTCAATGATGGCACCGTGAAAGGCGATGAAGCAACAGAAGTTTGGTAGCTCTGGAAGTGGAGGGTGTCGTGGACGCTGAGCTGTTTCCAGATCTCAAATATTCCACCATGCATCTGAAATATATTTACTACTCCATCCATTCCAAATTATGACTCATTTTGACTTTTCTAAATGCACAGTTTTTATAATATATCTTAGACATAGAGCATATCTTTTTGCAATATGCTTGGAAATGCCAAAAGGATGGAGCGATGAAGTGTGTAAACTTTCCTGCGATGCCTGAGGAGTTCCATCGCCAGGGATGATGCTTCAGGATGCTGTCGGTTGCGTGGGCCTTTTCCGGAGCTGATCGATGGCATGCCATCTTATCTGATGGTCACATTTGTGTAGATAATAATCTCTGGTTGGCTCAGAGAGAAAGGAAATGACTCCCCTTTCGGTTGGTATACGGTCTTGTCAACTGTCGTACACGGAAGCACGGTGCAGGGAGTCTCCACCCAATTTTTTTTCCTCTTTTCCGTGTGGGAAACAGTTGAGCAACCACTGCATCACCTGCACCTTGTTGTGTCGTCCATGGCGGTTTGTTTAAGACCTCTTCCTTGGTCCGTAAACGGTAGGCTAGCTAGGGTCGGCCATGTTTAGCAAATATAGACAAGCAGGTTTTACTCGTTGCAAACTGGCTTCTCGGAGAAACAGAGGAAGACAGGGGGGTTGGTGGTGCGTTAAGCTAACGACTGTGCTGGCGAGGACTTGAAGCATCATGCTGCTTTCAGATAAAGCGATGGCCTCCTAAACATTTTTTTTTTGGAAAGATCCTCCTGAACATGTCCTTGGTGAATGTCTGTCATATGCCTCCGTTGCTGAGGATGAGGGGGAACAGGACATGACATAACATTGTGGCCACATTGGCACATCTGCAAGCCTAGGTACTGGGAATCTTGAAAGGAAAAATTTGCCTGGCAACGGCAGTCATGATCACATGGCACCACACCCTACATGGGCAGATGGTGGGCTATATGGACAAATAATTTACCTTTAGCTCGTGTTGGCCTTTACCGACAACAATCAATCATTTACAGTGTGTGTTTGTGTAAATTAACCTTGGACACGACCAGACCTTGTTTTTGTTCCTCTCCTCCTTTACCCAGAGAAAAAAAAGAAGAAAAAAAACAATGAAAGAAATGAATCTTTGCCACAAGAGTGGAGAGAGGGAGAGAGAAAAGGAGAAGAGAAAACAAGTGGAAGTATAGAACAAAAATCCAAAAAAGACATGGTGTGAACTGTGAAGTGAGCCTGCCTACTATCTACCAACCAGCTGACTGACGCTGAAGCTGAAGCTGCAGGCTGCAGCAGCAGAGCAGCTAGCTCCAATGCAATGTAATGCAACAAGAAGCAAGAAGCCAGCCATGAAACCTGCAGCAGCGAAAGGGGTCATCAGTCATGGTGACGGCAAGAGATCTCGCGCCTGCTCCAGCGTCCTGGTGAATCTGGTGAGGTTGAGCCTGACGTTCTGCTTGTCGAGGTACACCGTCTTGATGGCGTCCCATCCCTTCTTGTGCACGTCGTAGGGGTGCTTGAGGTAGAAGTGGTCCTTCGGGTAGAGGTCCCTGAGCGAGCTCTCCTCCAGCGACACGTTGTACTCCATGTAGGTGACGTCCATGTCCCTGGCGGGGTCCTTGAAGGTGACCCGGGTGAGCCACTCCAGCCCGCCGAACGGCACCACCTGCACCAGCACGGCGCGGCTGGGCAGGAACACCATGTTGGTGAGCCCGGCGCCGTGCACGCCCATCATGACGTCCGCCGAGTTCACCAGCCGCGCGAAGTTGGGCATGTCCGTGTGGTTGTCCGGCTCGGCGATCCGCACGTCGAACTTCGCCGCCGCCGCGGCTAGCGCCATGGCGCGCTCGTTGACGAACCGGCGCGAGCTCTTGCGCGAGATGATGAGGAGCCGGGGCCGGTCCTTGCGCCGGGGCGCGCCCGACCGCGACGCCACGGCGCGCTCCAGCCGGAACGCGCGGCGGAGCACGCGCTTGAAGTCGGCCACCGTCTCACCTCCCGGCGTCCGCGAGGCGTCGATGCCCATGGCGCGGTGGAAGGTGGAGCCGATGACGATCCGCGGGAAGCAGTGCACCTCCTGGTCGTTGTTCACGTCGATGACGTCGTAGCGGGAGAGCTGGCGGAAGAGCGGCTTGAACTTGTCGGCCCACCAGTCCTTGATGTCGGCGAGCAGGAACTGGACCTCCCCGCCGAAGTGGTGCGTGGACGCGAACAGCGGCACCAGCACGTCGGCGTAGTCGTGGTAGAGGTTGCCCGCGAACCCGCCGCTGGAGAAGAGGAACCCCGGGACGGAGTGGTTCCGCGTGCAGAGCGGCGGGACGGTGGTGTGGTTGGAGCCACCGAAGGGGACGAGCGTGAACTCGCGGACGTCGTCCATGGCCACCGGGTCCTGCAGCCGCGCGTACGGCTTGGTTCTCCACTCCCGGGACAGAGGATTGATGTAGATCTTGGAGTGGTTGCCGTCCACGCGGATGTCGCCCACCGCGGCGCACCGCTCCGAGCGCTTGCTGGTGTTGTAGCAGGTCGGGCGGGACGGGTCGAAGTCCTCCTCGCCGATGGGGCCCGAGAGCTTGTACCGCGTGCTGTCGTAGTCCTCGTCCACCTCCACCACCTCCAGCTCCTCCGCCGCCTTGGCTGCAGTGGAATGGATGGAATCAGTGAGTGGGTGGTATCGGTCAGGTAGTTAGGTTGGACTTGGAGGAGGAGGCAAGAGGAGGAATTGAAGGAGGAGGAGGGGAGGCGGCGACGTACGGAAGGGGTTGGAGCAGTATCGCGCCTTGATGTAGGTGAGGACGCAGAGGGAGACGAGCATGGTGACGACCATGGCGGCGTTGCCCATCCGGCGCGGCTCCTGCCGGCCCCTCGCCTGCTTCATGCTCATGGCCACCCCGGCACCCCTGCCGCCTCGCTCCGGCCGCTGGATCTGGAAGCGAGCGACGCGAGGCGAGCTGCAGTAGACGATCGAGAGGGTATTAATGGAAGCAAGGCCAGGGGTTCCCGGGGTTGGGTTCGCTCGCGCCCGTCGATGGCGACCCCGCGGCGAGCAACCAGAAACGAGTTGGTGGGTGGTTGACGCCCGATCCCCCTCCTCGCTCCGCTCGTGGGTGGGGGGTTGCGCGGCACGGCCACGGCAACCACTACGGCAGCTACGCTACAGCCGACGCGCTGGCCCCGCCGGCCGGGCCCCAGCGCAGCGTACCAACCCCGCGCTCCACCGATCCATGAATCCATCCATCGGCGAAGGAACGGTAAAGGCTTCCAGAATTCTTGTCCGACAGTGCGGTGGGTTCGTTAAACCTGGCGCTGGTGGTGGTGGGATCGATGGATGCCGGGCACGGCAGGCAGTGCGGAGCGGAGAGCTTTCGACGCACAGTAAAGCCAAGTACTGAACCTGGCGCTGTGCGCGGCGGCCGGCGGCGACCCTGGCAGCGCGCCGTGTGCCCGTGTGTGTGCGGACAGCCAGCGACTCGGCTGTTCACGGTCATGCACGTCGCGTCACCAGCGCTGTTTGGGCCGGACGGACCTTGCAGGCAGGCATGGTGTCCTTTCGCAAAGCTTCCGTACGCGCGCGCACGTGTTGGACTTGGCATTGGCGCGTTGCCTTGCAAACGCCGAAACCGGACGGGACGGGACGGGATGCAAGCACCAAGCAGGGTAATTTATCAAGACCATCCACATCTCGTGCAGGCTTGTAGTACAAACAACGCTCAATCCCAATCCACACAAGAGACGACTGAATACGAAGCGTAACAGGAATGCAGCTCCCGTTCACTCCCACTTTATCCTTTTAACCTTTATCCAGTGGAGCAGCTGCCATGTCATTCTCAGTCTTAGTTTAACACGGCTTCAATACTTTTGCGTGGTGTCGAGTCGGAGCTAGAACCTATGAAGCTAAAAATAATAATTTCATTGCTGGTTTATTATGATTGAGAGGAAAAAAAAAACTACCCTTGCTGGCCGCTTGCCAGGCAAGCTTGCTCGTCCAAGCAATAAAATACTTGCTAGCGCAGGTGAGCCAAATCAGCTCTCCTGCCCGAGCAAAGTTGGCTCGCCCGCTCGTTAGCCGACTCCACCGCCATCGAATTAGACTCTCCGGCCATCGATTCACCTCTGCCGGCATTGAATCAGACTCCCCTATCAGTGAATCAGCCTTCACTATCATCGAGTAGGACTTTGTTGTCATCAAAATGGTCTCTCCCAATGTTGAATCGCCCTCCACCATCGTCGAATCGAACTCCACAACAACATGATTGGTGTCCCTCACTGTTGGTTCAACGTTCCGGCTTGGTGTGGAATCGATCTCTAGCGCCGCCTGTTCGGTCTCCACCAATTTGAAAGATAGCTTTGCGAGCAAGCTAGACTAGGCGAGGTGAGGGGAGGAGCGAGCTGGGTGAAGCGAGCAGCGGCAAAGGTACCAAACACGCCCTACGTTTCGTGTTCTGAGTTCTAGAAACACCCGCAAGCCACATCTAGATCTAGACTCTAGATGCTCCCCAAATCGATGTCATCTCATCTCATCAAATCAACTGGCCACAGAGACAAAGAACCACCTGCTCTGCTTTTAGAGGCATGAACCACTGGCTGTTCGGTCACTCATCCAGTTTTCAACTGAATCACTGAAAGATATTGTAACGCACACCAAGGACAGAGGCGTAGATCCATAGGGGAAGCGAAGGTCCAAACGTCGCTTTCACGTTCCTGCTGCCCACAACGCTGGAATGGAATTACTCTTCCCTCTGCAGCTGAAGTTTACTAGCATTTTATAAACTTGGATCATGGTGTTATATATCCTGATCCGTAACTCTTGAATAGTCCTTAACTTGTACGGTAATGTCATATGGGTCTCAAATTCTCGATGTTTGATATTTAAGTTATTAAAGTTGTGTGATGGTGTTATCTAAGTCCCTAAAATTGCTCGTCAACACACGATTATCATGACATTCCAACGCCATGTATGTGTATAAGGTGCAAGTTTGCAGACCCATATGACATCGTCATACAAGCTAGTTCAGCGCCTAAATATCAAAAATTGAGAGTTGAGAATCAGAGTGACATCGTCGTATAGGTTAGGGGACAAATATCAAAATTTTAGAGTTAGGGACCTTGATGATACCGTCATACAAGTTTAGGGATTGCTCGTGAATTTTACTCCAAAATATGAGCTAAAGCTATTGTCAACATCACACTTTGACTGTTAGTTTCTCCTAGAATATATAATTTATGTTAATAAAATGTTTGAT

>BAC-32

ATATGGATCTCATTCTTACCAGGATTATGGCTTTTGTATACTAAAAATATCGAAATAGTTGATTTTGGATAGATAGTTTGTTTCTACAATATTTGGATCCCATTCTCAGTTTTTTCACCCTATTCTTATTTTTCTCAGGAGATAAAACAGGACCTTACTTAGCCGTCACGTAGGTAAAGATTAGTTAATTTGAATGGGTACTTTTTTAAAAATCCAGGCCCCACGCGGCCACACATCCGGCTCATCTTGCGATCAATCCAGTCCGTCCAATCCTTGCCATCCGAACACCCCGCCGCCGCAACCCTAGATTGCGCCTTCCTCTCTGTCCCCTCCTCCGCTGCGCCGCCATCACATAAAGTCCGCCGCCCTCTCCCTCCGTAAACCCCTCTCTCTTCCTCCCGCGGCCTGCTCCTGCGATCTGTCCAGCAGATCTCAGGTTCCGATCGGCCGTGAACCAGCGGCAGCTGTCGGCATGGCGCTCGTGAGTTCCATCTCCTTTCGATCTTTTCTTTTTTTTTATCGCTCTTTCAGAAGTGCTGTGTAGCTCATCGCTTGTCTGACTGTCCGTGAGGAAGTTTTGGTATGCTCGGCAGTTTGGTTTGTAGGGTTTTGTGATTCCTTCCGTGTAGTTTAACTTGATCTGATTGGTGATGCGCGCTGCTGAGCTGTGGTTTCTGTTGGATGCGATTTTCGCGAGTTATGGCCTCATAGGTATCGGAACTGTAGTCTGTAGATGTGATCGGAGCGCGTTAAGTTGTTTAGATCAGCAGCTTTGCTTTGGGCAGGCCTTTATGTTGGGTAACTTTGGACTGCTGGTTCGTTCCAATTTCAACATCACCCGAACTTTGGCCTTTGGAAATAGATCGCCTGTTGTTATCCCTTGTTCACACCCGTCGGTTCATACTGAGTCTGATCACAGTGCATGTGATGTCACTTAGAGTTCCTGGCTTACTAGTAGAATGGCACTGGAAAAGACCTTTGACCTTTGATACCCTGGTGACCATGTAGTTTTTACAAGGGAATTGCTCTATGGTCAATGGCATTTACCTCGTGTATTTGAGCCATTGGATTCATTTAGGTGCTATCATCTCAAGTGATTTTAACAATCATCTCAAGCGCTTTTAAGTTTTTGACCCTGATCCTAAGTTGTAAGTTGAAGAAAATGTTTGTACATGGAGCAATGCTCTTTTGGTGTTTGAAAGGAAGATGAGGAATTGAAGTTGGGAGGTCGAGCATGACTATGTTATAATTTGTTTTTATAAGTTAGCAATTTAGGCAATTCAATTGTAGTAATGTTTTGGCTACATTAATGGTACTGTTTGCGTTATTAAAGCATCTTTATTGGAATCTGTGTGAAAATGTTCATTTGTGCCTTGTTGGGAAACAGACATGTCATCAGCTACTTCATATTATGATTTGTGAATGCTCAGTCCGGGATGTTGCTGCATTTGCTTGTGTATTTTTTTCCCAAATAGTGAAATACATGTGTTCTGTGGATTATGTTTATGTTCTGGTTCTATCAATTAGGTAATGGTGTAGATGTCATTGCCTGCTTGTATGTGTACTATTAAATTACCATGCACAAACAGAGAGGTTTCAATTTGTAATACAATGTCTAATTTTTTTATTGCATAGGTATTGCATACTGGGGCTGGAAACAAGAATGCCTTCAAGGCACTTATTGCTGCAGAATACAGTGGGGTCAAGGTTGAGGTGACCAAGAATTTTGAGATGGGTGTCTCCAACAAGACCCCTGAGTTTCTTAAGATGAATCCCCTTGGGAAGGTACACCATGCACCAATCATTATCTTTGAATTGCAATGTATAGTGGTTCATGAACTAAAAATTACTCTTCAGGTTCCTGTTCTGGAGACTCCTGATGGCCCTGTTTTTGAGAGTAATGCTATTGCACGCTATGGTATATATAGGACCCCACACTTTTTCAATTTAATTTTTTCTTAACTTTTTGCCGCTGATGACCATATCTCTGTTTTCCTTTCAGTTGCTCGTTTGAAGGACGACAACCCTCTTCTTGGATCTTCCCGTATTGAACAAGTGAGTGATTTATCGAGTGTTAACATAGTTAACTGAGTTTTACTGCTTGTGAACCTCATCAATGGTTCTTGCAGGCCCACGTTGAGCAATGGGTGGACTTTGCTGCAACAGAAGTTGACCCTGGTGTTGCATGGTACTTGTATCCAAGGCTTGGGTACATCCCTTATGCTCACACAGTAAGGGCTGGGAAACTTGGTTTTTGTATTATTGAACATTTACTAGCTATATTATTAACCCTTTTGTGTGTGTGTGCAGACTGAGGAAACAGCTATTGCTTCATTGAAGAGATCGCTTGGAGCTCTGAACACACACCTTGCCTCAAACACATACCTCGTTGGGCATTCTGTTACTCTAGCTGACATTGTATTGACATGCAACCTCTACCATGGAATTGCACATATCTTGACCAAGAGCTTCACTTCTGATTTCCCTCATGTTGAGAGGTATTTCTGGACCATGGTTAACCAGCCTAACTTCAAGAAGGTCATTGGTGAGGTCAAGCAGGCAGAGTCTGTACCTCCTGTTCAGAAAAAGGCCGCTCCTCCTAAGGAGCCGAAGGCAAAGGATGTCAAGAAAGAAGCCCCAAAGGAGGCCCCCAAGCCAAAGGTGGTTGAGGCACCAGCAGAAGAGGAAGCACCAAAGCCAAAGCCAAAGAATCCTCTTGACTTGCTGCCACCAAGCAAGATGATCCTTGATGACTGGAAGAGGCTATACTCAAACACAAAGACCAACTTCCGTGAGGTTGCTATCAAAGGTATCATCTCCATGTTTTCAATAATATGGTCAATATGTTTGCTTCTTCCCTAACAATGTTTAACTGTGATAGGATGGTGTTAGAAGTCTGTACTTTGCAATCTATCAATCTTACAGGAAAACAAATTTGGGAGTCACCTTCCTGACTAGATTTTGGTTTTGGCCTTGTAATATTTTTTAGACACCTGAAGGATTATGCACCCTATAGCTGAGTCAAATCACAGATAGCCACAAAGAGTTTATGTCGACGTGAACTAACAAAGTGCTAATAGTAATTATGTATCATGTCTGCTTTAATAAGCAGTTACTTCGTCATCCATTTGCCACATGAATTTACTAGTCCCTCCATTTTGCTAGGACAAGCATTGGACCAACAGTTTTTAATAAGCAGTTACTTCGTCATCCATTTGCCACATGAATTTACTAGTCCCTCCATTTTGCTAGGACAAGCATTGGACCAACAGTTTATTAATACATAGTTTGTGACAAAATTATGCAATTAGATTCATATTTATTTAAAATATATGTAGTTATGGTTATAATTTTGTATAACAAAAATGAAGTATTACTGTGGTAATTGCCTGTCGAAGCCTCGTGCTATTGAAACAAAATACGCCTTGGAAAGAAAAATGGAGTACTGTGAGTAAATTAATTAGATGTTGGATTTCCTTTCCTATCCCATGTTTGTGTAGTCTTGACCAAATAATCTGTCTGTGTTTCAGGTTTCTGGGACATGTACGACCCAGAGGGCTACTCTCTGTGGTTCTGTGACTACAAGTACAATGATGAGAACACTGTCTCCTTTGTGACCCTGAACAAGGTTGGTGGATTCCTGCAGCGGATGGACCTGGCCCGCAAGTACGCCTTTGGGAAGATGCTCGTGATAGGCTCTGAGCCACCCTTCAAGTTGAAGGGCCTTTGGCTCTTCCGTGGCCAGGAGGTTCCCAAGTTTGTAATGGATGAGGTCTATGACATGGAGCTCTACGAGTGGACCAAGGTGGACATCTCTGATGAGGCCCAGAAGGAGCGAGTCAATGCCATGATTGAGGACCAGGAGCCCTTTGAGGGTGAGGCCTTGCTTGACGCGAAATGCTTCAAGTGAGTGTGCCGCCTGGATAGAACTGTTTAGCCCTTGAATTGGATGTTTATGGACGAGTTTGCCCTTTTACTTGTGTCTATGTTGATCTGGTATACCTTGCTATGAACTGGTTAAAGTTTGTGCTGGTTTGAGATTTTTTGTCTATCTTGTGGTACCGTAGTCTTCCTCATTTAAATGTTTCGCCGGAACAATCTGGTCATGGTTTTTTTGGCTTATATCTGAGATGTTGTGATTATTGTTGAAAAAAAACATGCAAGAACTTGCAAGACGACAGAGCCATTTTGGAAAGCCTGAGTTTTGATCTAGGAAAGCCTTCTAGGACTAGTATCATGCCTTAAGCAGAGTTTTAATCCGAGCAGTAGTTGGTGCTATTTGTGCAAACTATTCGGTTTTATCAGATCCAAATGGTATTATTCTCAAGTATTTTAAGTTTTAATCTCCCAGGAAGGTCAGTTCACGACATAGTTAGCGACAAGTTGGTACATCTACAGTGACGGACAAGTTGGTACATCTCCAGTGACGGACCAGGTGAAAGTGGGATGAGCGACGGCTTAAAGCATCTGCAAATAGCACAAATTTTGCCGGTGACACTGCGCCAGTTCTACTCAAATAGTCAATACTAACAACATCCGATGGCGAAATTGCCTTTACTACAACATAGATATAGTGTACAGTGATGGGGAACCACATTACATATTCAACAGGAAGGGATAACAGAAGGGGCAAACCAAAAGAACAAAAGGTGAATTCCTACGTGGTCACCCTGTGTATGGTTCTCCTTGTATGGGTTCCAACTTCTAATGAGCTATCAAACAGTTTCACACCAATCTCACTTCCCTGCAAGTAGCTTAACATATTCTTGTTGCATAATGAATGATACTCGTCCATTTTATGTTGATCCGAATTCTTGAACTCTTGCGATGACAGCCTCATGGTCAGCAATTTTTGTATCAGATGAGGAACACACAGCAGCTTTGAGTAGGATCAACTGTAAAGAAAAGATAAACTTGTCAACAAAACACTTCATACCAAAGGGGAAAGTCTAATAGTCAGGTTACTTTGGTAAAGATAGCGAGGATGAGAGTGCGGGAGCATACCATATGTCAGTTTTTAACCAAAAGTTCAGAAGTCATATTTCGTCCGGCCACTTCTCAAGATTAGGCATAGCATATCAGATAGCTTAGAGGTTGAGTTGTAGAAATTCCATGCCTCAAGCATCAAACGCTTTCTTGAACCTTTGAAATGGACAATCTGAAAACGGTACAAGAATTATTTTGGATGTCACAGTAAATAGAAAGCATATACAACAATGTTACACAGCATGCACATTTATTGGATTAGTATAATCCTGCAAGAATGACCCACATTTGCATGCTGACGAAATGGTCATCAATCGGAGCATATAGCTTATAACAAAAGAAAACATCATGAATCCACAATACACAGTATTATCACAATACCTAAAAATTTATTTGAATTATAAATGCCATACATGCGAGAAAAAGAAACGGTGAACCAATCTTCGTAGTTTTCACATCTATATTAGCATCATGTATTCATGTGTAAGGAGCCTGCAGAATACTATTTTCTATTAATGTTAGGCCAAAATTTATTTCCATGAAGAAAAAGAGGAAAACTTTGACCAATTAAGTTCCTTACAAGTAAGAAAAGTGTTATACCTTAACATCCAAGGGTATACCATGAAACTGTCCAGCACCCTCAGGCGGGGTCCAATTATAAACAGCACAAGGCAAGAAGAGAACAGATGCTCCATTAACTTCACCAGTAAATGCTTCATGCTTAGAAAATTTTCCAAAAGCCGATGGTAGATGAGACTTGACAACCCATGCCAGTGCTAATTGGTCACCAAGCATACGGGAAGCCTTGATATATCTTGAGCTGTAAGCTTCAAGGACTTGTTTCAAGAATTCCACAGCTCTGAAAAAAGAGAGTTTAATTTTAAGTTTGCCAGCTTTCTACAATACGATTTATTCAATAGCTGTCAACTTACTTAGTGATGCCATCCCTGGTTCCTCTTACCGCAACAAACCCAGAGTTCAAAGGTTGCCCTTTGTTATTACGAAAAGTAAGAGCCAGATGACAATGGGGATATTTTTTGAATATATGTCCAAGATCATCAACCACTGCTATATCGGAATCAGTTAAAACAAAATGATTCAACCCCTCCATCCTATCAAATTCCACAAGTTTTTGCTCCAGAAATGCCTGAATATAAGGATAAGATACATAAATATAATTCTTACAGTTGAAAATAATGCATAAAGATGATGATAGCAGTAACACATGGATCACATTAAAATTAATTTTGAGAAAGAATATGCAGAAATTTTAACGATCTAAATGAAGCCAATAGACTTACAATGTATGTCTTTATTCTTTGAAGCATCAAATTTCCTCGAGAATAGTTTCCTTCAATAGGCAATAGTGTAGCACTCCCTTGATTTACTGAAATTTTTGAACCAGGATCAGTCAATATGATCACGTTGCTTCTTGGCATTGATACCTGCAGGGTTCTACAGATAGGATCAGGTGTTAAACAACTCACATCACAATTGTTGTTGGAAATAATGTAGCACACTGTATATTTGCTATGGGGCATAGGTAACAAGATCATGAACTCATACGTCCTTTCTAGAAAGGGTTATGATAGTGATGTTAGTATTCACATTCATTATATTATCATGTTTTCCTACCACACAAAAAGGATAACAATCTCAACAATAGGGACAAAGTACCAATTCTTTAGTAACTCTAATAAACAGGACTGTTTGATAGCAATCACTCACAACATGCAATAATGCAAGACTTGGAAAATCTTAGTTGAAATGCTGGATATGCTATCATAAACATAGGGATGGAACTAGAATTTCAACTGTAATTCAAATATATGGGGTGTCCTGCAATCCATCATTCACAAGAGAGAGAAAATTATTTGAGCCATCAAAATGTTAGTGTTAAACTTGCCACCAGTAAACAAAATTCACACAAACTTAGAGAAAAGGAAGAGGTACCTTTATGAAACTGATGAATGTGTTCAGAATGGCCATGGATCTTTCTATTTTGCTATAAGAATGGTTTCCAACAGTTACAGAATCAGATGGAACATTGCCATCTCCAGCTGGGACAGAATTGTAGGTTGTGAATACAGTAACAAAAGAAATGTGTTCTCCAGAATAGTTCTCTTCACTTGATAGGCCAATCTTGTTCAGAGCTCTAAGTCCTGAACAAAGTGACAGAAACATCATCAGTCATATGAACTCAATCACCAGGTTATGTTGTGCCAACAGCAAGGAGGTACTGATTTGAGAAAGACAGAATGCAATCTACCATTATTAAGTTAAGATTACAGAACATCATTGACACCTATAAGCAAATTAACAACTATTTAAGATCTAAGTCGAGCATCATTACATCAAGATTCTAGATTTATCTGAAACATGCTGTTCAAAGCAATGCATTAGAGAAATTATTTTACTGCTCCTGATGAACTGATTTTGCAAAAAGCAACATTCTTATCCTGATTTTGCCTCACTTTGTCACAGAAAAACAAAGGCTTCCAGTTTGTTAGGCTGTAAATCCAGCTCAGCAAAATCTAATATTACAGTGGCAGAGCAAAAATGCAGAATGAGCATATAATCGTATCCCCATCATTTTTTTTCCTCAAAAAGATAAATATTACCGTGTGATGGCATAGTAAAACGATTCCAGTGCATGATGAATAAACGAGCTAACTTTTGACACTTTATTAGTTGGACATGGACTATCGAATGTGAAATTTGTACCCTCAACACAAAACATTCCATTGCCACAATGCCATTGGTAACAGTAGCTCAATTTTCAAAGCAATCCTCCGTGTCTAGACAGCCCTAGATGTCTCCAACTAAGATCTAAGCAGCCTCCACAAAGACCACTAAATTCAGCATGAATGGAGCTTGCCGAAGCGAGAGGATTAACCTTGGCACTGTAGGCGGTCGGGGCGGCCCTGTCCGGCGGCGGGGCCGAGCACGAGACGGTCAGGCCGCTTCAGCGCGGTTGGGGGTGGCAGGCACACGCCCTTCTCCGGCGCCCAGGGGTGCAGCCTAGACACTGGCGCGGATTGGATCGTCAGGGGGGTCCTCATTGTGAGGGGAATTGGCCGACAGAAAGGAGAGGTCCGTACCGGAGTAGATGAGCGGGACGAGGAGGAAGAGGAGCAGCGGGCCTCGACGTACGGCGCGGTTCCGAGGCCTCGGGGCCGCCATATAGGGGCGAAAGAGGCGGCAGCGGAGCCCCGGTCCCGTCGGGGGATGAAATCGCGTCGGCTTCGCCGACGGCGGTGGTCGGTGGGCAAATGATAAGCGTATTGCAGATATACGTCAACACGTCATCAGCTTCCGCAGAATATAAGTCAATCAAGATTCAAACCGTCCAACGTTTGATCAATTTGATAATGTCAAATTTGTATCAAAATAATAATAGTCATAAACAGTATATCTAAATCTGTGCATACCTATAATATAACAATAATAATATTAAAAGGTGCCCTCACACTTTTATCCATCCGTCTATCCTACCACATCTCATCATTCACAACATAAATTCAAATTCAATCCAAGATCAGGTTGAGAGCTTATTTTTAATTCCTTCACTTCTTTATAGTATATAGATAGATAGATTAACGCGCATCTAGCCACATCACATTCTCAGACGTATTAAAATGGATGACACAATCATTGCATAAATCTATCTCGTTGCAAAGTATAGATATATTTTCTAGAATATAAAATAAATGAATTAAGTTAAAGTATAACTTGAAAGACCATCTCATGTCAAAGTATAGCAGATAAACAAATTTAGAGGAAATATTATTTCATGTCTGGCCTTGACAAATGAAATGCCATTGATCTTTTATTCAAGAAATTGTCCATGTTTCCATGTGGGCTGAATGAGTTTTACTTTTTTTACTATTAATATTGCATTCCCATGGAGTCTTTTGCTCTAAAACCTTAGAACCATTTTAATTACTACCAAAAAGTAATTATTACCACCATTAATCACAAATTAAATGCATTTTGGATGGATTTTGACTTGTAGTTTGTTATATTGGTTTTCATGTTCAGAATGTGCGAAGCCCAAATCACTCCTGGTGTAGCCACTAATCCAAGGAGTCTTCATGGTCTCTAGGATTAGTAGGAGGCAAAATTAAGCTGCACACGAGCTTACAAAATTTGCTTTTAGTTTAAGAGAGAATCGAGTTTACTTTTCTTTGTTTCATGAGAGTGTTAAGGCACTCGTTAATAAAGCTCTCTTTTCTCGCAAAACAAAAGATCACCACTTGCCTTTATAACAATAGCCTATGTAACACTCATTGTTGAACTTTCTCTCGCATAACGGGACTAAACTATCATTGTCCAGTGGTAGATCCAGAAACGGAGCACGAGGGAGGCTAATAATGAAAGTCTTCACAAAAAGGCCAAGCCAACAGTCAATTTGGTTTTGATAAAGAATTTAATTCAAACATTGCATTAGGTAAGCCTGTTTTGCTAATAACACAAATTTTGCATTTGGTTTAACATTAACTCCAAGTTAATGAACTTGGAGTTAATTCAAACAATTACATTAGTTAATAACATTGCATTACACGTAATTTGCATTCGGTAATAAAACCTGTTTTATAGCAGCAATTTGCAACATTGCAGTTAAAACCTTTCTTCATTGAAAACTTTGACCTCGCATTTGATATTTAACAACAAAGGTAGTAGTGAAAAAACTAAGTTAAAAGTGAAACACAAGCTTGAGAAACGACTAATTCACAACAAAGCAAGTAAAGGTCGTGATCTGCGAGTCTGCAAATTTCTCCATATTTTTGTAAATTAGTTCCTGCTCAGGTCTTGTGGCGAATTGCATCTTCAAATAAAATGCGAGGATTAGAATAACTGAAGAAGAAGAAATAACACGTGTAGAAGAAGTTATACATTGGTCTTGGCCATAACCAAGTGTACTAGGTGAGGCTTGATCATCACTTTCAATTACACATTTGGCATTCCTCTAACCCAAACTAAATCAGAGCGTCCACGGATAGACTAAAATCATACTTAGCTATTTTCATGATAGAGTCAACATTAACTCCAAGTTATTAACTATAATTGTCGTTGTTAGCCCTTCCATACCCTCTTGTATGGTACTTCCTAATTTTTAAATTATAAGATGTTTTGGCTTTTCTAGATACATAATTTTTTGCTATGCACTTACATAACTAAGTTTACATACATATTAAAAAACAATGTATATAGAAAAACCAAAAAAGTCCATCTAGTCAAGCCCAATTAACTAGTTTGAATTGAGCCTCACAATCCACAATAATATATACGACATAGGTCTAATTCAAATTAGGTCAAATGTTAGCATGCTACATGAACAAACTATTTAGTAAACCCCTCTTCGT
